# Dynamic Expansion and Functional Evolutionary Profiles of Plant Conservative Gene Family SBP-Box in Twenty Two Flowering Plants and the Origin of miR156

**DOI:** 10.3390/biom10050757

**Published:** 2020-05-13

**Authors:** Jing Li, Xiaoyang Gao, Xuan Zhang, Changning Liu

**Affiliations:** 1 CAS Key Laboratory of Tropical Plant Resources and Sustainable Use, Xishuangbanna Tropical Botanical Garden, Chinese Academy of Sciences, Menglun, Mengla 666303, Yunnan, China; lijing2@xtbg.ac.cn (J.L.); gaoxiaoyang@xtbg.ac.cn (X.G.); zhangxuan@xtbg.ac.cn (X.Z.); 2 College of Life Sciences, Univeristy of Chinese Academy of Sciences, Beijing 100049, China; 3 Center of Economic Botany, Core Botanical Gardens, Chinese Academy of Sciences, Menglun, Mengla 666303, Yunnan, China; 4 The Innovative Academy of Seed Design, Chinese Academy of Sciences, Menglun, Mengla 666303, Yunnan, China

**Keywords:** gene family, flowering plants, SBP, synteny, duplication, miR156, coevolution

## Abstract

Conservative gene families in plants, which are closely related to innovations in flowering plants, have long and complex evolutionary histories. Here, we used the *SQUAMOSA* promoter-binding protein (SBP-box) gene family as an example to study conservative gene families in flowering plants. In total, 11 groups, including nine angiosperm-conservative groups and two monocot- and eudicot-specific groups, were identified. Among the nine angiosperm-conservative groups, four are conserved in all land plants and the remaining five are angiosperm-specific. The five angiosperm-specific groups exhibit structural and functional diversity and evolved together, along with the evolution of flowering plants. The expansion of *SBP* genes was affected by miR156, and the miR156-regulated *SBP* genes tend to retain more copies. Our results reflect the dynamic evolutionary process of the different groups, with the identification of two genetic lines via synteny analyses. In addition, miR156 showed a close evolutionary relationship with *SBP* genes, suggesting that it may originate from face-to-face tandem duplication of *SBP* genes. *SBP* genes without an miR156 binding locus are usually functionally conservative or housekeeping like, belonging to the terrestrial-conservative group. In contrast, *SBP* genes with miR156 binding sites are selected by angiosperms to regulate more complex physiological processes.

## 1. Introduction

Angiosperms (flowering plants), with at least 300,000 species in multiple families, are the most diverse and latest-diverging group in the plant kingdom [1,2,3,4]. A gene family is a group of genes with the same characteristics, which usually contain a specific domain, motif, or special structural composition. Conservative gene families consist of genes whose sequences are conserved over time and are widely observed in many species. There are many conservative gene families in flowering plants. They appear in a large range of taxa and often play critical roles. Multiple types have also been found. For instance, some gene families are the main catalytic carriers of important biochemical processes in plants. The P450 super family has a large number of plant members and is the key enzyme for chemical synthesis [5,6]; some conservative families are important transcription factors (TFs), which, in plants, are structurally diverse and involved in many development processes and responses to abiotic and biotic stresses. For instance, the WRKY superfamily is involved in pathogen defense [7], senescence, and trichome development [8,9], and the MADS-box gene family is strongly correlated with the origin and evolution of reproductive structures such as flowers and ovules [10].

*SQUAMOSA* promoter-binding protein (SBP-box) (*SBP*) genes encode plant-specific TFs (SBP or SPL) that were first identified in *Antirrhinum majus* [11]. They were named for their ability to regulate the expression of *SQUAMOSA* (a kind of MADS-box) genes. Several characteristics make SBP-box a representative conservative gene family in plants. First, group members have a long evolutionary history. *SBP* genes have been identified in green algae, bryophytes, and angiosperms [12,13,14]. In addition, the SBP-box gene family has evolved many groups [12,13,14,15]. In a previous study, we showed that the *SBP* genes of Euphorbiaceae can be divided into 10 groups [16]. The SBP composition of different plant taxa is usually different [12,13,14]. In addition, *SBP* genes can be regulated by miR156 [17,18], and the evolution of SBP may be influenced by it. Finally, the SBP-box gene family is functionally diverse. Although it is highly conserved in plants, *SBP* genes in different groups may be specific to certain plant taxa [12,13,14]. These characteristics suggest that the SBP-box gene family is a very typical conservative gene family. Therefore, it would be helpful to study this family to understand other conservative gene families in angiosperms.

Over the last two decades, tremendous efforts have been made to explore whole-genome duplication (WGD) events. WGDs accelerate the birth and death of plant genes and have long been recognized as an important source of biochemical, genetic, and evolutionary novelty [19,20,21]. The moves from aquatic to terrestrial, from sporogenesis to seed reproduction, from gymnosperms to angiosperms, and from monocots to eudicots have typically been accompanied by WGDs. For example, all seed plants experienced WGD event ζ [22]; all angiosperms experienced WGD event ε [22]; and two important clades in angiosperms, namely, monocots and eudicots, both experienced WGD events (γ for eudicots and τ for monocots) [23,24,25,26]. However, it seems that flowering plants appeared abruptly and were soon the most successful organisms on the planet [1,3,19]. Harsh environmental conditions are an external factor that promotes plant evolution. In the Cretaceous, the Earth was dry and hot, with high levels of oxygen. However, around the Cretaceous–Paleogene (K–Pg) boundary, global cooling and darkness were the two main stresses. The following question remains: what role did conservative gene families play in the success of flowering plants? WGD events promote gene evolution and provide us with the clues we need to understand plant gene families.

The evolution of a gene family is a dynamic process. As changes occur in the physiological processes and phenotypes of plants, the structures and functions of a gene family may also be undergoing constant change. Typically, ancient WGDs in plants have been accompanied by key innovations [20], including the formation of vessels and flowering mechanisms, and the formation and evolution of many flower organs [21]. In a specific gene family, both the composition and function may change after a WGD [21,27,28,29,30]. The influence of different WGDs in different periods on a certain gene family may differ. At least 10% of all floral regulators in *Arabidopsis thaliana* can be traced back to the common ancestors of the angiosperm [31]. The TF gene families that are involved in responses to biotic and abiotic stresses tend to have retained large numbers of copies during the three waves of angiosperm WGDs [32]. The evolutionary trajectories of the different taxa of angiosperms are diverse. All eudicots experienced γ [23,24,26], while all monocotyledons experienced τ [25]. After τ, Gramineae experienced σ and ρ [25], unlike other monocots. Angiosperms also feature diverse physiological and reproductive processes and phenotypes among different taxa. Some plants are androgynous and others are diclinous; some plants blossom each year and others blossom only once in their lifetime. The gene families associated with these processes or phenotypes may experience a distinct evolutionary trajectory for different taxa. Therefore, the evolutionary process of a specific conservative gene family may be in a synchronous relationship with the physiological and phenotypic evolution of flowering plants. Hence, the evolutionary patterns of a specific conservative gene family during typical periods of its evolution need to be studied.

In this study, we comprehensively analyzed the *SBP* genes in 22 angiosperms and investigated their dynamic evolution to better understand the SBP-box gene family of flowering plants. First, 22 sequenced plant genomes, which represent major lineages of flowering plants, were used to predict *SBP* genes, and phylogenetic analyses were performed to obtain a comprehensive SBP-box gene family classification. Then, an evolutionary profile of *SBP* genes was created in phylogenetic analyses of angiosperms and other typical control species, providing a map of the evolution of the *SBP* genes. Moreover, the dynamic evolution of different SBP groups and the correlations between SBP and miR156 were assessed based on synteny relationships, and a possible model of the emergence of miR156 was developed. Finally, we performed a preliminary functional exploration of different groups of *SBP* genes by analyzing their upstream regulatory sequences, along with their tissue-specific and stress-treatment expression profiles. We performed comprehensive analyses of the angiosperm SBP-box gene family and its dynamic evolution to provide a reference for the study of other conservative gene families in plants.

## 2. Materials and Methods

### 2.1. Data Sources

The genomic and proteomic sequences of *Brachypodium distachyon*, *Ananas comosus*, *Sorghum bicolor*, *Panicum hallii*, *Oryza sativa*, *Musa acuminate*, *Setaria italica*, *Elaeis guineensis*, *Zea mays*, *Brassica rapa*, *Camelina sativa*, *Phaseolus vulgaris*, *Vigna angularis*, *Cajanus cajan*, *Medicago truncatula*, *Solanum lycopersicum*, *Capsicum annuum*, *Nicotiana tabacum*, *Prunus persica*, *Malus domestica*, and *Prunus mume* were taken from the NCBI database [33]. The genomic and proteomic sequences for *A. thaliana* were downloaded from the TAIR database [34]. The SBP protein sequences for *A. thaliana*, *O. sativa*, *Physcomitrella patens*, *Selaginella moellendorffii*, *Picea abies*, *Amborella trichopoda*, and *Chlamydomonas reinhardtii* were obtained from the PlantTFDB database [35]. The pre-miR156 sequences and mature-miR156 sequences for *A. thaliana* and *O. sativa* were obtained from the miRBase database [36].

### 2.2. SBP Gene Identification and Characterization

Both a genome-wide Hidden Markov Model (HMM) search and local Blastp were performed to identify possible *SBP* genes [37,38]. The HMM model of PF03110 was downloaded from Pfam [39]. A local version of the HMMER v3.2.1 program was used to perform the hmmsearch, and the E-value was set to the default value. The known sequences of *A. thaliana* SBP proteins were used as queries in the Blastp process, and the E-value was set to 1 × 10^−10^. The results obtained by the two methods were combined and left for later analyses after removing redundancy. To ensure the authenticity of the SBP domain, all retrieved sequences were tested using the SMART v8.0 tool and the NCBI Conserved Domain Database (CDD) [40,41]. Sequences with incomplete SBP domain structures were completely discarded. Some basic physicochemical parameters of SBP proteins, such as the protein length, molecular weight (Mw), and isoelectric point (pI), were predicted using the ExPaSy Proteomics Server [42]. Additional conserved motifs outside the SBP domain of all SBP proteins were identified by MEME v5.1.0 [43]. The settings for the minimum width, maximum width, maximum number of motifs, and minimum sides were specified as 6, 150, 15, and 2, respectively.

### 2.3. Phylogenetic Analyses and Detection Orthologous Genes

The SBP protein sequences of these 22 species were aligned using MUSCLE v2.0 [44], and the alignments were used for phylogenetic analyses. Both the Bayesian method in MrBayes v3.2.7 and the maximum likelihood method in PAUP* v4.0 [45,46] were implemented. The orthologs among these species were detected using OrthoFinder v1.0.6 [47].

### 2.4. MIR156 Prediction

MiRNAs were predicted using a homologous search based on mature miRNA sequence conservation in plant species. The mature miR156 sequences were downloaded from miRBase [36]. A total of 470 mature sequences were deposited in miRBase (Release 22.1). After all redundancies were removed, the remaining unique sequences were kept as miRNA reference sequences, and they were subjected to Blastn against the genome [38]. Output sequences > 18 nt in length and with less than three mismatches compared to the miRNA sequences were collected for further analyses. The predicted miRNAs of mature sequences, along with sequences 200 nt upstream and downstream, were used as assumed miRNA precursor sequences. If either the upstream or downstream sequence was less than 200 nt, the entire available sequence was used as an miRNA precursor sequence. Sequences in protein-coding genes were removed, and the remaining sequences were subjected to RNA secondary-structure prediction using MFold v3.6 [48]. All sequences met the criteria given in a previous study and could be used for subsequent analyses [49]. The sequence logo was created using WebLogo v2.8.2 [50].

### 2.5. MiR156 Target Prediction

The target sequences of *SBP* genes were predicted using psRNATarget [51]. All well-identified mature sequences of miR156 were downloaded from the miRBase database [36]. All miR156 mature sequences were put into psRNATarget as query sequences, and they were then made into searches with default parameters. The sequence logo was created using WebLogo v2.8.2 [50].

### 2.6. Gene Duplication and Synteny Analyses and Estimations of Synonymous (Ks) and Nonsynonymous (Ka) Substitutions per Site and Their Ratio

Tandem duplications are defined as two paralogs that are adjacent or separated by one non-homologous gene in the same chromosome, while segmental duplications are homologous and distributed in two blocks of the same or different chromosomes. Where each gene was analyzed as the anchor point, both 30 coding genes upstream and 30 downstream of the anchor points were used to form micro-fragments. Blastp searches were conducted between these micro-fragments and the orthologous fragments were those containing no less than 10 gene pairs from the same gene family with an E-value ≤ 1 × 10^−10^ [38]. The same method was used for synteny searches and segment duplication searches, although synteny relationships describe those between species. *Ka*, *Ks*, and their ratio in all of the duplicated *SBP* gene pairs were estimated using the KaKs_calculator v2.0 [52,53], following the MA model (which combines multiple computational models and averages them) [54]. It should be noted that only the *SBP* genes located on chromosomes were used for the exploration of duplications and synteny relationships. Considering that some genes show synteny with multiple other genes (one-to-more situation), we took the average of these *Ks* values. If a one-to-more situation came from different species, they were not averaged. In addition, according to the synteny relationships, a heatmap cluster was developed. Each synteny was counted once for the two genes making up that synteny. The R package Pheatmap v1.0.12 was used to cluster different groups and genes [55].

### 2.7. Conservative Analysis

All *SBP* genes from 22 angiosperms were regarded as the reference group, and *SBP* genes from the five species of *C. reinhardtii*, *P. patens*, *S. moellendorffii*, *P. abies*, and *A. trichopoda* were used as control groups. We adopted both OrthoFinder v1.0.6 search and phylogenetic relationships to find the sequences sharing a common ancestor for reference and control species [47]. A phylogeny analysis followed the above-mentioned algorithms. In addition, the HMM profiles of each group were created by HMMER and were used to find the homologous gene of each group [37].

### 2.8. Upstream Regulatory Element Analyses

At the transcription initiation site upstream, −100 to 2000 nt were collected. All of these upstream sequences were uploaded to Binding Site Prediction (PlantTFDB) to predict their possible TF-binding sites [35]. Then, the binding sites identified were transformed into the *A. thaliana* TFs with the homologous binding site. Finally, Gene Ontology (GO) analyses were conducted using the R package clusterProfiler v3.12 [56,57].

### 2.9. Tissue Expression and Stress Response

The normalized expression data for *AtSPL* genes in different tissues and under different treatments were retrieved from AtGenExpress Visualization Tool. To visualize the tissue-expression data, the normalized data were log transformed and presented using Pheatmap v1.0.12 (an R software package) [55]. To show the response-expression profiles of *SBP* genes under different stresses, we counted the number of experiments in which the value of the expression changed significantly (the expression value was double the control group or less than half) under each stress condition.

## 3. Results

### 3.1. The SBP-Box Gene Family Is Diverse in Angiosperms

To fully understand the SBP-box gene family in flowering plants, we selected nine species from four families of monocots and 13 species from four families of eudicots. The chosen monocots were *B. distachyon*, *A. comosus*, *S. bicolor*, *P. hallii*, *O. sativa*, *M. acuminate*, *S. italica*, *E. guineensis*, and *Z. mays*, and the eudicots were *B. rapa*, *A. thaliana*, *C. sativa*, *P. vulgaris*, *V. angularis*, *C. cajan*, *M. truncatula*, *S. lycopersicum*, *C. annuum*, *N. tabacum*, *P. persica*, *M. domestica*, and *P. mume*. Because *O. sativa* and *A. thaliana* are well-studied model plants, we took their SBP information from a public database. We identified the *SBP* genes of the other species to obtain more reliable SBP information (Table 1). A total of 522 *SBP* genes were obtained from 22 species, of which 310 were eudicots and 212 were monocots (Table 1 and Table S1).

**Figure 1:**
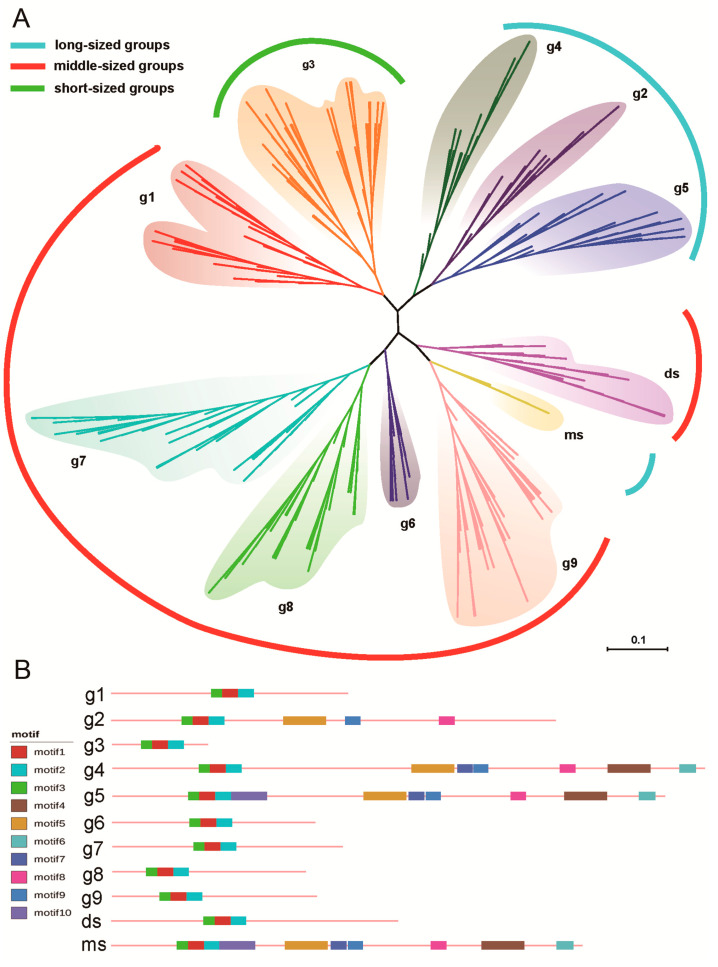
Phylogenetic grouping of *SBP* genes in flowering plants and the conservative motif structure in each group. (**A**) The phylogenetic tree of *SBP* genes in flowering plants. Eleven groups were obtained, namely, g1, g2, g3, g4, g5, g6, g7, g8, g9, ds, and ms. All these groups were divided into three length ranges (including the long groups, moderate-length groups, and short groups), and the groups that belonged to the same length range were marked in the same color in the outmost area. (**B**) The conserved motif of each group. The motif1, motif2, and motif3 together refer to the SBP domain.

We constructed phylogenetic trees including all 522 genes. To obtain more accurate results, the trees were constructed using both the maximum likelihood (ML) and Bayesian methods. The criterion for reliable results was that each clustered group contained the same genes, regardless of what method was used. Indeed, we obtained reliable results, with the outcomes of both methods matching; our trees also had relatively high bootstrap values (Figures S1 and S2). An unrooted tree (Figure 1A) was used to show the grouping scheme, according to the results of the ML process. In addition, the OrthoFinder v1.0.6 results suggested that the clustered genes were orthologous. These analyses resulted in 11 groups. The SBPs were concentrated into three length ranges, namely, the short group (g3), the moderate-length groups (g1, g6, g7, g8, g9, and ds), and the long groups (g2, g4, g5, and ms) (Figure S3). Most of the *SBP* genes were conserved in nine groups (g1, g2, g3, g4, g5, g6, g7, g8, and g9) of angiosperms, containing both monocot and eudicot SBPs. The monocots and eudicots also exhibited differences: one group (ds) was eudicot-unique, and one group (ms) was Gramineae-unique. In general, the SBPs of conserved groups from monocots and eudicots had similar physical and chemical properties (Table 2). Likewise, the SBP protein structure in each group was highly similar, based on conservative motif analyses (Figure S4). Due to the high similarities within each group, we display the most representative structure for each in Figure 1B.

Groups g1 to g9 were conserved in angiosperms, but their abundance varied between eudicots and monocots. We counted the average *SBP* gene number per species in each group (Table 2). Some groups had a lower abundance in both monocots and eudicots; for example, the average gene number for g2 and g4 was less than 2. Three moderate-length groups (g7, g8, and g9) were relatively abundant in both monocots and eudicots. Some groups exhibited significant differences between the monocots and eudicots. Group g1 had less than two *SBP* genes per species in eudicots, but nearly four genes per species in monocots. In addition, g3 contained more than five *SBP* genes per species in eudicots, but less than two per species in monocots. Most groups of *SBP* genes were conserved in all or nearly all species (Table 2), but g6 covered only 54% of eudicots and ms was only present in Gramineae.

### 3.2. Short and Moderate-Length SBPs Are Typical Targets of miR156

Because some *SBP* genes can be regulated by miR156, it is a key link for exploring the regulatory relationship between miR156 and SBP. We used mature sequences of miR156/157 (due to the high similarity between the two, they are both referred to as miR156 here) to search against the SBP mRNA sequences to determine *SBP* genes with miR156 binding sites. A total of 346 *SBP* genes containing binding sites for miR156 were detected. Moreover, we found that the binding sites in the *SBP* genes are not random. All long groups (g2, g4, ms, and g5) contained no target sites, while most of the other groups contained binding sites (except for g1). Genome-wide predictions of miR156, including all pre-sequences and mature sequences, were performed (Tables S2 and S3). All details of the miR156s are given in Supplementary Table S4. A corresponding prediction of the secondary structure was also performed (Figure S5). The length of the mature sequence for miR156 and its target site were both generally 20 to 21 nt. The target sequences showed a high consistency at each site, while the miR156 sequences had less consistency at sites 8–13 (Figure S6).

### 3.3. Evolutionary Profiles of SBP Genes in Typical Ancient WGD Events

**Figure 2:**
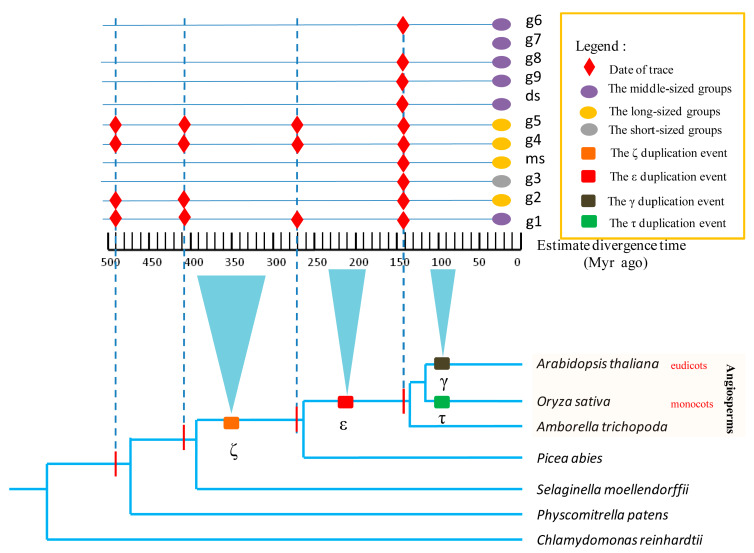
The evolutionary profiles of flowering plant *SBP* genes compared with other taxa of plants. The timeline is in the middle, and the approximate time range of each whole genome duplication event is shown in an inverted triangle linked to the timeline. The dotted line refers to the furthest traceable time of each species. The red rhombus on the cross of a dotted line and a solid line represents homologous genes of a group that were found in a species.

After obtaining the grouping scheme for the angiosperm SBP-box gene family, we investigated the phylogenetic relationships of this family in green plants to explore their evolutionary profiles. We carried out phylogenetic analyses of *SBP* genes between angiosperms (we chose *A. thaliana* and *O. sativa* as representative angiosperms) and control plants (Figure 2). All groups were far from aquatic organisms, because no homologous *SBP* gene was found in *C. reinhardtii*. According to the typical WGDs and the evolution of the main characteristics of angiosperms, three typical stages (the early evolution of angiosperms [ε to γ and τ], the differentiation of monocots and eudicots [γ, τ, and later events to the K–Pg boundary], and the recent evolution of angiosperms [after K–Pg]) were analyzed. The *SBP* genes of angiosperms showed different evolutionary profiles in these three typical periods.

In the early evolution of flowering plants, the ancestors of four groups (g1, g2, g4, and g5) were formed. All of these were homologous to *P. patens*, allowing them to be traced back to about 500 million years ago [58]. Some conserved functions could be assumed in these groups [59,60]. Many angiosperm-specific *SBP* genes emerged during the transformation from gymnosperm plants to flowering plants (after ε, and before γ and τ). Five groups (g3, g6, g7, g8, and g9) were angiosperm-specific. Like the angiosperms themselves, these groups appeared suddenly in the long history of plant evolution. Moreover, during the differentiation of monocots and eudicots, ds appeared as an independent eudicot group, while ms became a specific Graminaceae group, distinguished from other monocots. Although the SBP phylogenetic composition of monocots and eudicots was not significantly differentiated during this period, some eudicot and monocot groups showed obvious differences in their copy numbers (Table 2). However, monocot and eudicot groups also displayed similar retention tendencies. For example, g7, g8, and g9 exhibited high copy retention in both eudicots and monocots (Table 2). In angiosperms, some WGDs occurred in individual families or species. Many copies were formed during these WGDs, resulting in a large degree of gene redundancy.

### 3.4. Expansion Profile Describing the Dynamic Evolutionary Histories

**Figure 3:**
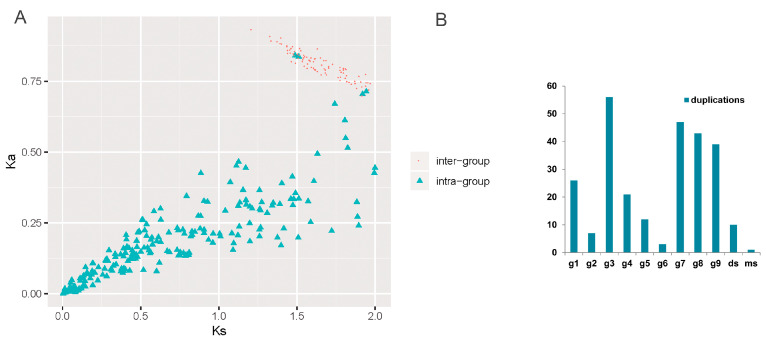
The selection pressure analysis of intra- and inter-duplicated *SBP* genes and the number of intra-group duplications in each group. (**A**) The X axis refers to the *Ks* value, and the Y axis refers to the *Ka* value. The inter-group duplications were marked with red dots, and the intra-group duplications were marked with green triangles. (**B**) A bar chart of the intra-group duplications in each group.

The inheritance and innovation of plant gene families is a complex and dynamic process. A deeper understanding of the dynamic process of evolution will help reveal the underlying mechanism of that evolution in plants. To this end, we explored the expansion of SBP by duplication. In total, 339 *SBP* genes were found in duplicate relationships. These duplicate genes consisted of many *SBP* genes (71%) (Figure S7A). Segment duplications played the most significant role in SBP expansion (Figure S7B and Table S5). Interestingly, both intra- and inter-group duplications were found (Table S5), which implies that some groups may have had genetic and evolutionary relationships. Selection pressure analyses showed that the intra- and inter-group duplication pairs were all subject to purification selection (Figure 3A). The *Ks* values of all inter-group duplications were greater than 1.2 (mostly earlier than γ and τ). In contrast to the inter-group duplications, 81.2% of intra-group duplications had *Ks* values of less than 1.2 (mostly later than γ and τ). Of all intra-group duplications, some miR-regulated groups had the largest degree of duplicated retention (Figure 3B).

**Figure 4:**
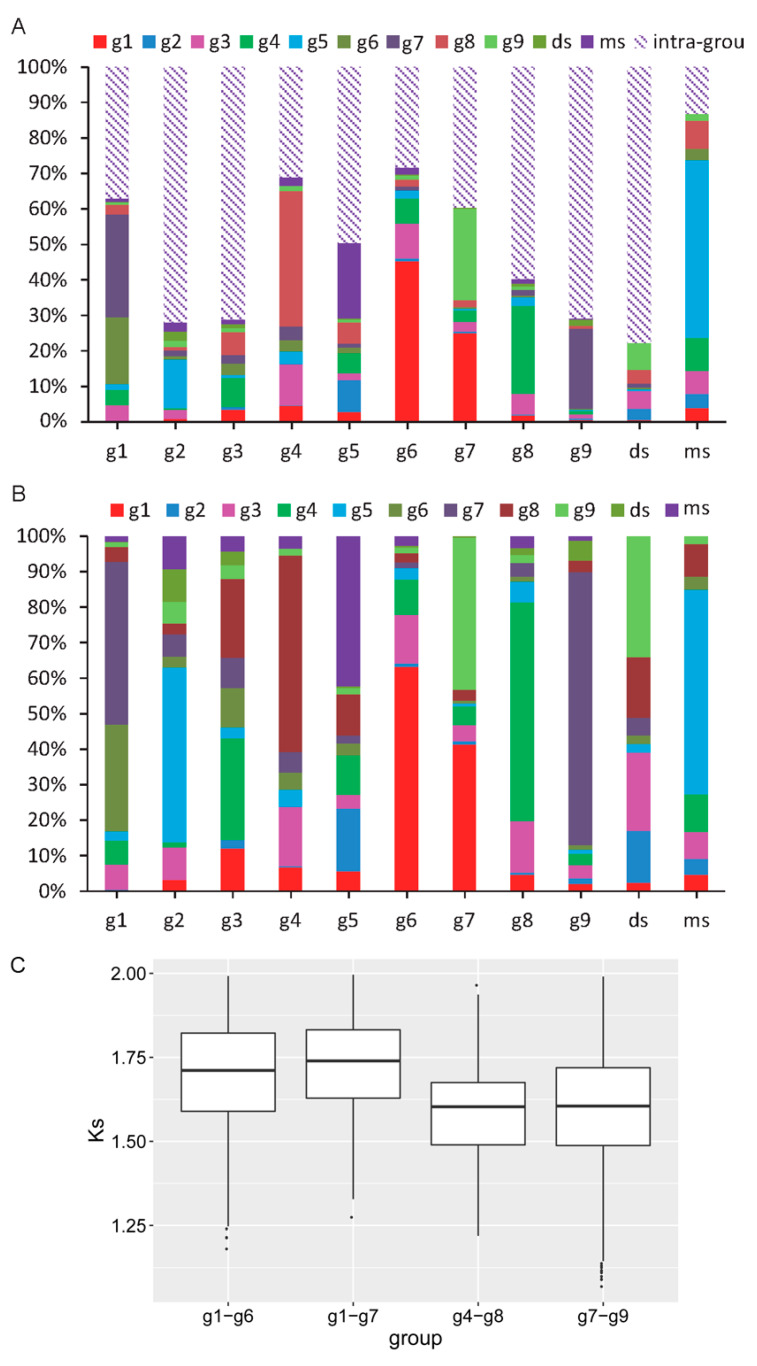
The synteny relations between groups and the *Ks* value of three pairs of groups. (**A**) A percentage stacked bar chart of all inter-group and intra- group synteny relationships. (**B**) A percentage stacked bar chart of all inter-group synteny relationships. (**C**) The *Ks* value of four pairs of groups with a relatively high number of synteny relationships.

These analyses provide genetic correlations between groups, but a higher-resolution correlation must be obtained to reveal dynamic evolutionary histories. Synteny mining among groups revealed clues that could be used to explore such correlations. All *SBP* genes which have been identified as being located on chromosomes were used for synteny analyses. As with the duplication results, there were both intra-group and inter-group synteny relationships. A total of 442 *SBP* genes had synteny relationships, accounting for 98.2% of all *SBP* genes (Figure S8A). There were significantly more intra-group synteny relationships (2999) than inter-group ones (1342) (Table S6.1,6.2). Any given group may have synteny relationships with multiple other groups, but the most relationships often appear among any given group’s own members (Figure 4A); this was the case for nine groups. Interestingly, the inter-group relationships were not arbitrary. These are presented as a stacked bar chart in Figure 4B, organized into eight groups in two clusters. Four with-target groups were directly or indirectly associated with g1. Group g1 had 30% synteny with g6 and 45% synteny with g7. In addition, g9 was closely related to g7, having 76% synteny with it. In addition, ds had more than 34% synteny with g9. In addition, two other with-target groups (g8 and g3) had strong synteny relationships with g4. Because the synteny relationships involved in each group are not all one-to-one correlations, that is, any given gene may have synteny relationships with multiple other genes, we conducted cluster analyses for all synteny relationships. The results support our previous analyses to some extent (Figure S9).

The *Ks* values for g4–g8, g1–g6, g1–g7, and g7–g9 were calculated, for they all produced more than 100 pairs of synteny relationships and provide greater statistical significance (Figure 4C). All of these *Ks* values mainly fell between 1.2 and 2, consistent with the duplication results (Figure 3A). For the four groups (g1, g6, g7, and g9) identified in the same cluster, the *Ks* values for the with-target groups and g1 (g1–g6 and g1–g7) were larger than those for the two with-target groups (g7–g9), suggesting that duplications between the angiosperm-specific group and the plant-conservative group mainly occurred before the expansion of angiosperm-specific groups. After the differentiation of the with-target group from the without-target group, the with-target group differentiated into other angiosperm-specific groups.

### 3.5. Coevolutionary Profile of miR156 and SBP Showing the Origin of miR156

**Figure 5:**
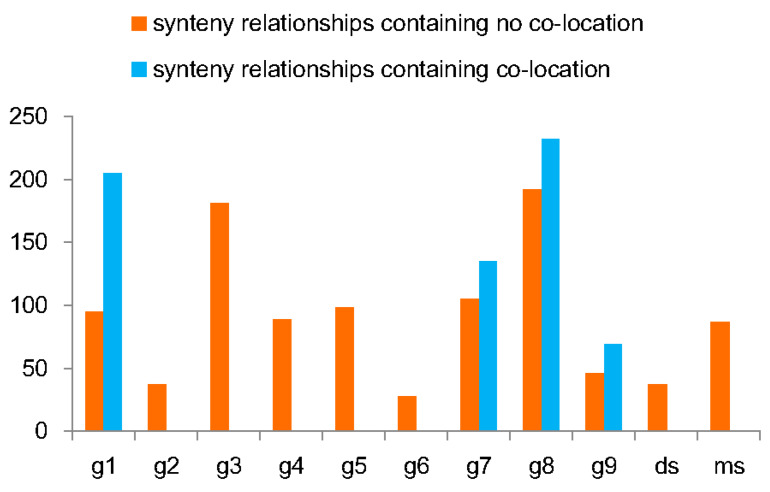
The number of synteny relationships between the SBP group and miR156. The orange bar refers to the number of synteny relationships without co-locations, while the blue bar refers to the number of synteny relationships with co-locations.

There may be evolutionary correlations between miR156 and SBP. We investigated this possibility and found that there were many duplications (Figure S7B and Table S7) and synteny relationships among miR156s (Figure S8B and Table S8). This makes us suspect that there are synteny relationships between miR156 and SBP. If so, it may be that their coevolution could be derived through these relationships. First, because of the possibility of the co-location of miR156 and SBP, we examined coincidence of genes in the micro-fragments formed by SBP and miR156. Co-location was defined as the co-occurrence of miR156 and SBP in a micro-fragment. After excluding all co-locations, we identified 161 miR156 genes (accounting for 73% of miR156 genes) with synteny relationships with 214 *SBP* genes (accounting for 48% of *SBP* genes) (Figure S8C,D and Table S9). Synteny relationships between miR156 and SBP were found in all groups (Figure 5). Some groups displayed a significant correlation with miR156 due to their relatively large number of synteny relationships. For seven groups, more than 50 synteny pairs were found (Figure 5). Co-location with miR156 was found for four groups (g1, g7, g8, and g9) (Table S10). These results further confirm their evolutionary correlations and produce an adjusted synteny number for these groups (Figure 5 and Table S9).

Next, we considered the formation of stem-loop structures. We found that the location of the genes of g1 and g6 provides natural conditions for the formation of miR156, such as AcSBP3 (in g6) and AcSBP4 (in g1), which are separated by less than 8000 nucleotides, and their 3′ ends are in the face-to-face form (Table S11). It is very interesting that up to 13 of the 21 g6 *SBP* genes had a gene from g1 located close to them (Table S11). In a previous study, we revealed that the distance between g1 and g6 in Euphorbiaceae is consistent with tandem duplication [16]. The data suggest that the same is true in this study for g1 and g6. In addition, as many as 10 of the 13 pairs of *SBP* genes are in the reverse direction. Furthermore, nine of the 10 reverse *SBP* gene pairs are in face-to-face relationships, which provide a potential structure for the formation of an miRNA stem-loop frame.

### 3.6. Functional Evolutionary Profile Indicating the Functional Differentiation of Each Group

**Figure 6:**
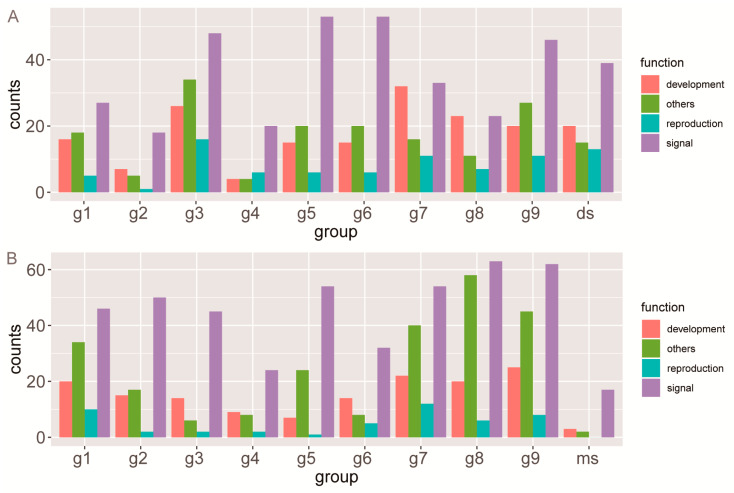
Gene Ontology (GO) enrichment of the predicted binding transcription factors (TFs) upstream in each group. (**A**) GO enrichment of dicots. (**B**) GO enrichment of monocots.

Gene transcription initiation is closely related to the upstream regulatory region. To predict the functions of each group, we predicted the possible binding TFs for each and performed functional enrichment analyses of the predicted TFs. To improve the reliability, we deleted annotations that shared fewer than three species in a group. Following the annotation for each Gene Ontology (GO) item, we divided all enriched biological processes into four categories (development, reproduction, signal, and other) (Figure 6 and Tables S12 and S13). Regarding dicots, almost all miR-regulated *SBP* genes were associated with relatively high numbers of GO-enrichment items in development and reproduction (Figure 6A). Similarly, most groups of miR-regulated *SBP* genes from monocots were also enriched in these categories (Figure 6B). However, the g3 monocot group (g3-monocot) was significantly different from the g3 dicot group (g3-dicot), which was enriched in many fewer items related to development and reproduction (Figure 6). In addition, the long groups, which are not miR-regulated groups, had relatively fewer enrichment items related to development (Figure 6).

**Figure 7:**
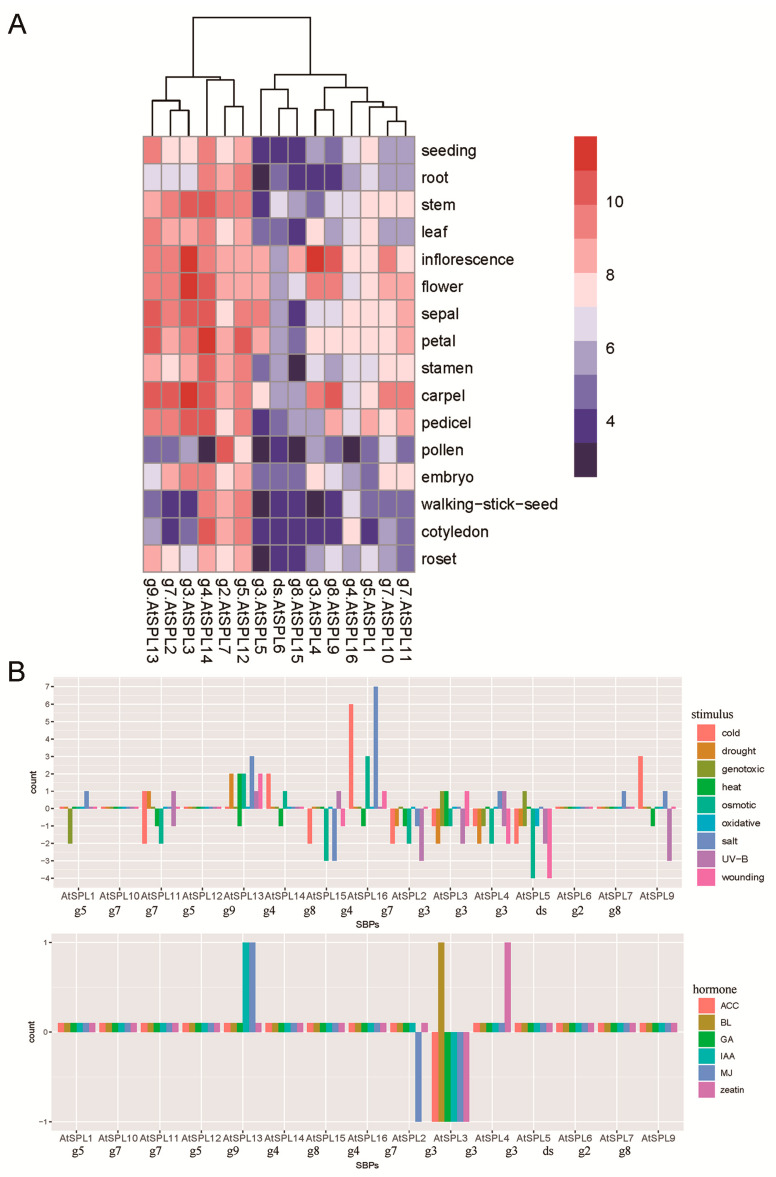
The expression profile of AtSPLs. (**A**) The tissue expression of AtSPLs. (**B**) The number of differential expressions of *AtSPL* genes in each stress treatment.

To validate these results, we analyzed *AtSPL* gene expression in various tissues (Figure 7A) and under various conditions (Figure 7B). Some miR-regulated *SBP* genes indeed showed a high expression during certain growth and reproduction processes (Figure 7A). *AtSPL3/2/13* (belonging to g3, g7, and g9) were highly expressed in multiple tissues, and they exhibited strong correlations with growth and reproduction. *AtSPL12/7/14* were highly expressed in nearly all tissues and displayed a housekeeping expression pattern.

## 4. Discussion

### 4.1. A Consistent Classification Standard Is Helpful for Exploring Conservative Gene Families in Plants

With reliable classification standards as a reference, it is simple to determine similarities and differences in the analyses of gene families. As genome-sequencing data for plants increase and bioinformatics tools are developed, the in-silico identification of gene families will grow. However, previous studies have often been limited to only a few species, lacking representativeness of the broad range of angiosperms. Some species may lack individual groups of a gene family. In a phylogenetic study of *A. thaliana* and *O. sative*, all *SBP* genes were divided into nine groups, but *A. thaliana* was absent in one [14]. Different studies may give different classifications. In analyses of *A. thaliana*, *O. sative*, and *P. patens*, all of the AtSPLs were divided into six groups [13]. However, in another study of *A. thaliana* and *O. sativa*, all AtSPLs were divided into eight groups [14]. The differences in the species and evaluation criteria used may have led to great differences in the classification results. We obtained a reliable angiosperm SBP classification through large-scale phylogenetic analyses and described the evolutionary characteristics for each group, providing a reference for further research.

### 4.2. Promotion of the Evolution of Angiosperms by the Differential Expansion of the SBP-Box Gene Family during Typical Ancient WGDs

A certain conservative gene family may have a similar evolutionary profile to a given plant. The origin of angiosperms can be traced back to at least the Cretaceous [1,2,3,4], and the ε event can be traced to about 200 million years ago [22]. During the Cretaceous, the Earth was dry and hot, with high oxygen levels. Some typical characteristics of angiosperms were formed during this period, such as the evolution of the carpel, the emergence of double fertilization, and the origin of the flower [61]. Likewise, the main SBP branches of angiosperms were formed during this period (the grouping scheme including g1, g2, g3, g4, g5, g6, g7, g8, and g9), and the angiosperm-specific *SBP* genes (g3, g6, g7, g8, and g9) all appeared in this period. The *SBP* gene promotes the adaptation of angiosperms to the environment and the evolution of angiosperms’ specificity. It is involved in the regulation of floral organ formation, transformation of the plant from the vegetative phase to the reproductive phase, the formation of plant morphology, and signal response and stress resistance [17,18,62].

WGDs provide impetus for differentiation and innovation in plants. During the process of the differentiation of monocots and eudicots, multiple WGDs occurred. Monocots and eudicots showed different expansion profiles, and the expansions of different types of *SBP* genes were also different. The number of SBP copies retained by monocots after τ was much greater than that after γ (which occurred at a similar time as τ) in eudicots [32]. MiR-regulated groups are involved in multiple aspects of plant reproduction, growth, and the stress response [17,18,63,64,65,66] and tend to retain more copies of *SBP* genes. After the γ and τ events, many TF gene families related to development, morphogenesis, and the response to abiotic and biotic stresses retained large numbers of copies [32,67].

In angiosperms, the innovation of the SBP-box gene family was not shared by a large range of plants, but it increased the functional complexity through the high retention of copies. The genes of some multiple-copy groups perform similar functions redundantly [17,18]. Some genes from different groups may have similar functions [68], and even copies of a group may be functionally differentiated [69].

### 4.3. Short and Moderate-Length SBPs as Important Regulators of Growth and Reproduction in Angiosperms

There are many conservative gene families in plants, but those with many functions and differentiations are more attractive to study. They react to changes in the environment and respond quickly. Some *SBP* genes, regulated by miR156, can be quite variable. Although they appeared much earlier than angiosperms [70], they are also continuing to evolve as angiosperms do. It appears that regulation by miR156 determines the fate of *SBP* genes. Groups without a target site are all conservative groups of terrestrial plants. Those with a target are all angiosperm-specific groups. The miR156_SBP module allowed *SBP* genes to adapt to more complex and variable regulatory processes and participate in more complex regulatory networks [65]. G1 appears to connect groups with and without target groups. The *SBP* genes in g1 were significantly different in length from other terrestrial-conservative SBPs; although their length is similar to that of angiosperm-specific groups, they do not have target sites. Moreover, instead of the functional housekeeping of other terrestrial-conservative groups, they are involved in angiosperm-specific physiological processes [68,71,72,73].

### 4.4. Dynamic Evolutionary Process Analyses Reveal the Expansion Process of Different Groups

**Figure 8:**
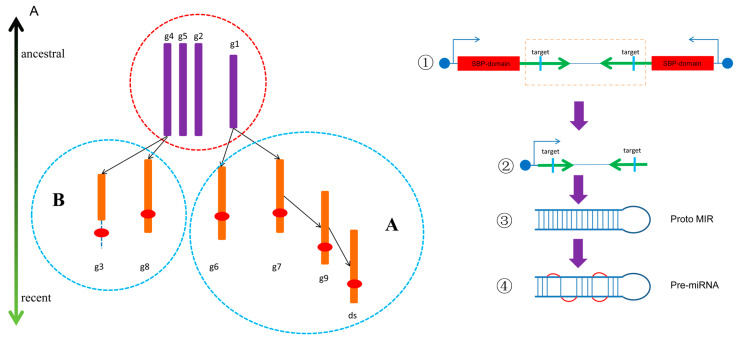
A derivation graph of the dynamic evolutionary process of *SBP* genes in flowering plants and the presumed model of the emergence of miR156. (**A**) A derivation graph of the dynamic evolutionary process of *SBP* genes in flowering plants. The terrestrial plant conservative groups are circled with a red dotted line. Angiosperm-specific groups associated with a terrestrial group relate to solid arrows, and the direction of the arrow represents the direction of evolution. Line A and Line B are circled by a blue dotted line, respectively. The red ovals refer to miR156 target sequences and the 3′UTR region is marked with a dotted line. (**B**) The presumed model of the emergence of miR156. ① The emergence of the face-to-face tandem duplication of two *SBP* genes. ② The formation of genes containing the ancestor sequence of MIR156 and MIR156*. ③ The formation of proto MIR156. ④ The formation of pre-miR156.

The emergence of a new branch of a conservative gene family in plants may be accompanied by plant innovation [21]. Gene families related to important physiological processes have been used to determine plant classifications [74]. However, it is difficult to understand the evolutionary genetic relationships between branches. The duplication of one branch by another is likely to leave synteny clues. The lack of exploration of these relationships has led to an overall poor description of inter-branch evolution in previous gene family analyses in multiple species. Using the inter-group synteny relationships, we made an inferential map (Figure 8A) of the dynamic evolution of the SBP-box gene family. Different groups of *SBP* genes do not originate independently, and with-target groups may form without-target groups. Although moderate-length groups have similar sequence lengths, they may originate from two lines: line A and B. Line A contains five groups, four of which are with-target groups and one of which is a no-target group. G6 and g7 may come directly from g1, and g9/ds may have been produced successively after g7. Line B contains three groups, and its two with-target groups (g3 and g8) may originate from the without-target group g4.

### 4.5. MiR156 May Originate From Face-to-Face Tandem Duplication of SBP Genes

The regulation module between a conservative gene family and its regulator miRNA is often traced to the origin of terrestrial plants. Very long periods of independent evolution make it difficult to clearly explain the relationships between an miRNA and its target gene. However, three prevailing models account for the possible emergence of miRNAs, namely, the inverted duplication of target genes [75], intermittent evolution [76], and inverted-repeat transferable elements [77,78]. Our research shows that there is another possibility for the generation of miR156. Based on our results, the formation of miR156 may be related to tandem duplications between g1 and g6, with a face-to-face relationship leading to the formation of a stem-loop structure (Figure 8B). Then, this stem-loop structure may have evolved further to obtain the precursor structure of miR156. In protein structure analyses, we found that moderate-length and short SBPs seem to be formed through the truncation of long SBPs. The ancestors of g1 may also have the characteristics of long sequences, but the formation of miR156 led to sequence loss in g1. There are still many unknowns about the formation of miRNAs, and much work remains to be done to reveal their generation and evolution.

## 5. Conclusions

Our results provide a comprehensive phylogenetic classification of the angiosperm SBP-box gene family. Moreover, we studied the evolution of angiosperm *SBP* genes, accompanied by several typical ancient WGDs of flowering plants, and presented the different evolutionary profiles of these genes corresponding to the WGDs. In addition, synteny relationships between different SBP groups and between SBP and miR156 were found, and the correlation between *SBP* genes and miR156 was elucidated. The dynamic genetic and evolutionary development of different SBP groups suggests two obvious genetic lines, and a possible path of emergence was proposed for miR156. Furthermore, an evolutionary map of SBP functional evolution was presented in a preliminary exploration of the angiosperm SBP-box gene family.

## Figures and Tables

**Table 1:** Source and number of *SBP* genes of each species.

Organism	Source	Number	Nomenclature
*Arabidopsis thaliana*	PlantTFDB	16	*AtSPL*
*Brassica rapa*	This research	29	*BrSBP*
*Camelina sativa*	This research	45	*CsSBP*
*Phaseolus vulgaris*	This research	22	*PvSBP*
*Vigna angularis*	This research	20	*VaSBP*
*Cajanus cajan*	This research	23	*CcSBP*
*Medicago truncatula*	This research	17	*MtSBP*
*Solanum lycopersicum*	This research	16	*SlSBP*
*Capsicum annuum*	This research	15	*CaSBP*
*Nicotiana tabacum*	This research	40	*NtSBP*
*Prunus persica*	This research	16	*PpSBP*
*Malus domestica*	This research	34	*MdSBP*
*Prunus mume*	This research	17	*PmSBP*
*Brachypodium distachyon*	This research	17	*BdSBP*
*Sorghum bicolor*	This research	19	*SbSBP*
*Panicum hallii*	This research	19	*PhSBP*
*Oryza sativa*	PlantTFDB	19	*OsSPL*
*Setaria italica*	This research	11	*SiSBP*
*Zea mays*	This research	32	*ZmSBP*
*Ananas comosus*	This research	17	*AcSBP*
*Elaeis guineensis*	This research	24	*EgSBP*
*Musa acuminata*	This research	54	*MaSBP*

**Table 2:** The protein physical and chemical properties and gene number of each group.

Group	Species Number	Sequence Number	Average Number	Average Length (AA)	Average Molecular Weight	Average Isoelectric Point
g1-eudicot	13	22	1.69	306.9091	34,068.8	8.913333
g1-monocot	9	35	3.89	405.4857	43,528.37	8.200286
g2-eudicot	12	18	1.5	722.4444	80,763.75	6.218889
g2-monocot	9	13	1.44	859.6923	95,320.8	5.746154
g3-eudicot	13	70	5.38	167.0857	19,179.53	8.482571
g3-monocot	9	15	1.67	203.2	21,441.35	9.813333
g4-eudicot	11	20	1.82	1031.25	114,075.8	8.3985
g4-monocot	8	12	1.5	1029.75	112,982.2	7.616667
g5-eudicot	13	35	2.69	987.7429	109,648.9	6.420571
g5-monocot	8	15	1.875	983	108,311.9	5.984
g6-eudicot	7	12	1.71	352.5	39,079.3	8.4025
g6-monocot	8	10	1.25	436.9	46,449.51	8.838
g7-eudicot	13	36	2.77	422.0833	46,785.88	8.303889
g7-monocot	9	34	3.78	426.6765	46,448.93	8.961471
g8-eudicot	13	29	2.23	363.1034	39,381.31	8.907143
g8-monocot	9	30	3.33	382.1	40,155.08	8.927667
g9-eudicot	13	32	2.46	368.1563	40,526.01	8.3625
g9-monocot	8	42	5.25	396.4286	42,237.75	7.825714
ds	13	36	2.77	496.0278	54,836.9	7.570833
ms	6	7	1.17	879.2857	98,183.74	6.792857

## Supplementary Materials

The following are available online at https://www.mdpi.com/2218-273X/10/5/757/s1: Figure S1: The phylogenetic tree reconstructed by the maximum likelihood (ML) method; Figure S2: The phylogenetic tree reconstructed by the Bayesian method; Figure S3: The three length ranges of SBP proteins; Figure S4: The conservative motifs for each SBP protein. Figure S5: The RNA folding structure of each predicted miR156; Figure S6: The conservatism of the miR156 mature sequence and target sequence; Figure S7: The number and type of duplicated genes; Figure S8: The number of genes in synteny relationships; Figure S9: The heatmap clustering of synteny *SBP* genes and groups; Table S1: The basic information of *SBP* genes from 22 species; Table S2: The mature miR156 sequence of 22 species from 5′ to 3′; Table S3: The pre-sequences of miR156s; Table S4: The basic information of miR156s; Table S5: Duplications of intra-groups and inter-groups; Table S6: The synteny relationships of intra-groups and inter-groups; Table S7: Duplications among miR156s; Table S8: The synteny pairs of miR156; Table S9: The synteny pairs between SBP and miR156. The red ones show the synteny pairs with co-locations; Table S10: The co-location relationships between SBP and miR156; Table S11: The co-location of genes from g1 and g6, and their directions; Table S12: GO enrichment of eudicots; Table S13: GO enrichment of monocots.
