# Supplementary material for: Dynamic Expansion and Functional Evolutionary Profiles of Plant Conservative Gene Family SBP-Box in Twenty Two Flowering Plants and the Origin of miR156
Source: Biomolecules. 2020 May 13;10(5):757. doi: 10.3390/biom10050757 (PMC7277735; doi:10.3390/biom10050757)
Supplement: Supplementary file 1 [file biomolecules-10-00757-s001.zip › Supplementary Materials/Figure S2.pdf]

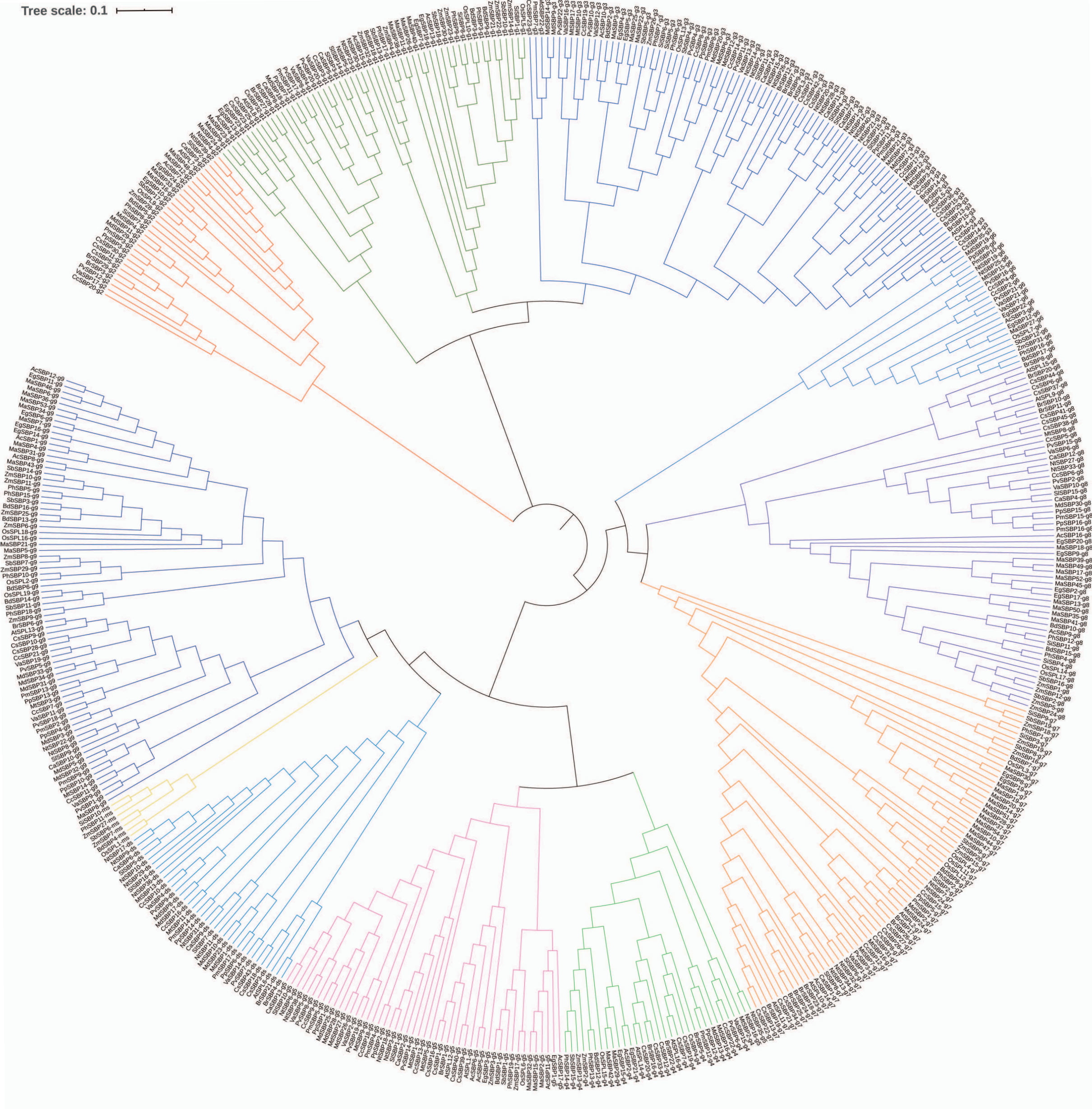

Figure S2: The phylogenetic tree reconstructed by the Bayesian method. Each group was highlight in a specific color.
