# Supplementary material for: Dynamic Expansion and Functional Evolutionary Profiles of Plant Conservative Gene Family SBP-Box in Twenty Two Flowering Plants and the Origin of miR156
Source: Biomolecules. 2020 May 13;10(5):757. doi: 10.3390/biom10050757 (PMC7277735; doi:10.3390/biom10050757)
Supplement: Supplementary file 1 [file biomolecules-10-00757-s001.zip › Supplementary Materials/Figure S5.pdf]

Figure S5.The RNA folding structure of each predicted miR156

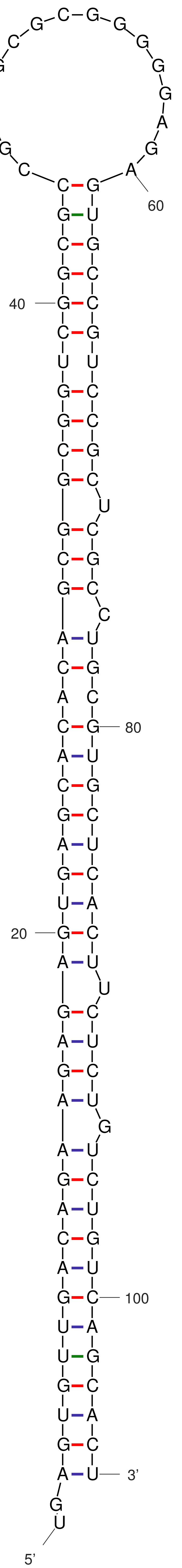

dG = -66.50 [Initially -66.50] Ac-miR156_1 NC_033622 1

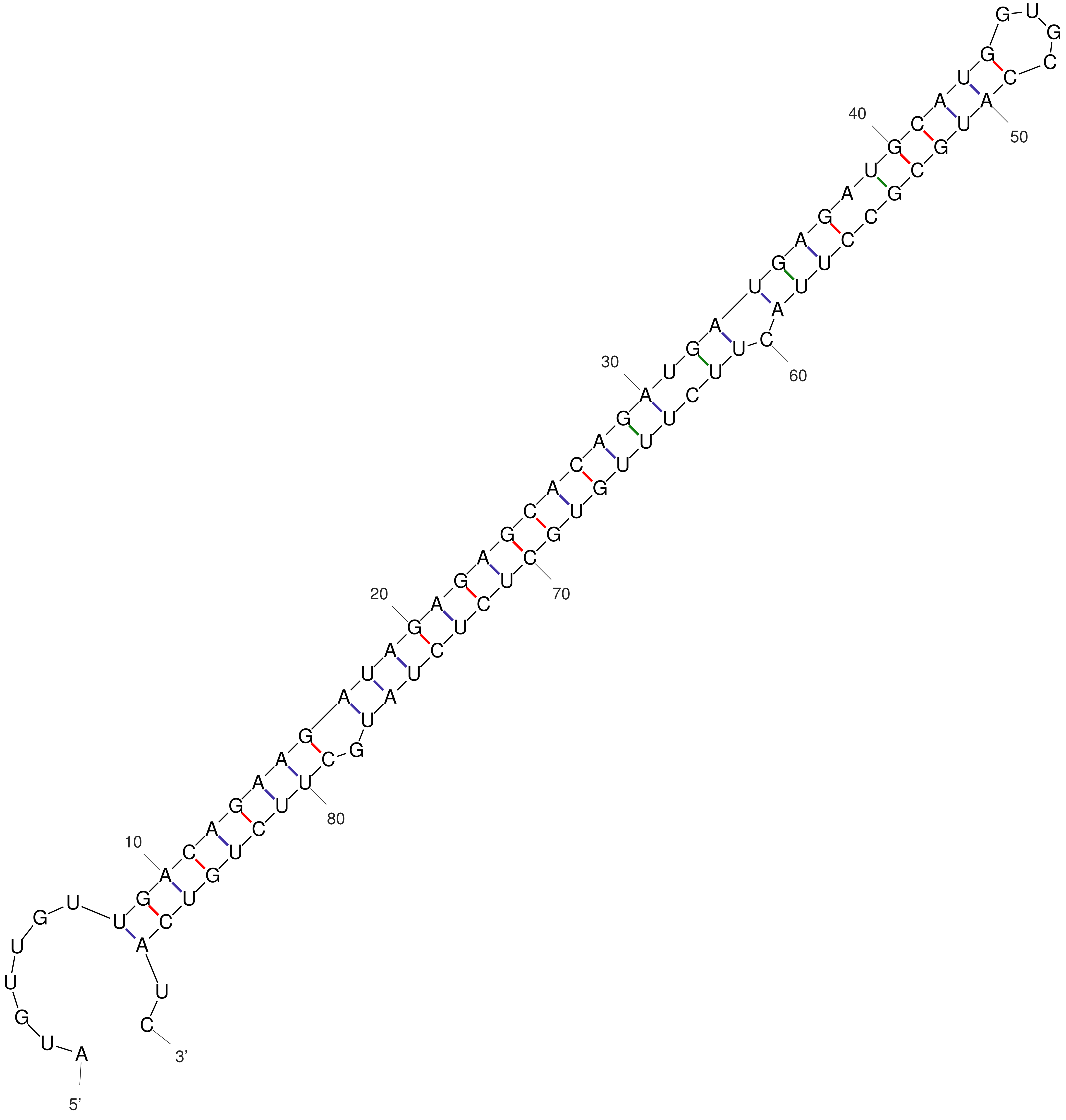

dG = -48.00 [Initially -48.00] Ac-miR156_2 NC_033625 1

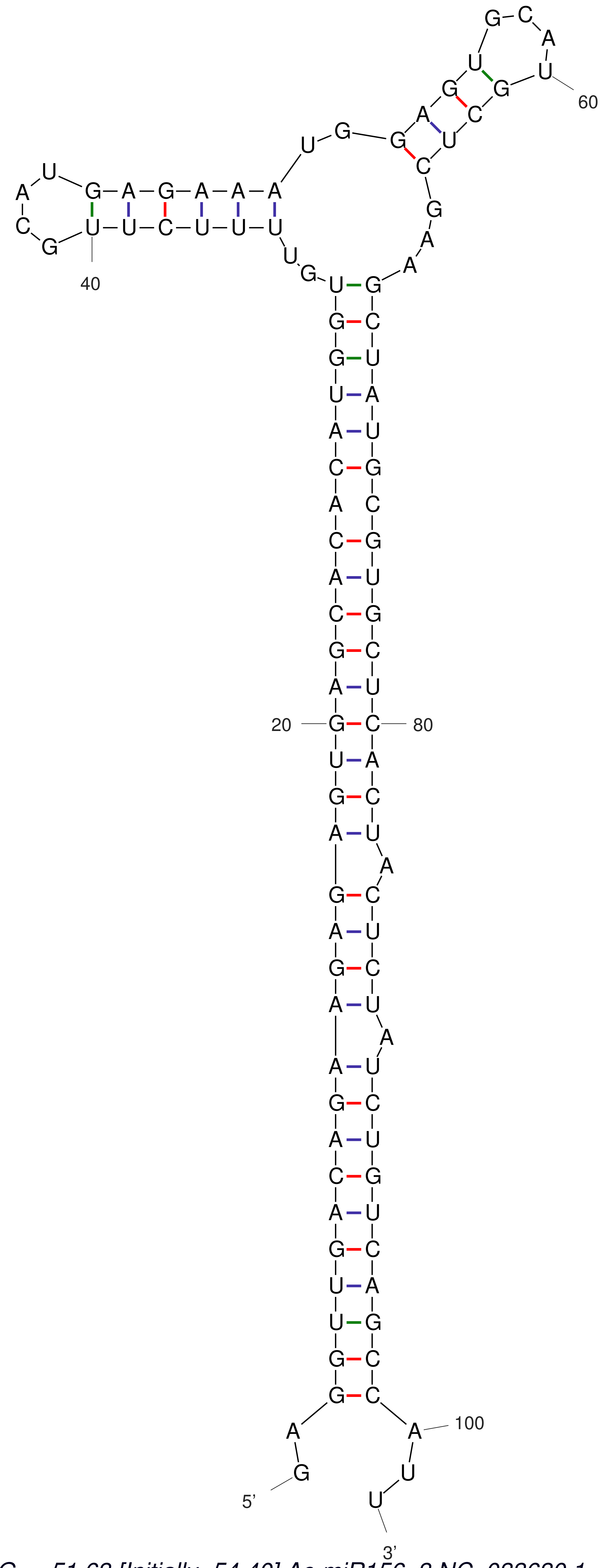

dG = -51.63 [Initially -54.40] Ac-miR156_3 NC_033630 1

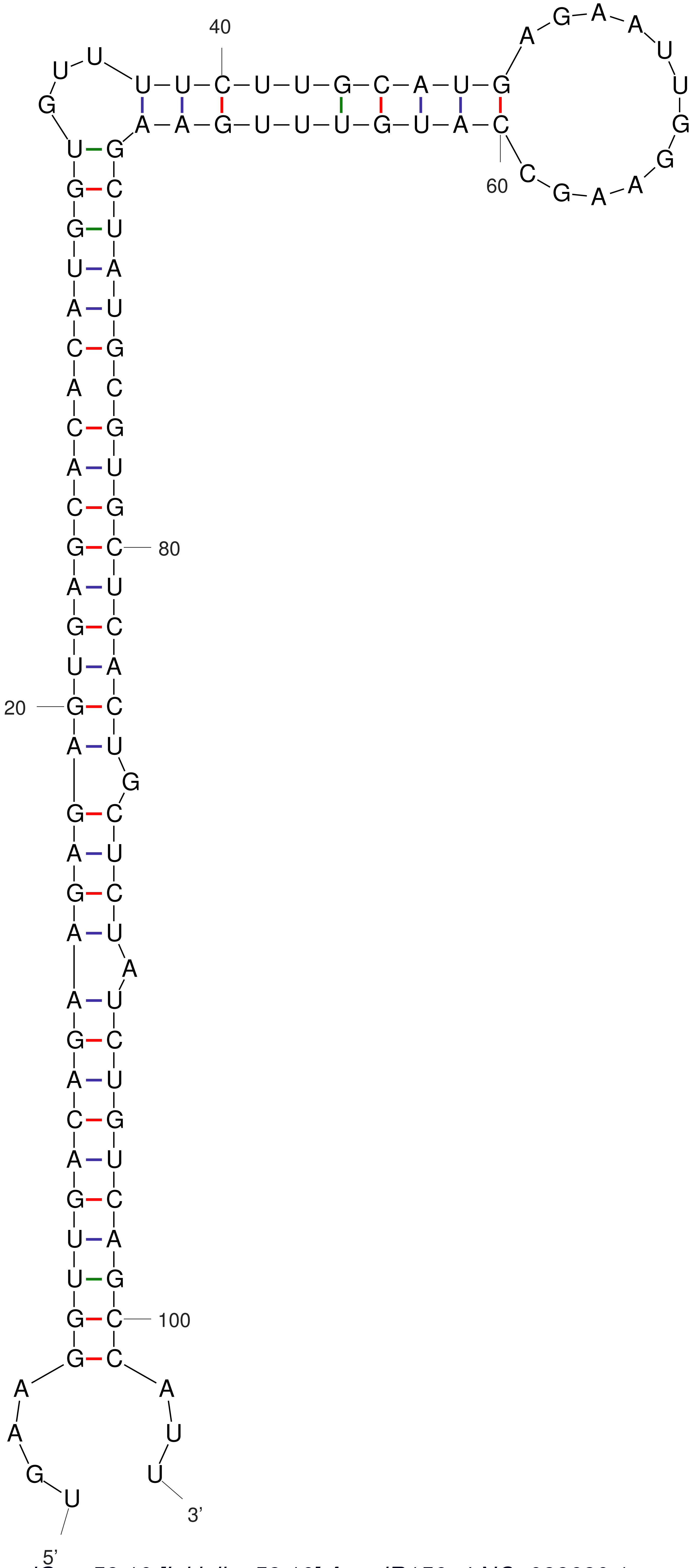

dG = -53.10 [Initially -53.10] Ac-miR156_4 NC_033630 1

Output of sir_graph (©)
mfold_util 4.7

Created Tue May 21 03:14:58 2019

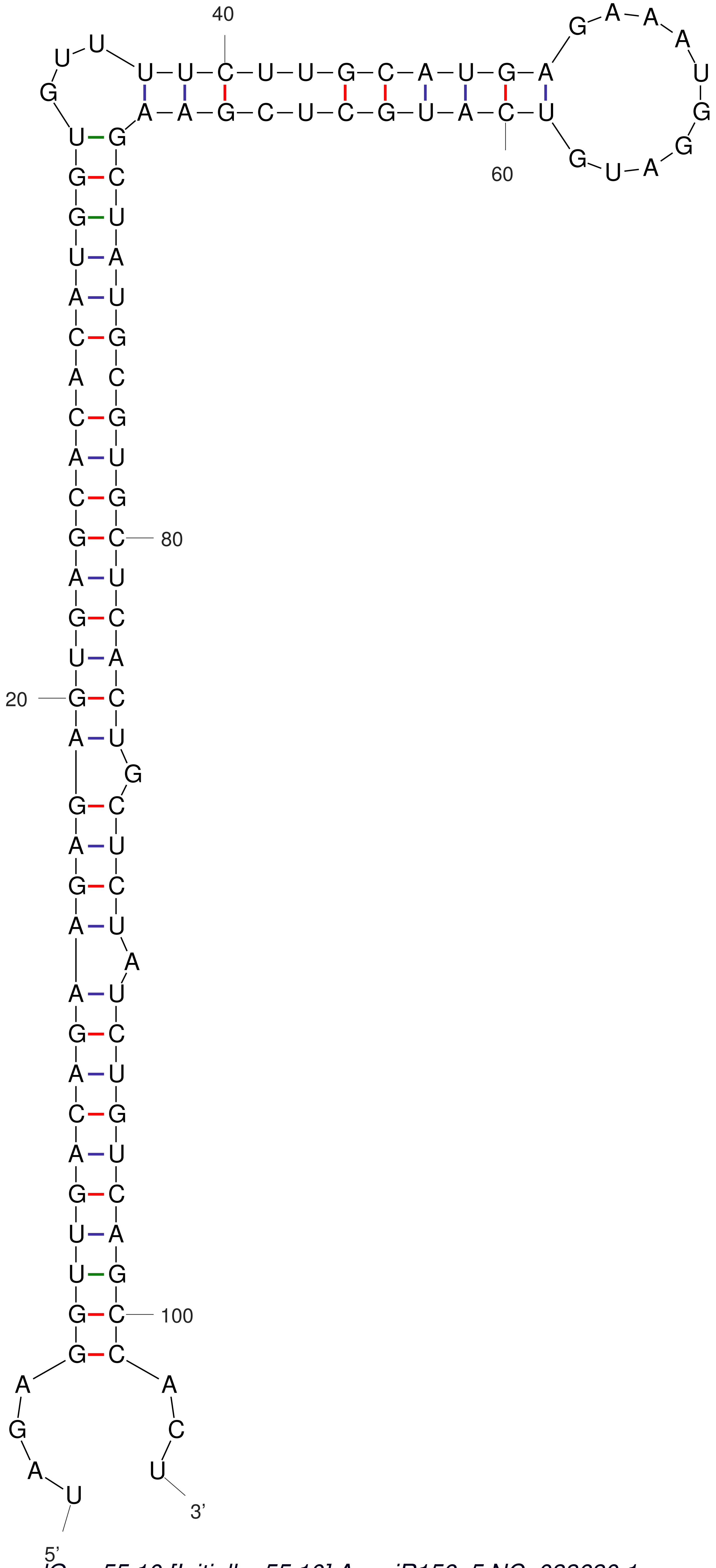

dG = -55.10 [Initially -55.10] Ac-miR156_5 NC_033630 1

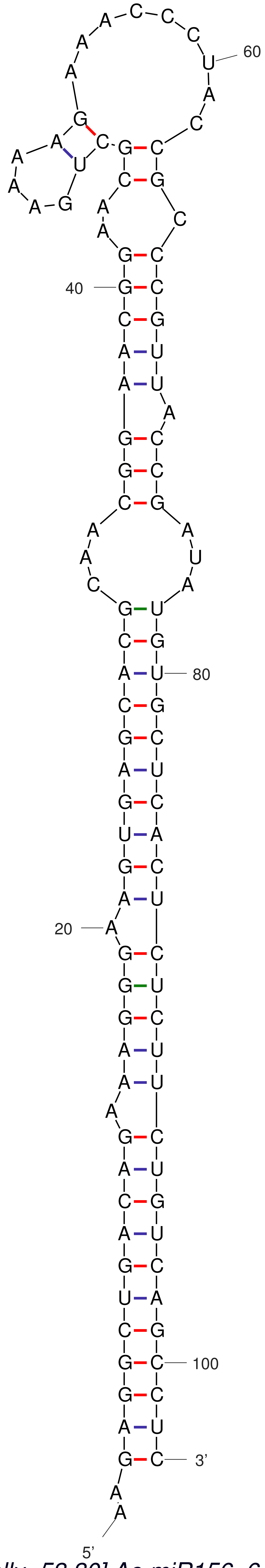

dG = -53.30 [Initially -53.30] Ac-miR156_6 NC_033634 1

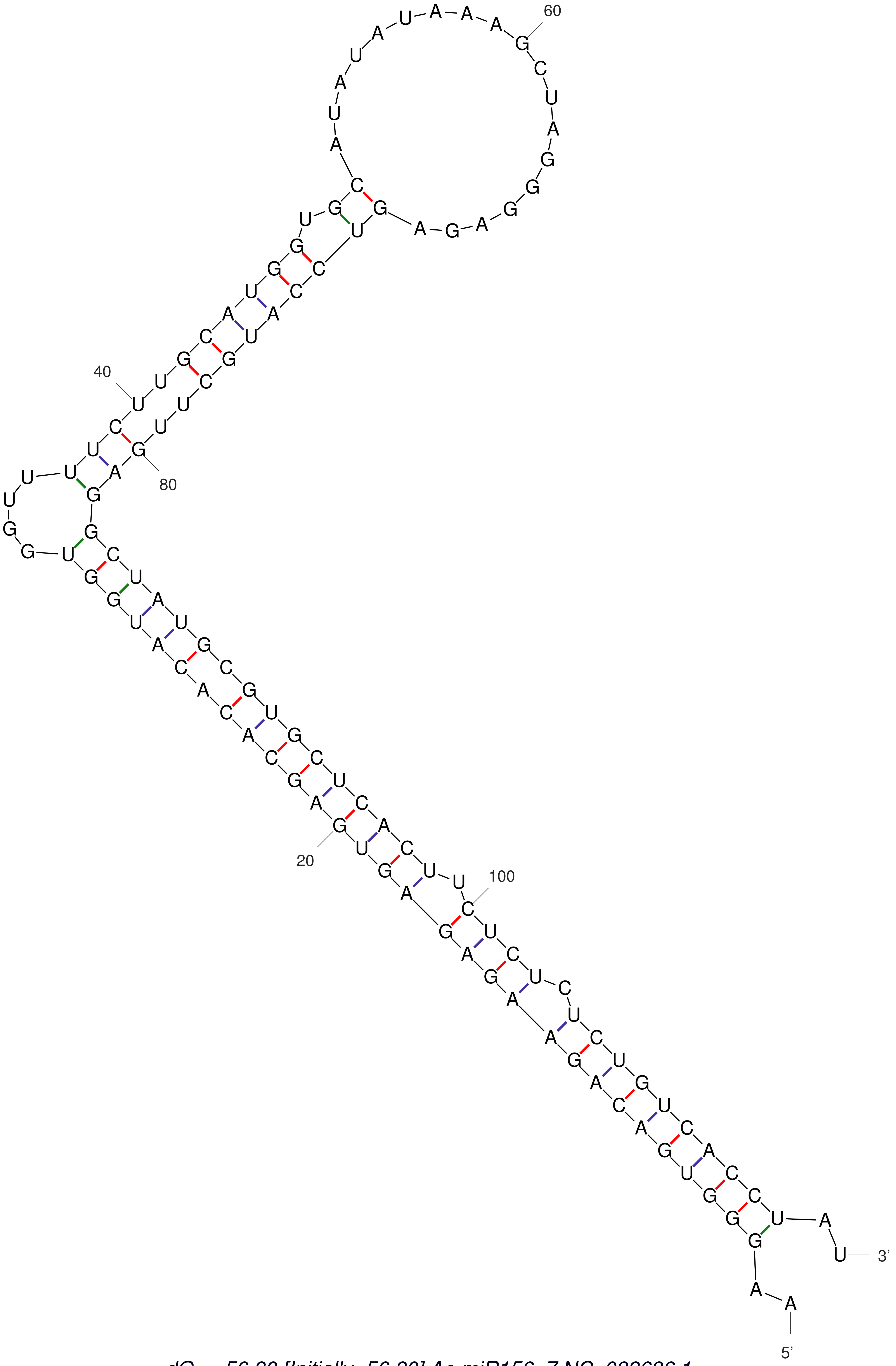

dG = -56.80 [Initially -56.80] Ac-miR156_7 NC_033636 1

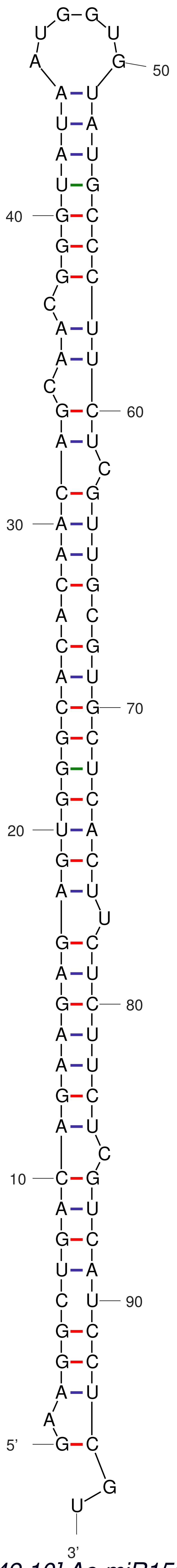

dG = -49.10 [Initially -49.10] Ac-miR156_8 NC_033639 1

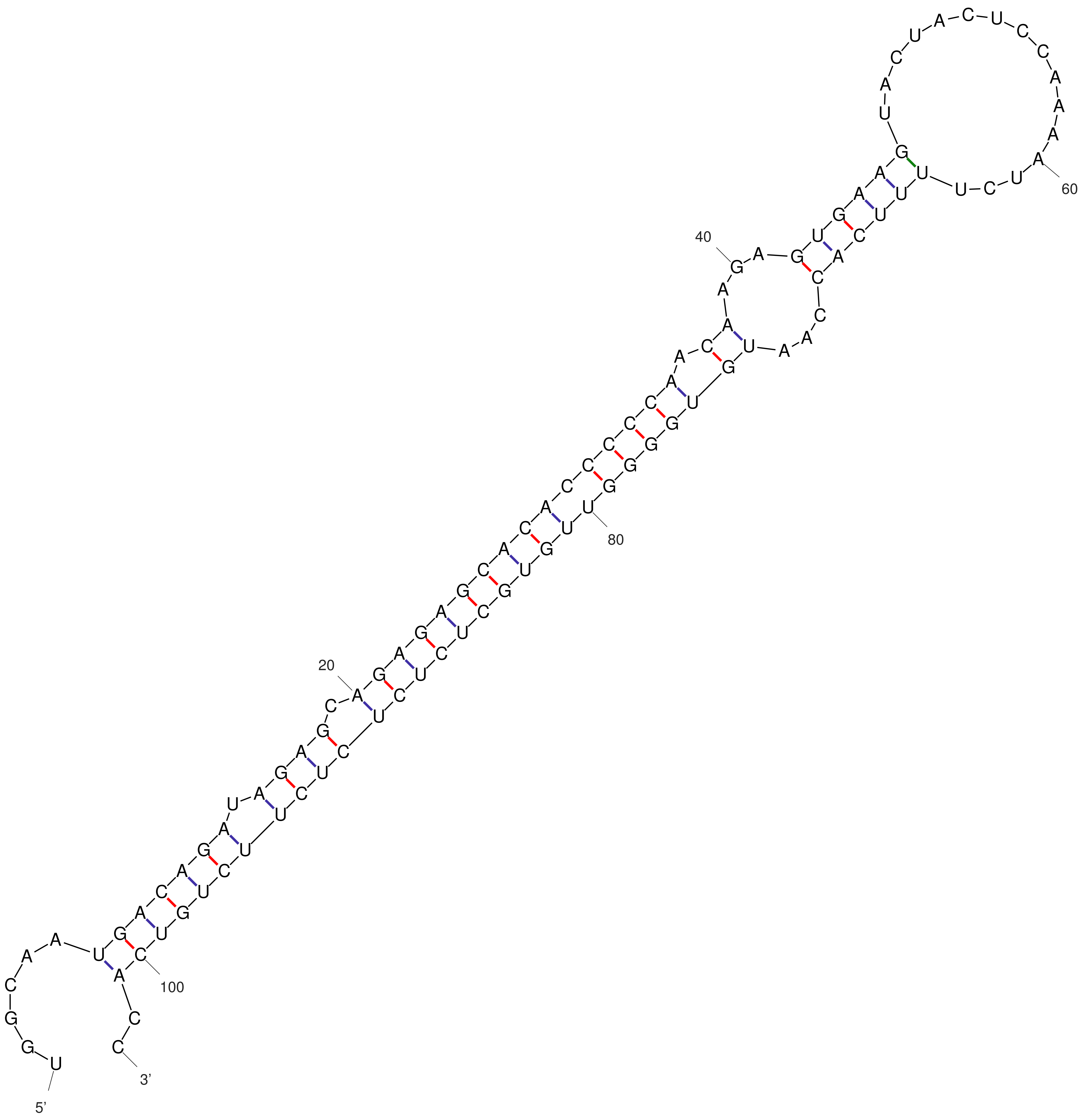

dG = -47.80 [Initially -47.80] Ac-miR156_9 NC_033640 1

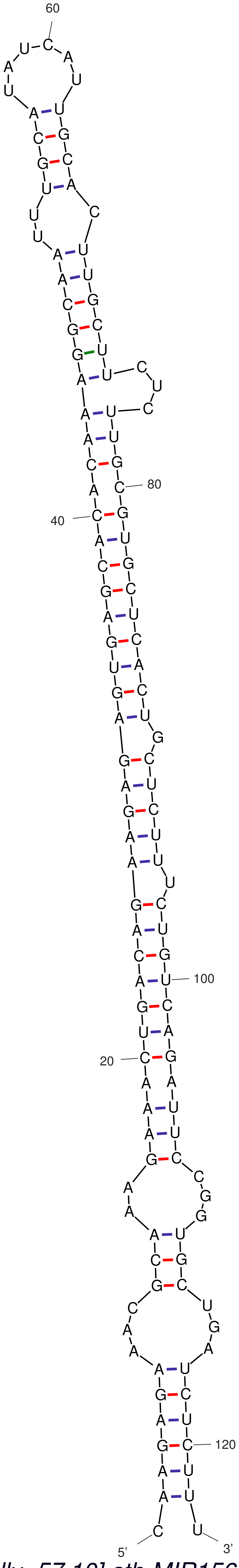

dG = -57.10 [Initially -57.10] ath-MIR156a MI0000178

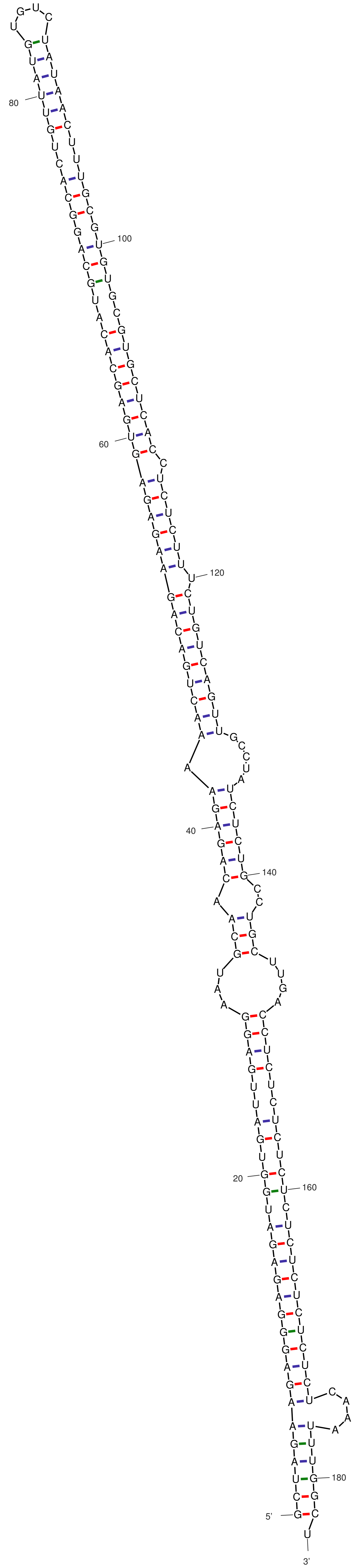

dG = -93.30 [Initially -93.30] ath-MIR156b MI0000179

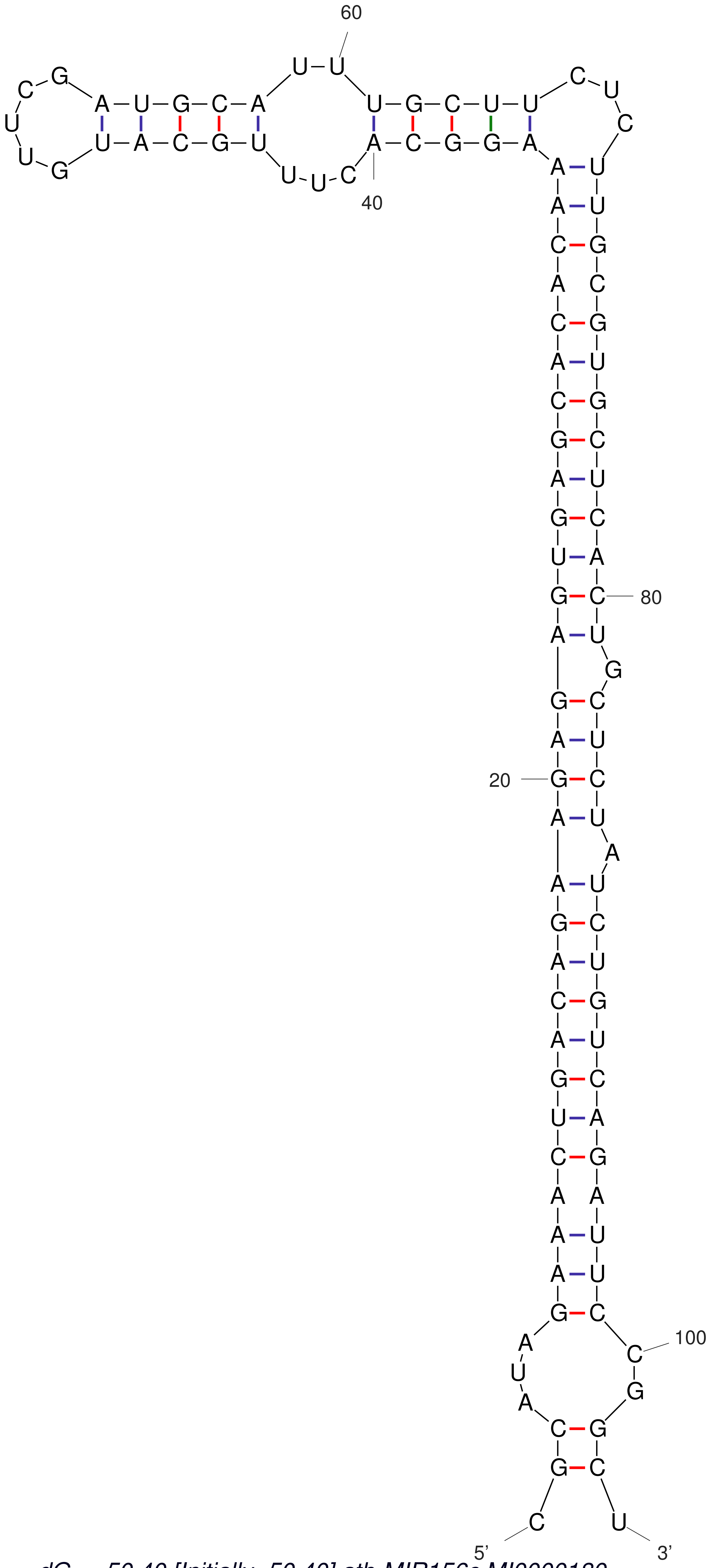

dG = -50.40 [Initially -50.40] ath-MIR156c MI0000180

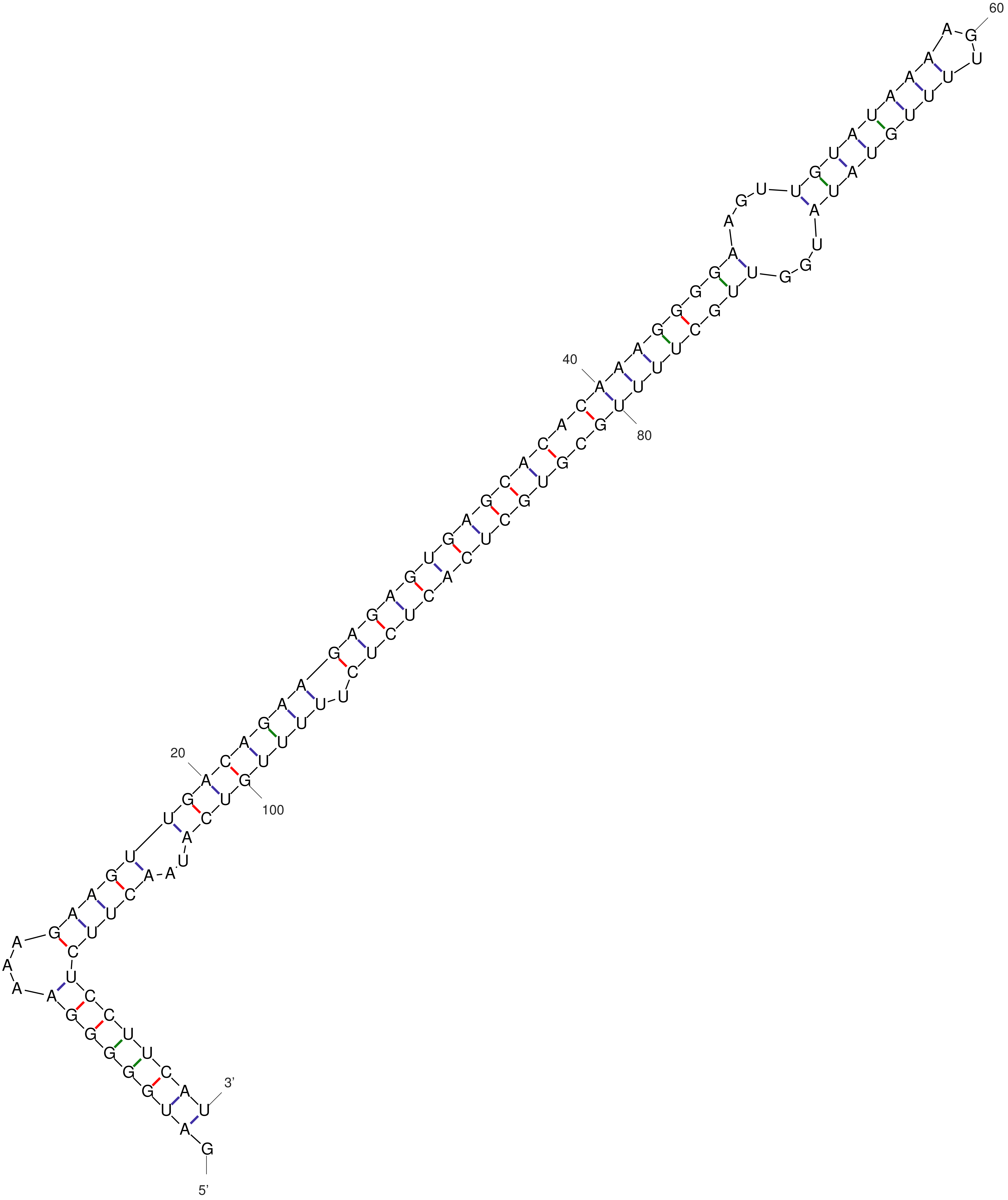

dG = -57.80 [Initially -57.80] ath-MIR156d MI0000181

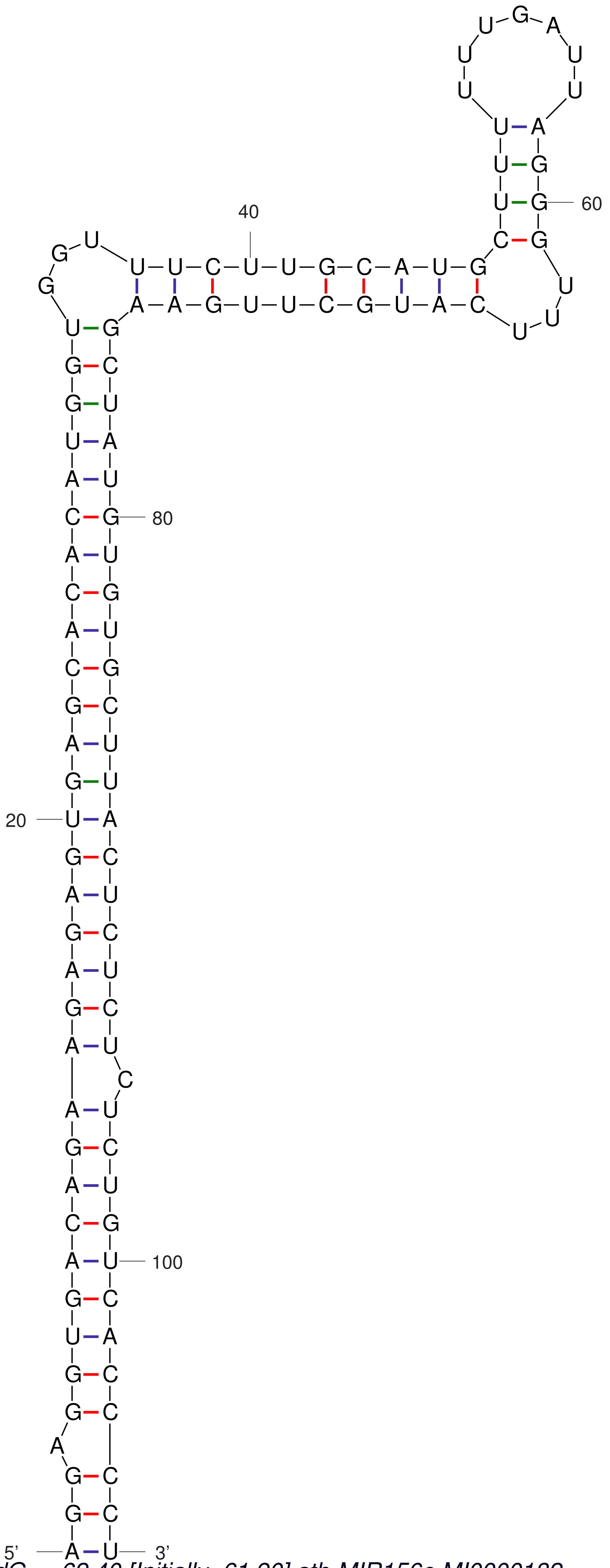

dG = -62.40 [Initially -61.90] ath-MIR156e MI0000182

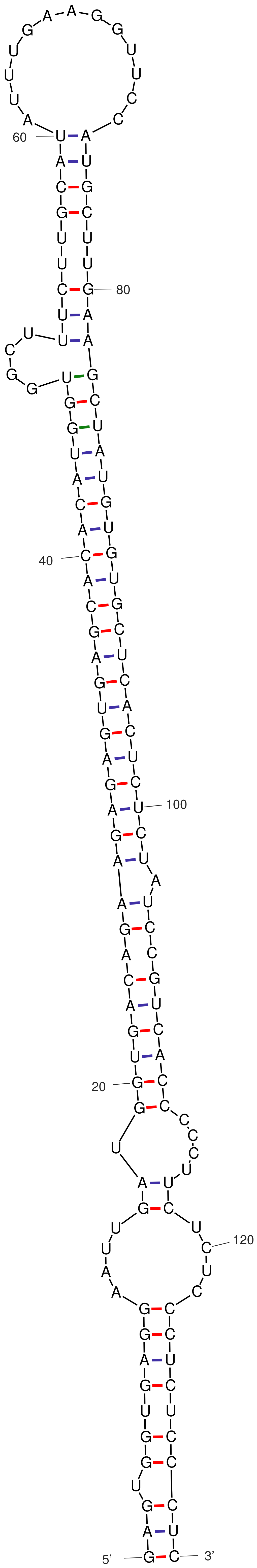

dG = -63.40 [Initially -63.40] ath-MIR156f MI0000183

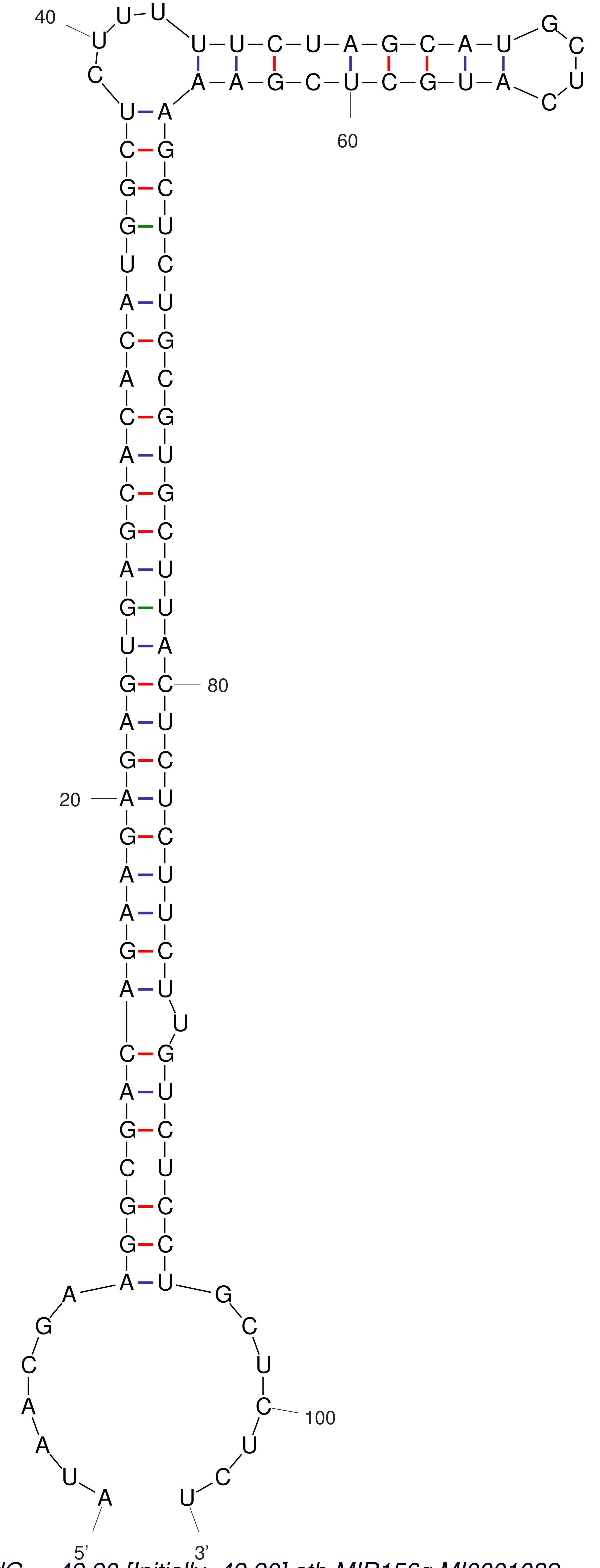

dG = -49.90 [Initially -49.90] ath-MIR156g MI0001082

Output of sir_graph (©)
mfold_util 4.7

Created Tue May 21 01:20:55 2019

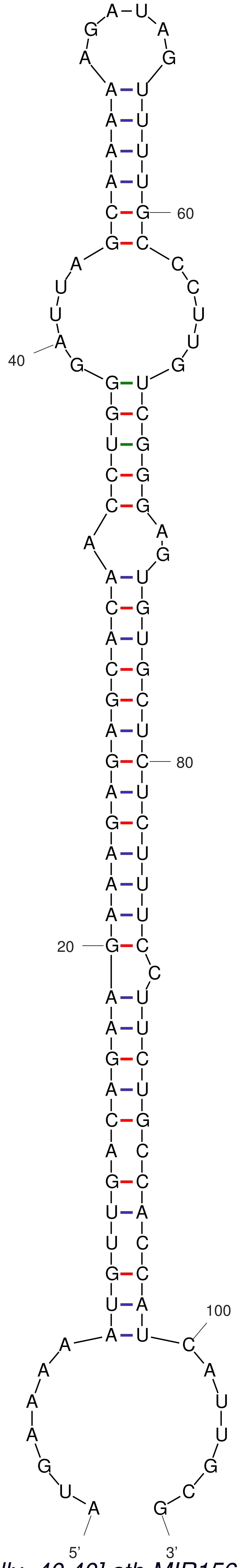

dG = -40.40 [Initially -40.40] ath-MIR156h MI0001083

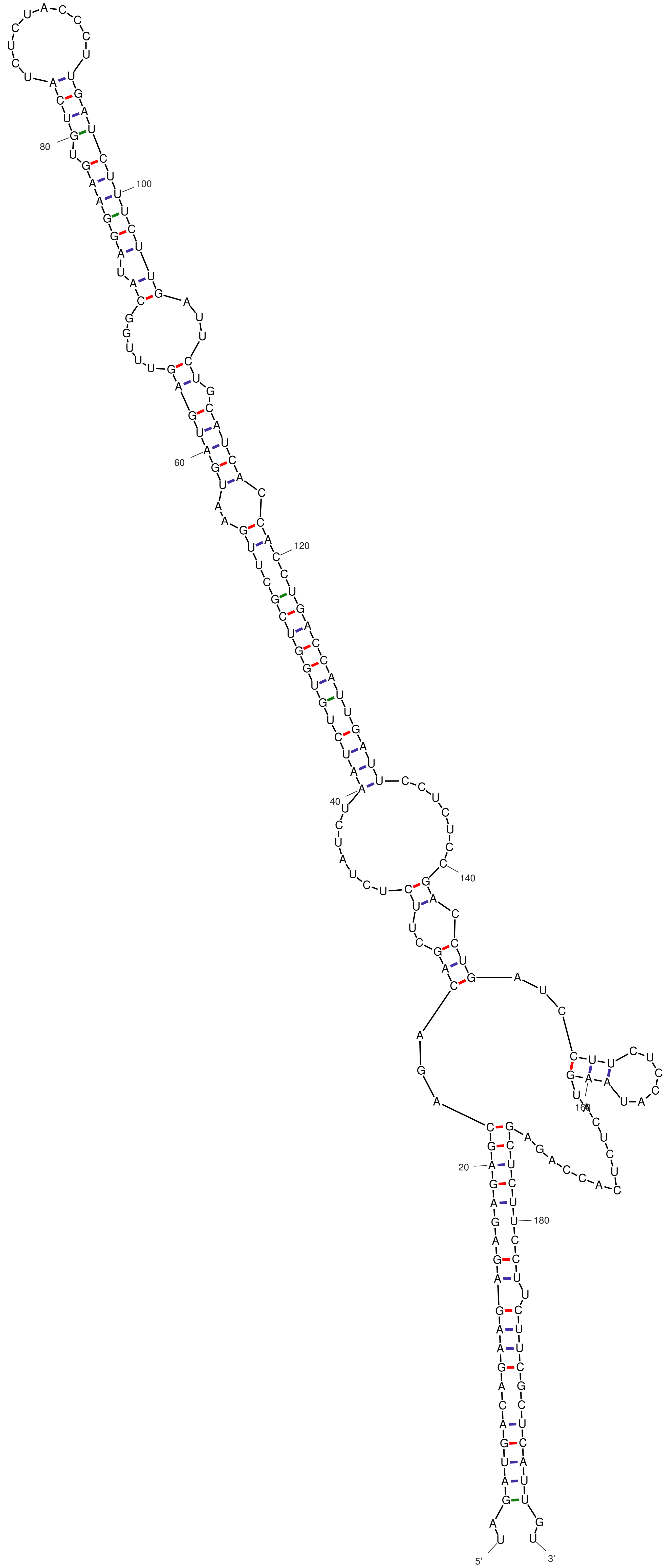

dG = -40.63 [Initially -45.30] ath-MIR156i MI0019232

Output of sir_graph (©)
mfold_util 4.7

Created Tue May 21 01:21:34 2019

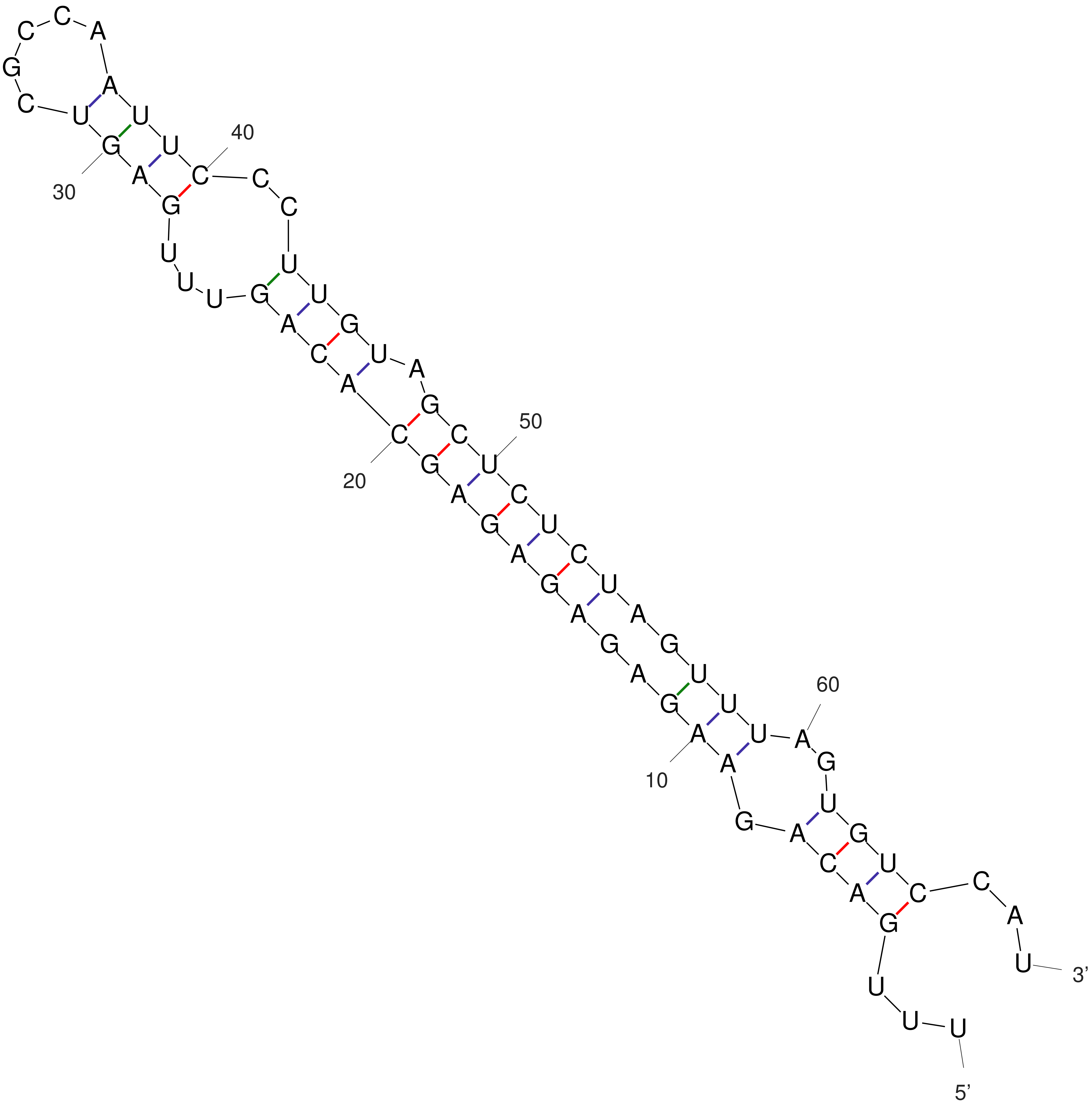

dG = -19.90 [Initially -19.90] ath-MIR156j MI0019234

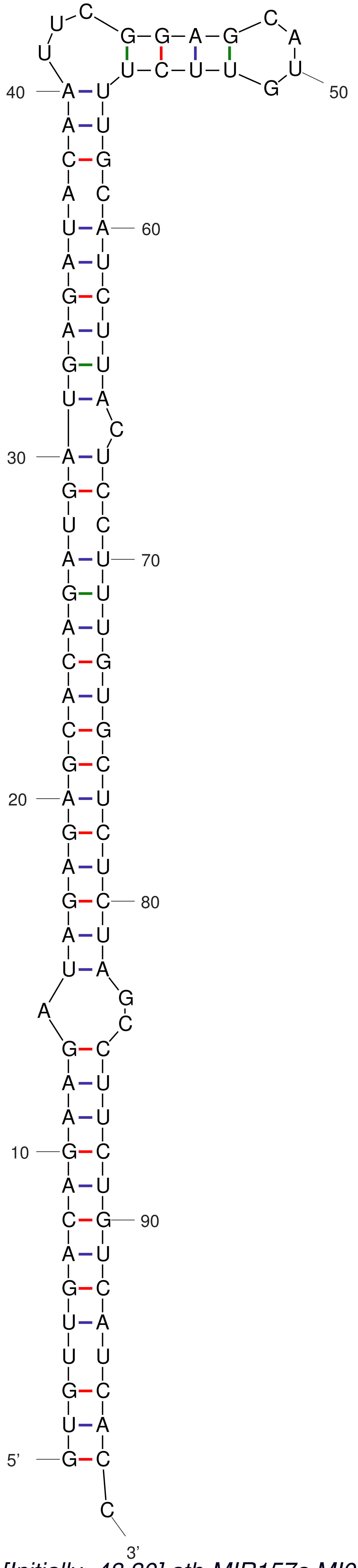

dG = -48.30 [Initially -48.30] ath-MIR157a MI0000184

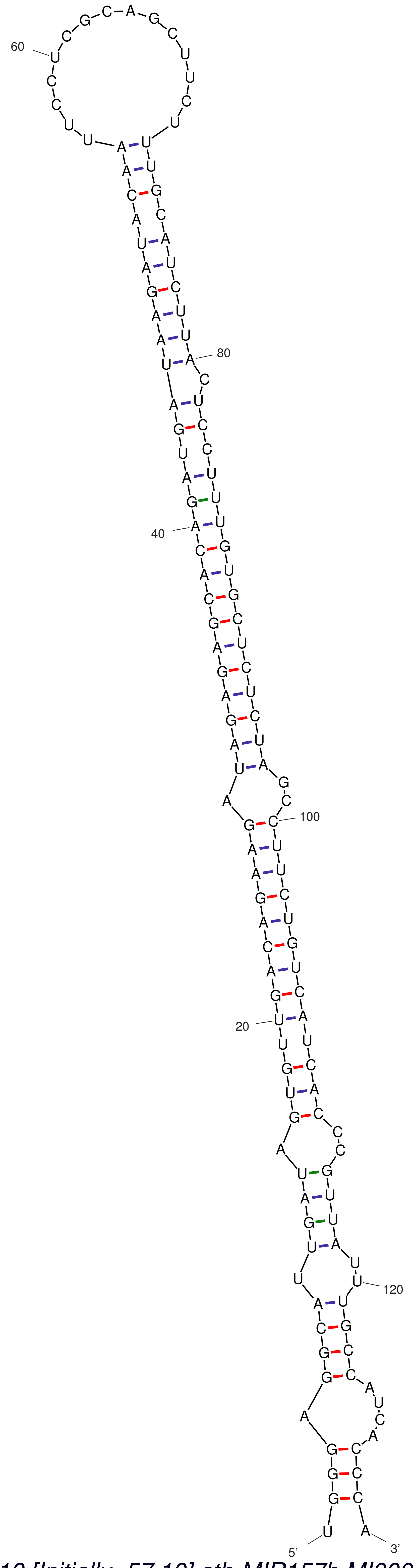

dG = -57.10 [Initially -57.10] ath-MIR157b MI0000185

Output of sir_graph (©)
mfold_util 4.7

Created Tue May 21 01:24:09 2019

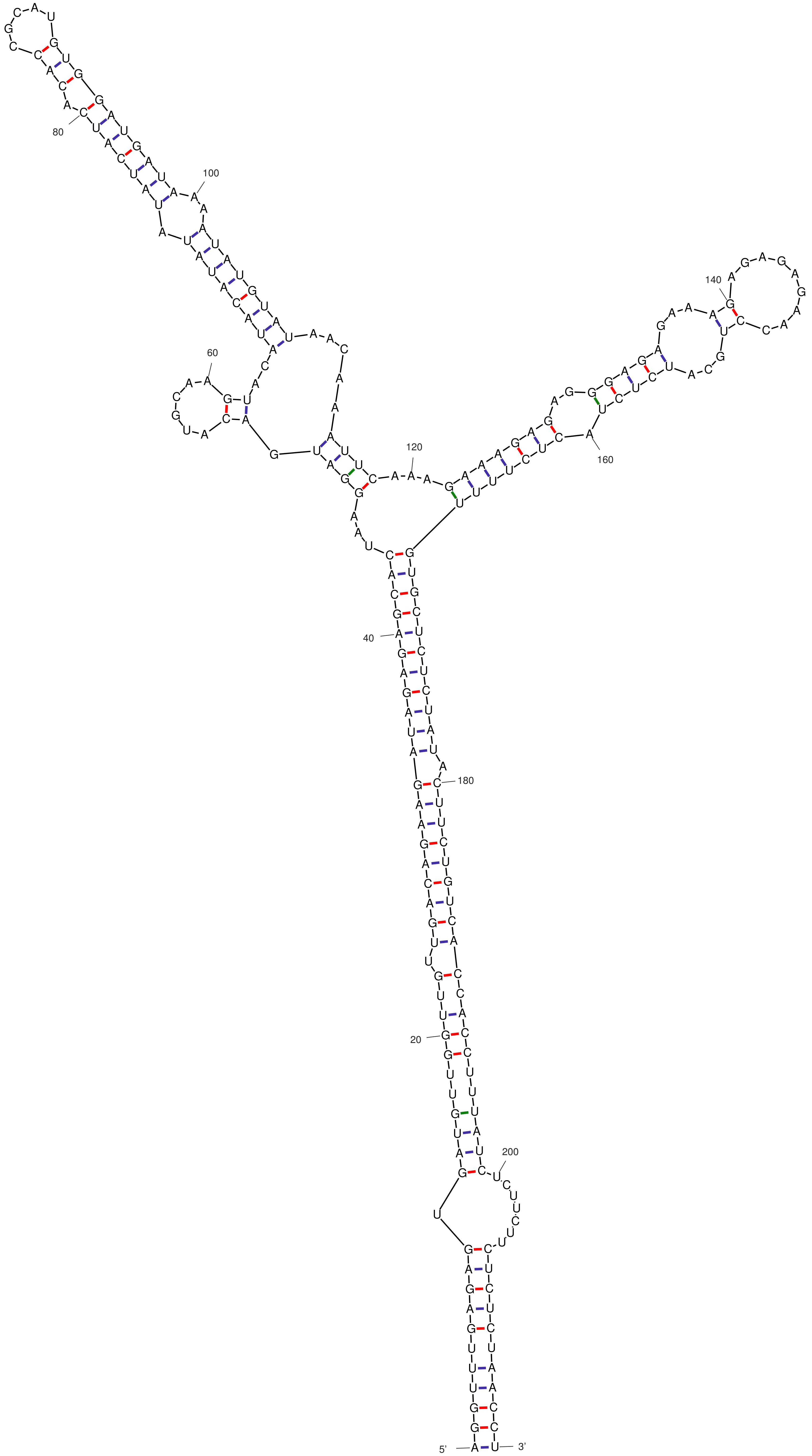

dG = -75.98 [Initially -79.00] ath-MIR157c MI0000186

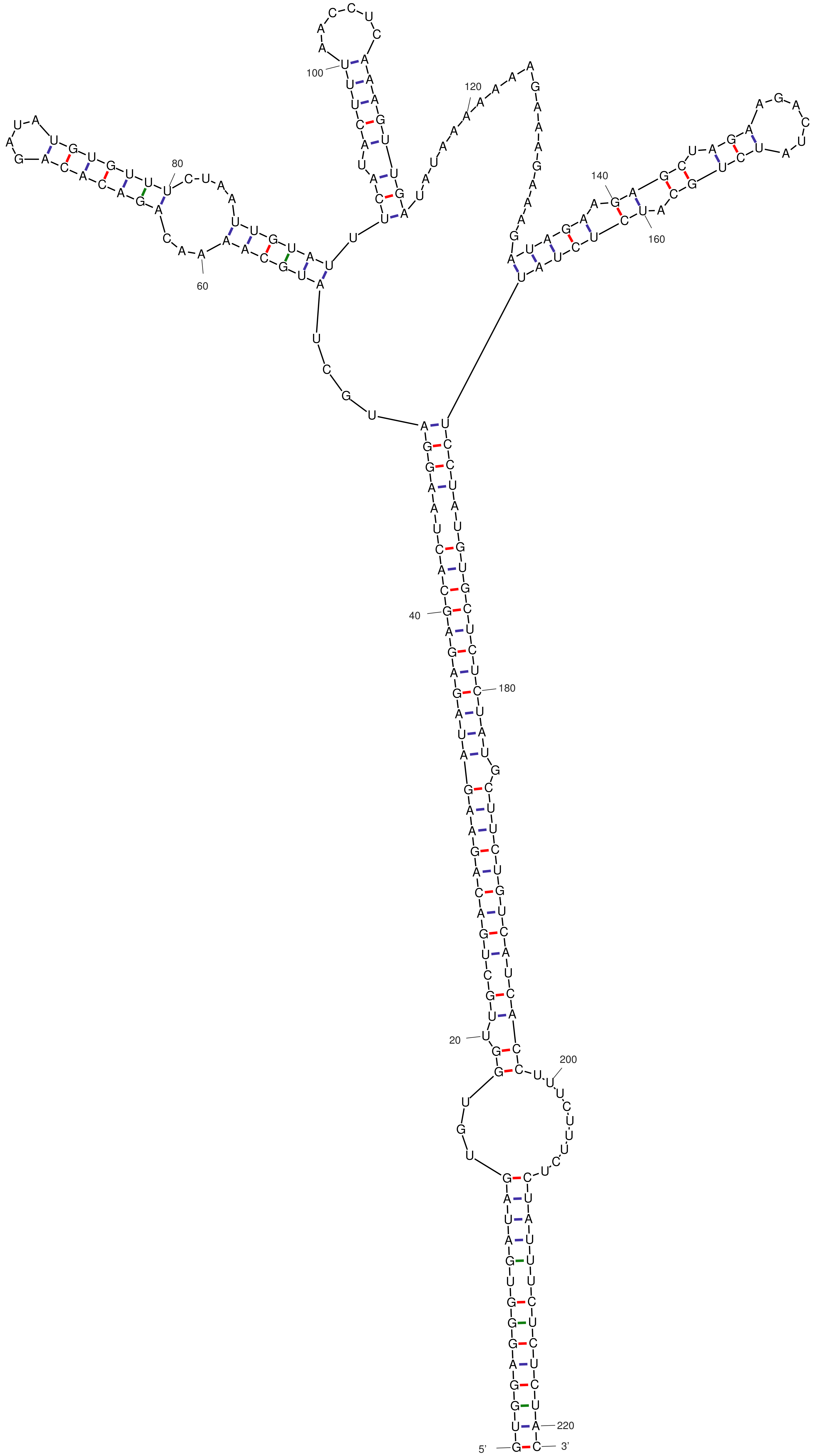

dG = -69.48 [Initially -72.30] ath-MIR157d MI0000187

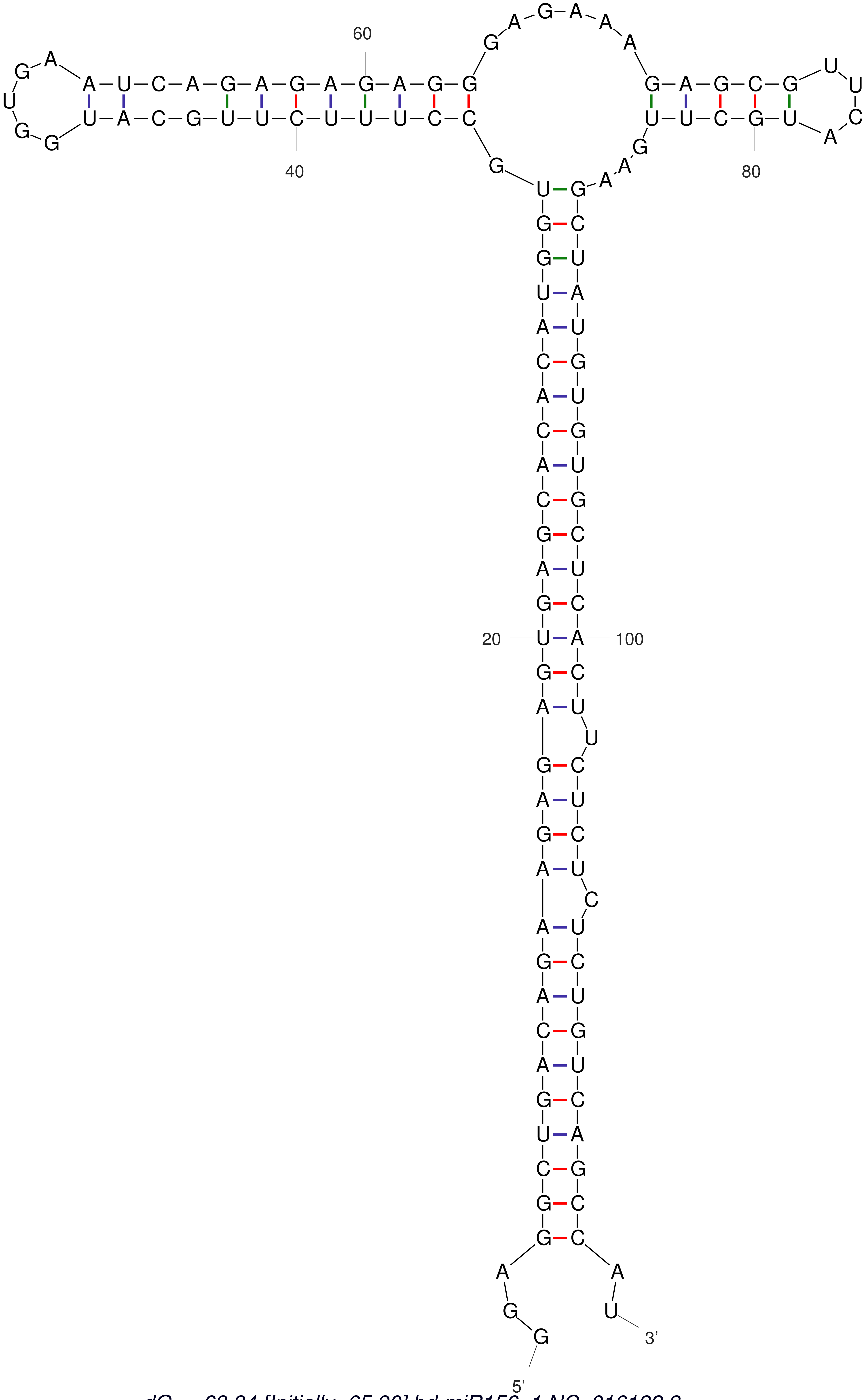

dG = -63.34 [Initially -65.90] bd-miR156_1 NC_016132 3

Output of sir_graph (©)
mfold_util 4.7

Created Mon Jul 22 08:46:58 2019

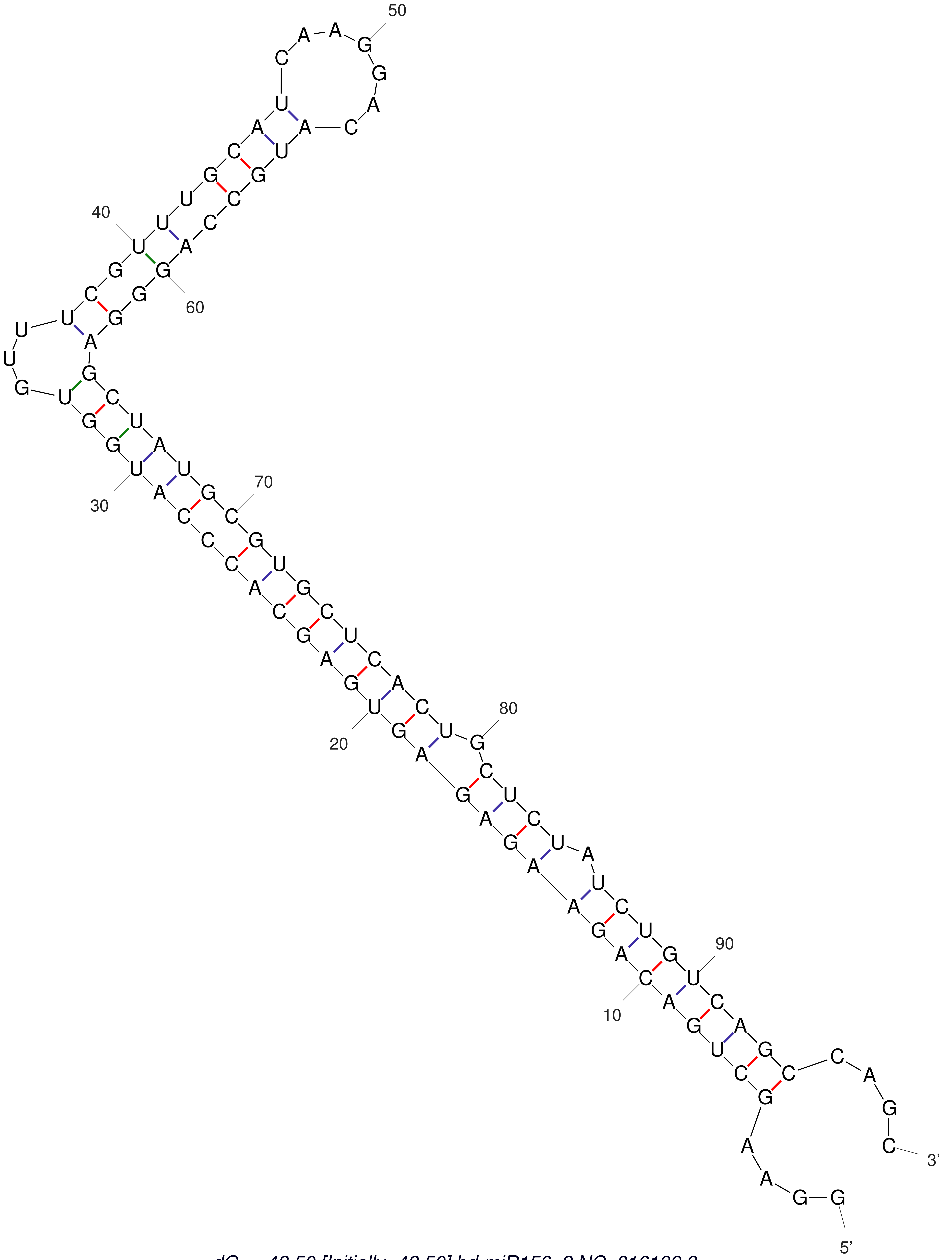

dG = -48.50 [Initially -48.50] bd-miR156_2 NC_016132 3

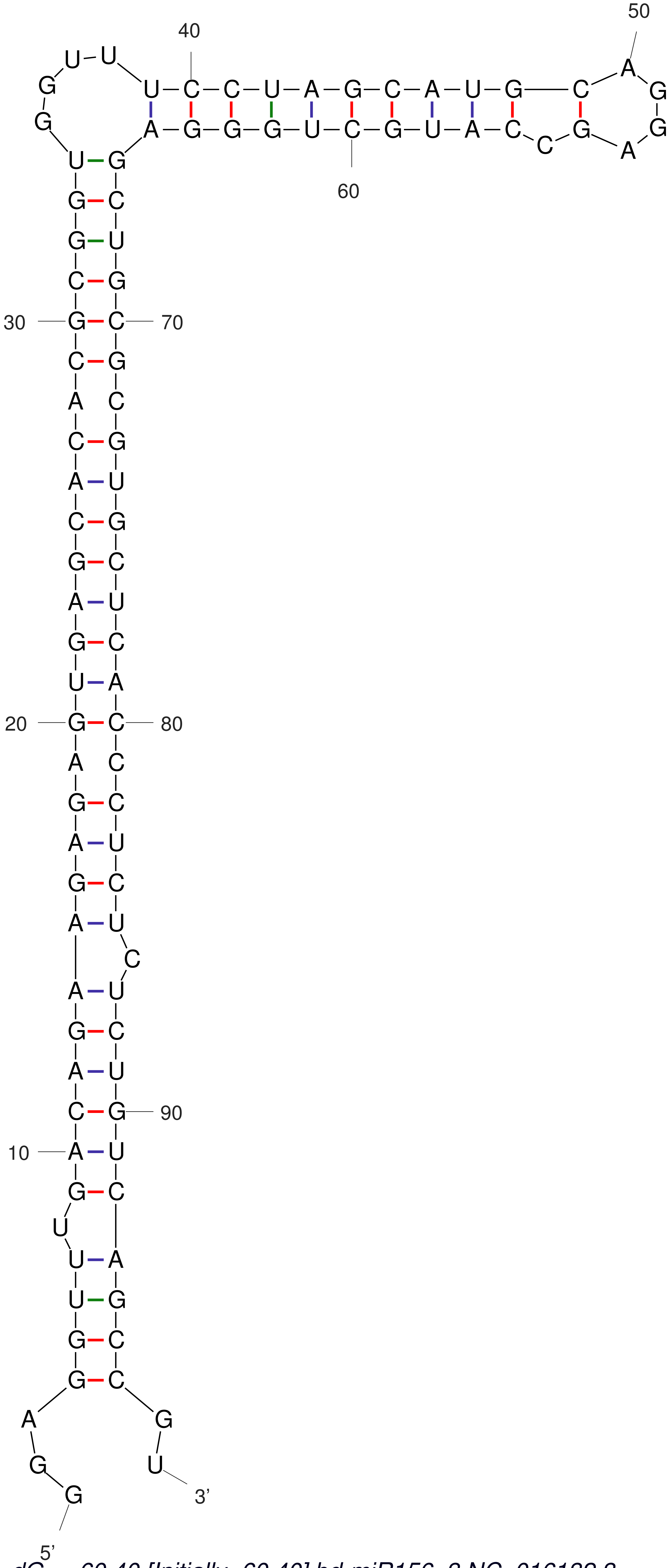

dG = -60.40 [Initially -60.40] bd-miR156_3 NC_016132 3

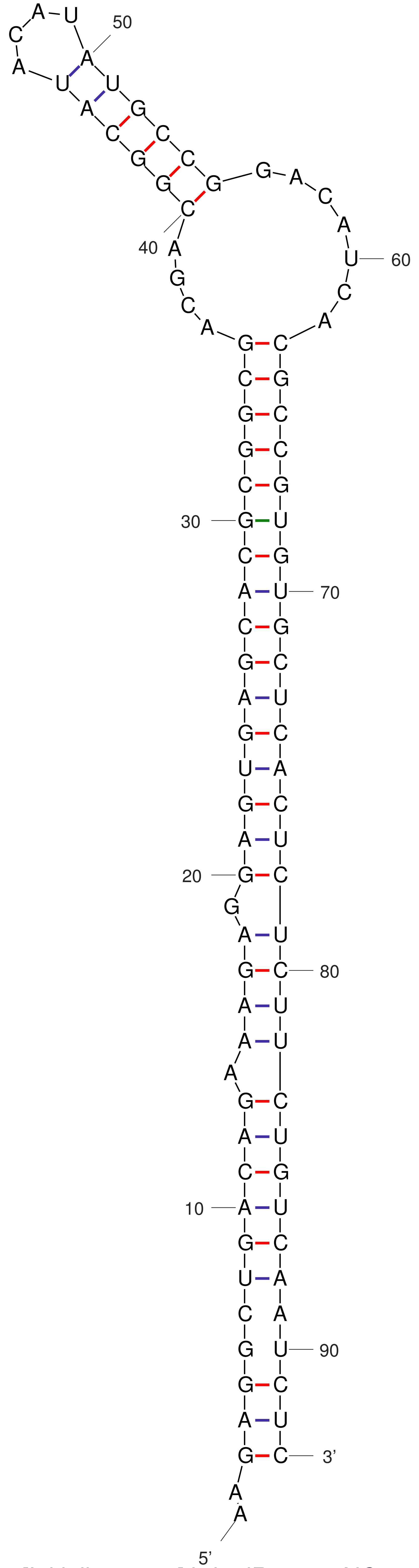

dG = -59.30 [Initially -59.30] bd-miR156_4 NC_016133 3

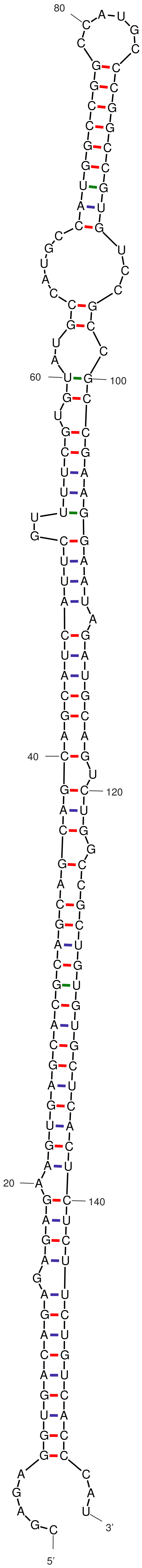

dG = -83.60 [Initially -83.80] bd-miR156_5 NC_016133 3

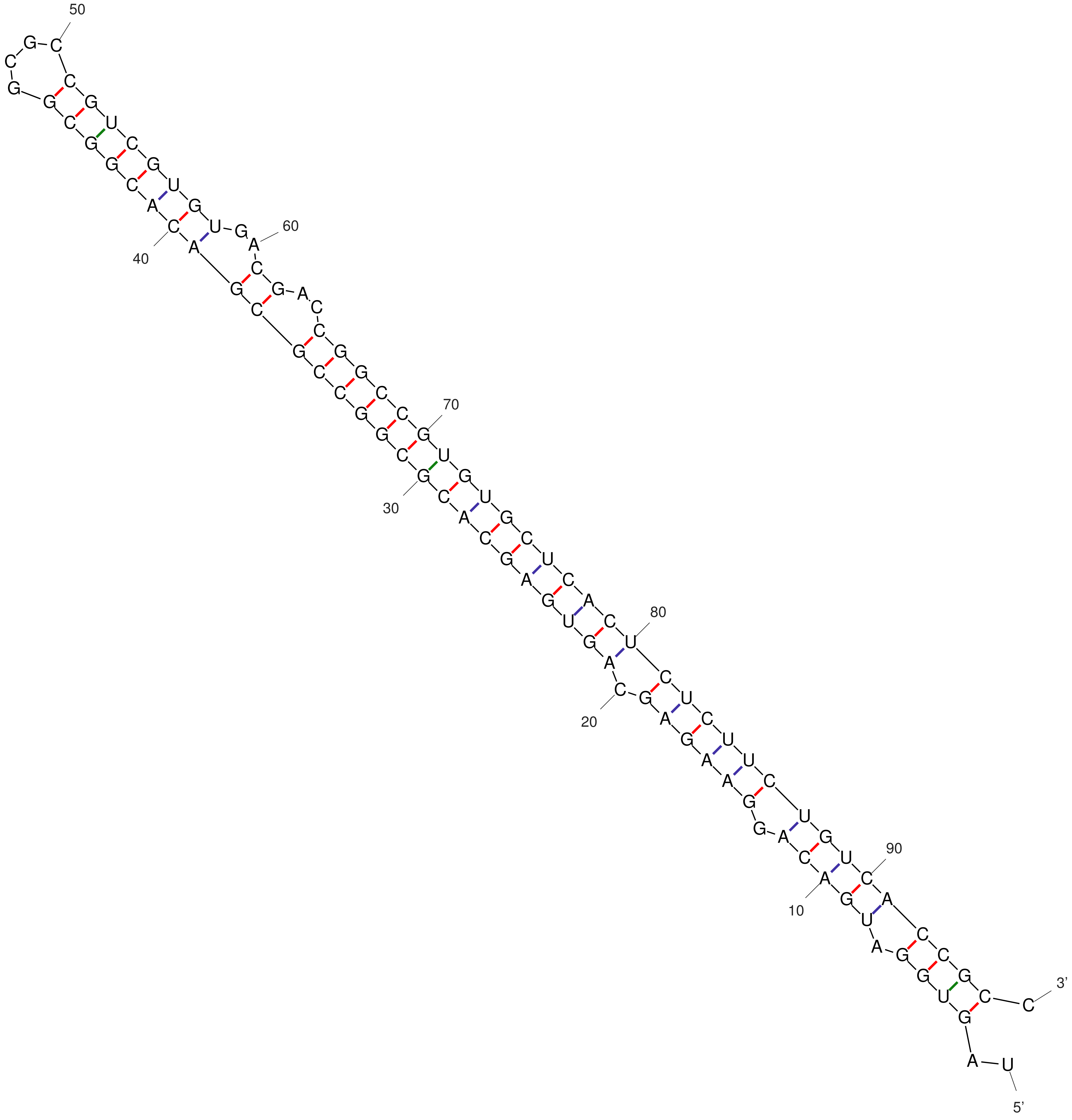

dG = -68.40 [Initially -68.40] bd-miR156_6 NC_016133 3

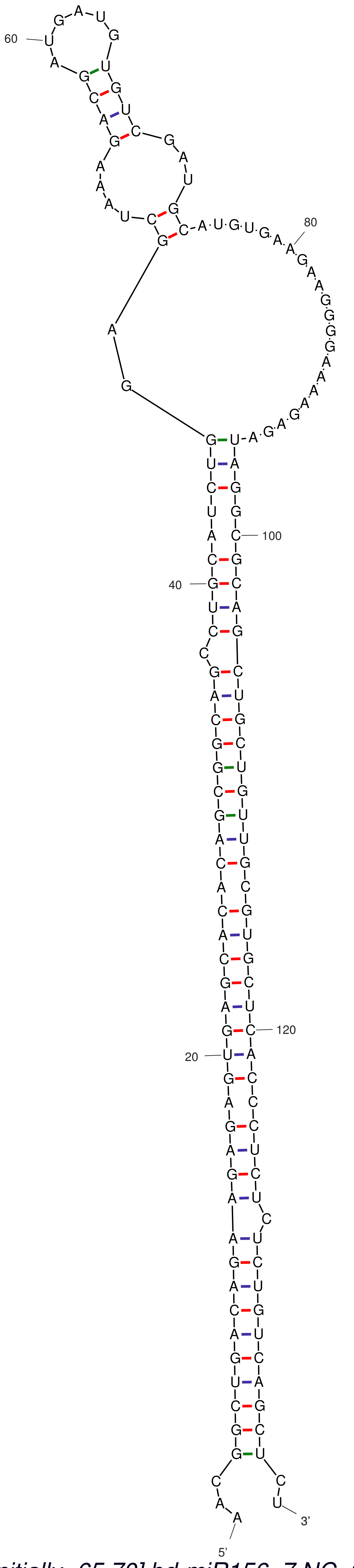

dG = -65.70 [Initially -65.70] bd-miR156_7 NC_016134 3

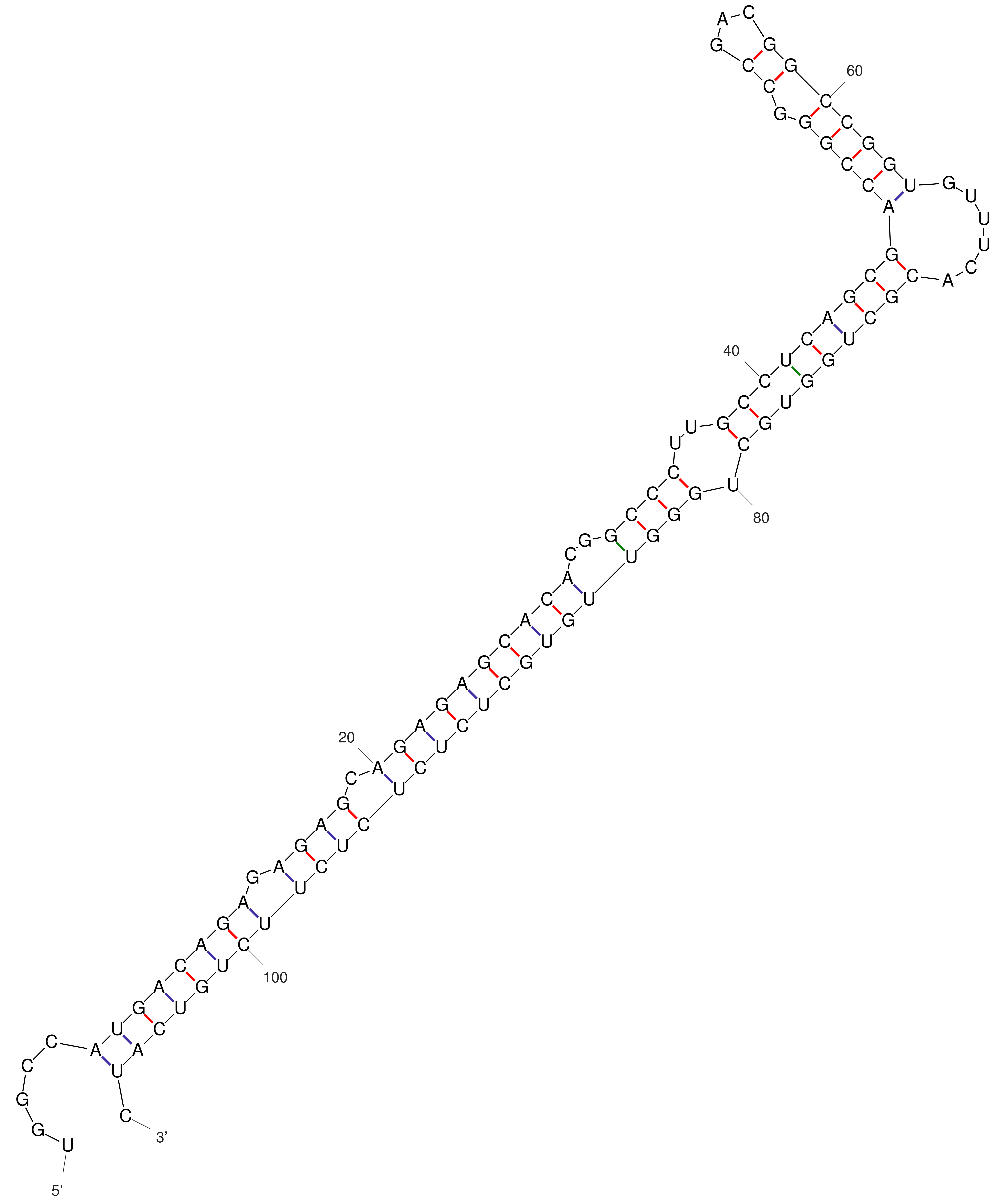

dG = -58.60 [Initially -58.90] bd-miR156_8 NC_016134 3

Output of sir_graph (©)
mfold_util 4.7

Created Mon Jul 22 08:49:17 2019

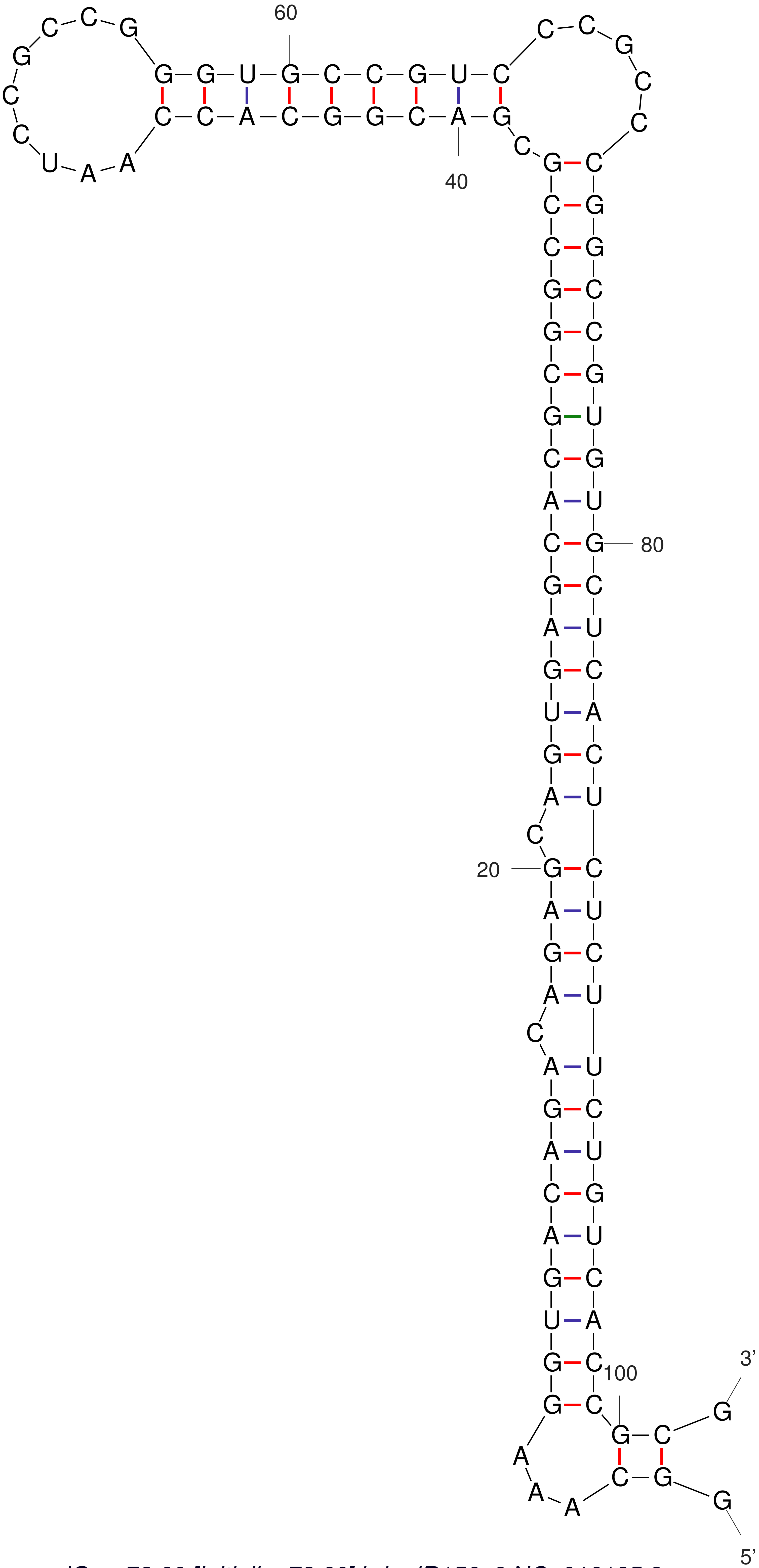

dG = -72.60 [Initially -72.60] bd-miR156_9 NC_016135 3

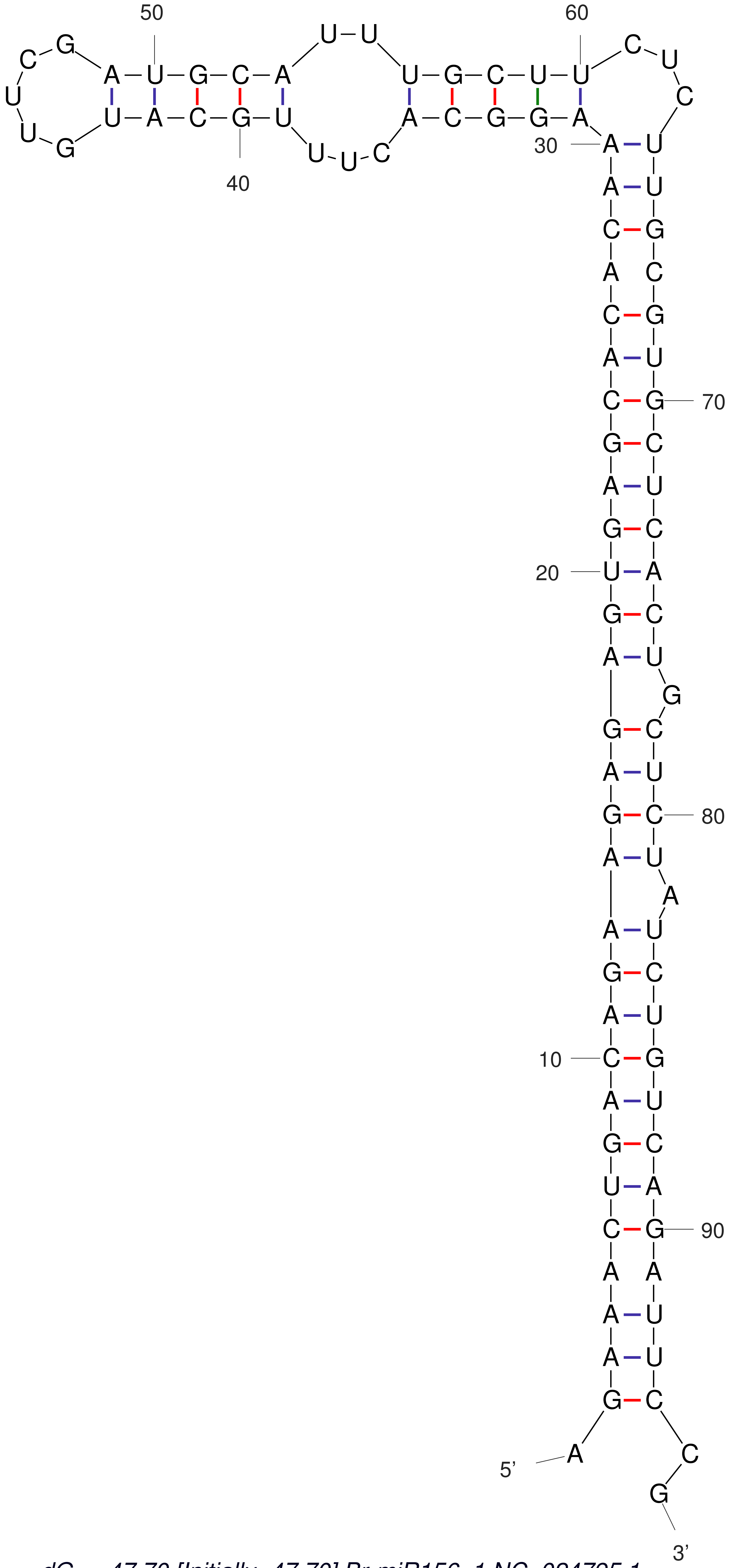

dG = -47.70 [Initially -47.70] Br-miR156_1 NC_024795 1

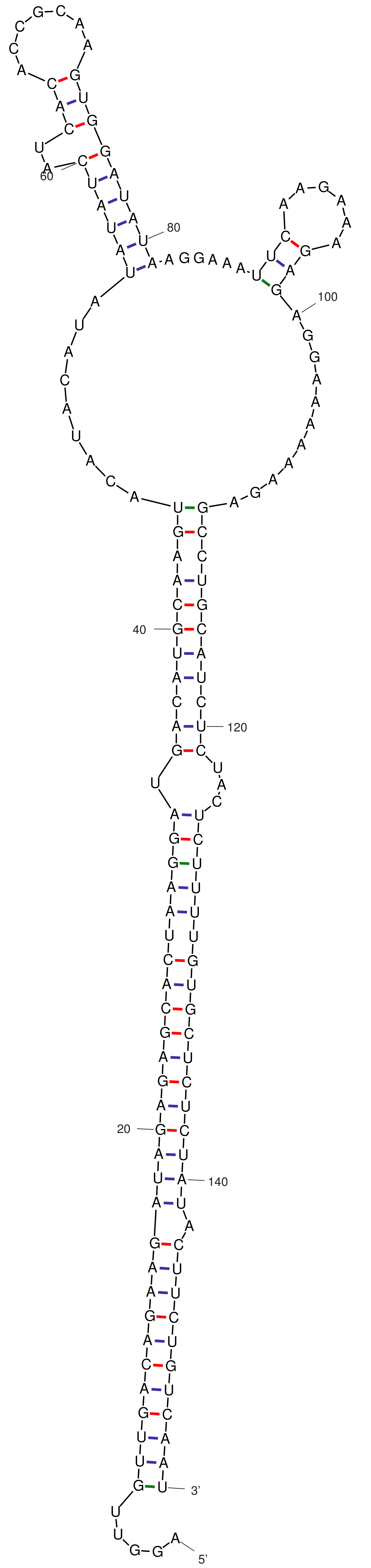

dG = -46.19 [Initially -50.90] Br-miR156_2 NC_024795 1

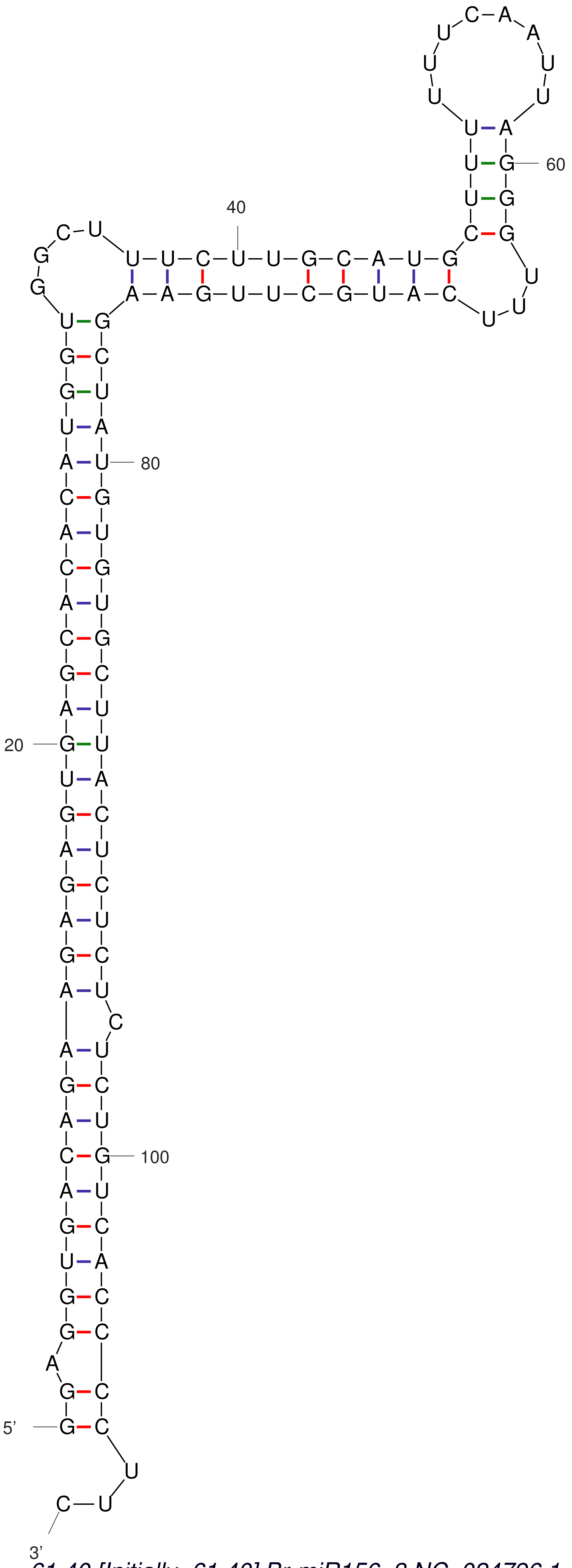

dG = -61.40 [Initially -61.40] Br-miR156_3 NC_024796 1

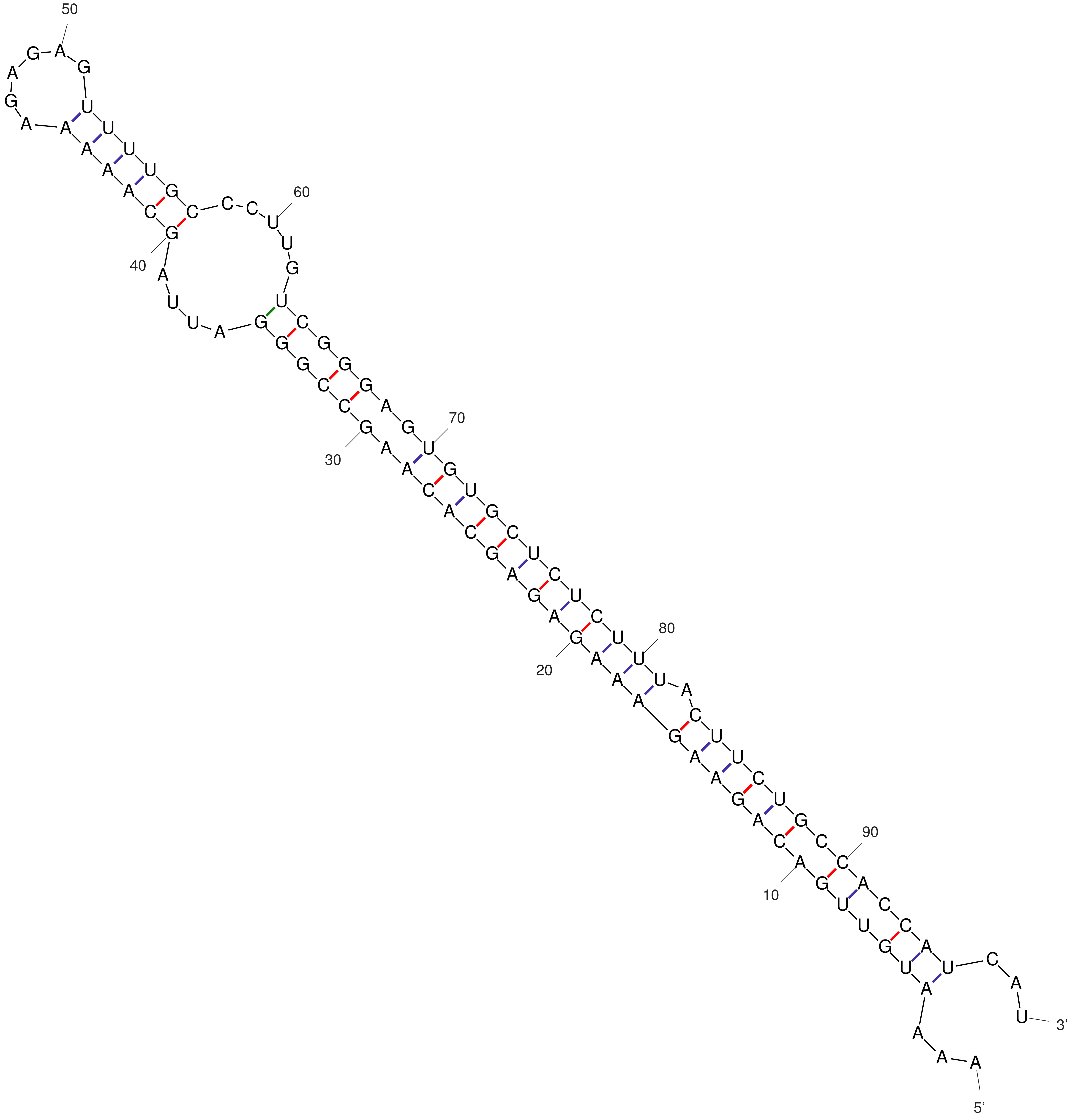

dG = -41.90 [Initially -41.90] Br-miR156_4 NC_024796 1

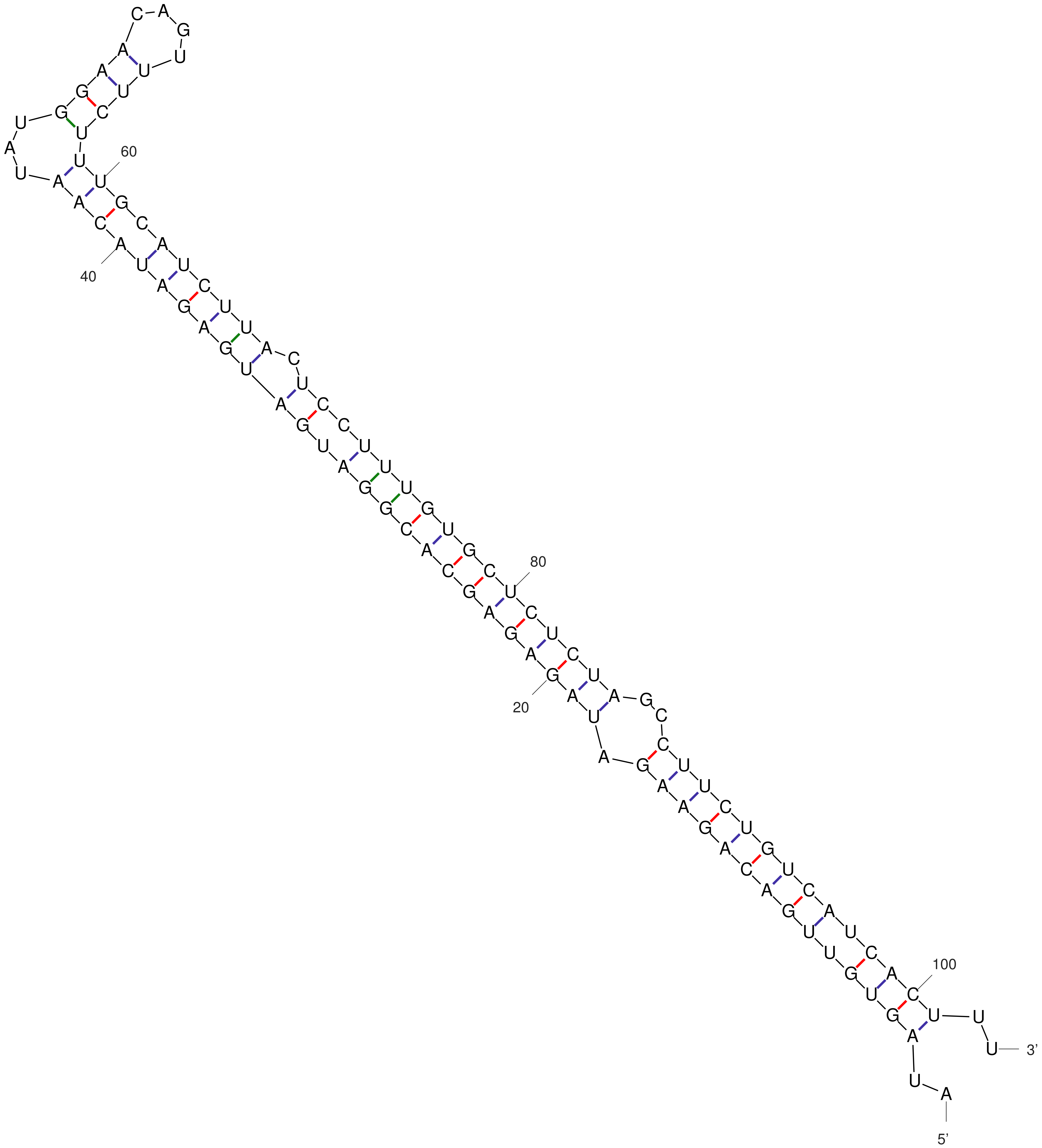

dG = -47.60 [Initially -47.60] Br-miR156_5 NC_024796 1

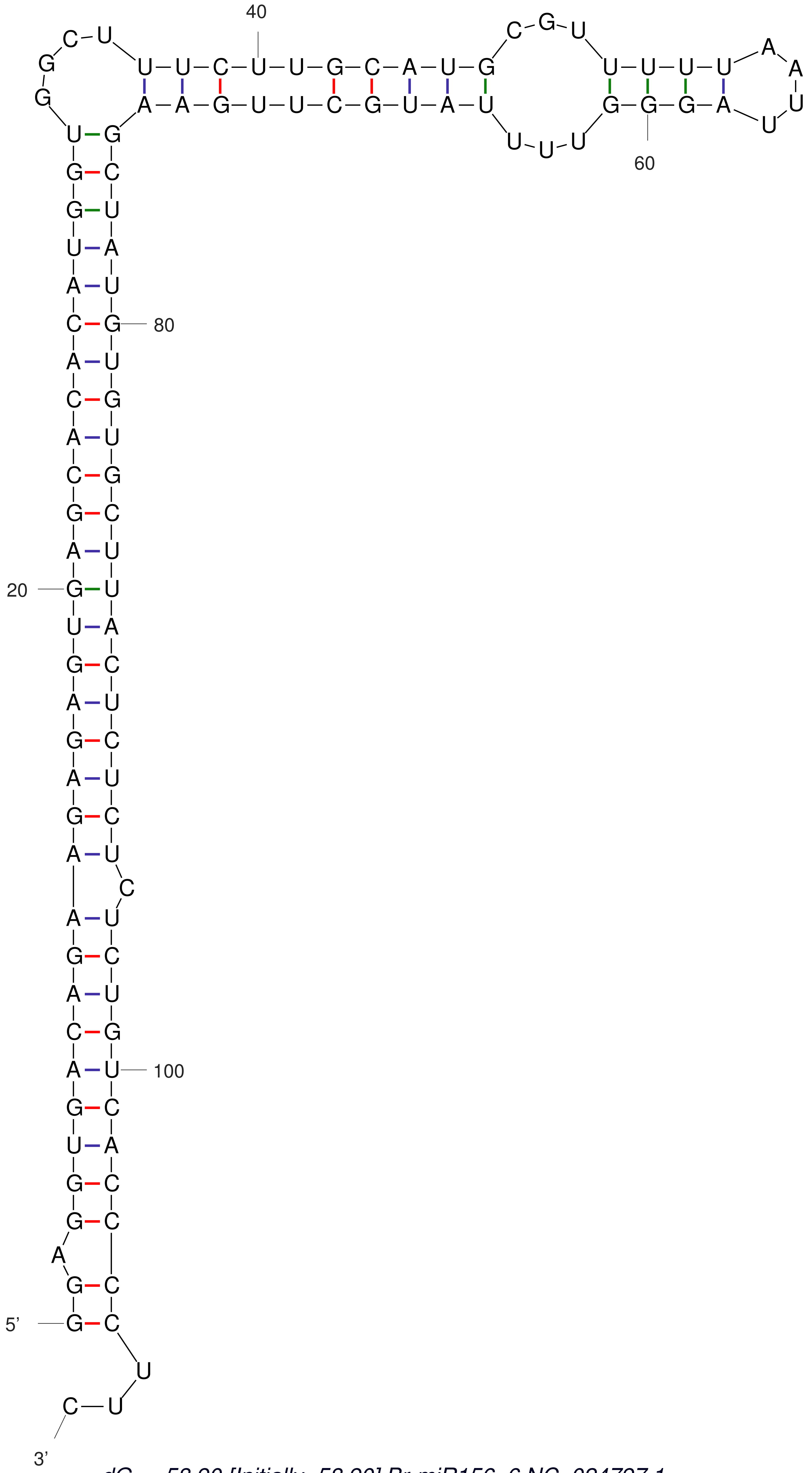

dG = -58.90 [Initially -58.90] Br-miR156_6 NC_024797 1

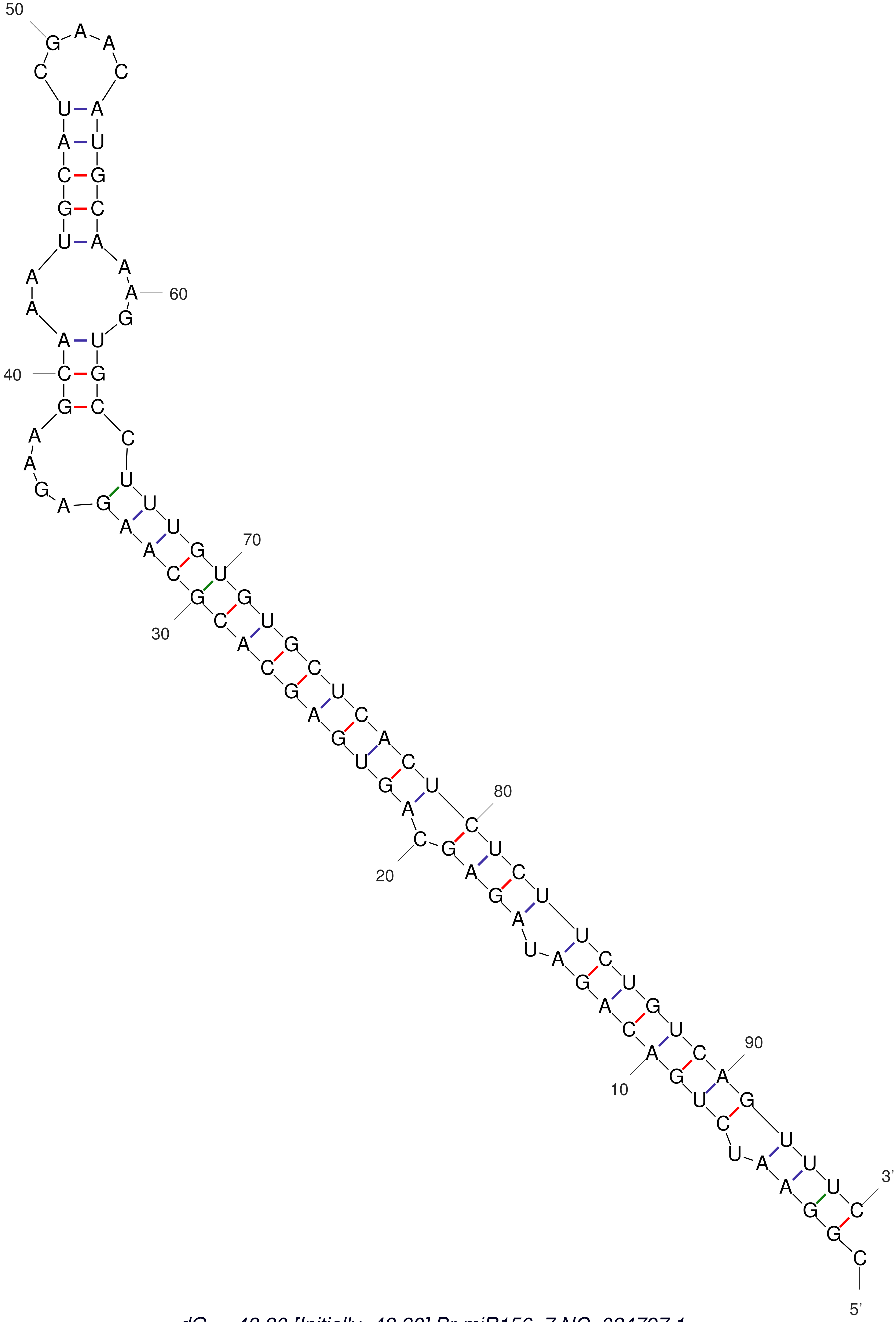

dG = -48.30 [Initially -48.30] Br-miR156_7 NC_024797 1

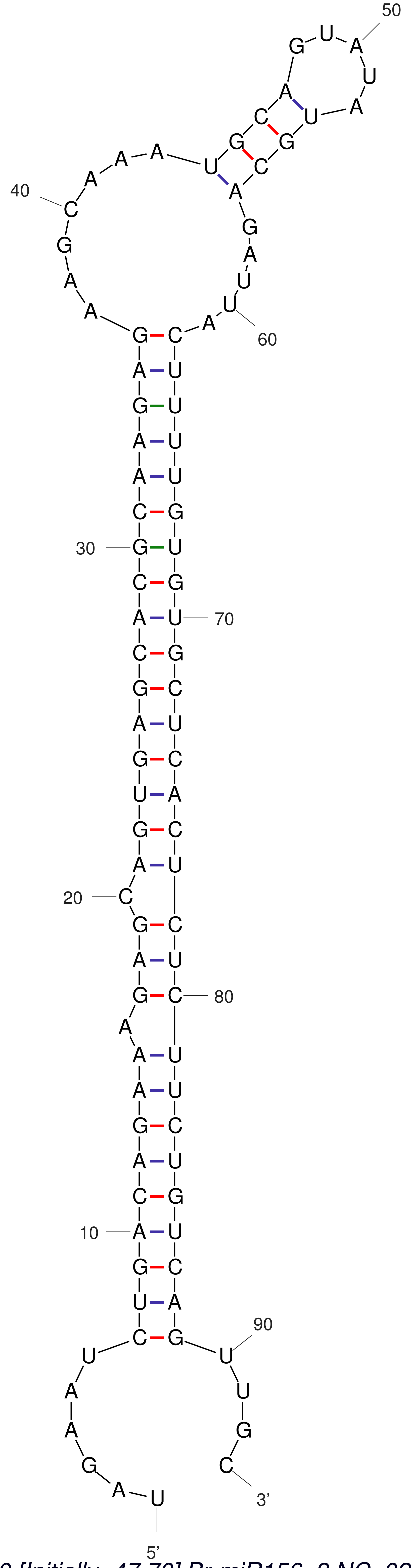

dG = -47.70 [Initially -47.70] Br-miR156_8 NC_024798 1

Output of sir_graph (©)
mfold_util 4.7

Created Mon May 20 23:17:27 2019

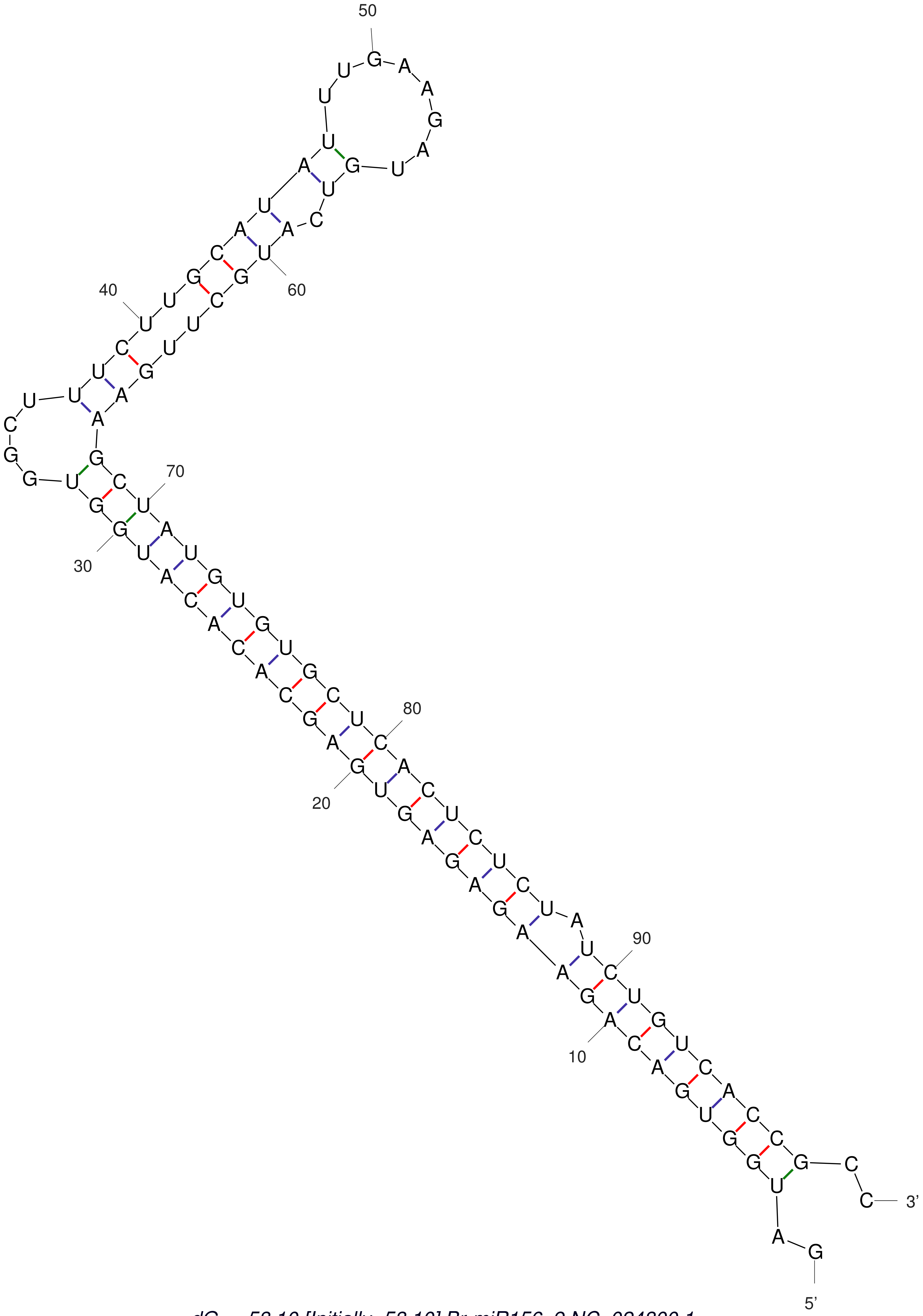

dG = -58.10 [Initially -58.10] Br-miR156_9 NC_024800 1

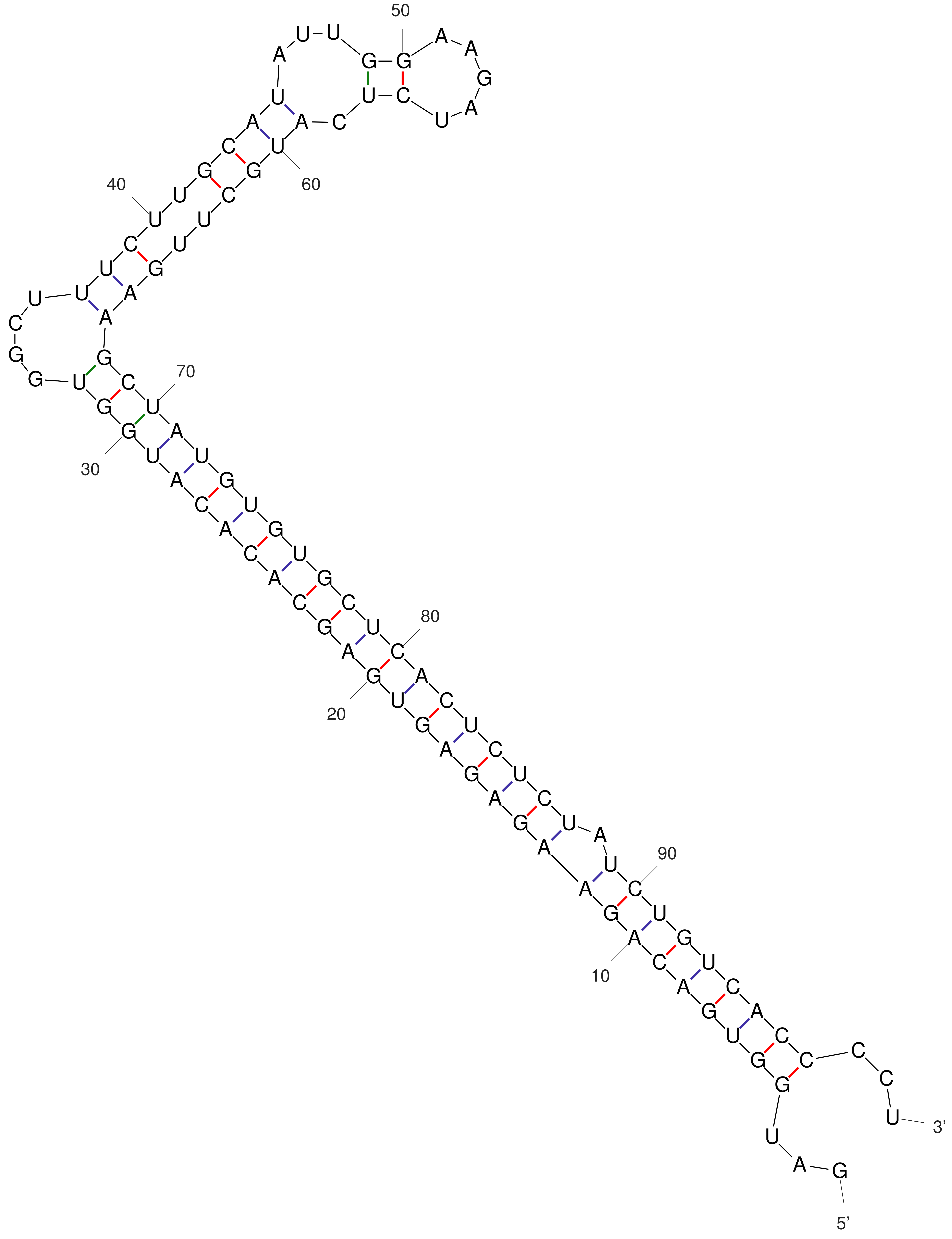

dG = -57.60 [Initially -57.60] Br-miR156_10 NC_024800 1

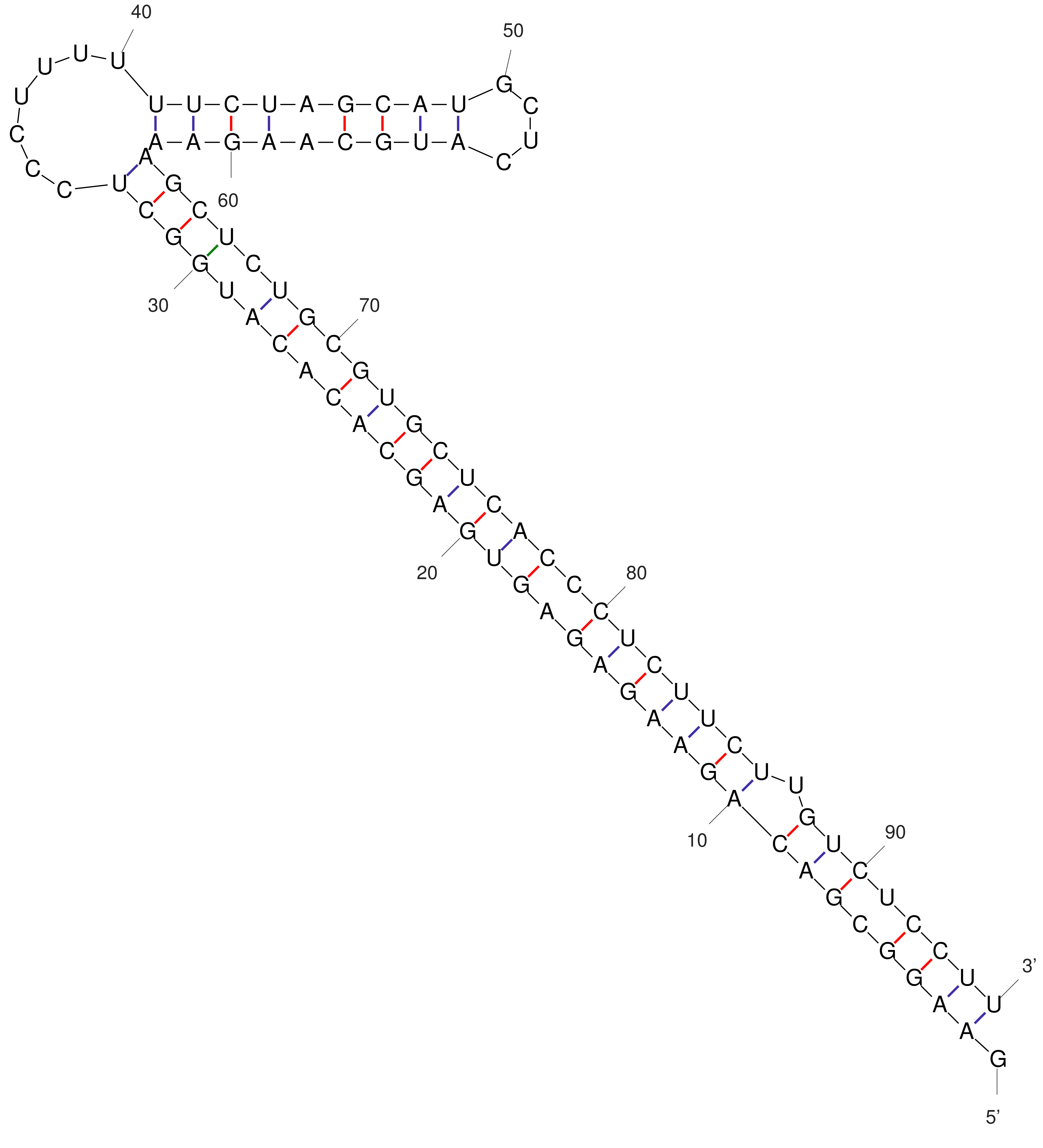

dG = -46.60 [Initially -46.60] Br-miR156_11 NC_024801 1

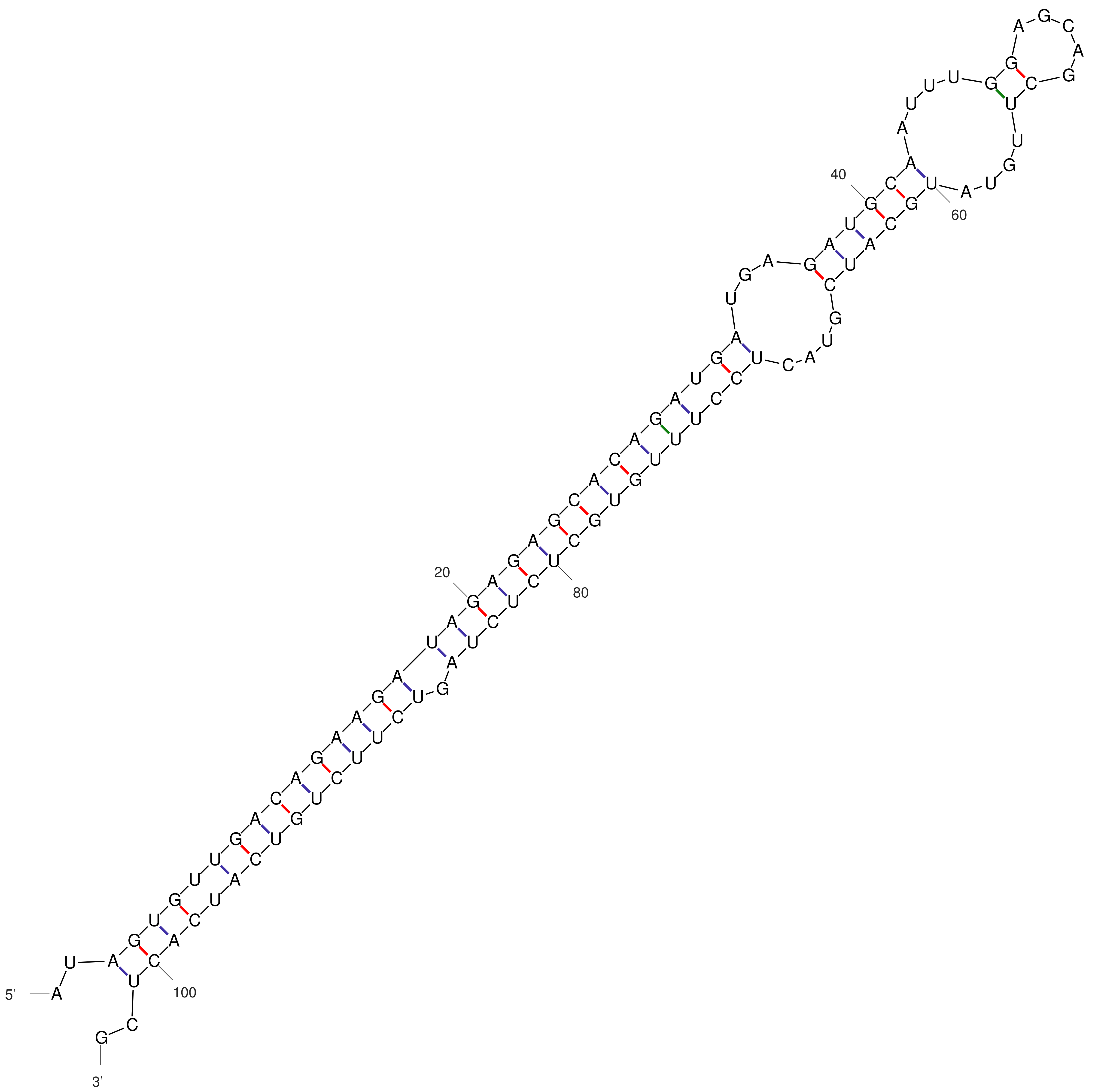

dG = -50.20 [Initially -50.20] Br-miR156_12 NC_024801 1

Output of sir_graph (©)
mfold_util 4.7

Created Mon May 20 23:19:15 2019

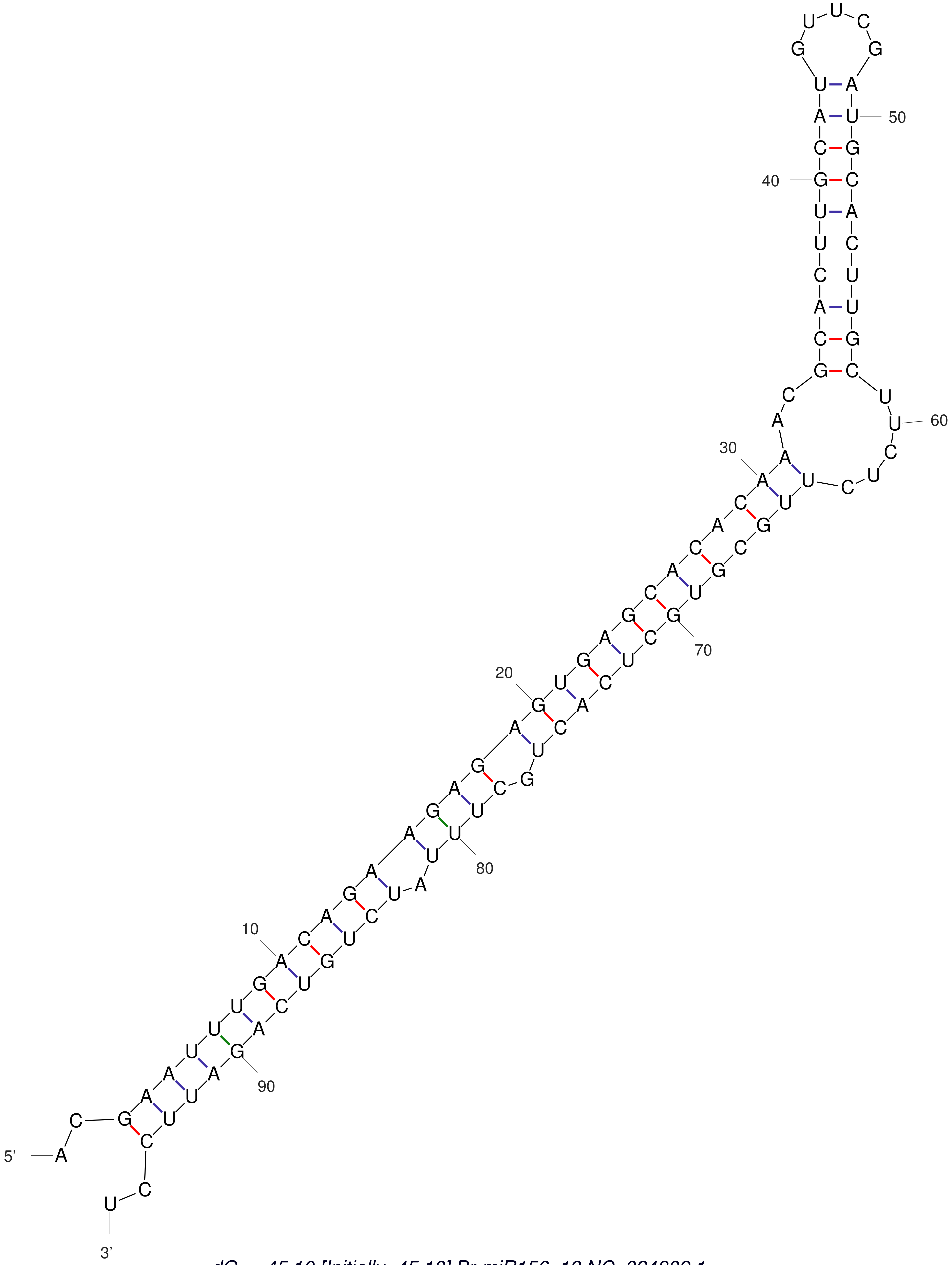

dG = -45.10 [Initially -45.10] Br-miR156_13 NC_024802 1

Output of sir_graph (©)
mfold_util 4.7

Created Mon May 20 23:19:22 2019

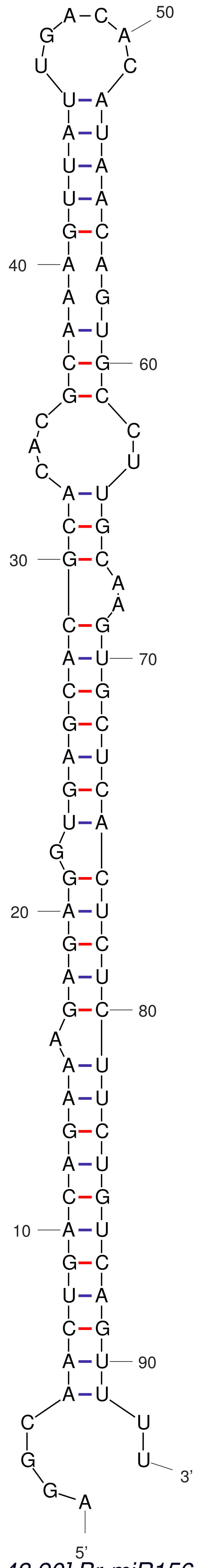

dG = -43.90 [Initially -43.90] Br-miR156_14 NC_024802 1

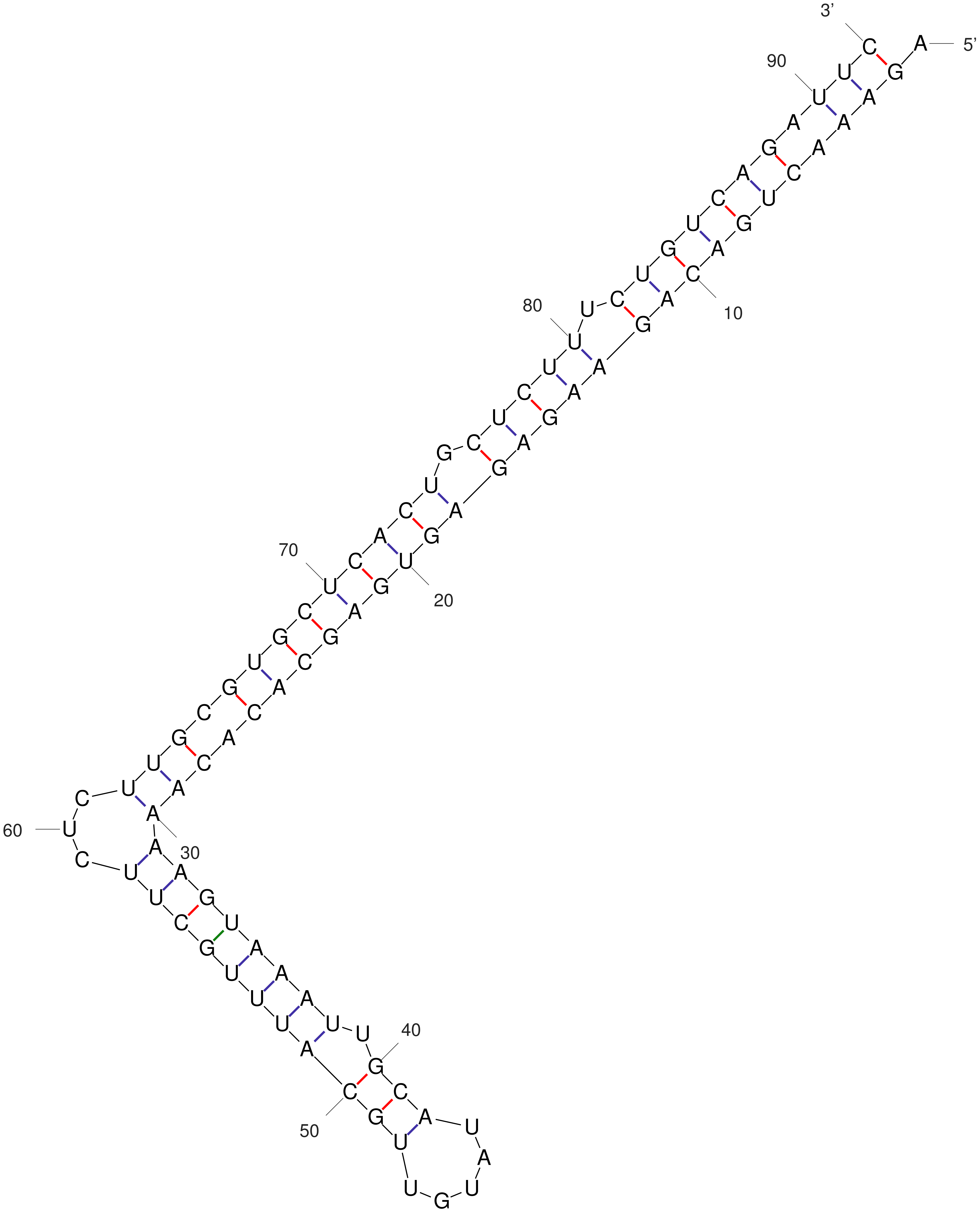

dG = -46.60 [Initially -46.60] Br-miR156_15 NC_024803 1

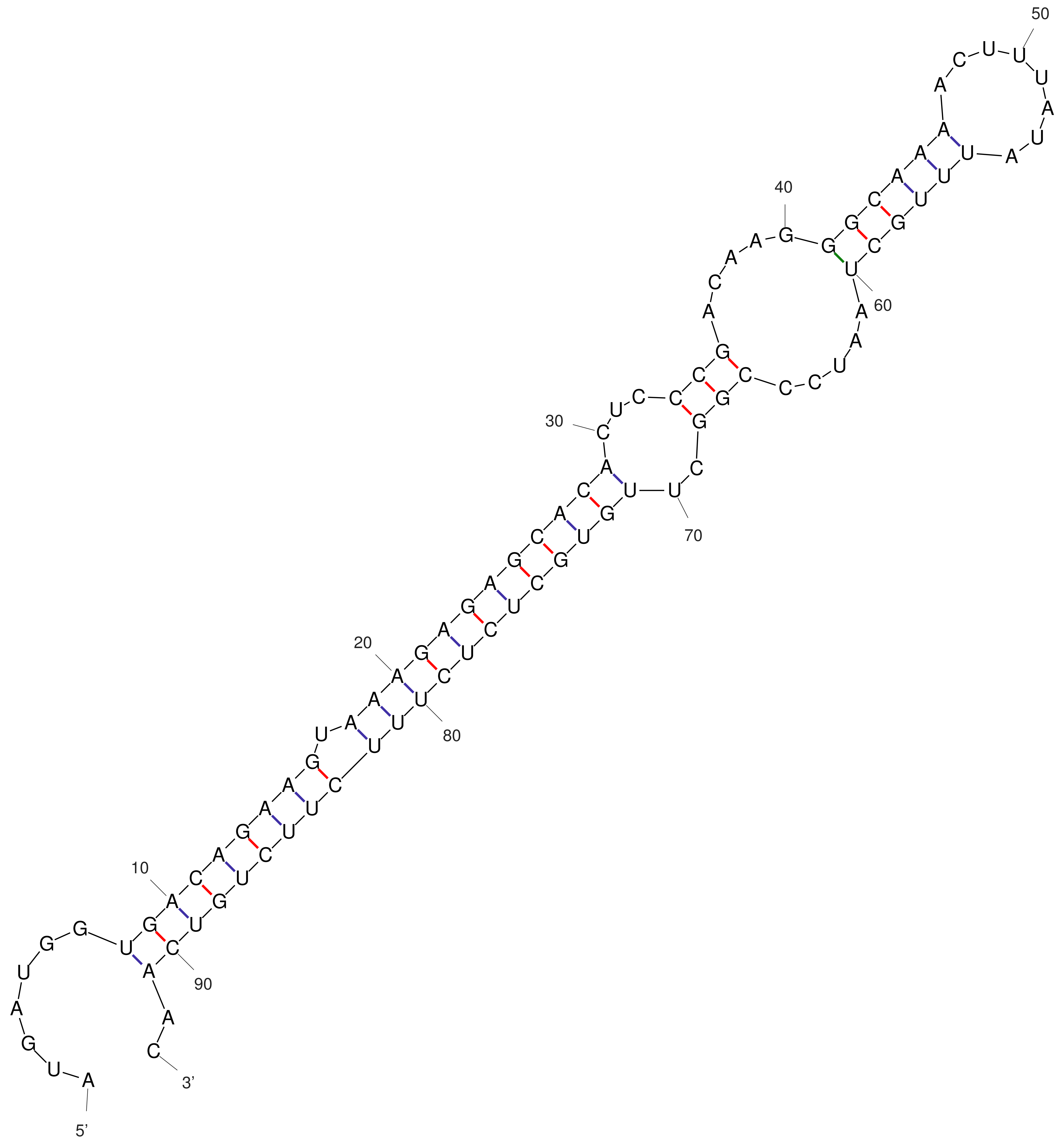

dG = -42.80 [Initially -42.80] Br-miR156_16 NC_024804 1

Output of sir_graph (©)
mfold_util 4.7

Created Tue May 21 00:18:58 2019

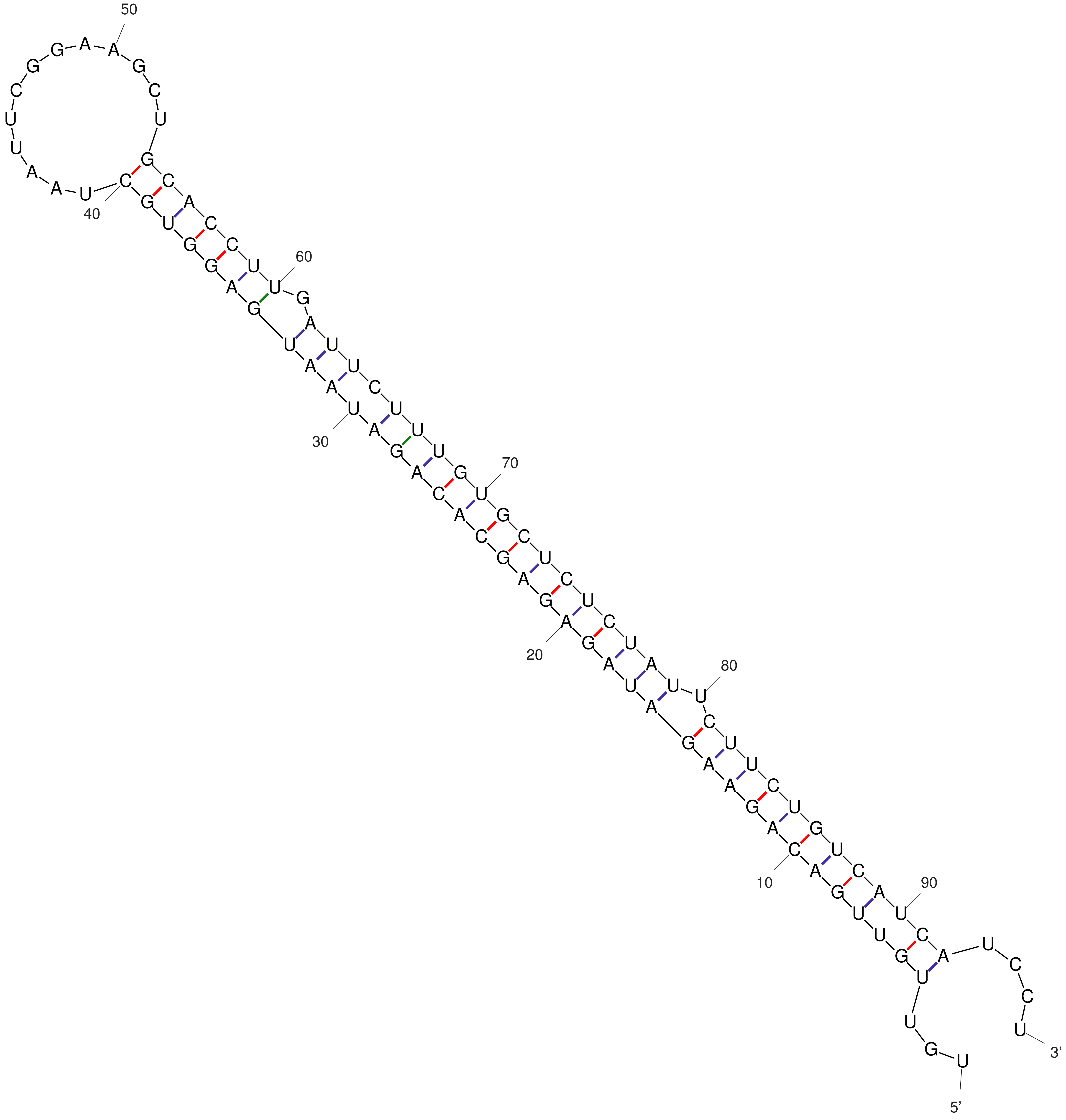

dG = -48.60 [Initially -48.60] Ca-miR156_1 NC_029978 1

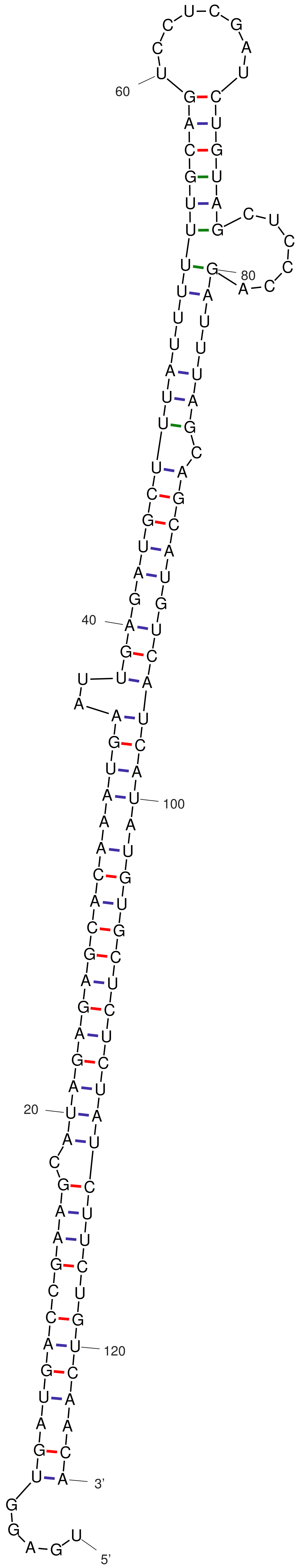

dG = -46.70 [Initially -46.90] Ca-miR156_2 NC_029979 1

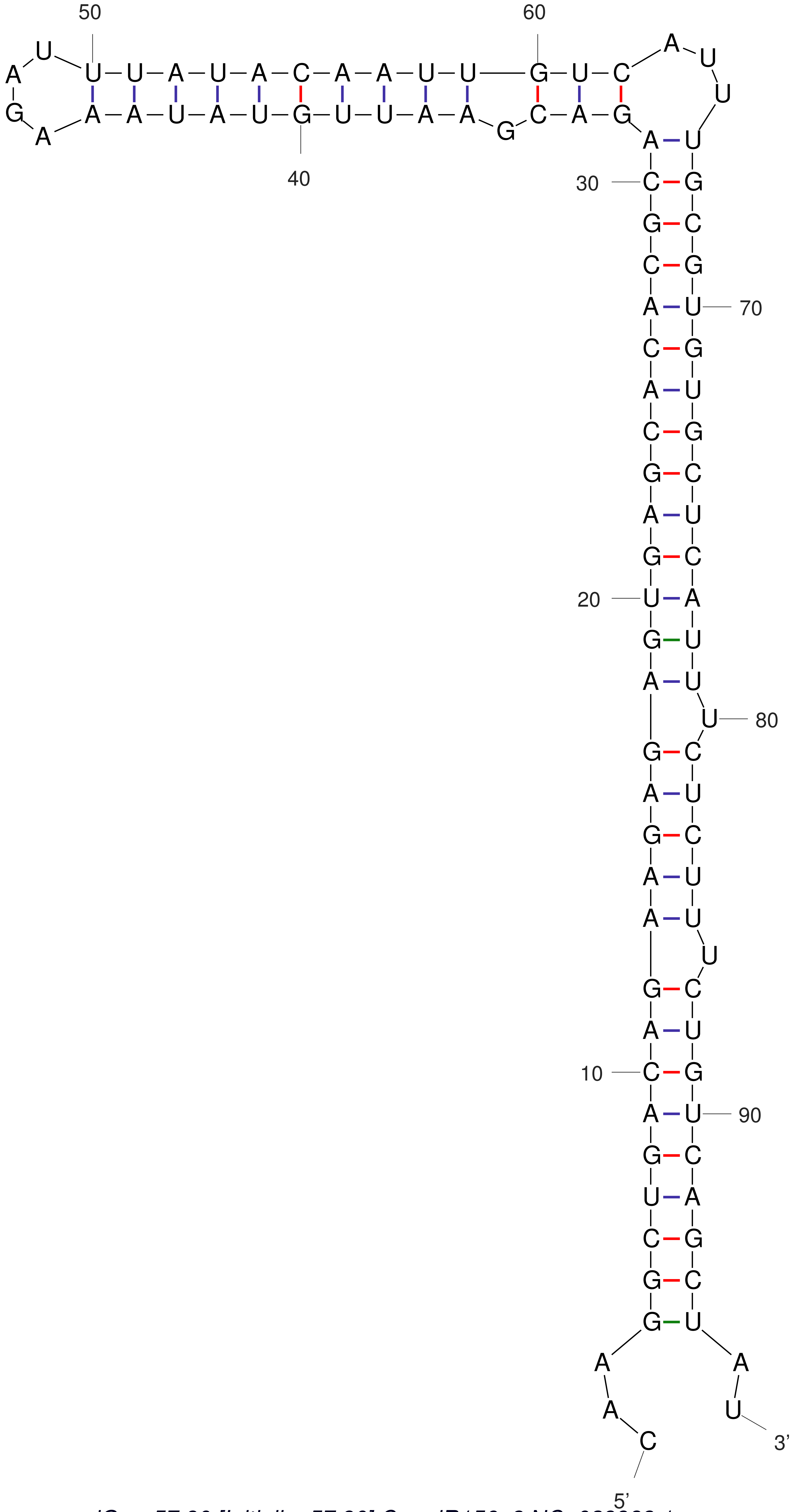

dG = -57.90 [Initially -57.90] Ca-miR156_3 NC_029983 1

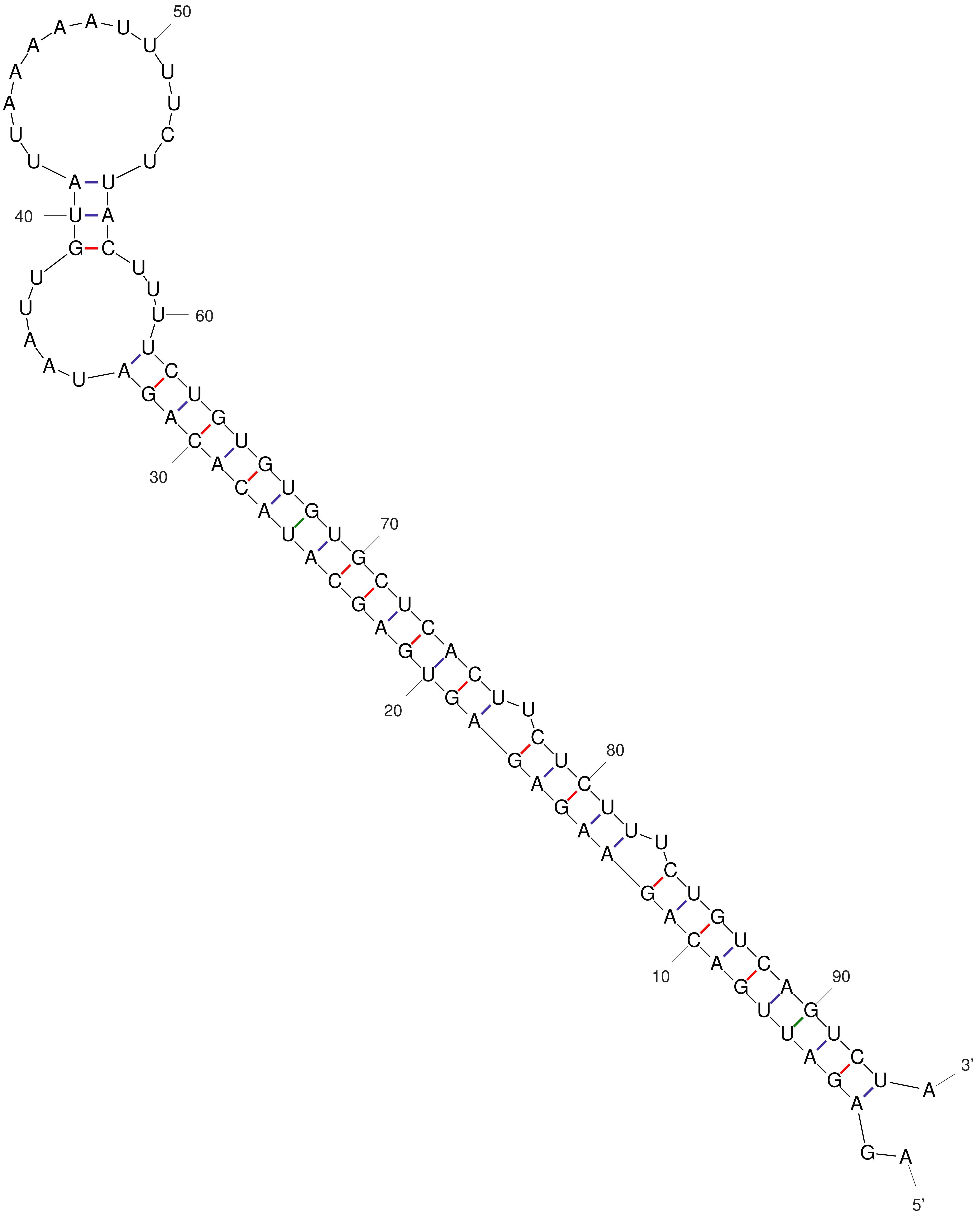

dG = -49.80 [Initially -49.80] Ca-miR156_4 NC_029985 1

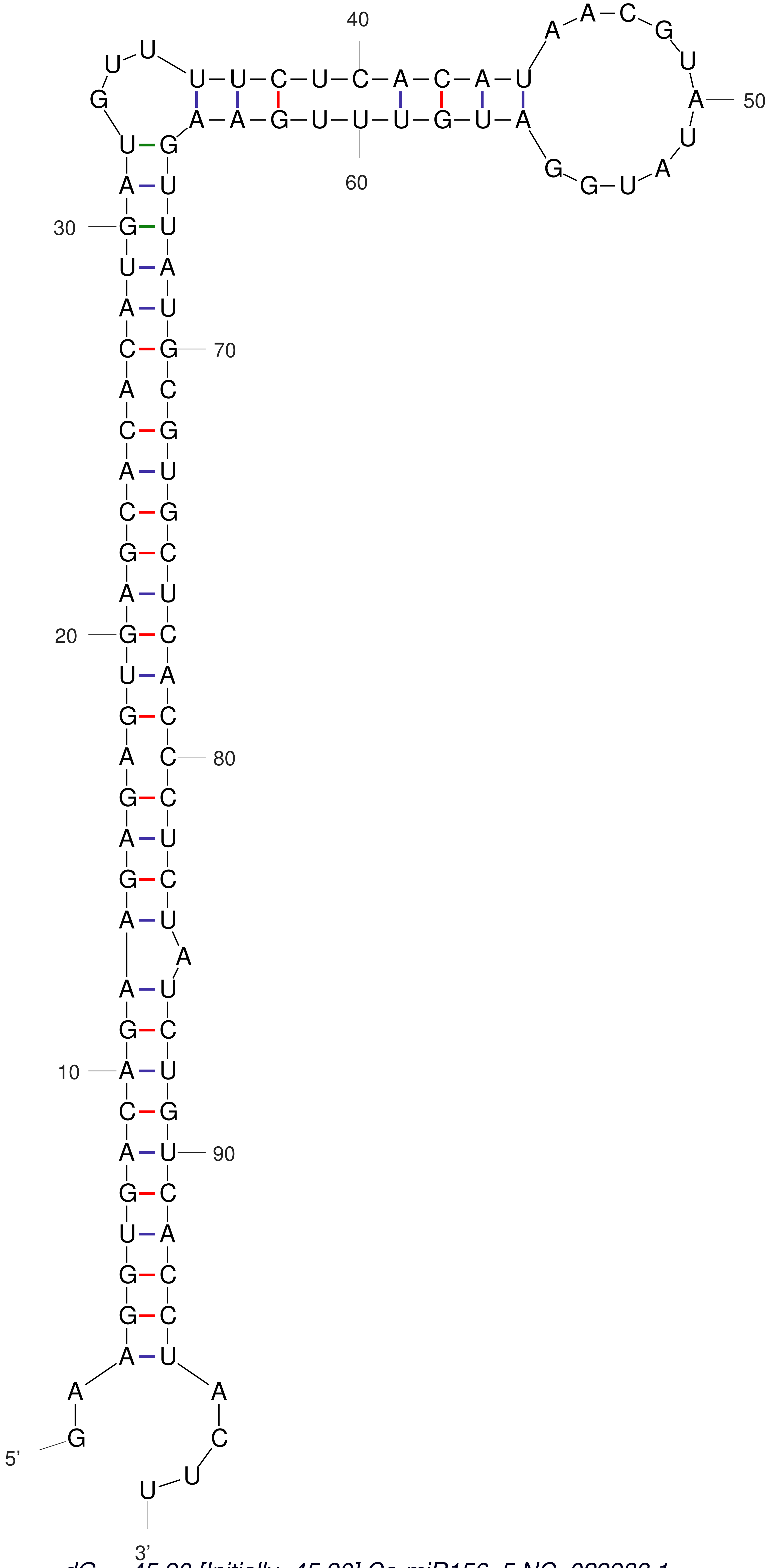

dG = -45.90 [Initially -45.90] Ca-miR156_5 NC_029988 1

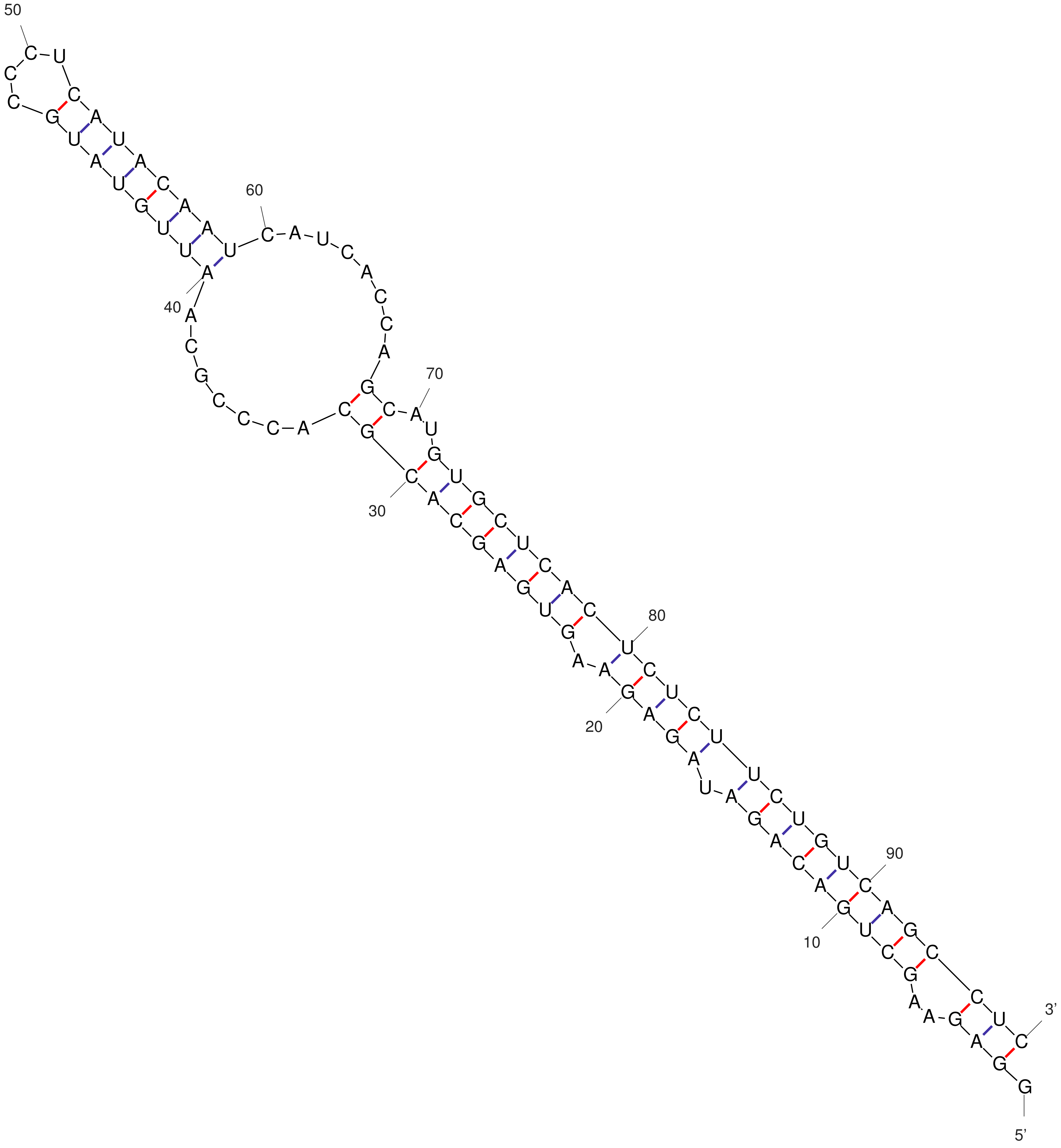

dG = -43.50 [Initially -43.50] Cc-miR156_1 NC_033809 1

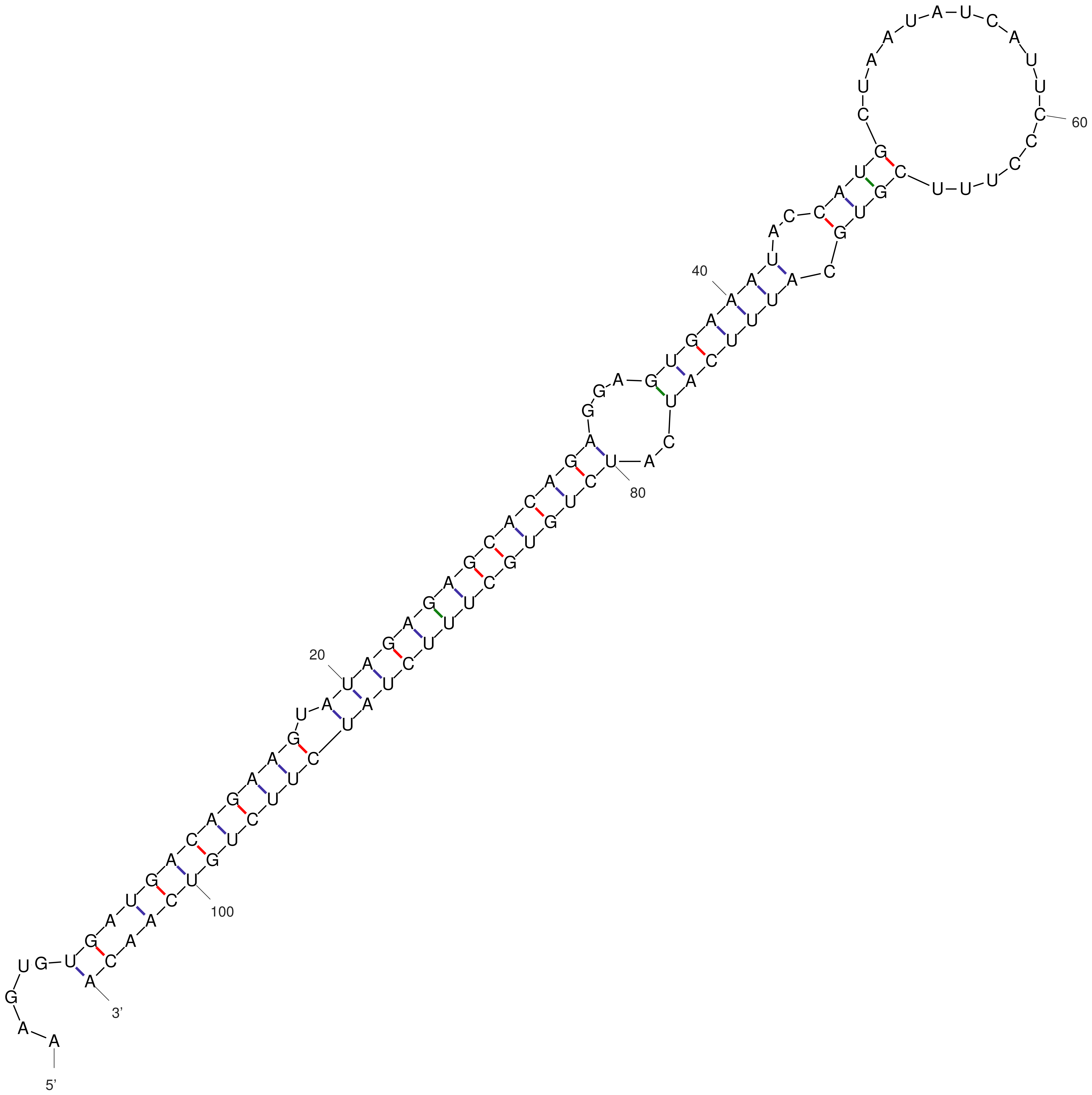

dG = -42.10 [Initially -42.30] Cc-miR156_2 NC_033810 1

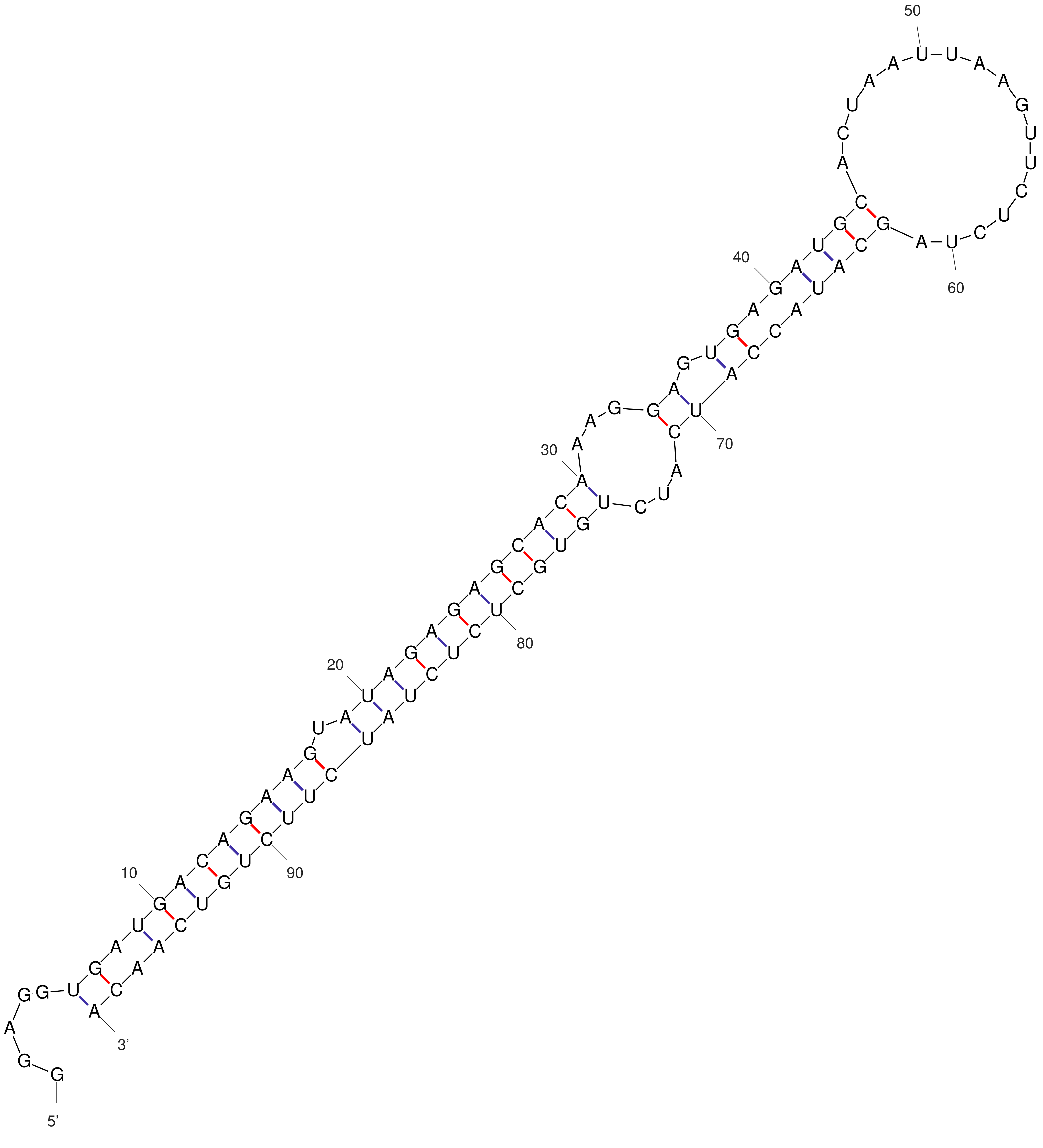

dG = -39.10 [Initially -39.30] Cc-miR156_3 NC_033811 1

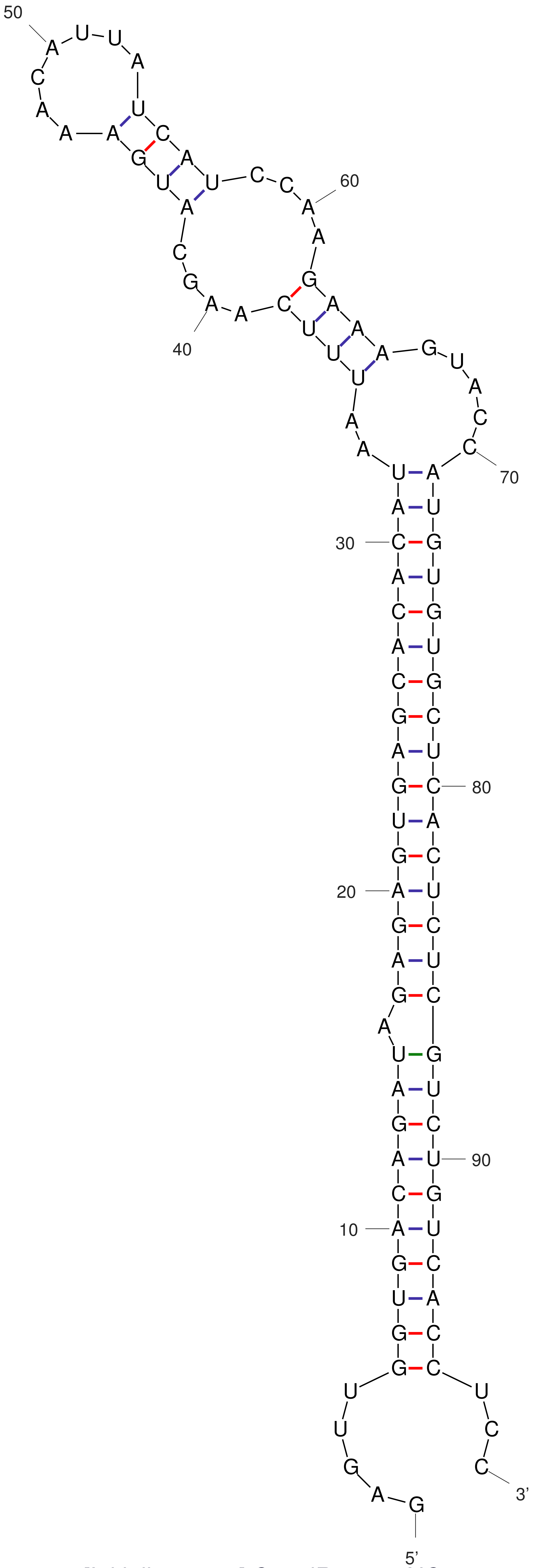

dG = -49.20 [Initially -49.20] Cc-miR156_4 NC_033813 1

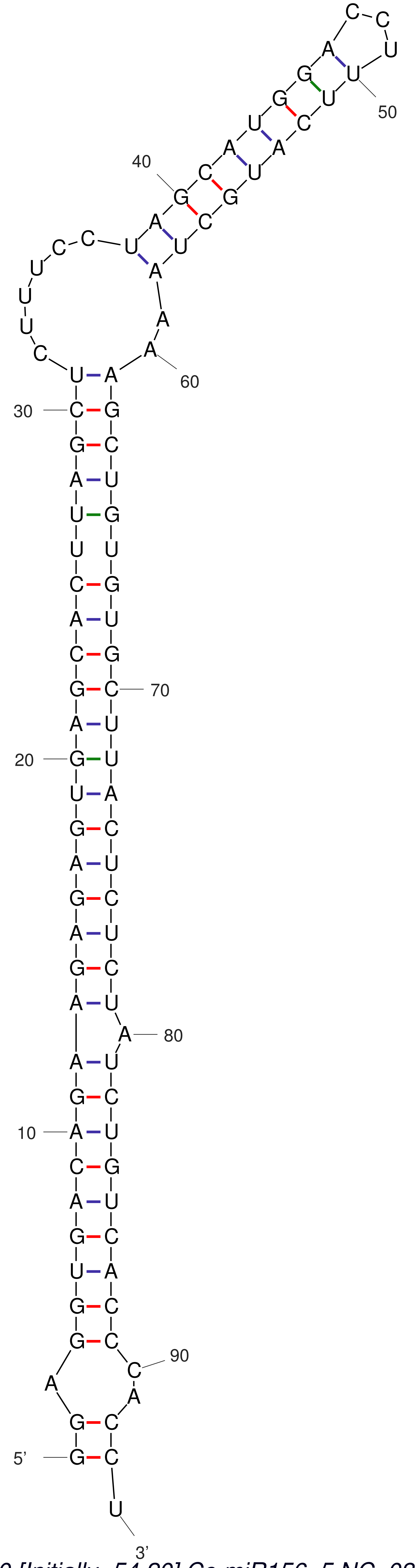

dG = -54.20 [Initially -54.20] Cc-miR156_5 NC_033814 1

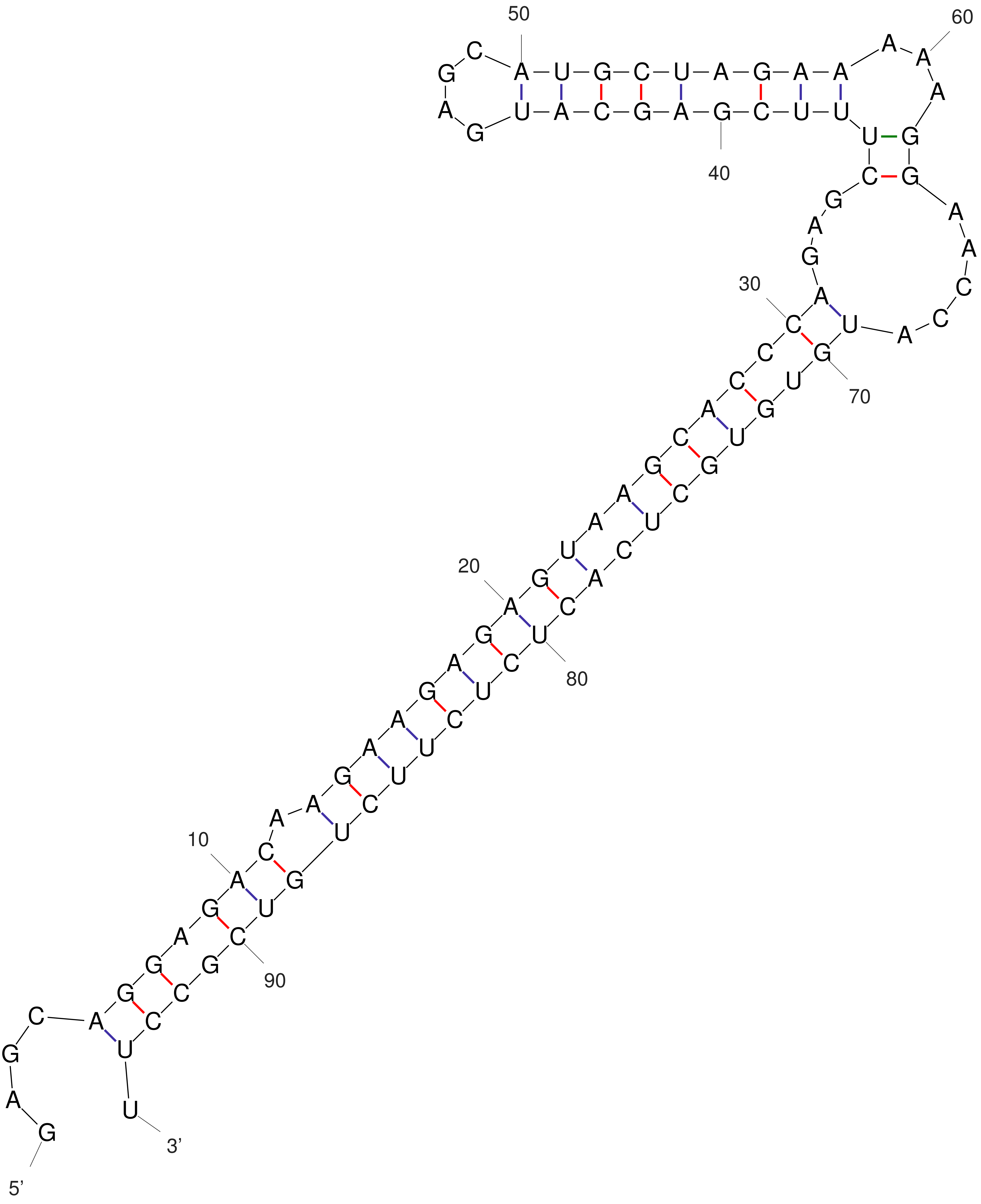

dG = -39.30 [Initially -39.30] Cs-miR156_1 NC_025685 1

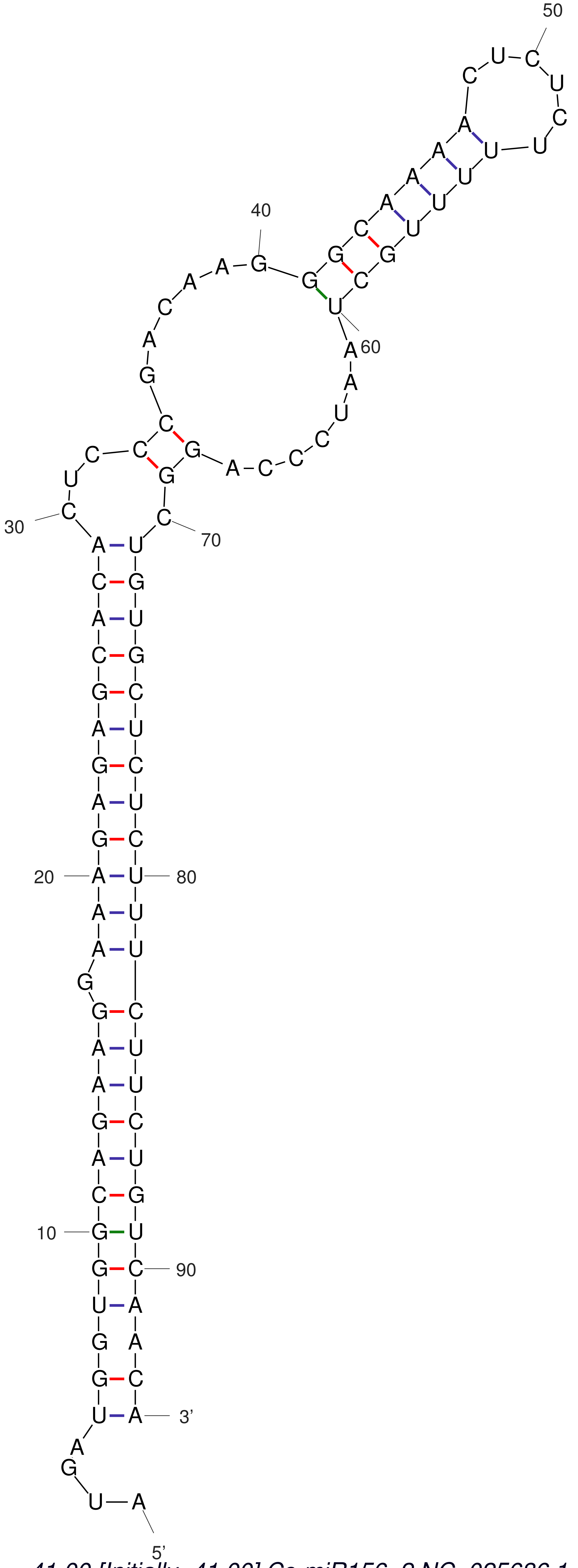

dG = -41.00 [Initially -41.00] Cs-miR156_2 NC_025686 1

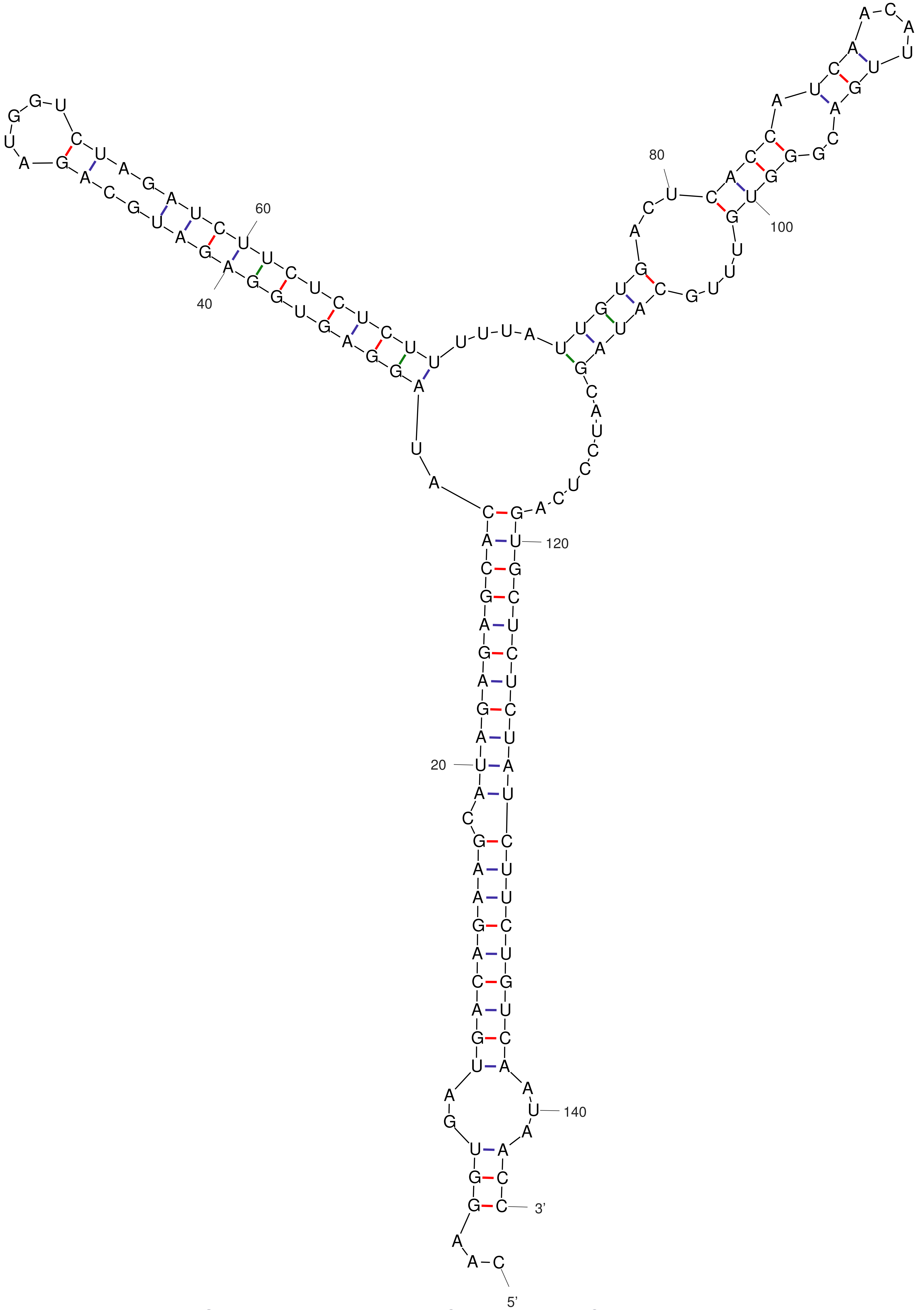

dG = -53.67 [Initially -57.60] Cs-miR156_3 NC_025687 1

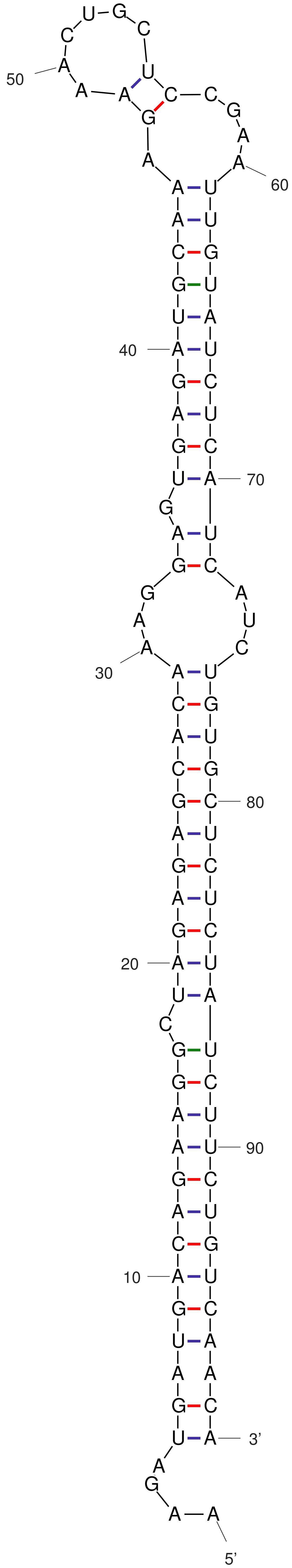

dG = -46.60 [Initially -46.60] Cs-miR156_4 NC_025689 1

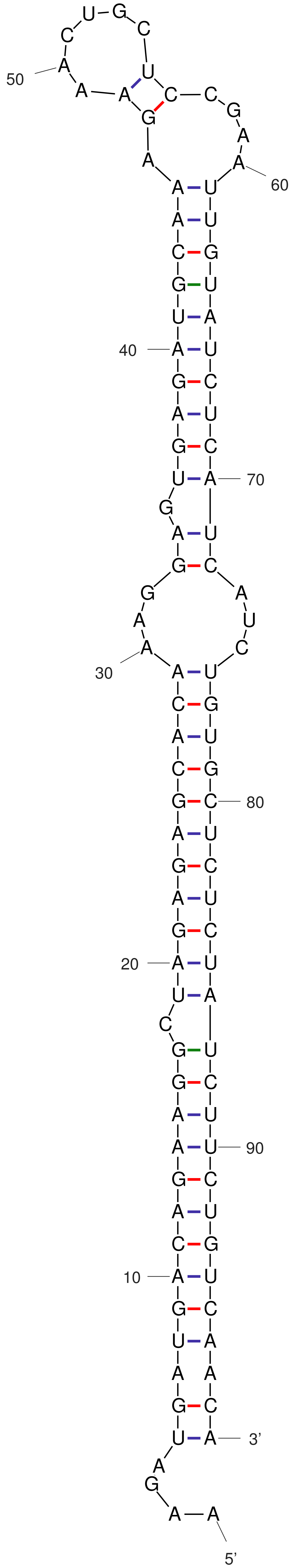

dG = -46.60 [Initially -46.60] Cs-miR156_5 NC_025691 1

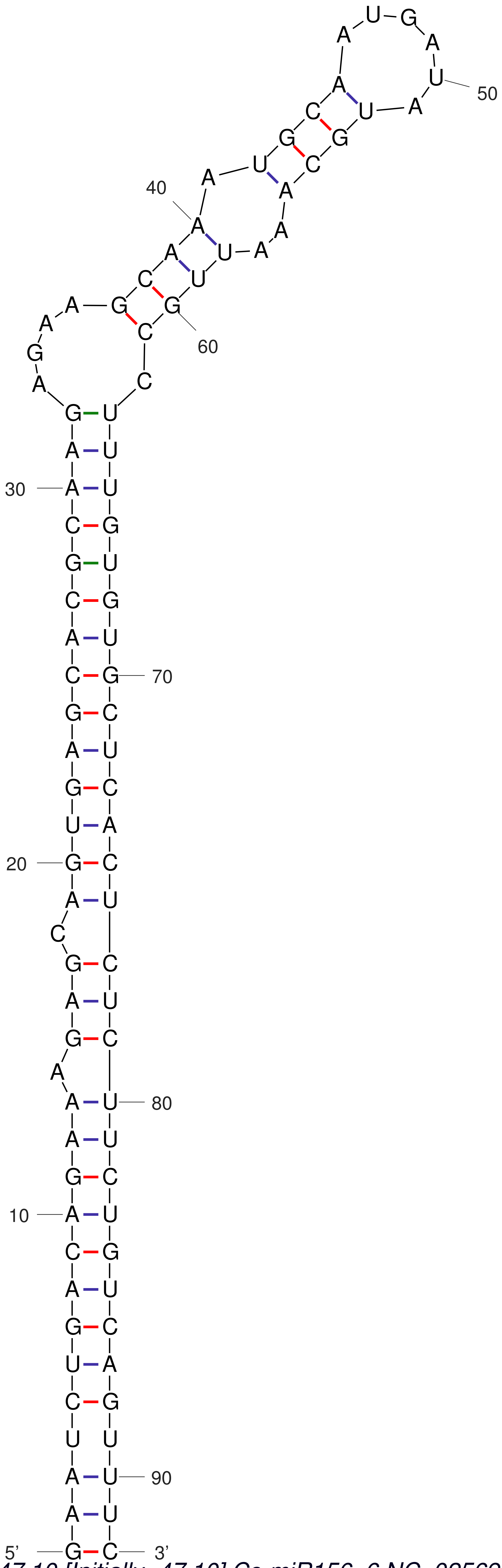

dG = -47.10 [Initially -47.10] Cs-miR156_6 NC_025691 1

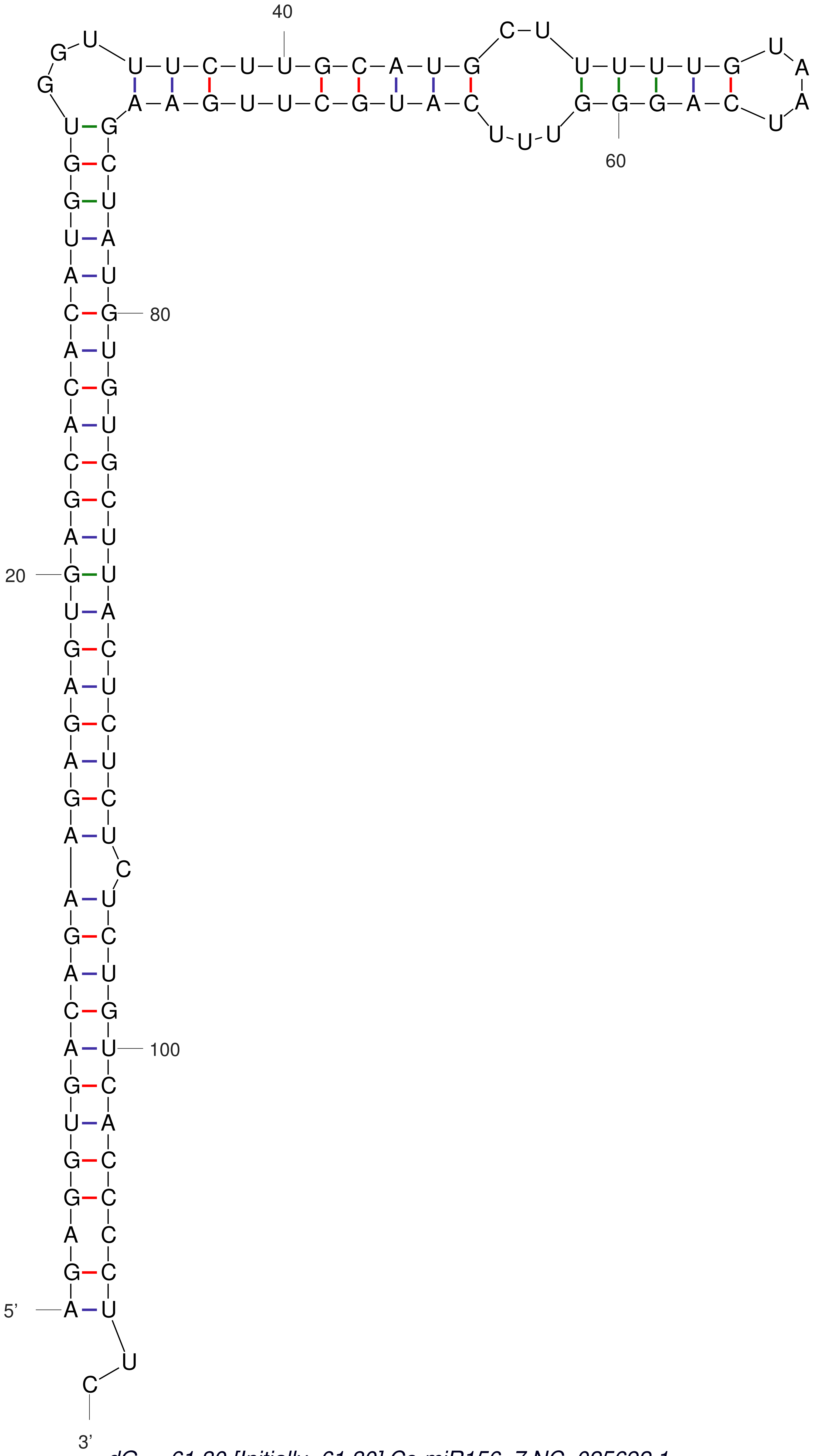

dG = -61.30 [Initially -61.30] Cs-miR156_7 NC_025692 1

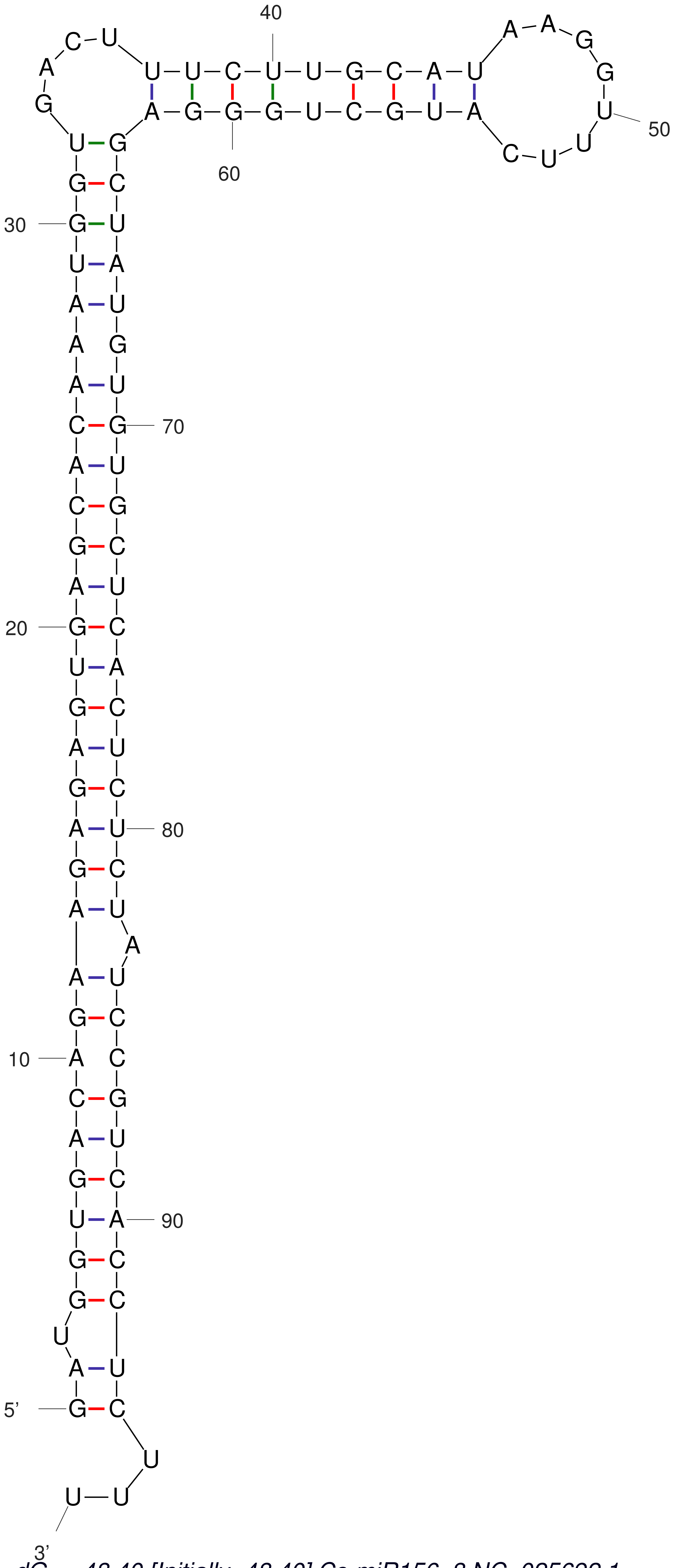

dG = -48.40 [Initially -48.40] Cs-miR156_8 NC_025692 1

Created Tue May 21 00:23:02 2019

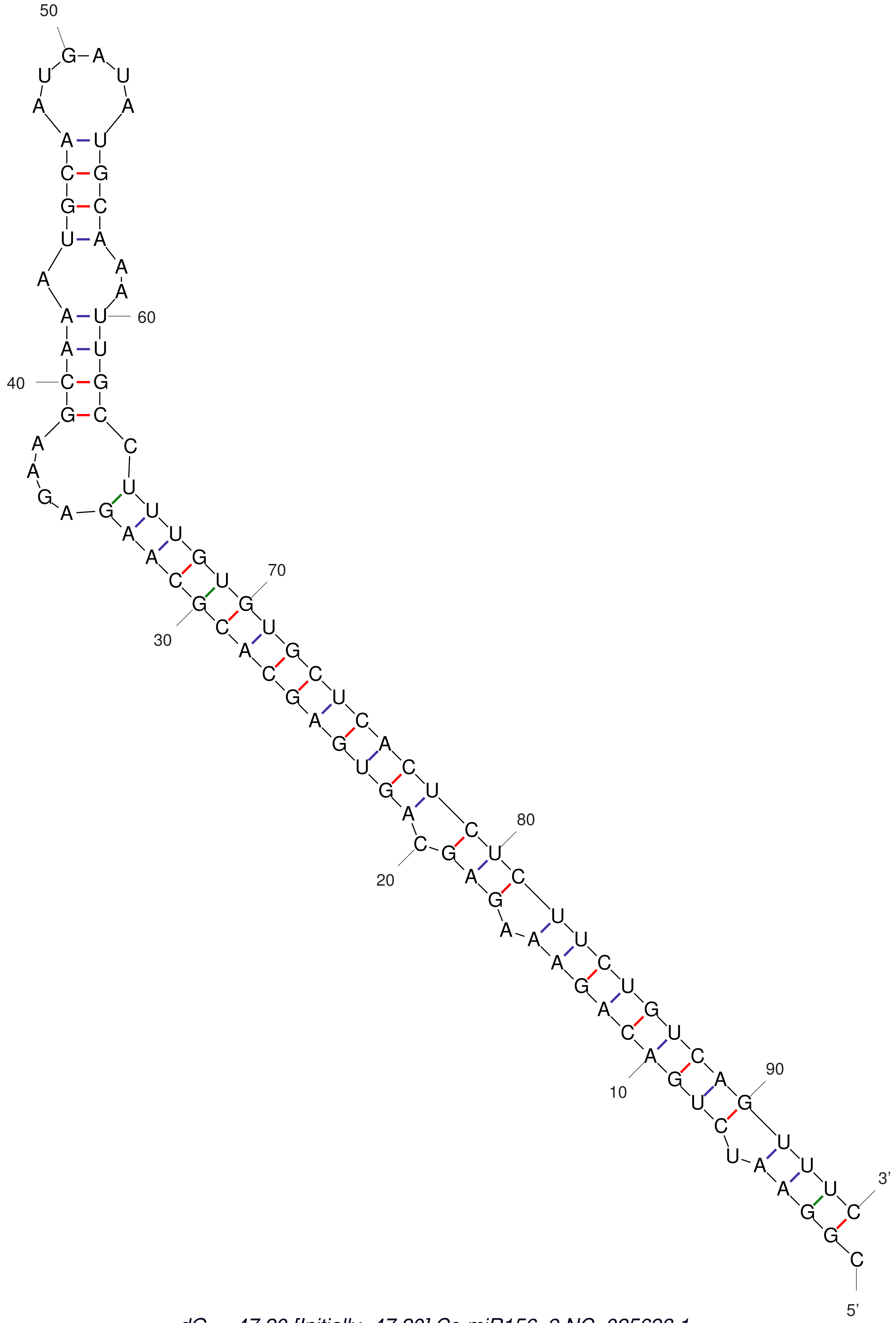

dG = -47.20 [Initially -47.20] Cs-miR156_9 NC_025693 1

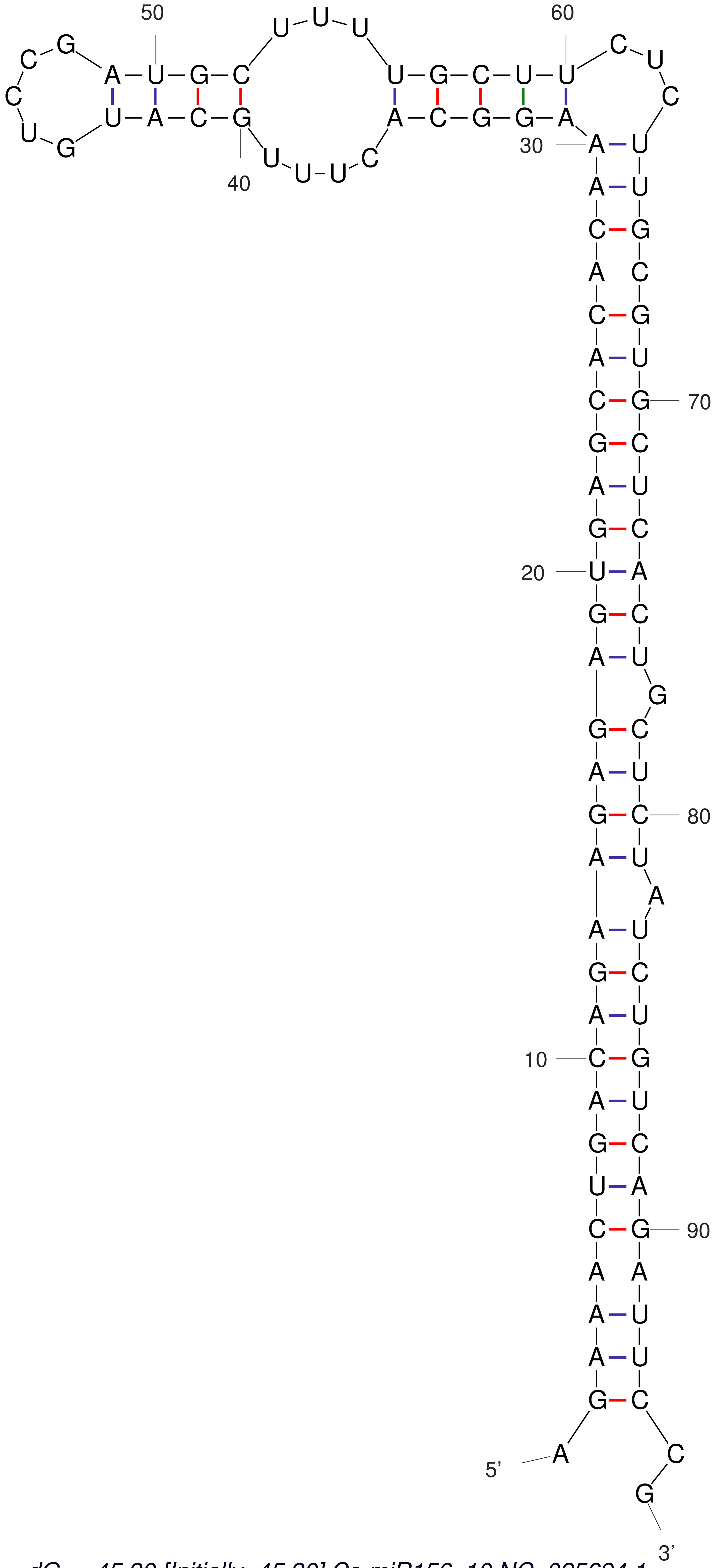

dG = -45.90 [Initially -45.90] Cs-miR156_10 NC_025694 1

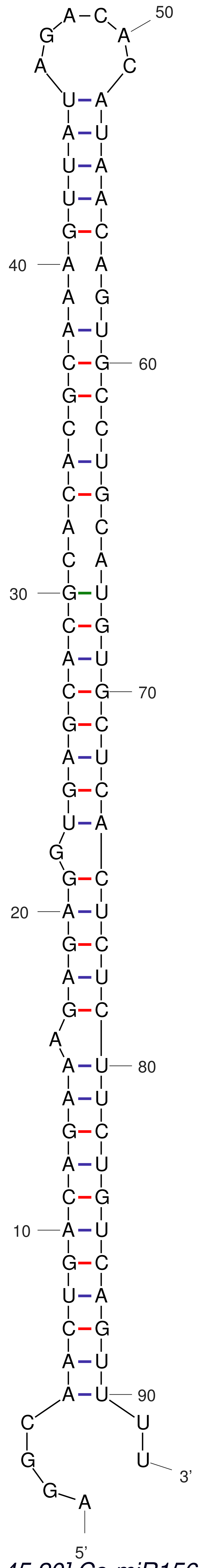

dG = -45.20 [Initially -45.20] Cs-miR156_11 NC_025694 1

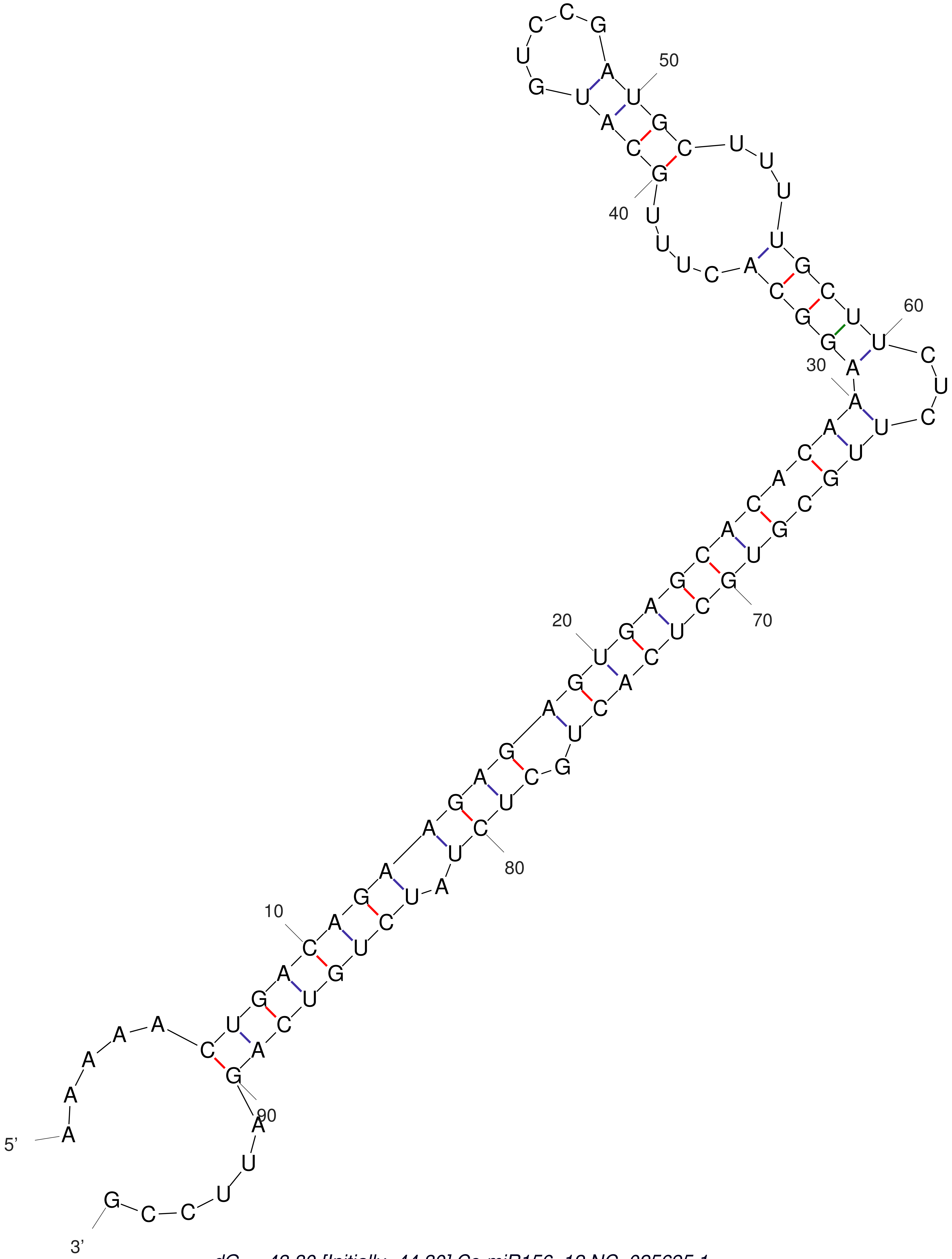

dG = -43.80 [Initially -44.30] Cs-miR156_12 NC_025695 1

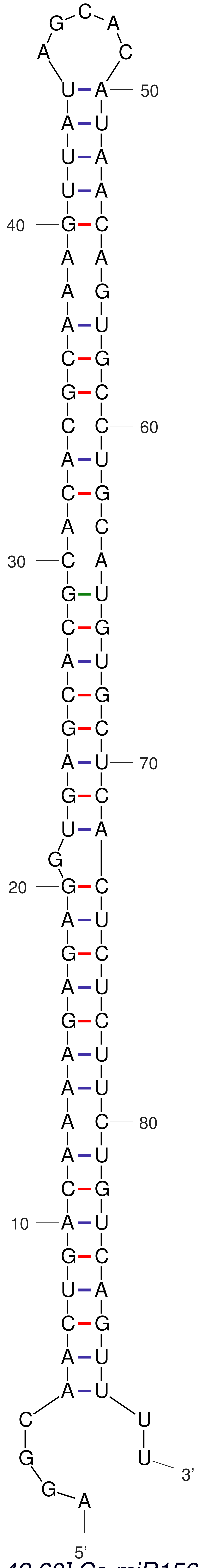

dG = -42.60 [Initially -42.60] Cs-miR156_13 NC_025695 1

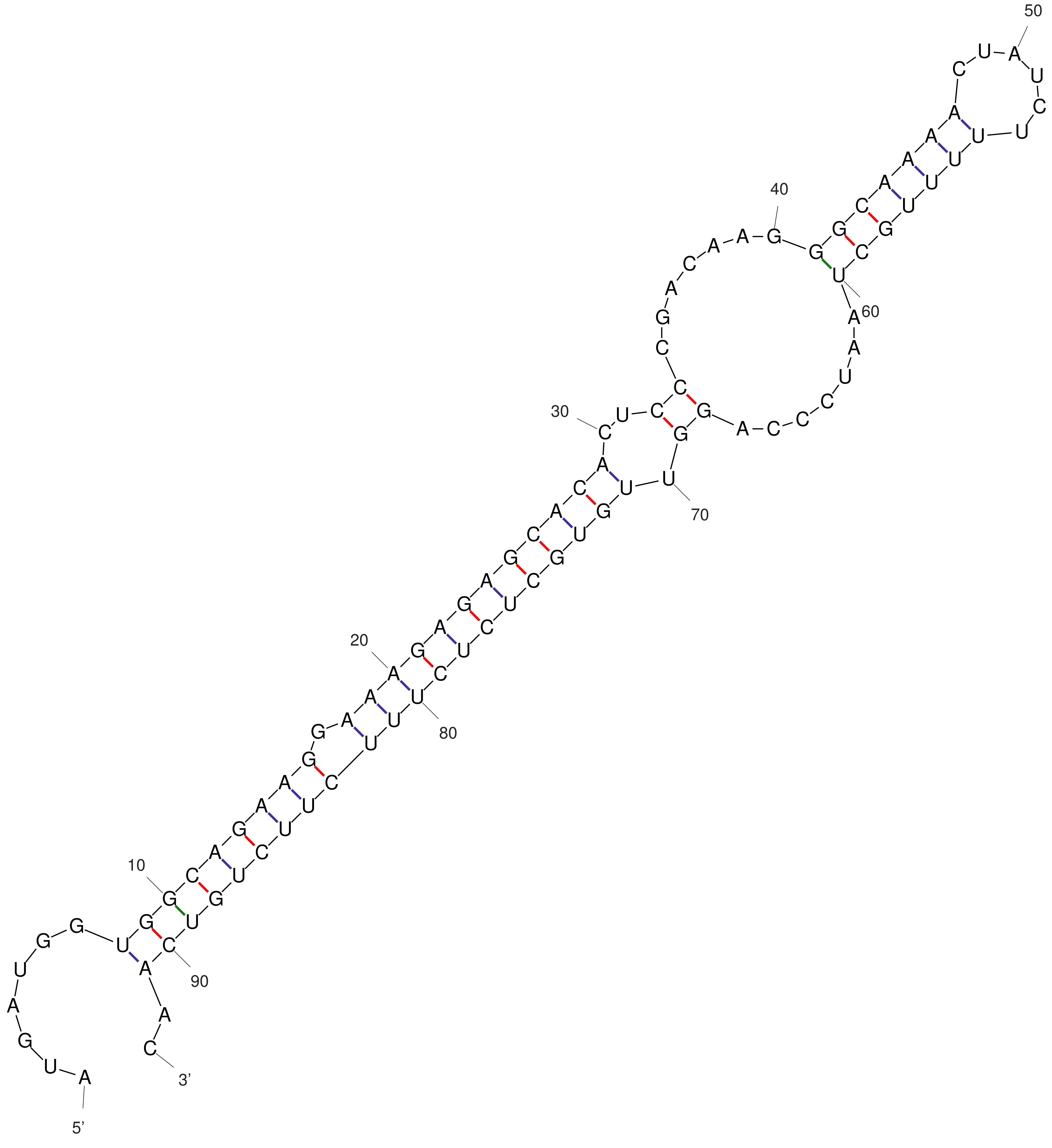

dG = -41.30 [Initially -41.30] Cs-miR156_14 NC_025695 1

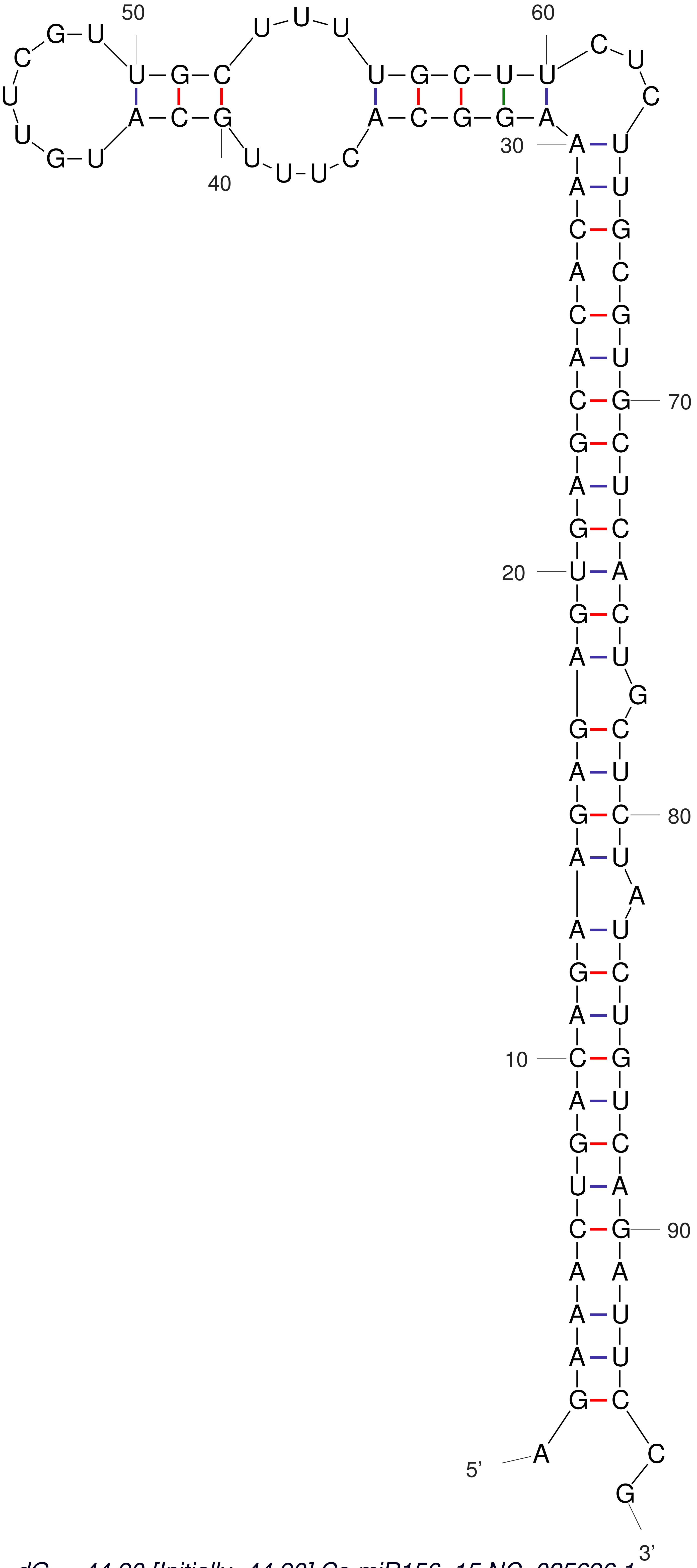

dG = -44.90 [Initially -44.90] Cs-miR156_15 NC_025696 1

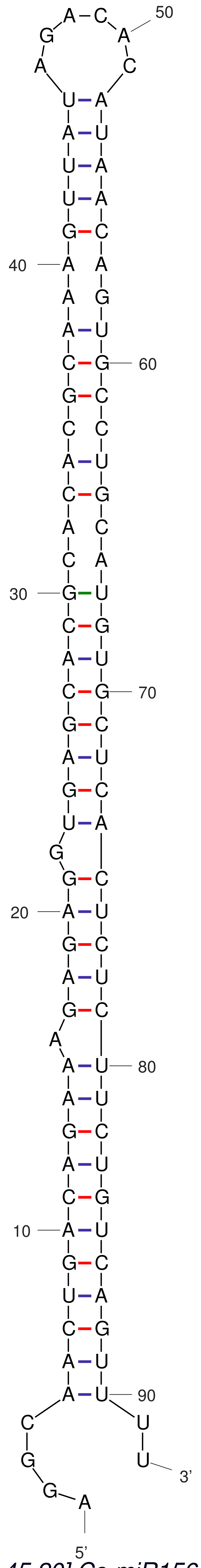

dG = -45.20 [Initially -45.20] Cs-miR156_16 NC_025696 1

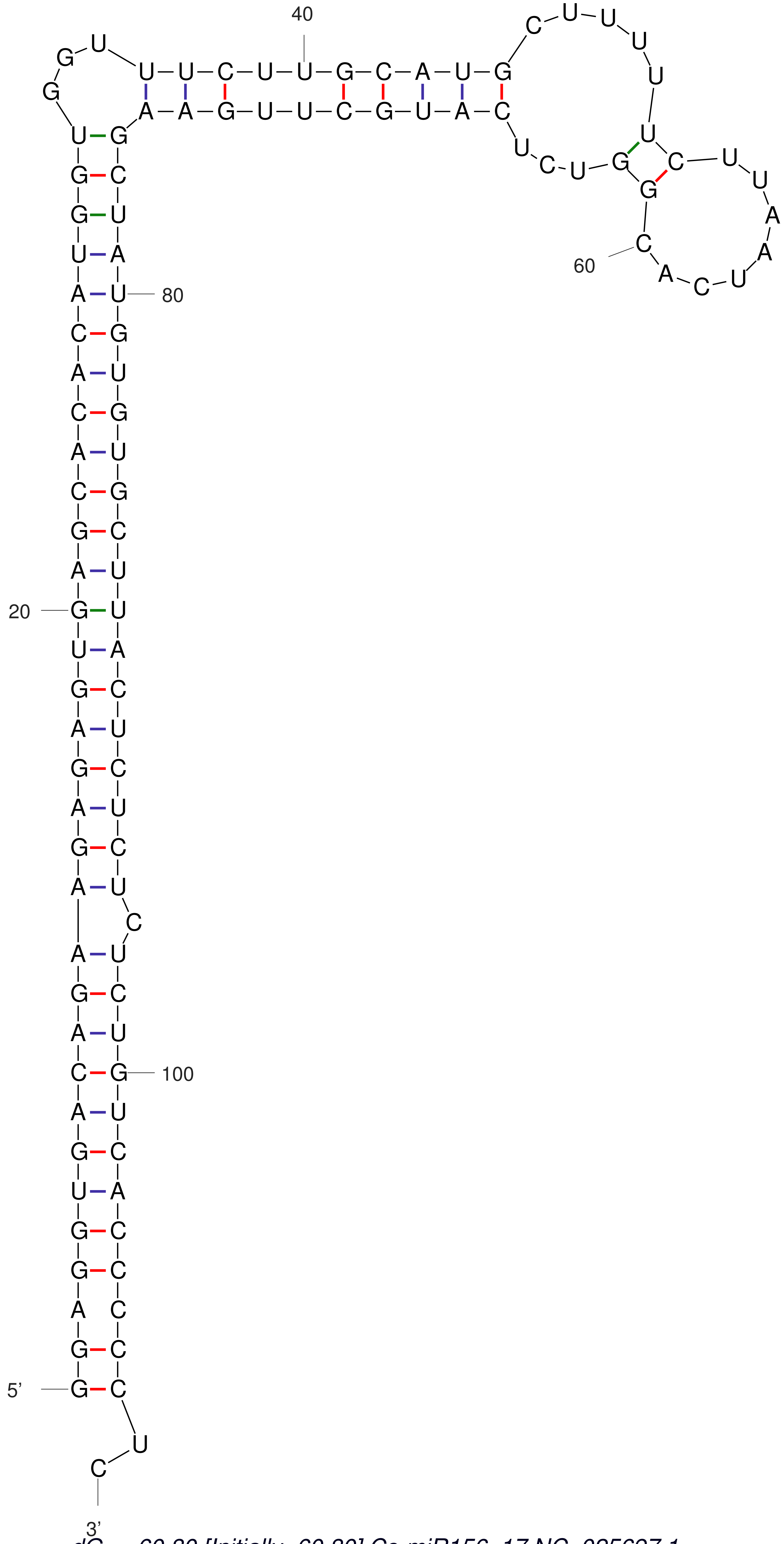

dG = -60.80 [Initially -60.80] Cs-miR156_17 NC_025697 1

Output of sir_graph (©)
mfold_util 4.7

Created Tue May 21 00:26:19 2019

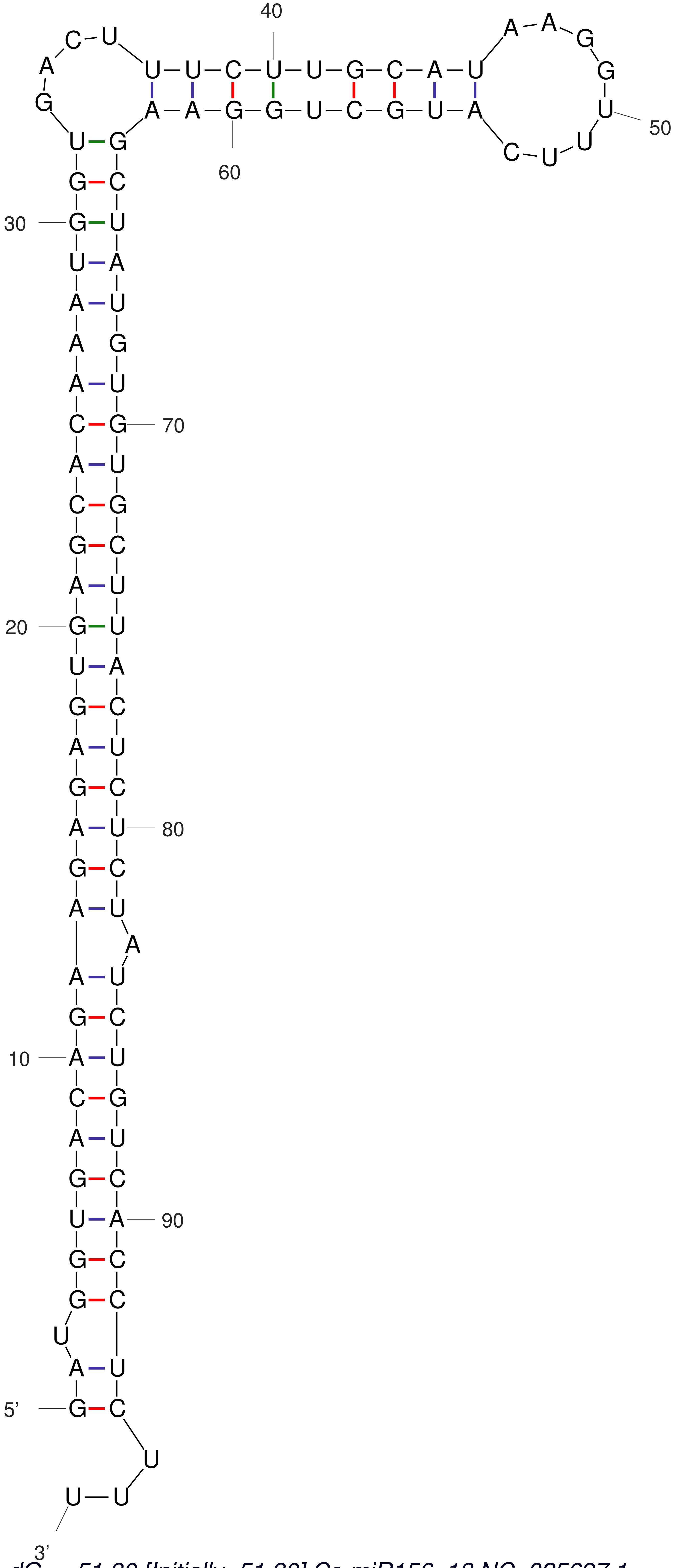

dG = -51.30 [Initially -51.30] Cs-miR156_18 NC_025697 1

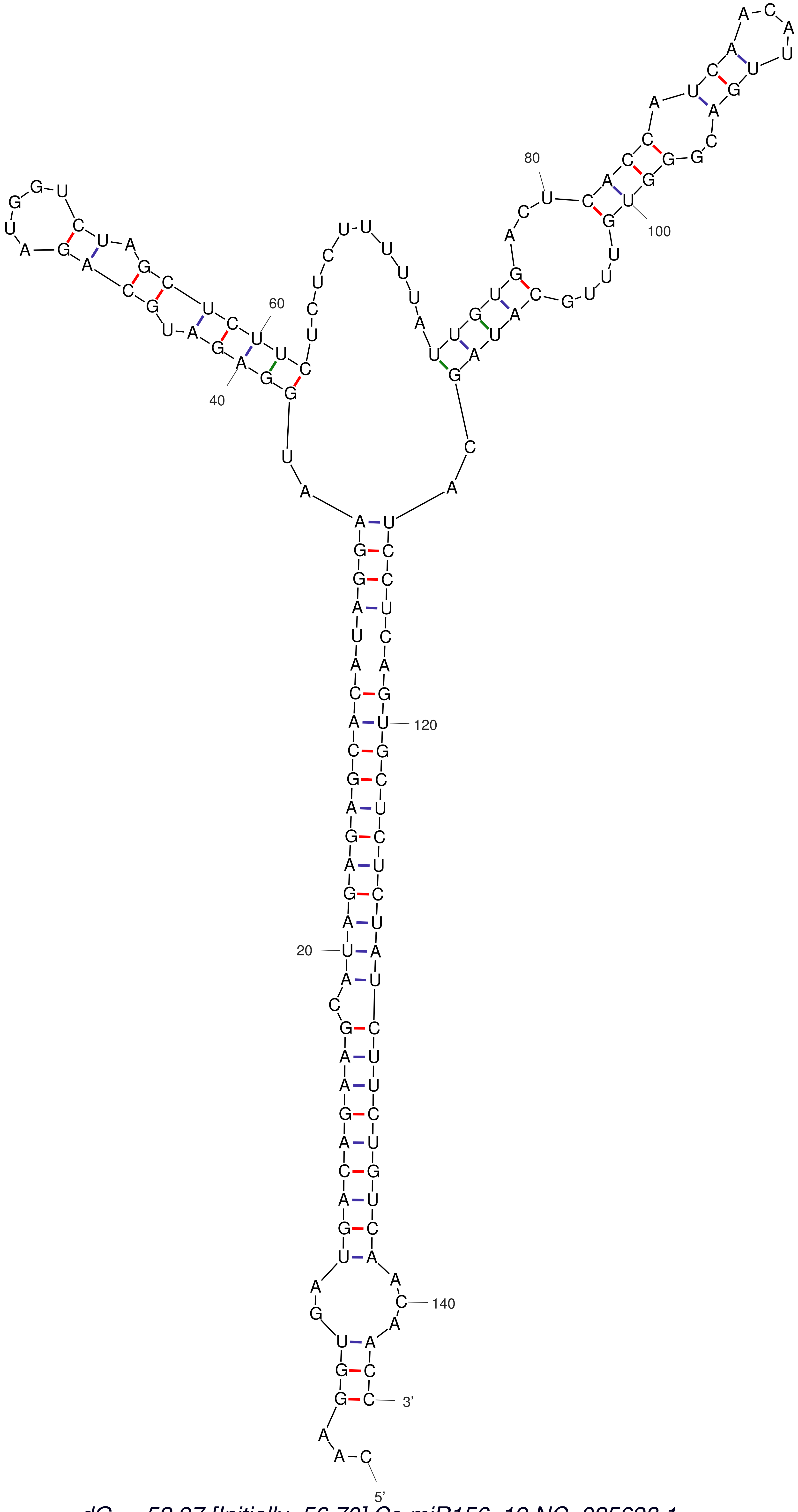

dG = -52.97 [Initially -56.70] Cs-miR156_19 NC_025698 1

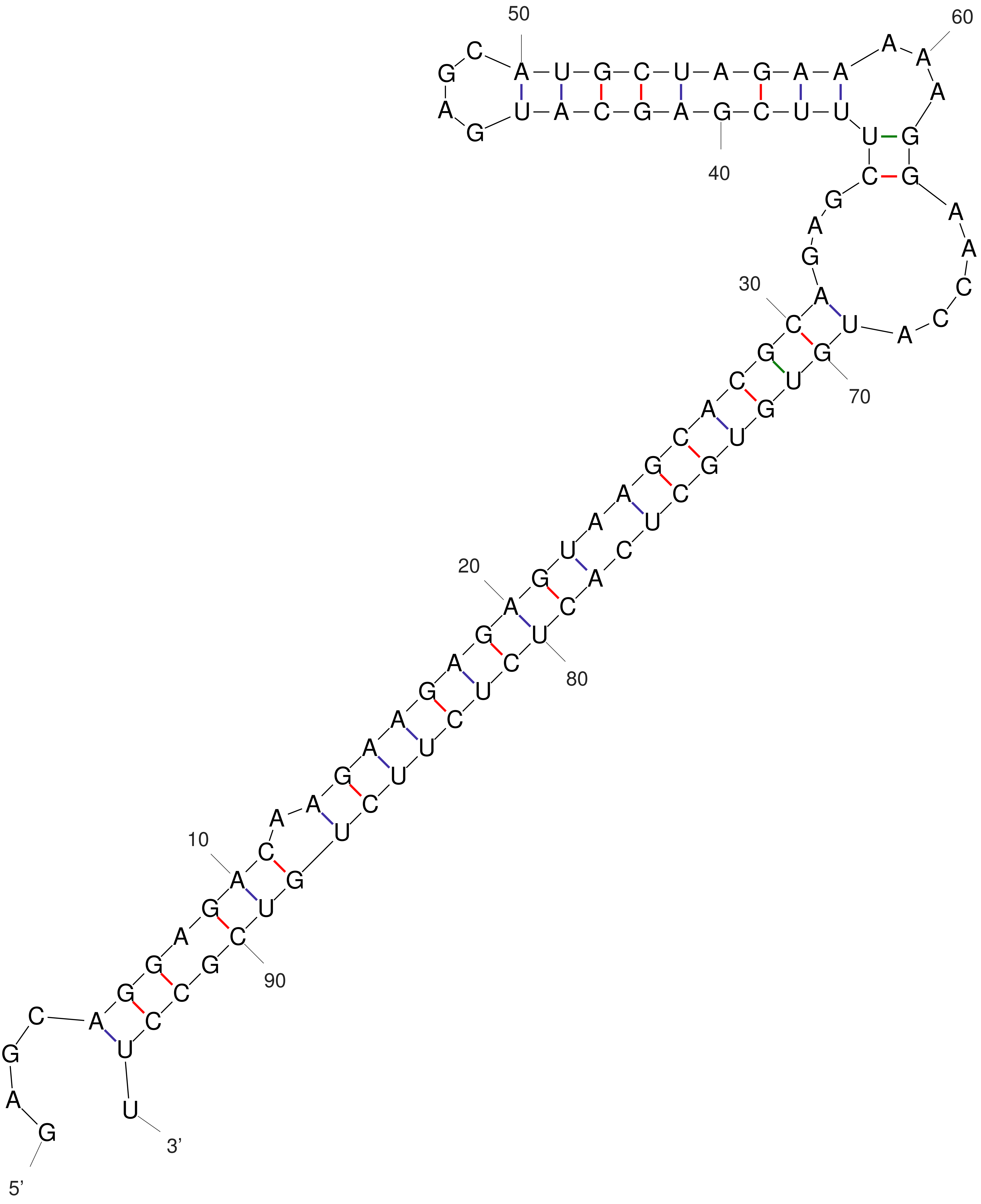

dG = -43.70 [Initially -43.70] Cs-miR156_20 NC_025699 1

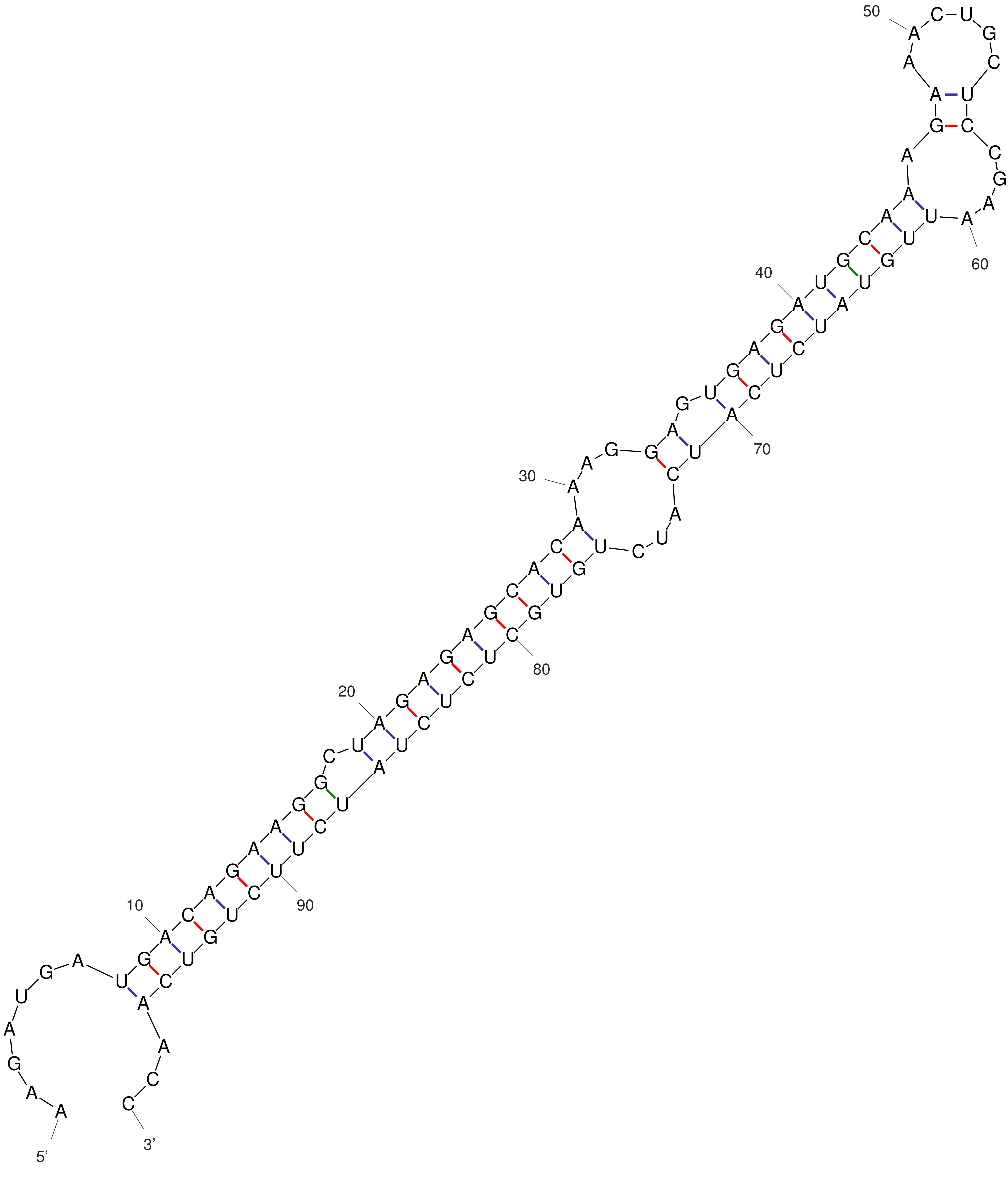

dG = -46.40 [Initially -46.40] Cs-miR156_21 NC_025700 1

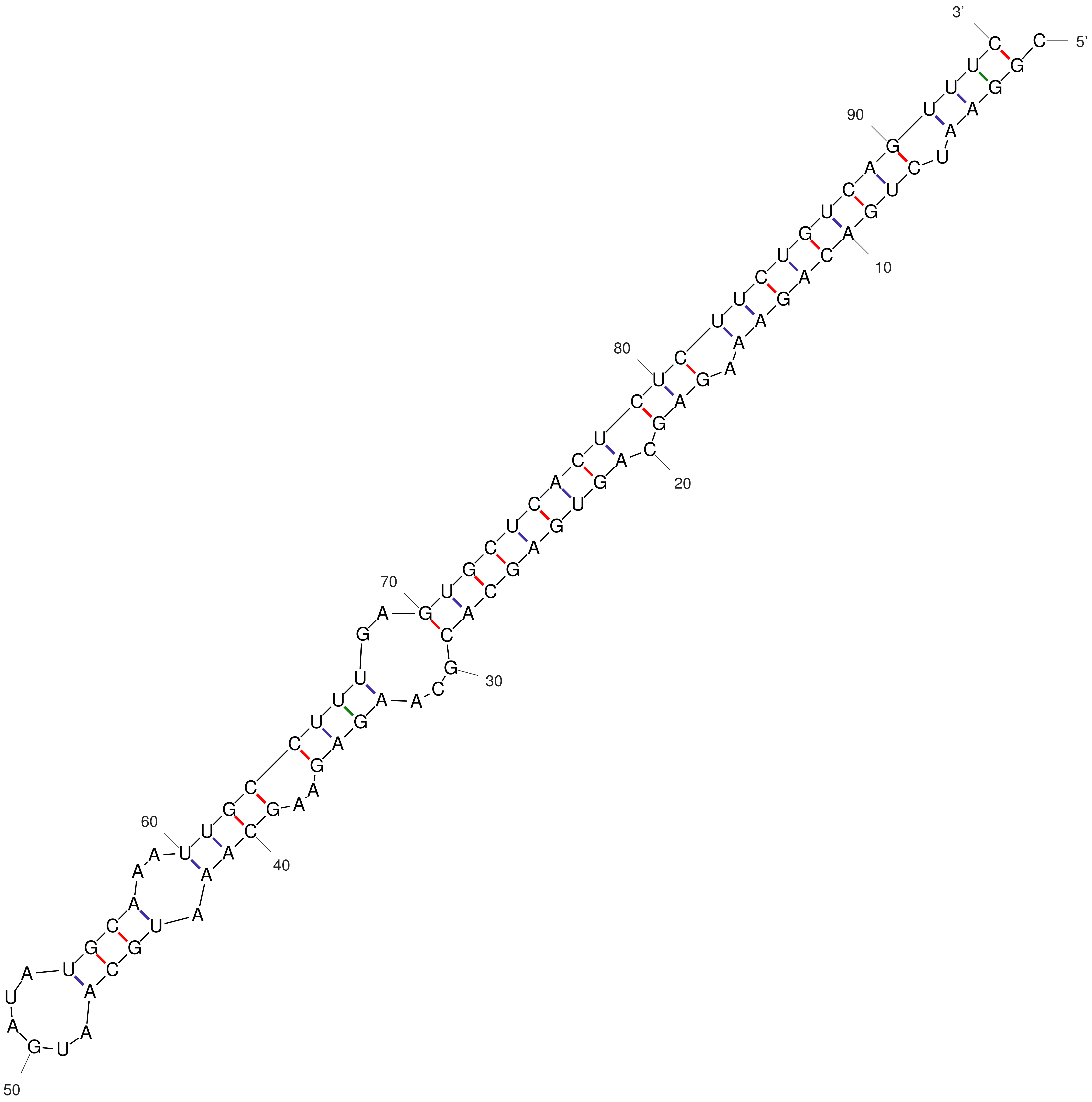

dG = -44.10 [Initially -44.10] Cs-miR156_22 NC_025700 1

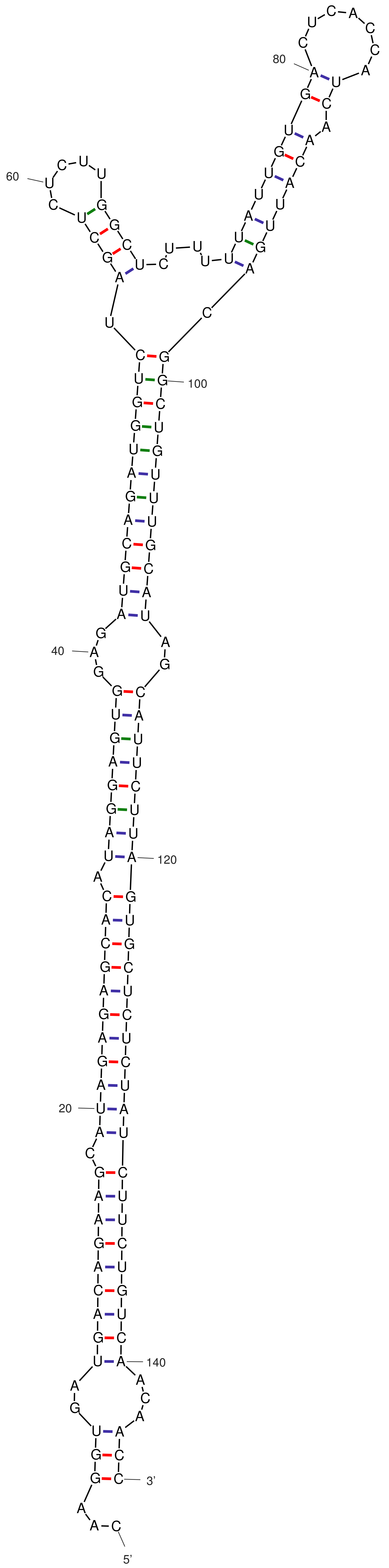

dG = -61.60 [Initially -62.60] Cs-miR156_23 NC_025701 1

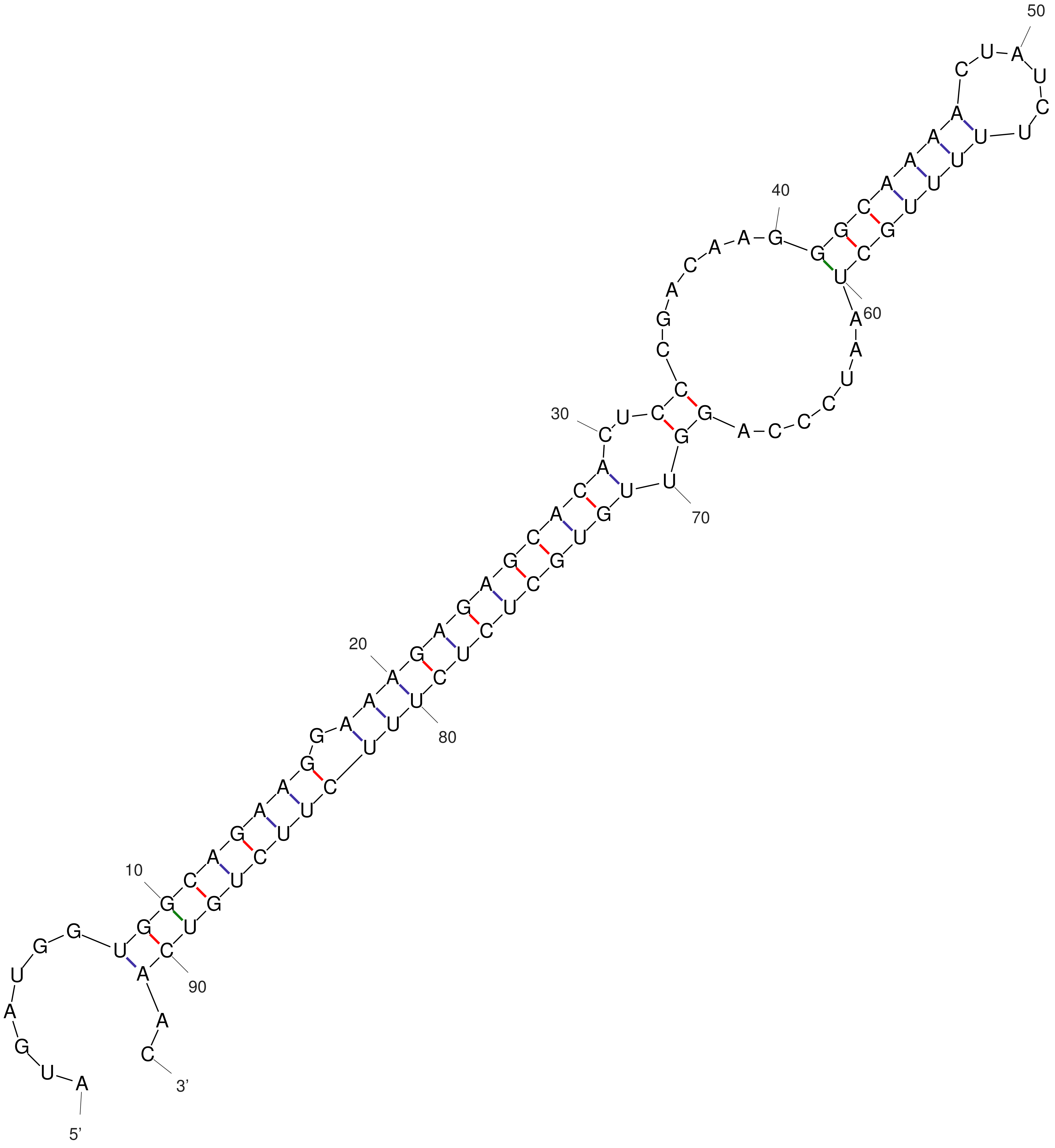

dG = -41.30 [Initially -41.30] Cs-miR156_24 NC_025702 1

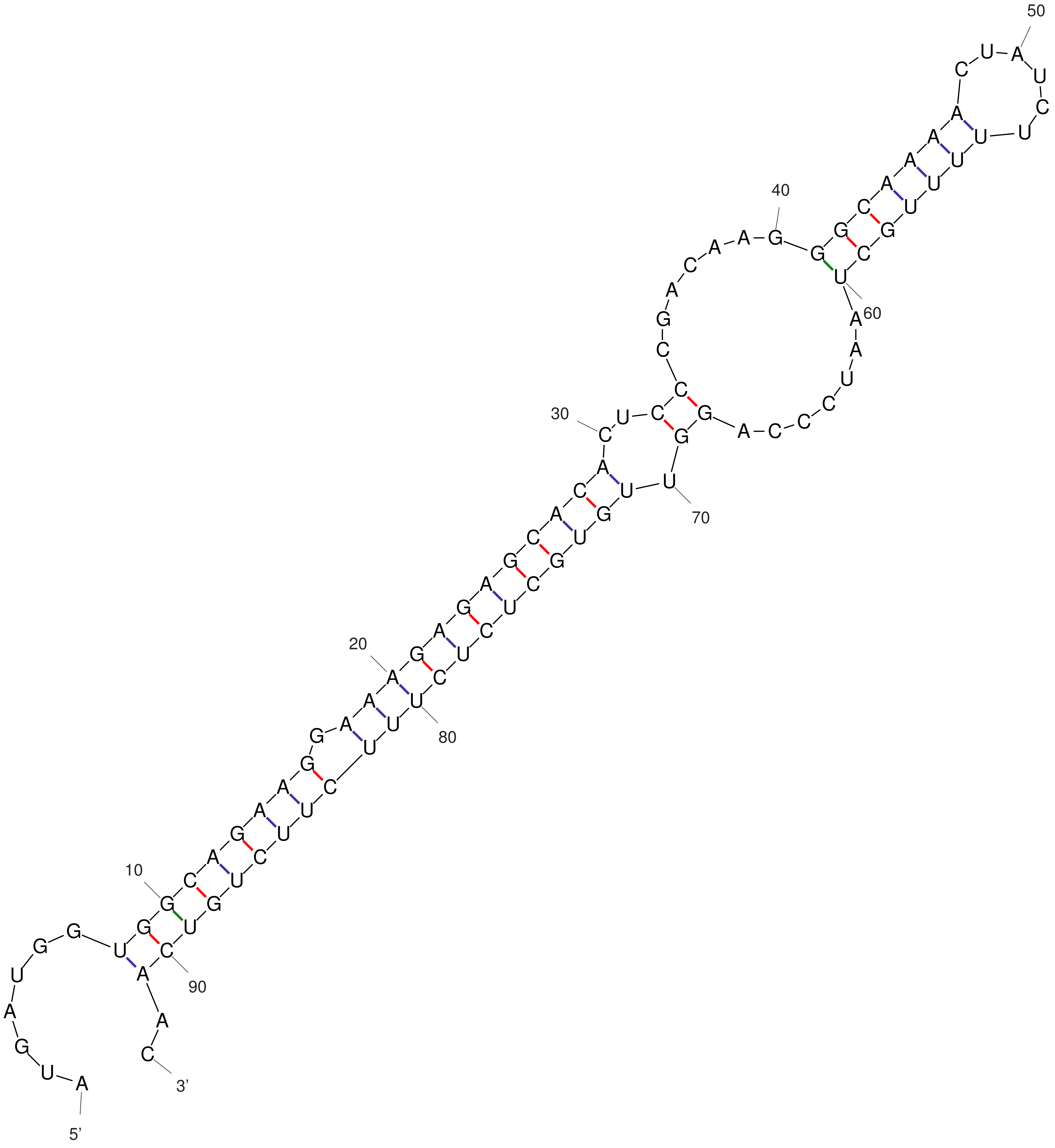

dG = -41.30 [Initially -41.30] Cs-miR156_25 NC_025702 1

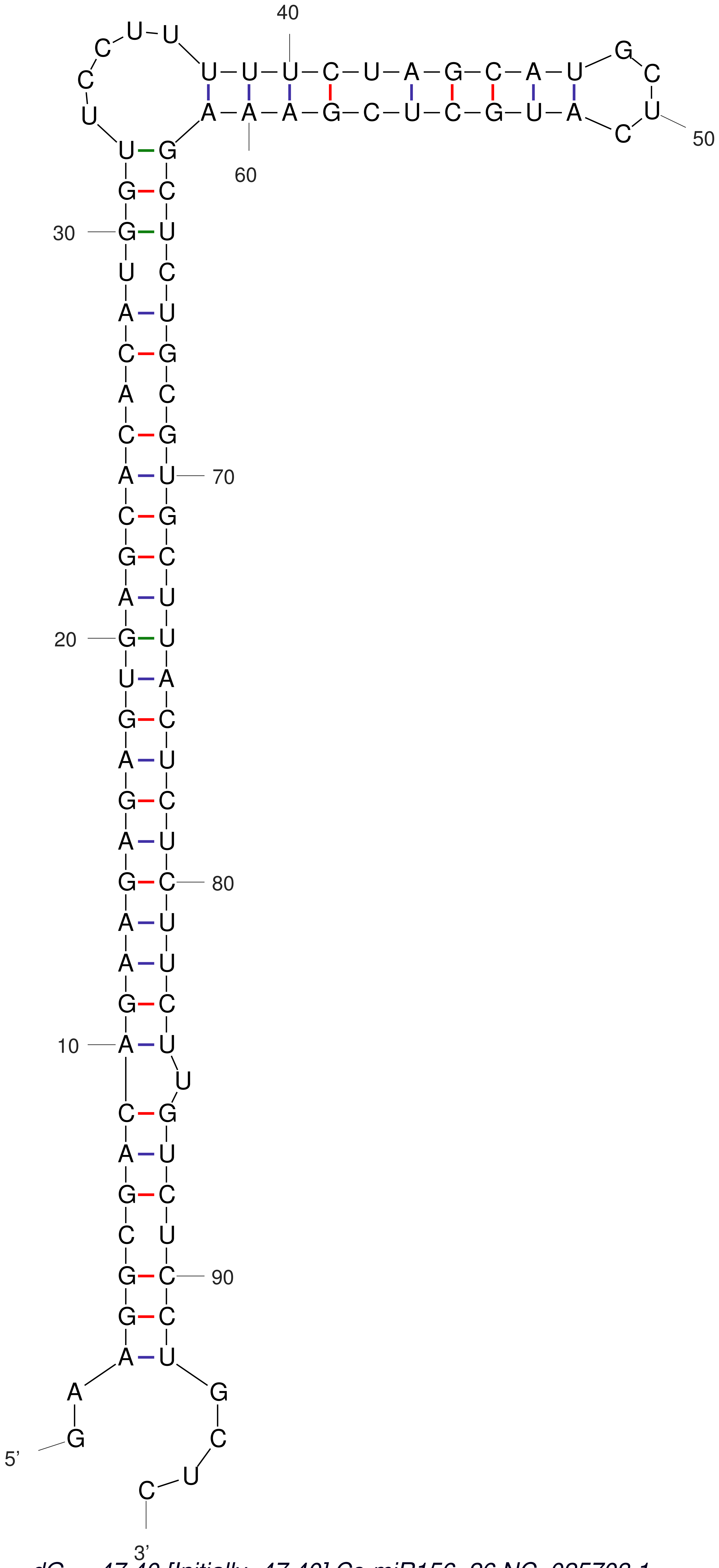

dG = -47.40 [Initially -47.40] Cs-miR156_26 NC_025703 1

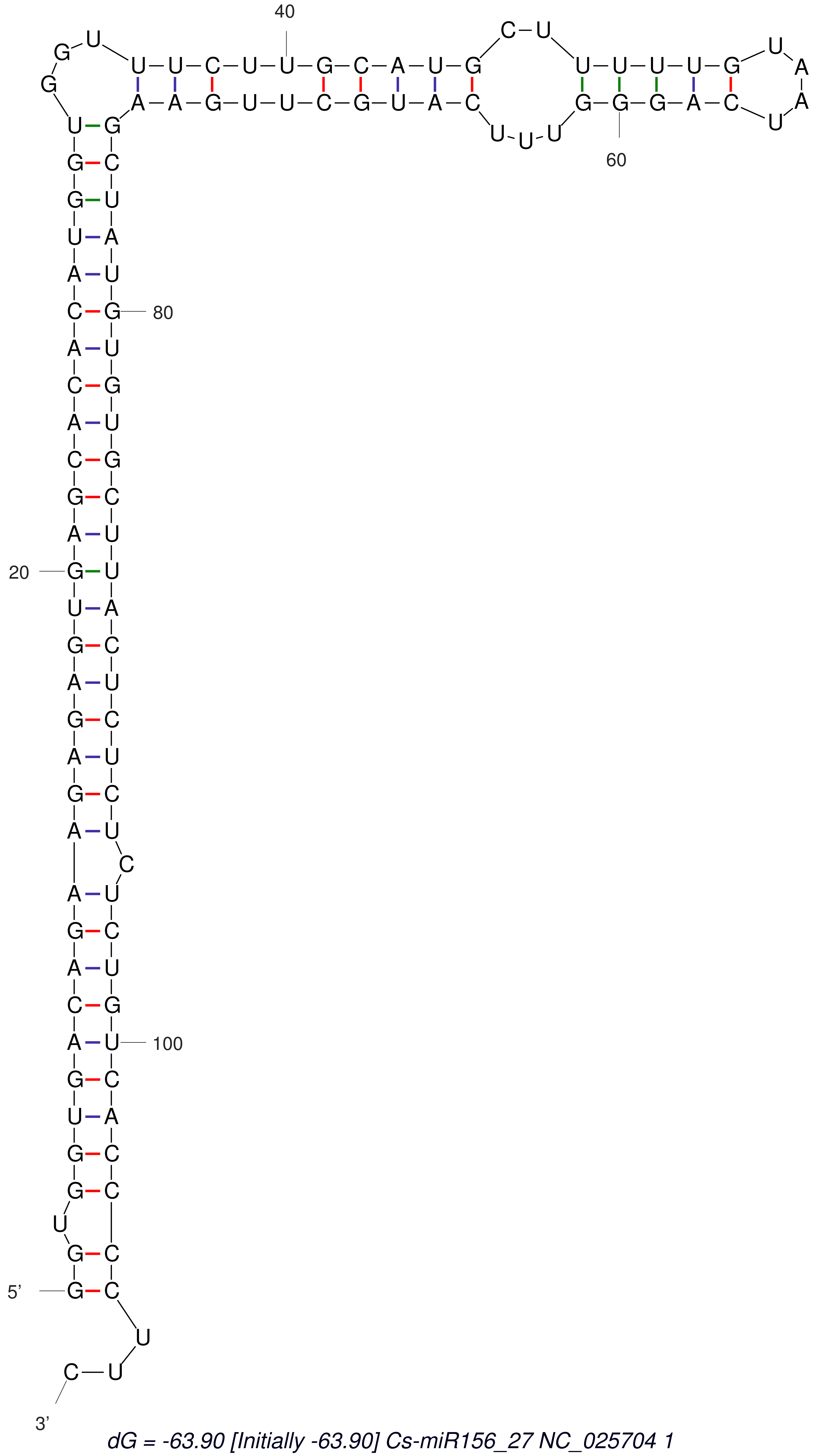

dG = -63.90 [Initially -63.90] Cs-miR156_27 NC_025704 1

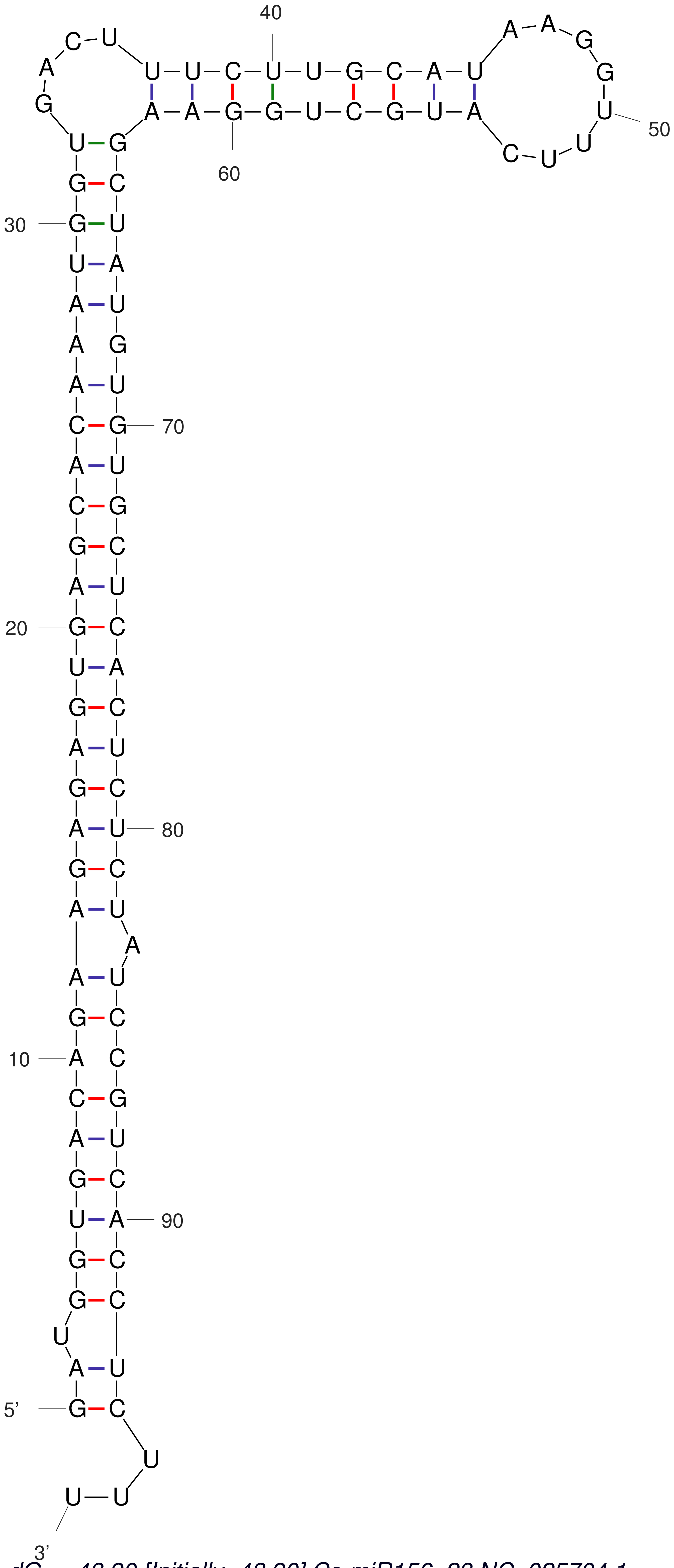

dG = -48.90 [Initially -48.90] Cs-miR156_28 NC_025704 1

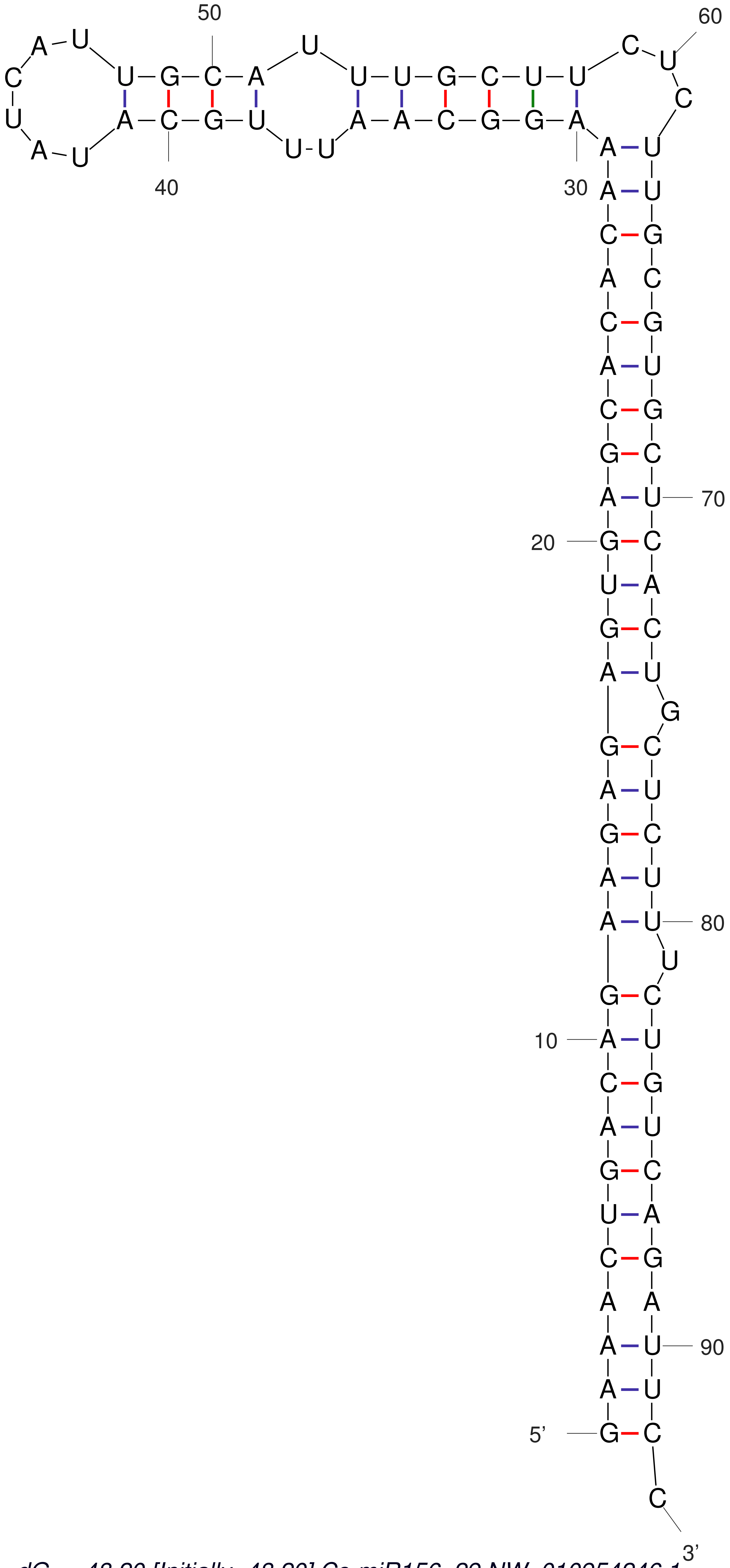

dG = -48.20 [Initially -48.20] Cs-miR156_29 NW_010954246 1

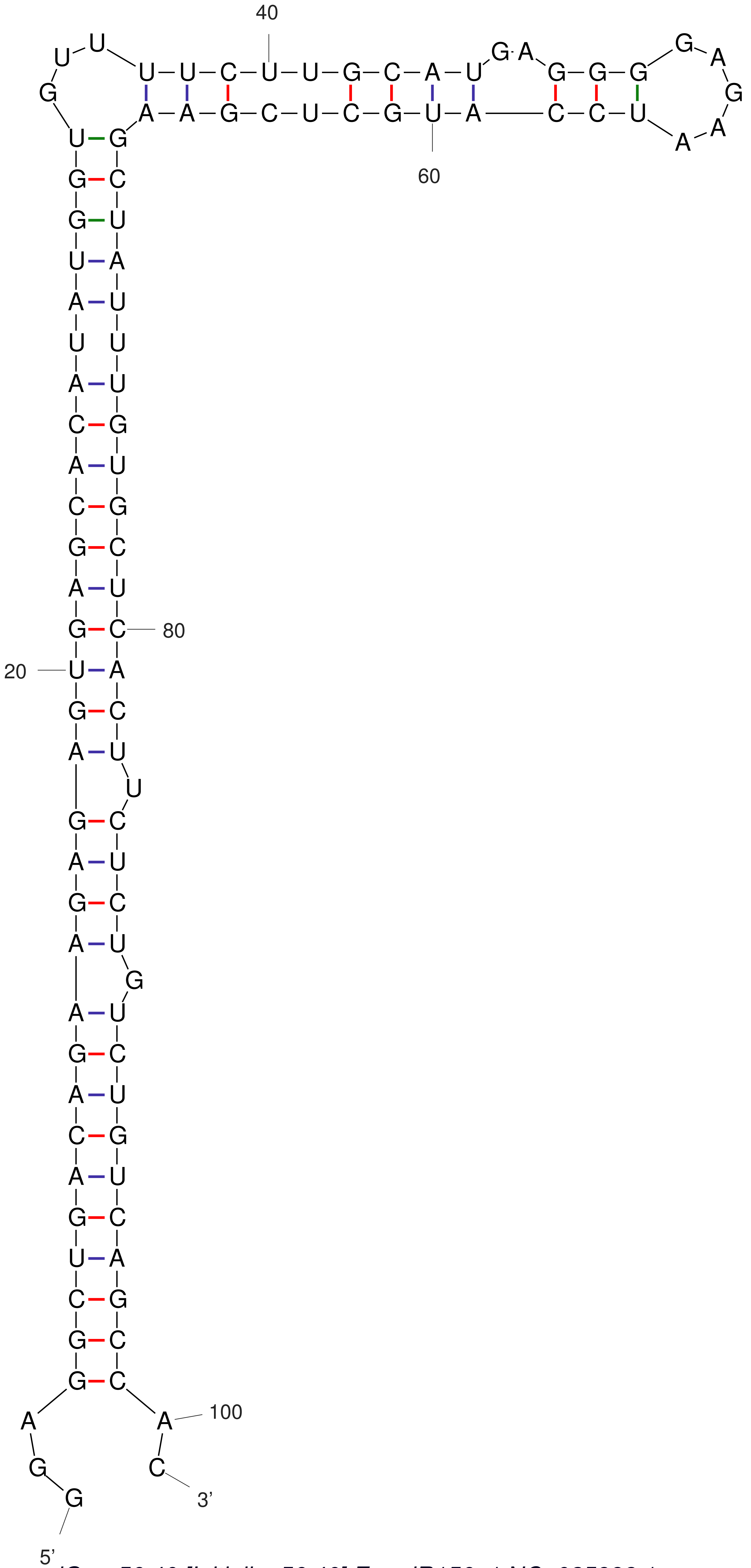

dG = -56.40 [Initially -56.40] Eg-miR156_1 NC_025993 1

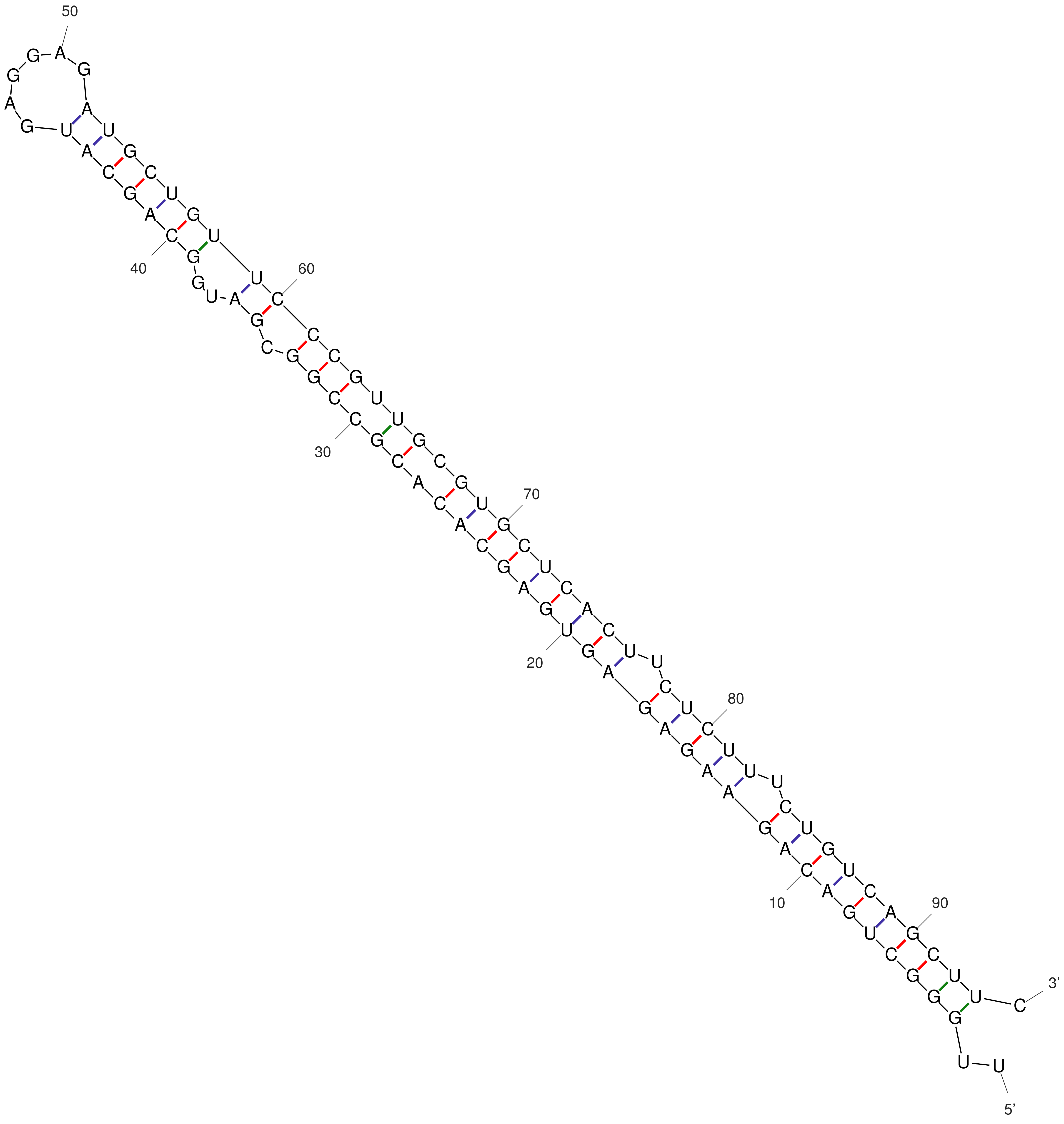

dG = -55.20 [Initially -55.20] Eg-miR156_2 NC_025994 1

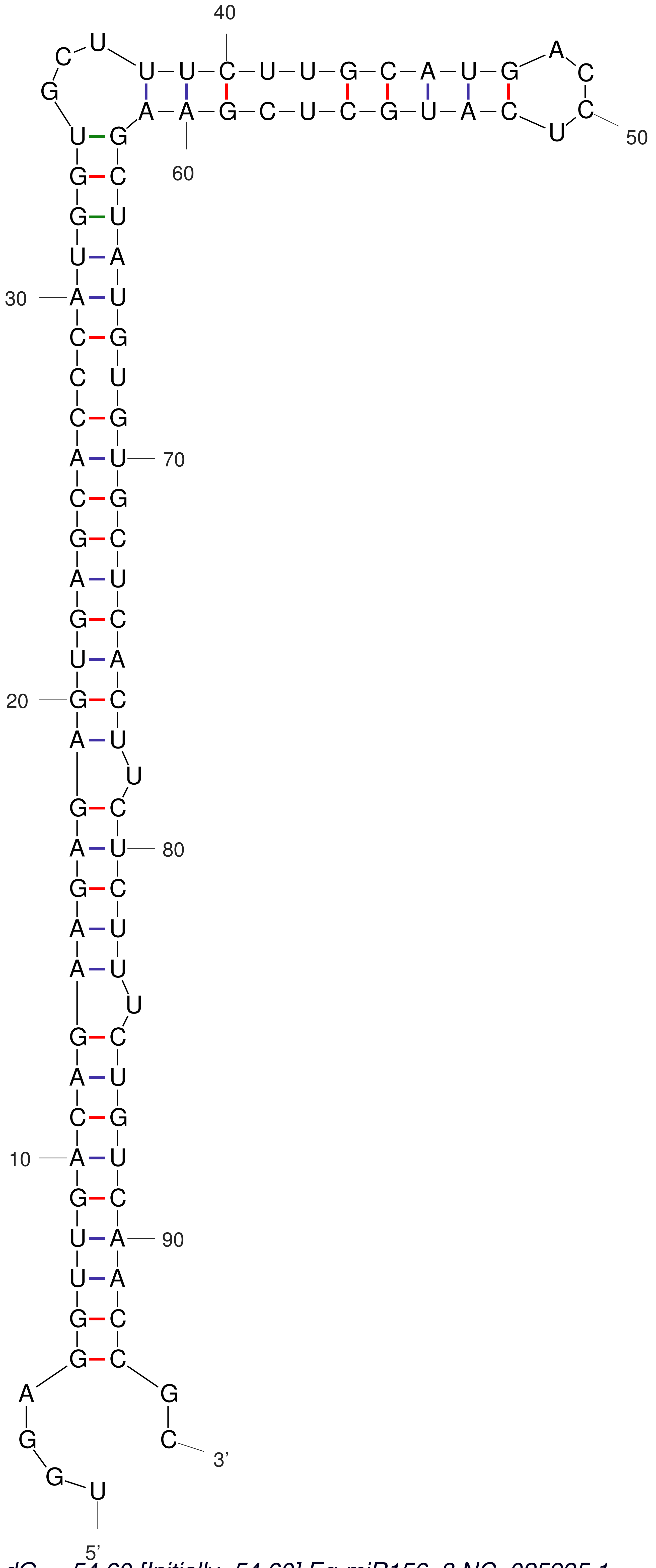

dG = -54.60 [Initially -54.60] Eg-miR156_3 NC_025995 1

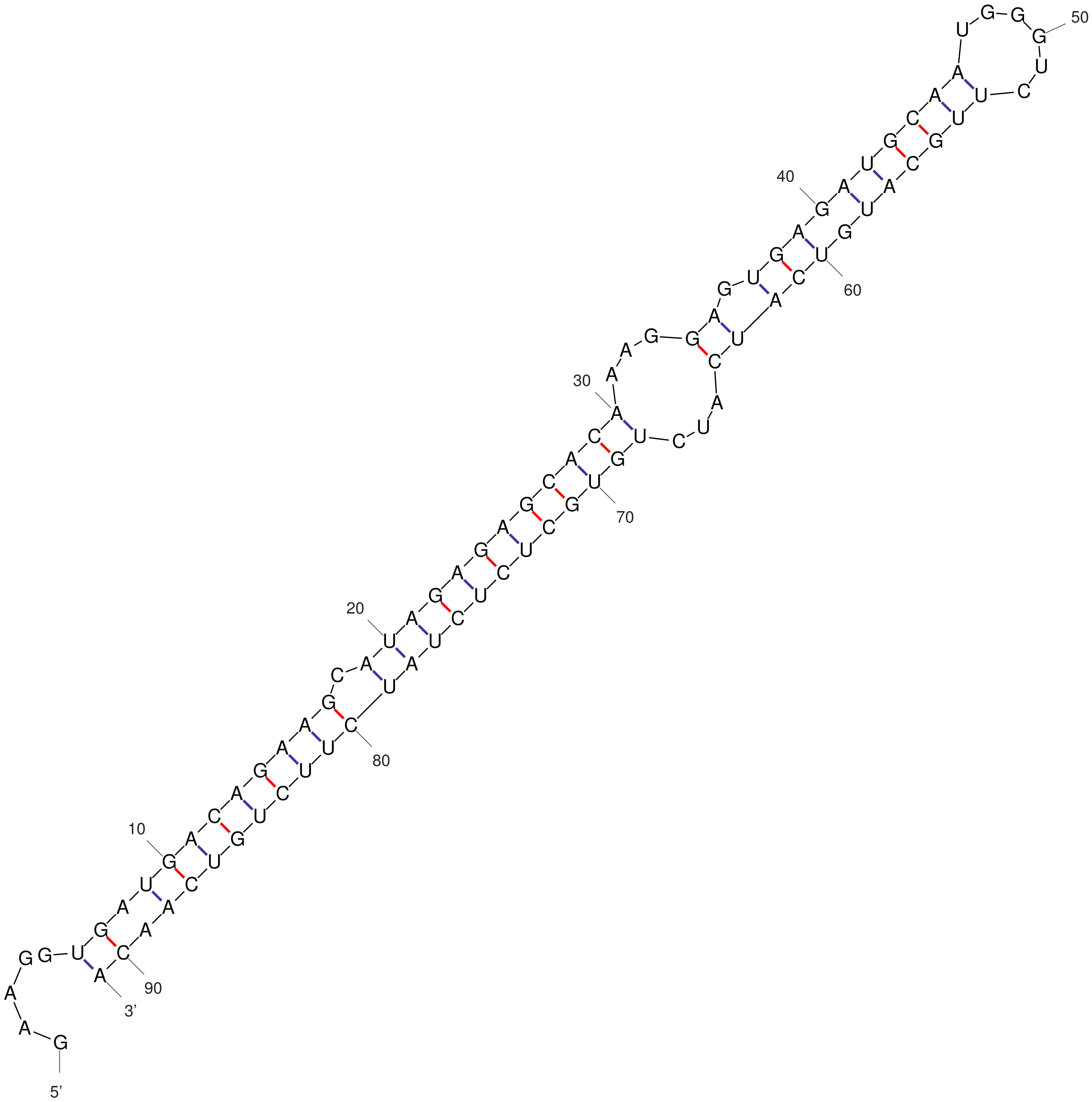

dG = -46.20 [Initially -46.40] Eg-miR156_4 NC_025998 1

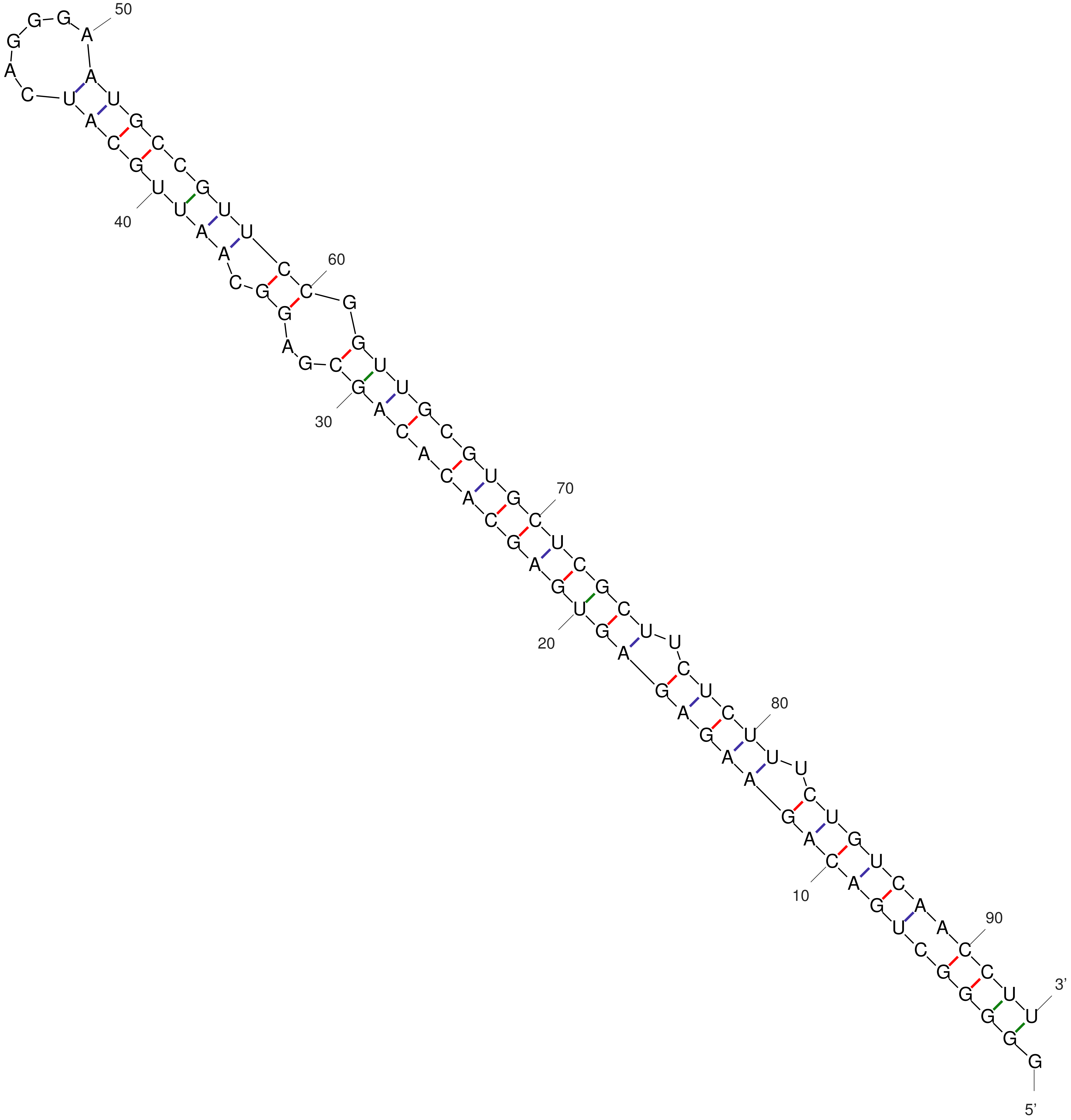

dG = -47.80 [Initially -47.80] Eg-miR156_5 NC_026000 1

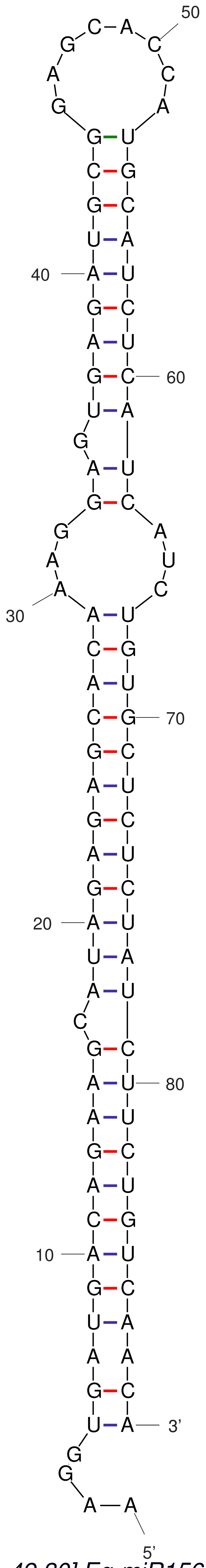

dG = -49.30 [Initially -49.30] Eg-miR156_6 NC_026005 1

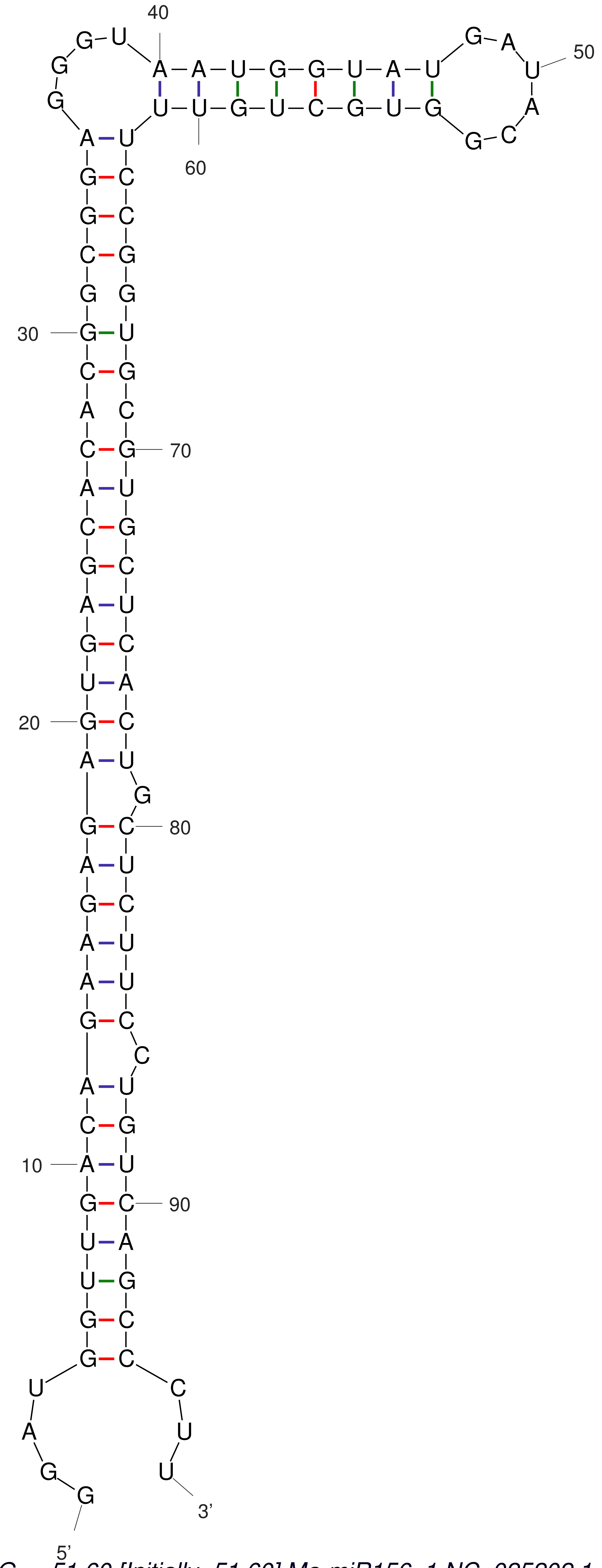

dG = -51.60 [Initially -51.60] Ma-miR156_1 NC_025202 1

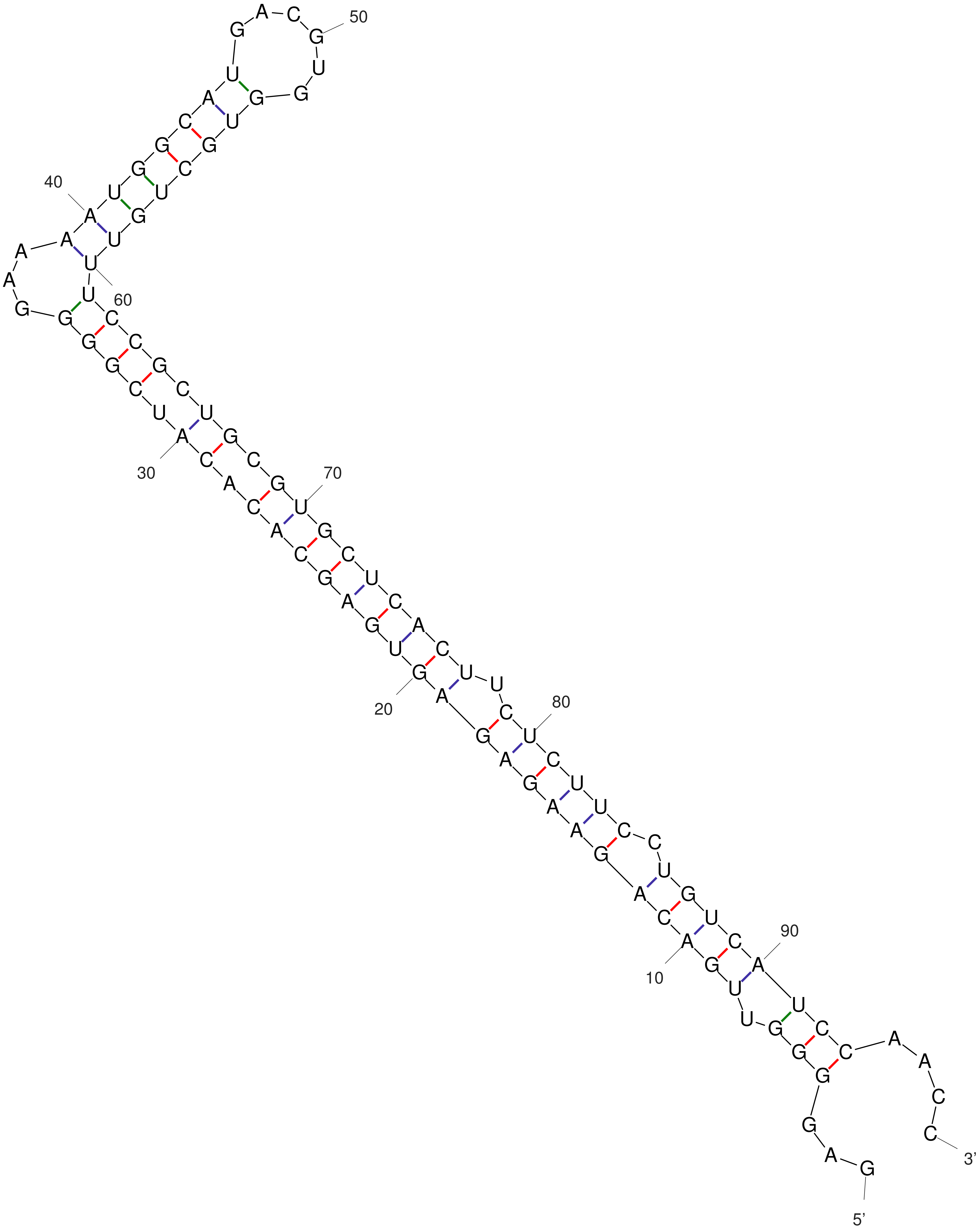

dG = -48.60 [Initially -48.60] Ma-miR156_2 NC_025202 1

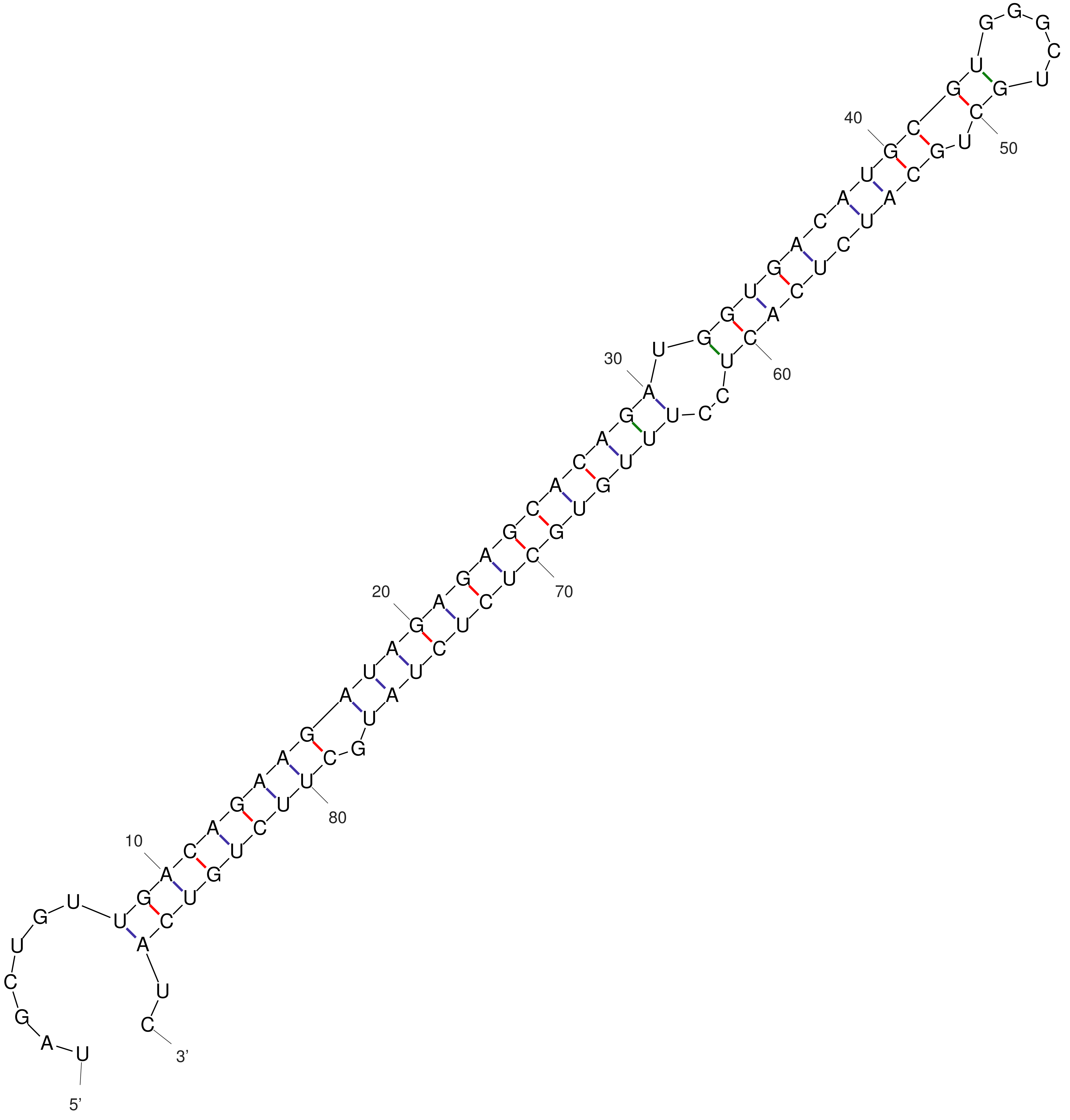

dG = -46.60 [Initially -46.60] Ma-miR156_3 NC_025202 1

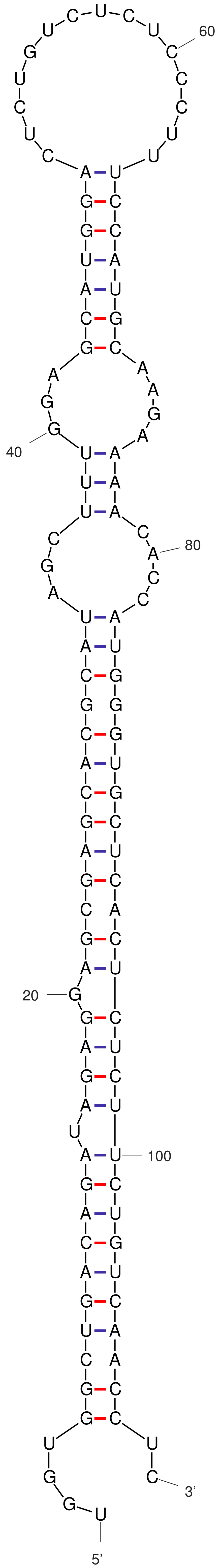

dG = -41.70 [Initially -41.70] Ma-miR156_4 NC_025203 1

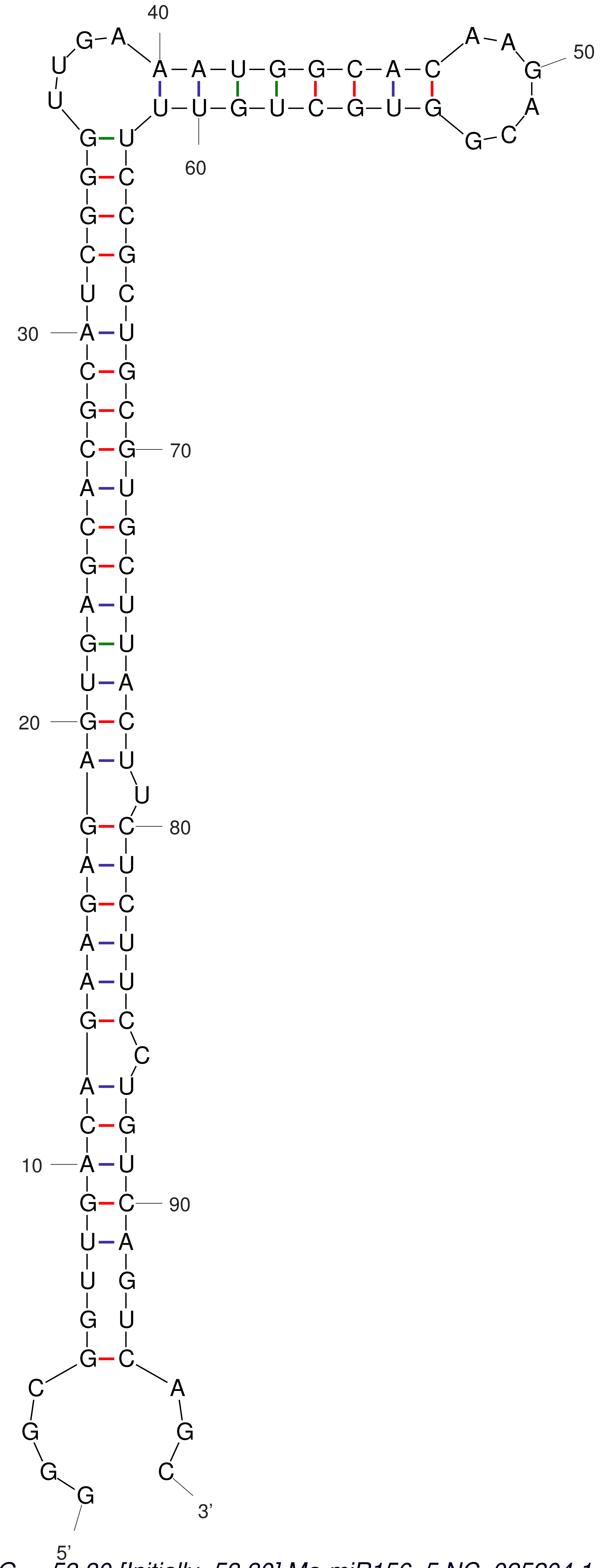

dG = -53.30 [Initially -53.30] Ma-miR156_5 NC_025204 1

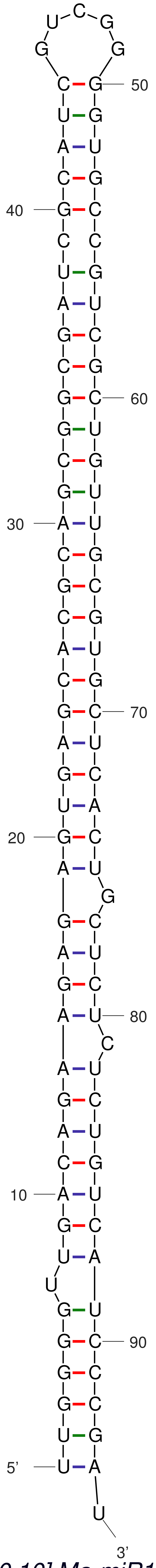

dG = -70.10 [Initially -70.10] Ma-miR156_6 NC_025204 1

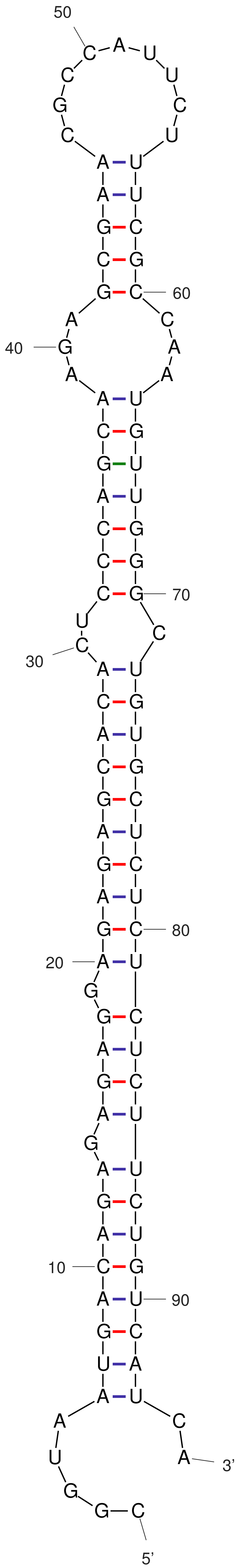

dG = -48.30 [Initially -48.60] Ma-miR156_7 NC_025204 1

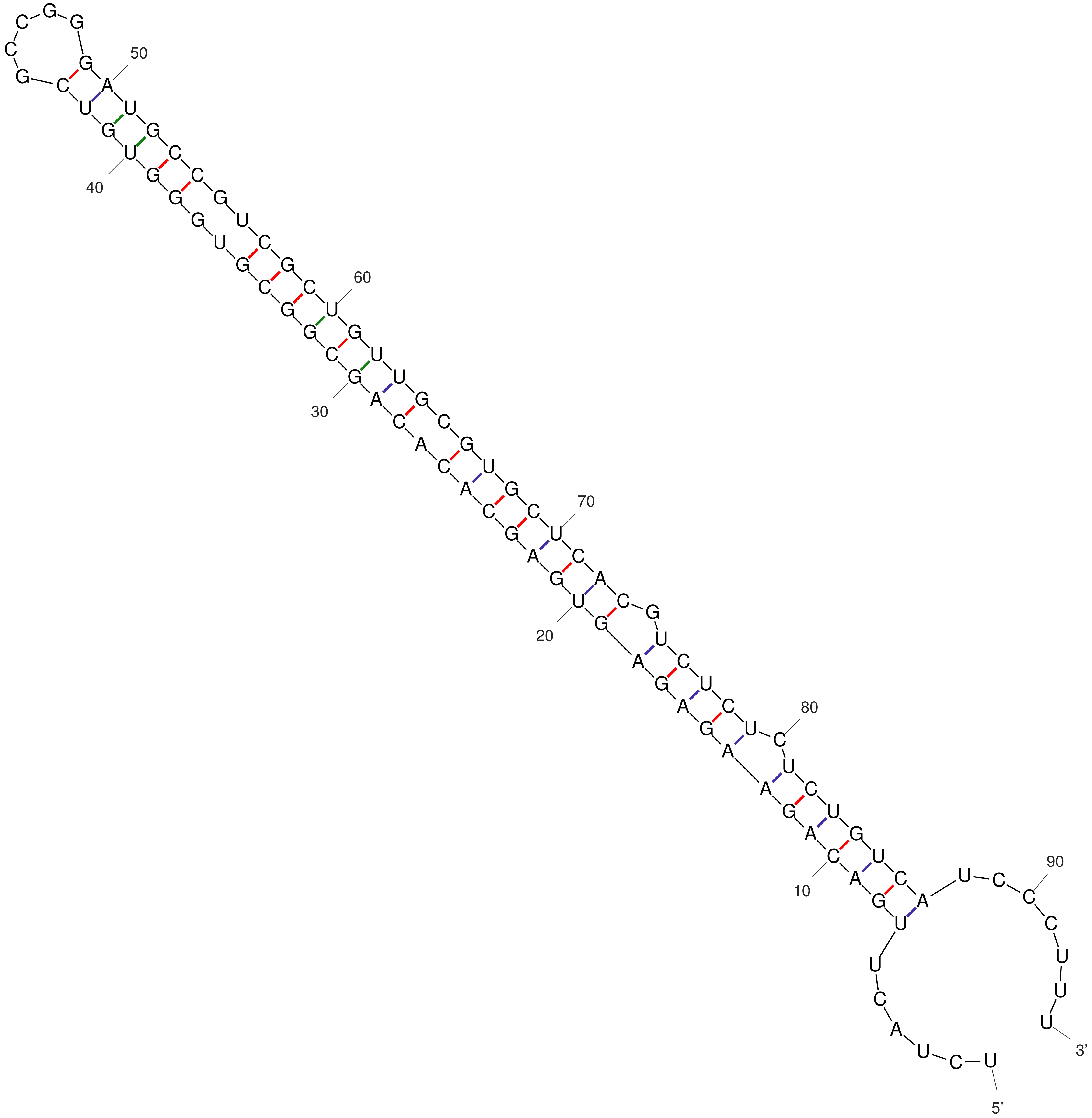

dG = -53.60 [Initially -53.60] Ma-miR156_8 NC_025205 1

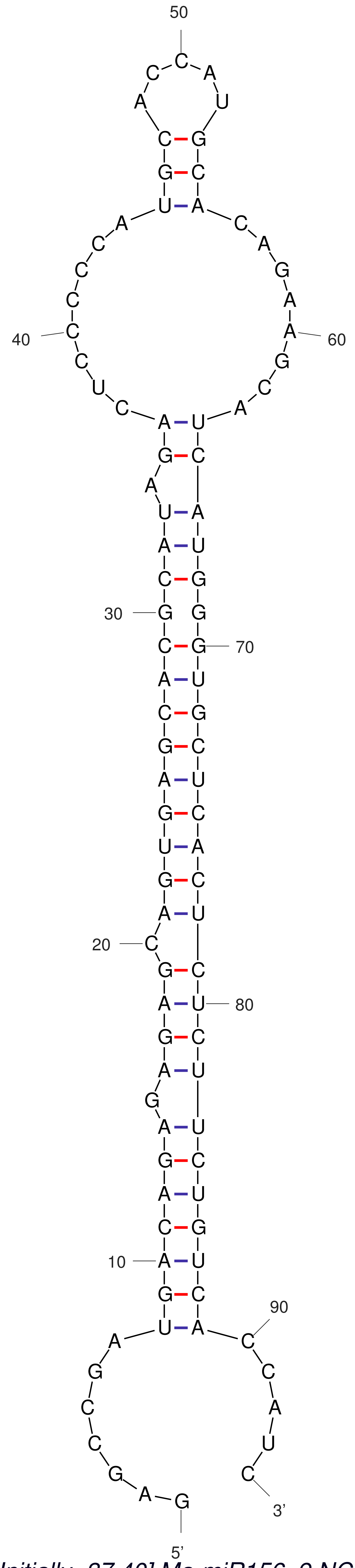

dG = -37.40 [Initially -37.40] Ma-miR156_9 NC_025205 1

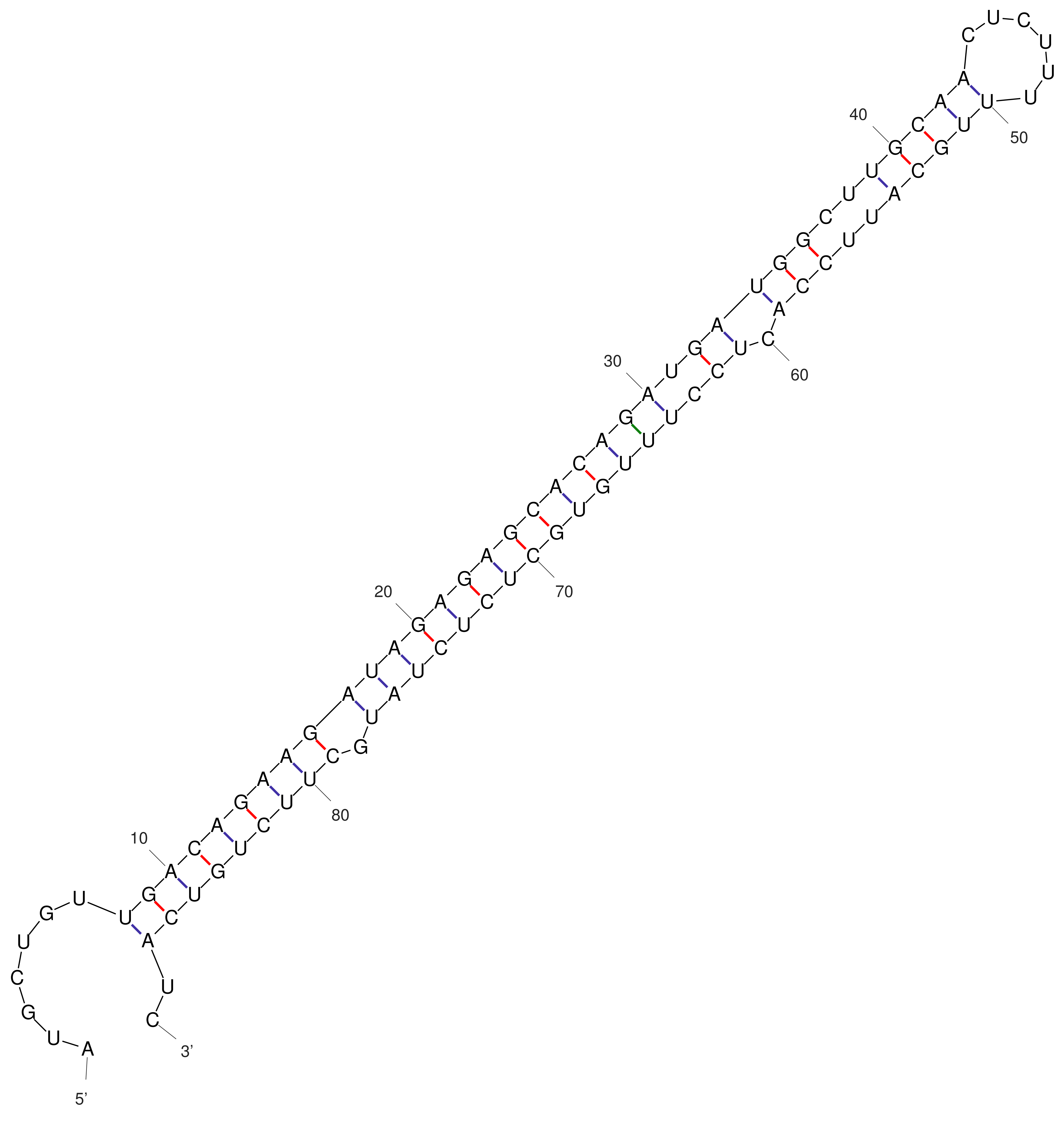

dG = -46.90 [Initially -46.90] Ma-miR156_10 NC_025205 1

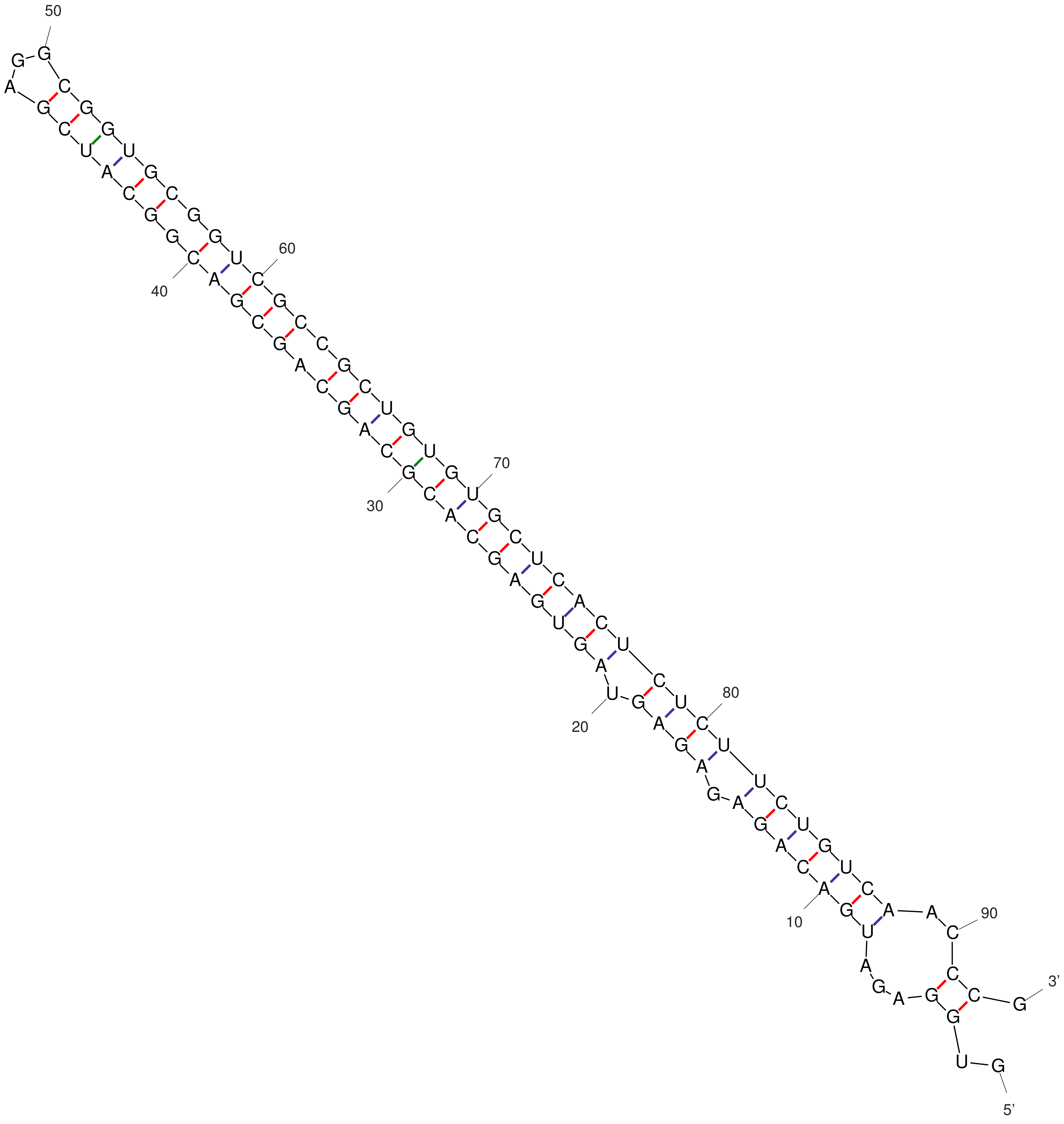

dG = -64.20 [Initially -64.20] Ma-miR156_11 NC_025206 1

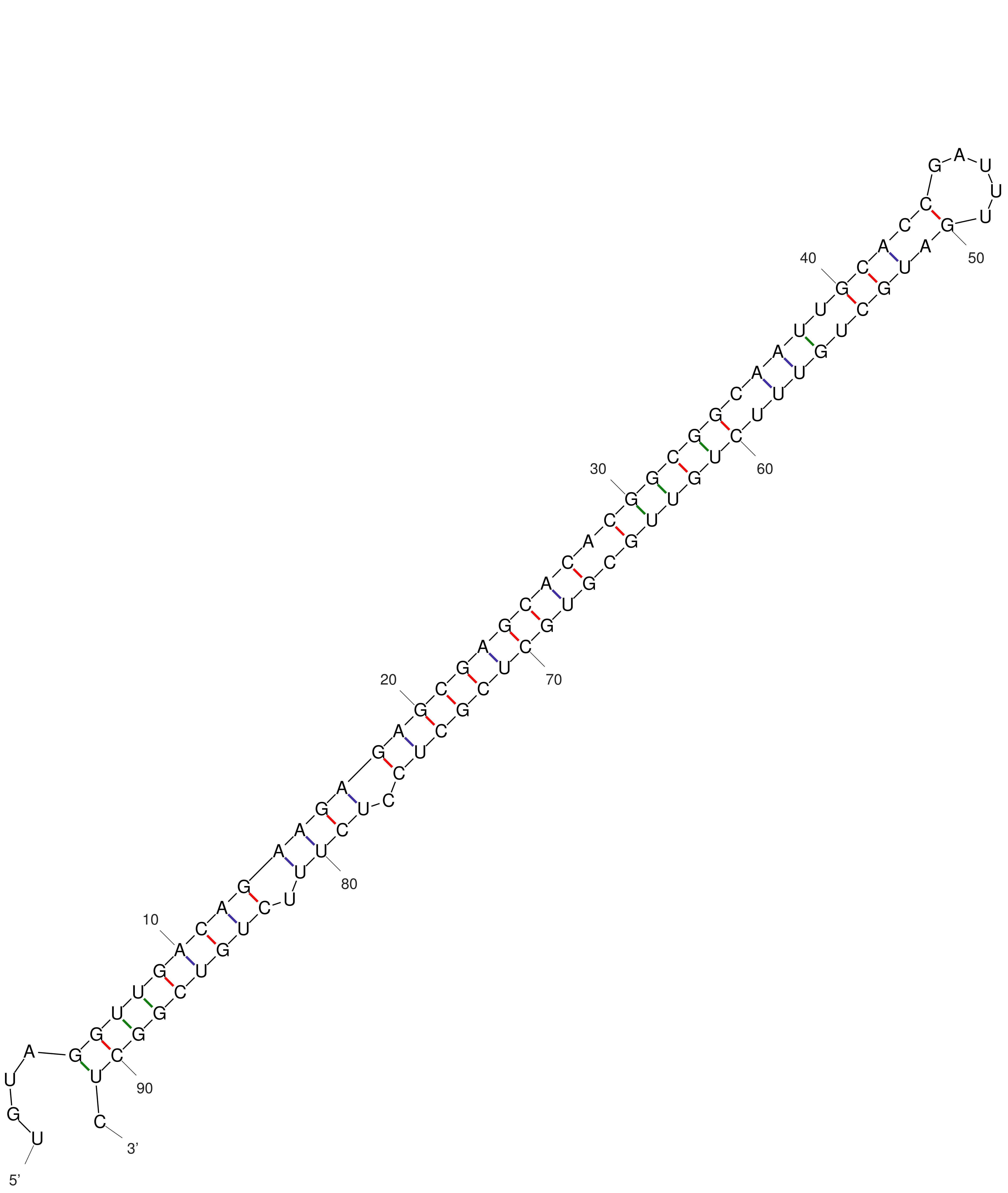

dG = -48.30 [Initially -48.30] Ma-miR156_12 NC_025207 1

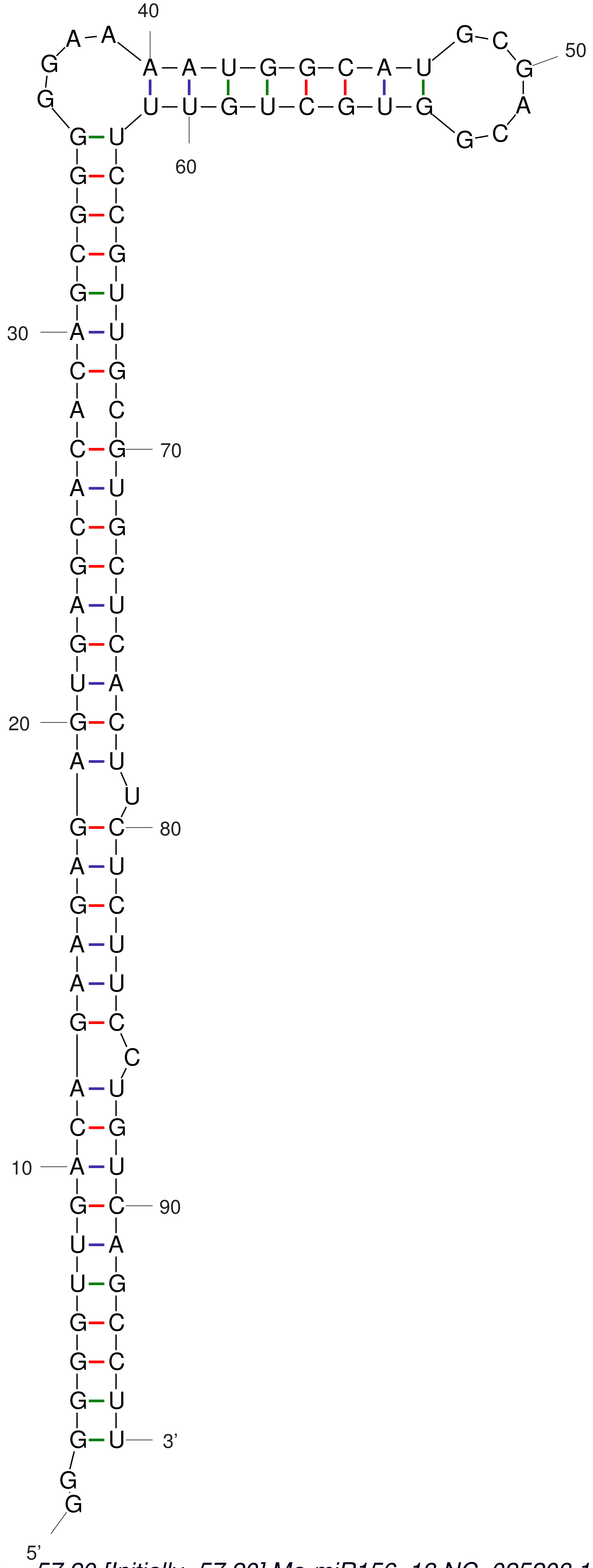

dG = -57.20 [Initially -57.20] Ma-miR156_13 NC_025208 1

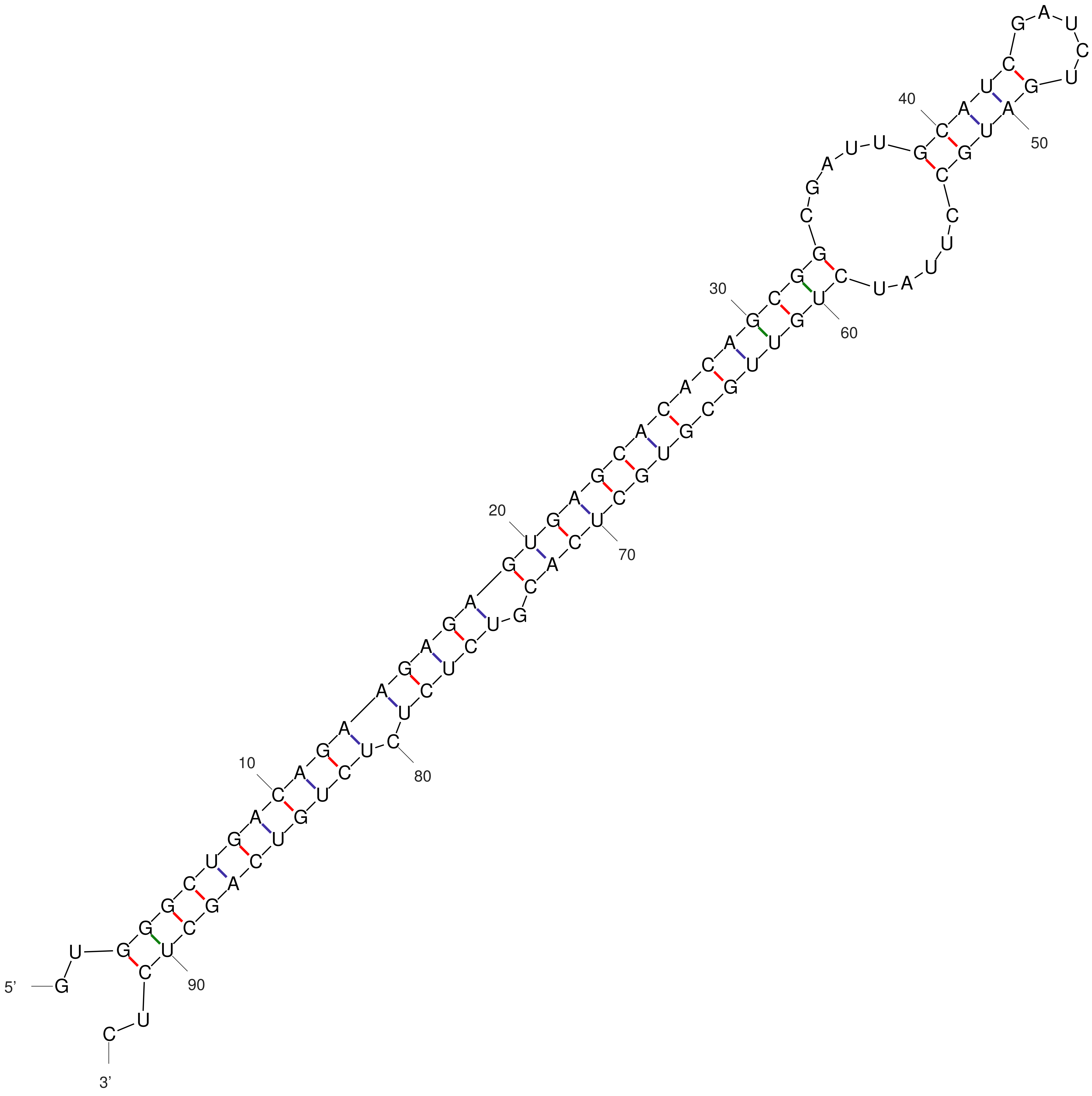

dG = -55.60 [Initially -55.60] Ma-miR156_14 NC_025208 1

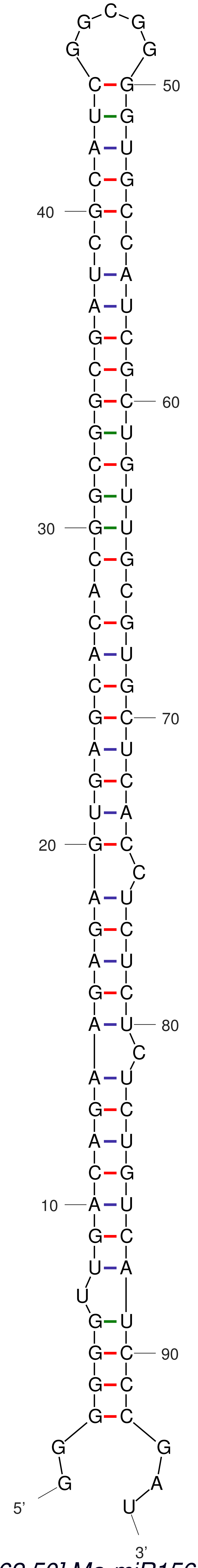

dG = -62.50 [Initially -62.50] Ma-miR156_15 NC_025210 1

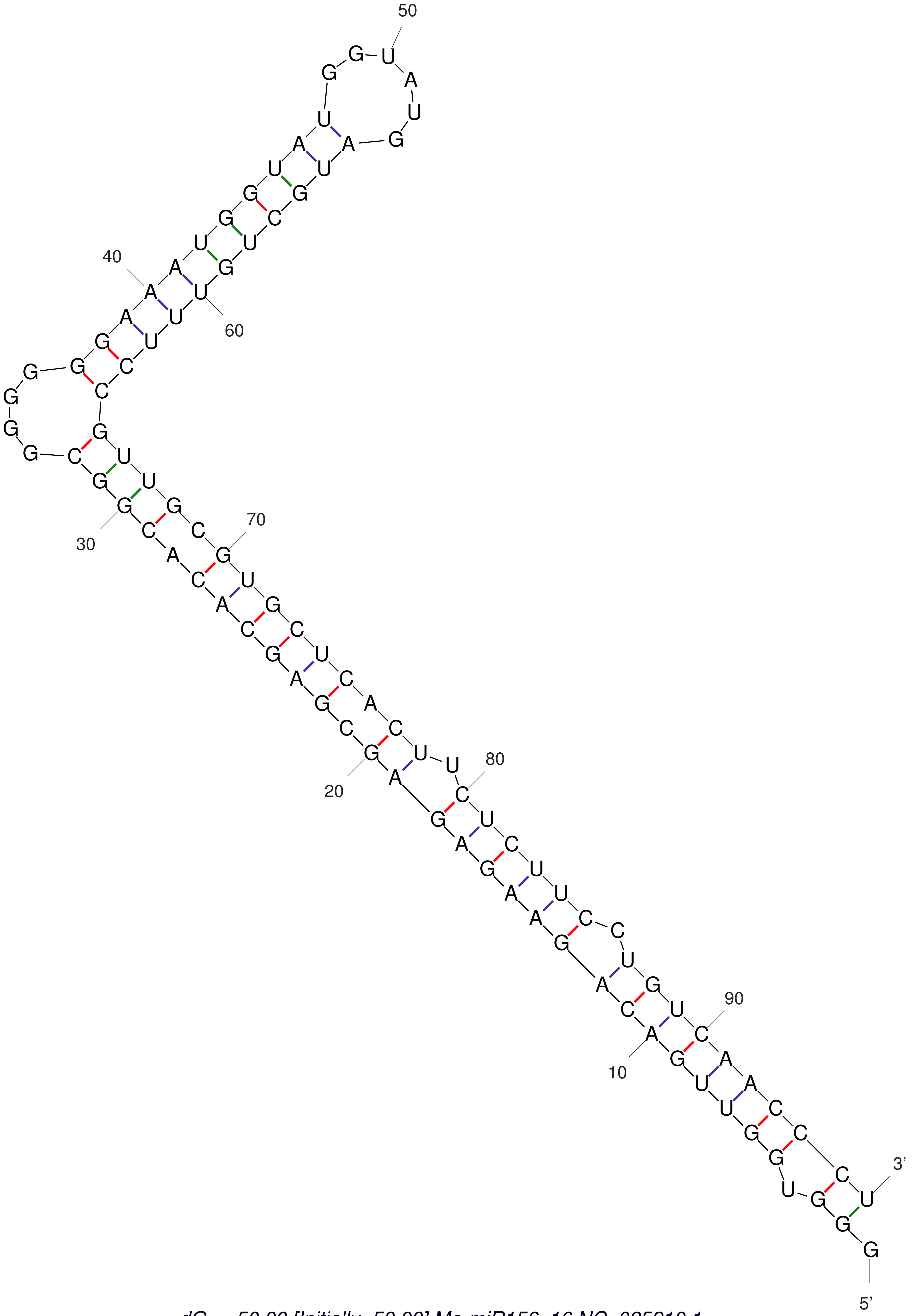

dG = -50.00 [Initially -50.00] Ma-miR156_16 NC_025210 1

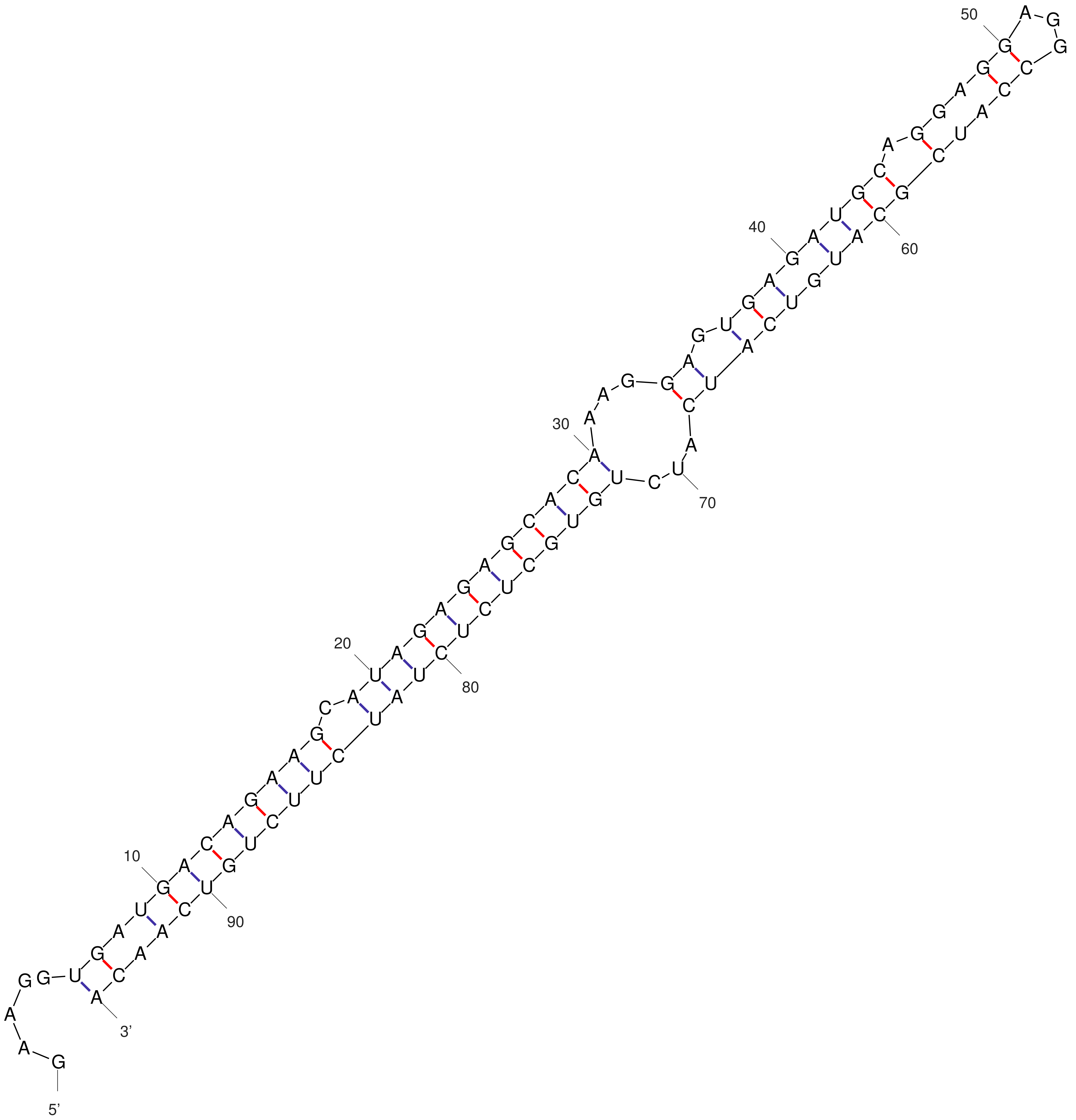

dG = -45.00 [Initially -45.20] Ma-miR156_17 NC_025210 1

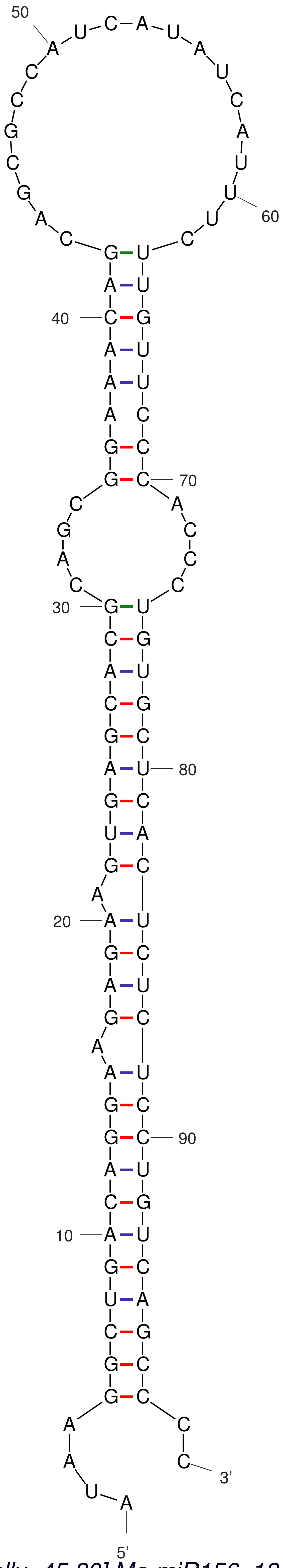

dG = -45.80 [Initially -45.80] Ma-miR156_18 NC_025210 1

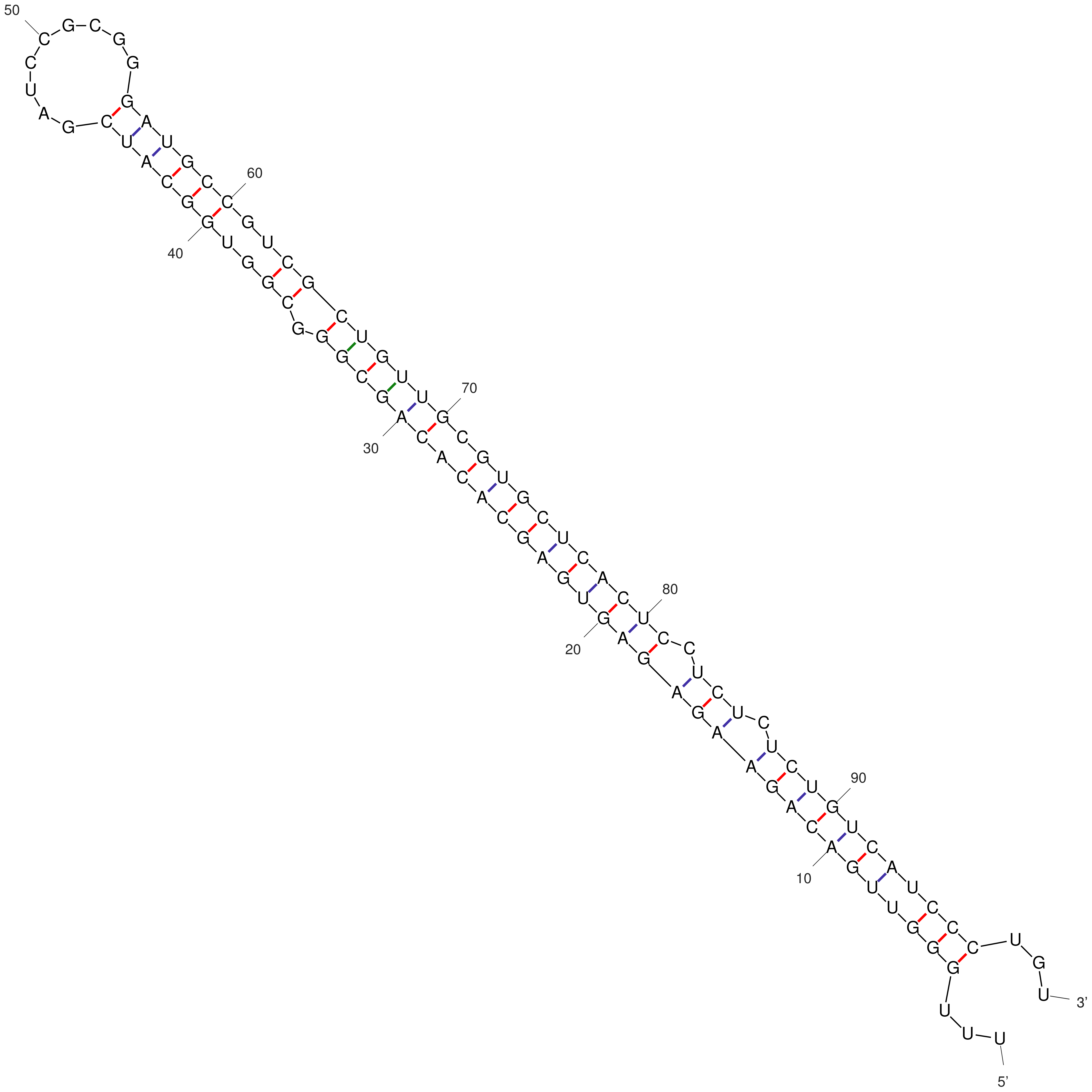

dG = -62.00 [Initially -62.00] Ma-miR156_19 NC_025211 1

Output of sir_graph (©)
mfold_util 4.7

Created Mon Jul 22 08:01:00 2019

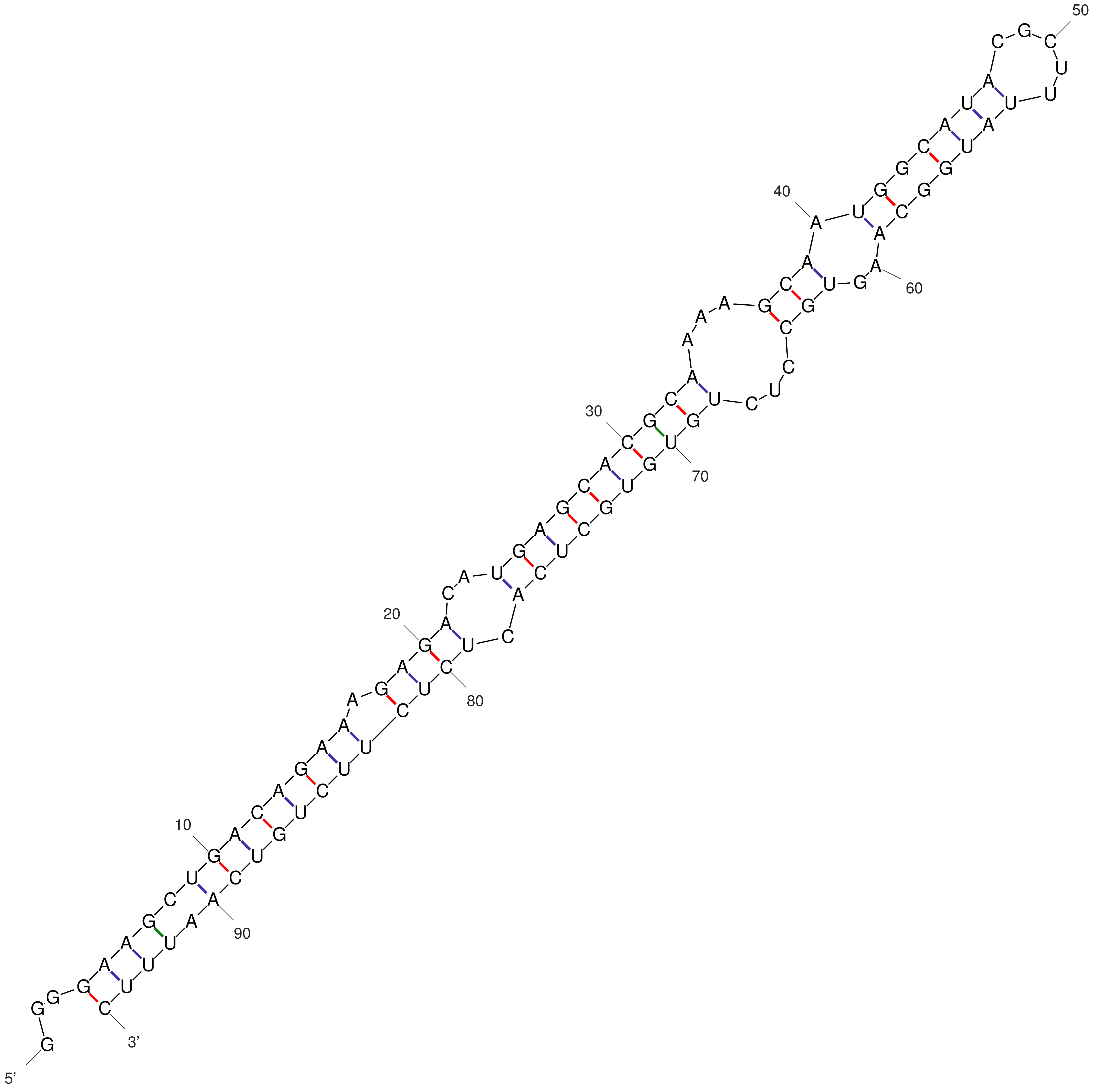

dG = -41.20 [Initially -41.20] 19Jul22-08-00-59

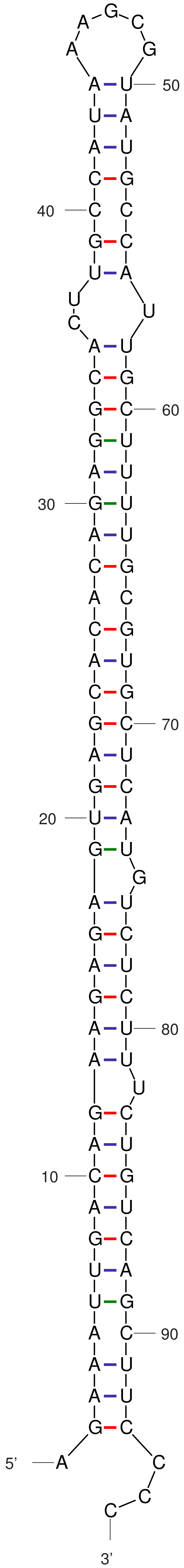

dG = -45.80 [Initially -45.80] 19Jul22-08-08-27

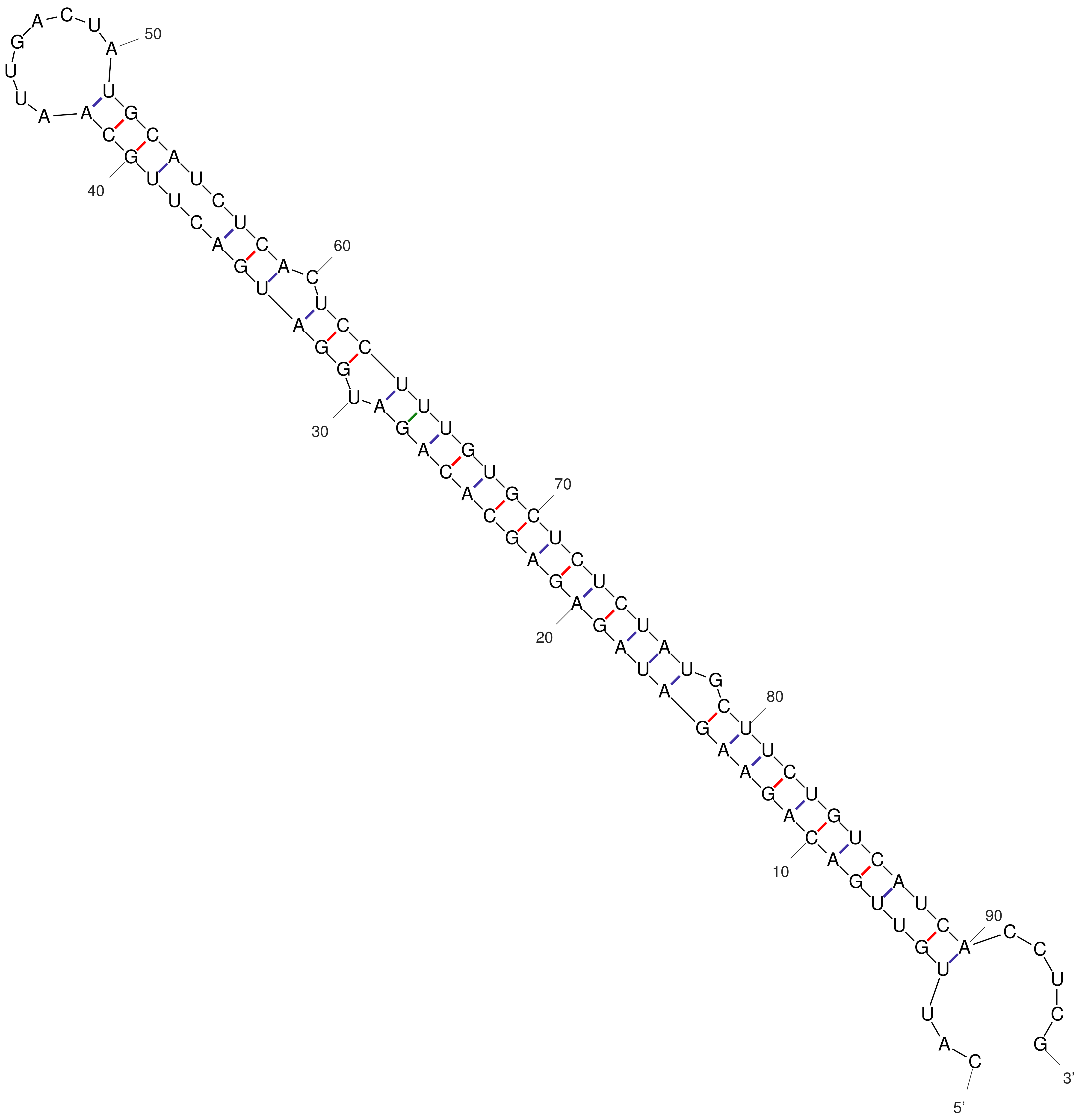

dG = -48.10 [Initially -48.10] md-miR156_3 NC_024244 1

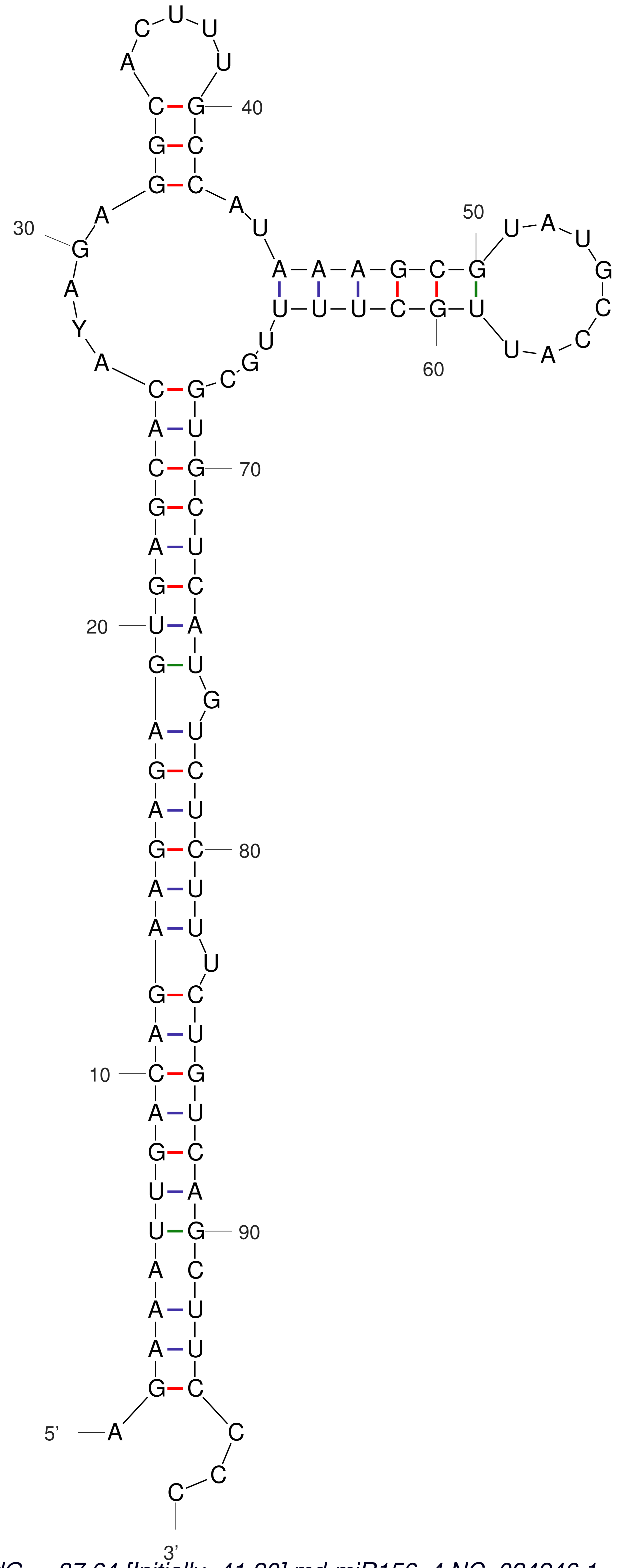

dG = -37.64 [Initially -41.30] md-miR156_4 NC_024246 1

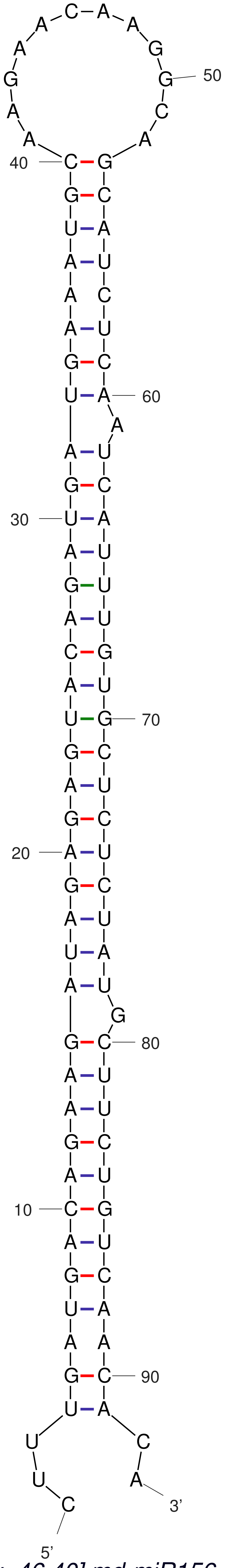

dG = -46.40 [Initially -46.40] md-miR156_5 NC_024247 1

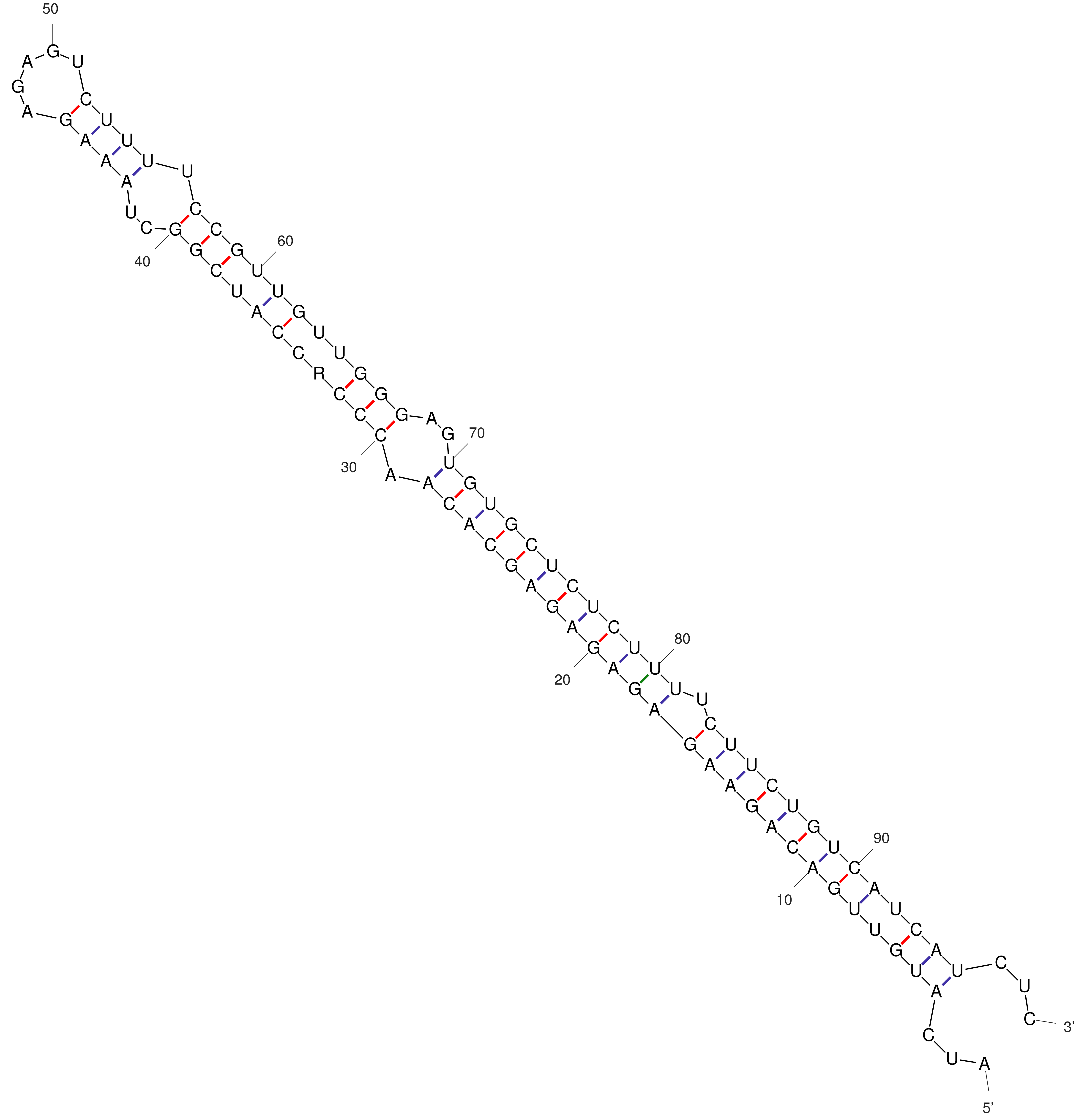

dG = -49.10 [Initially -49.10] md-miR156_6 NC_024248 1

Output of sir_graph (©)
mfold_util 4.7

Created Mon Jul 22 08:12:31 2019

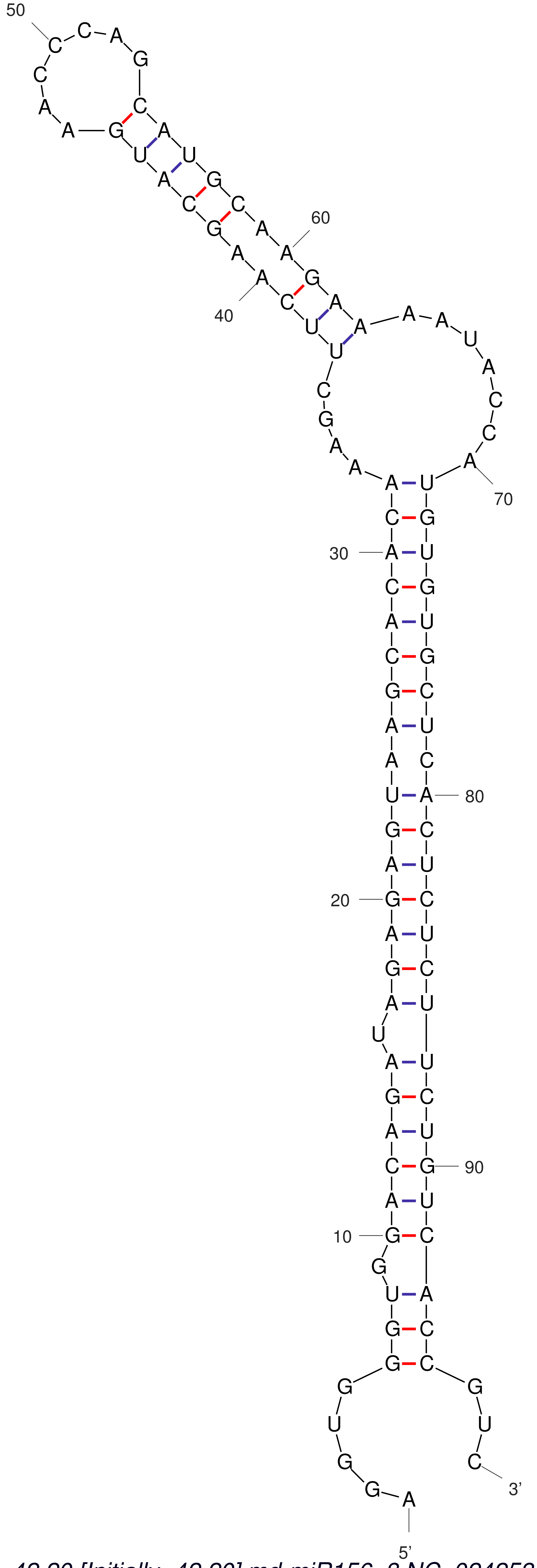

dG = -42.20 [Initially -42.20] md-miR156_8 NC_024253 1

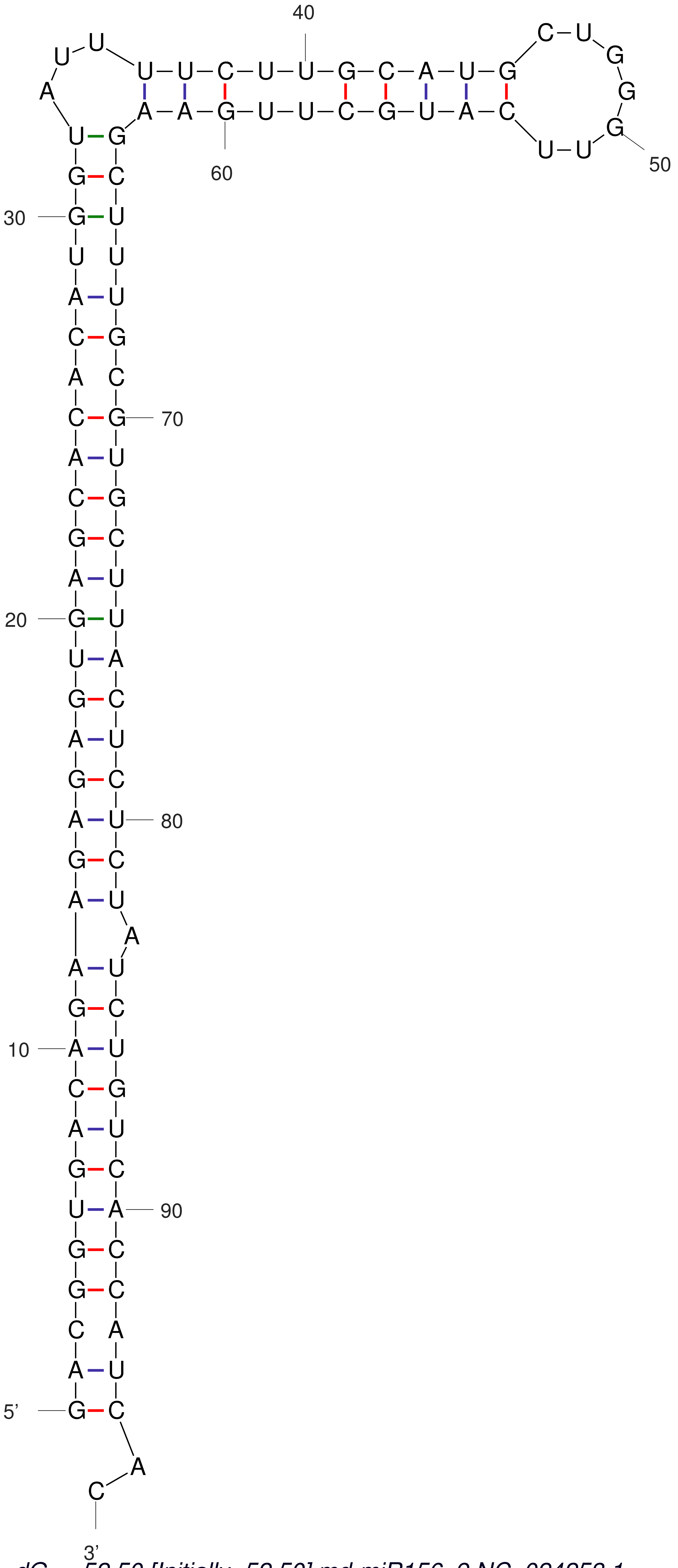

dG = -52.50 [Initially -52.50] md-miR156_9 NC_024253 1

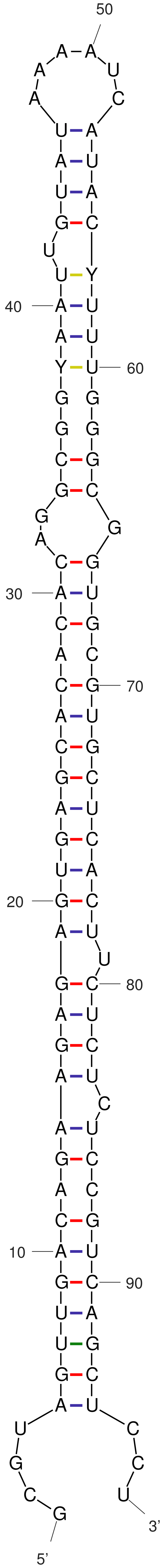

dG = -38.50 [Initially -41.50] md-miR156_10 NC_024253 1

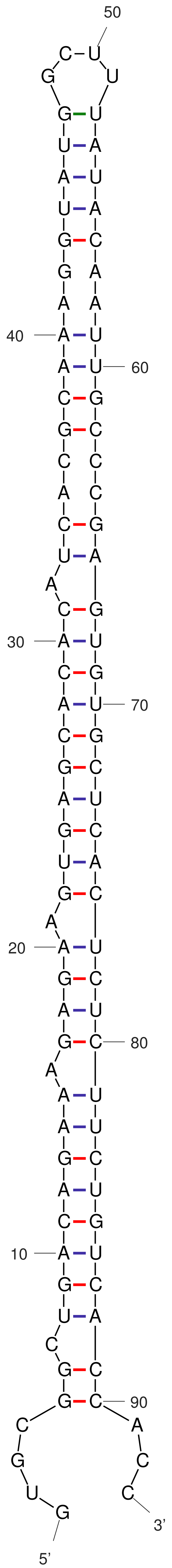

dG = -46.50 [Initially -46.50] md-miR156_11 NC_024253 1

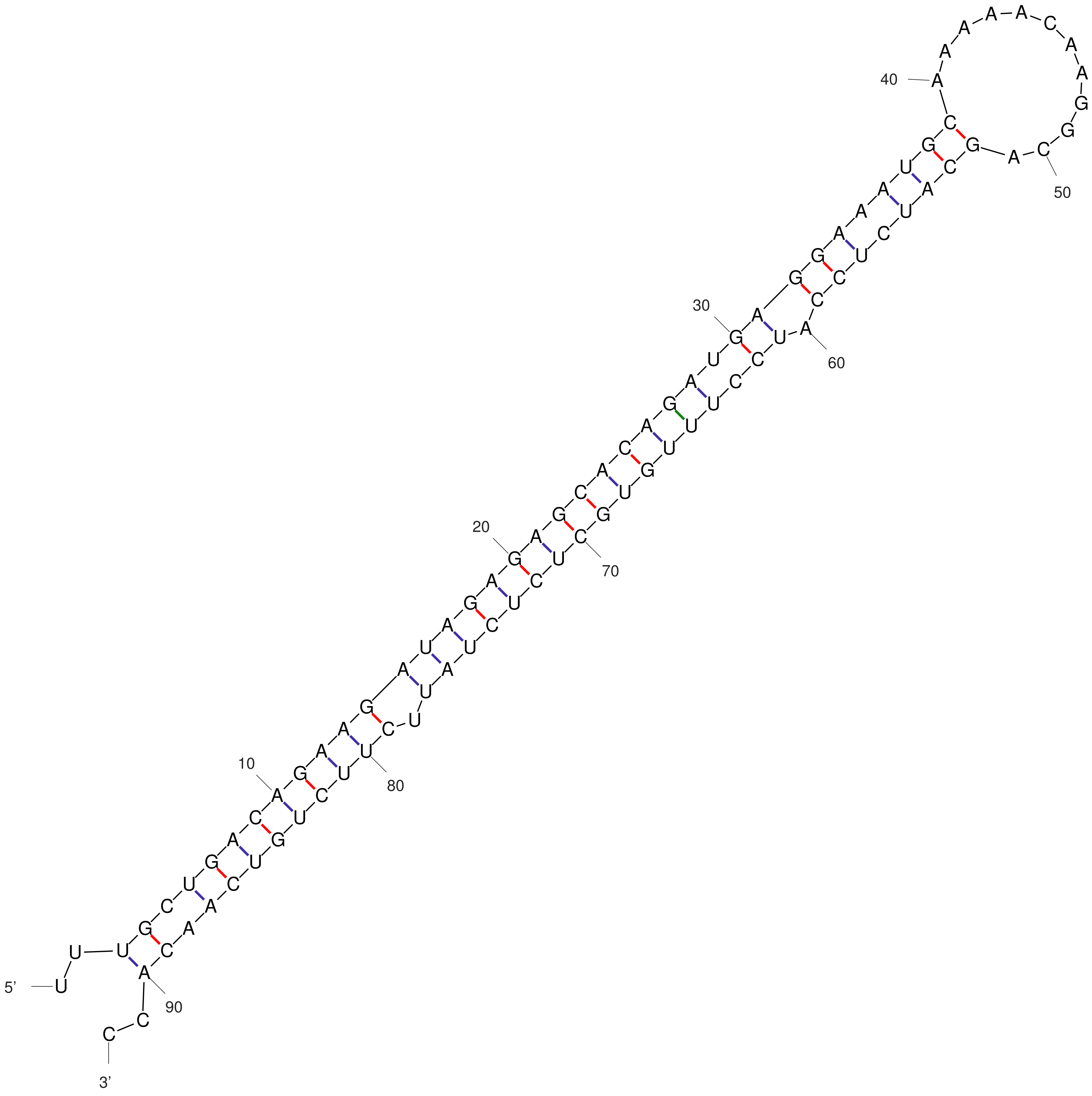

dG = -46.30 [Initially -46.30] md-miR156_13 NW_007545266 1

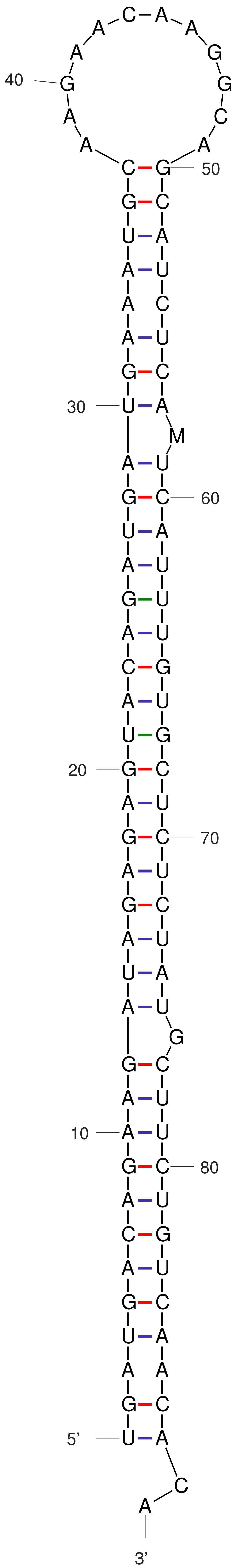

dG = -46.20 [Initially -46.20] md-miR156_14 NW_007545266 1

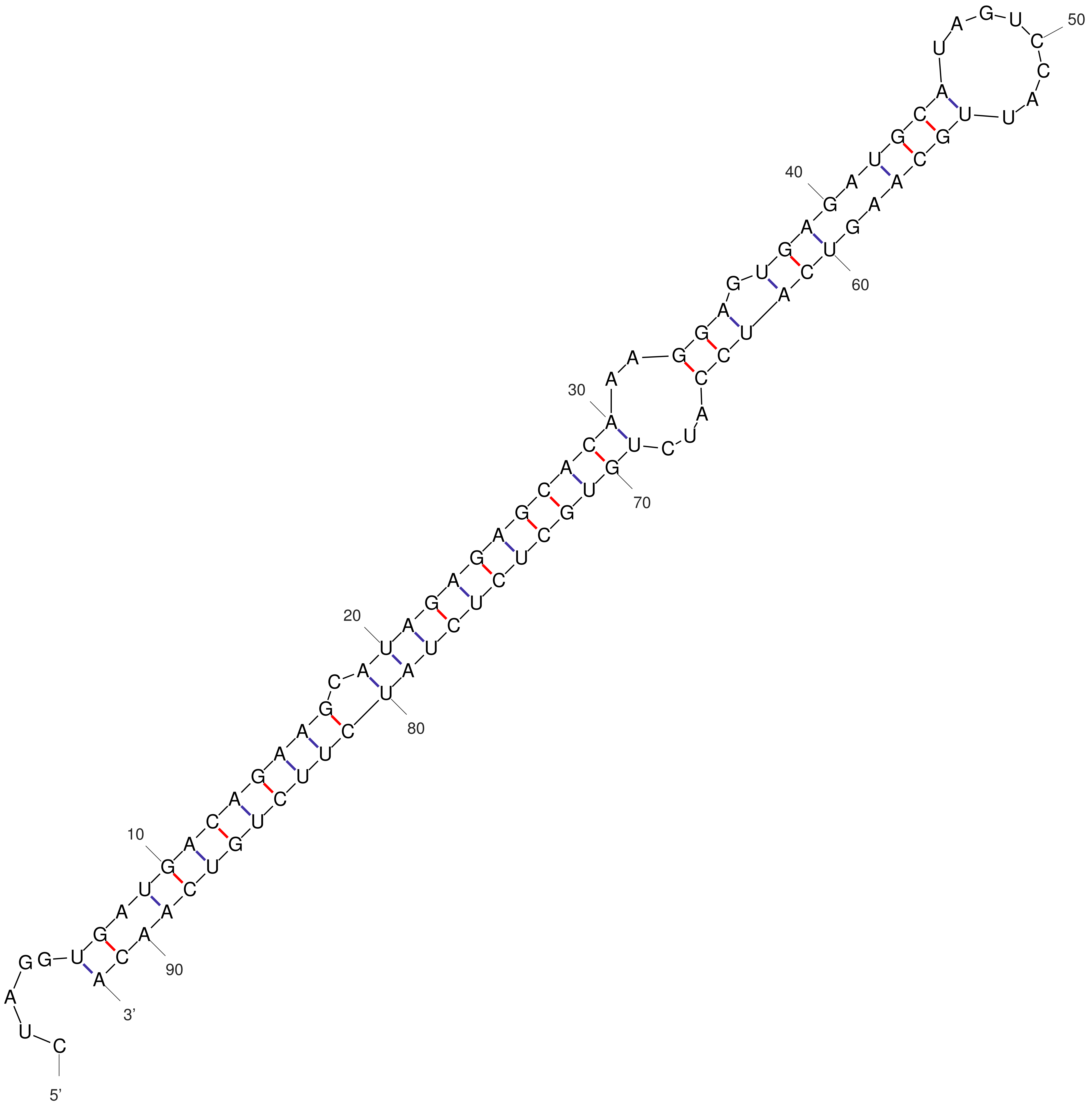

dG = -44.20 [Initially -44.40] md-miR156_15 NW_007545344 1

Output of sir_graph (©)
mfold_util 4.7

Created Mon Jul 22 08:19:01 2019

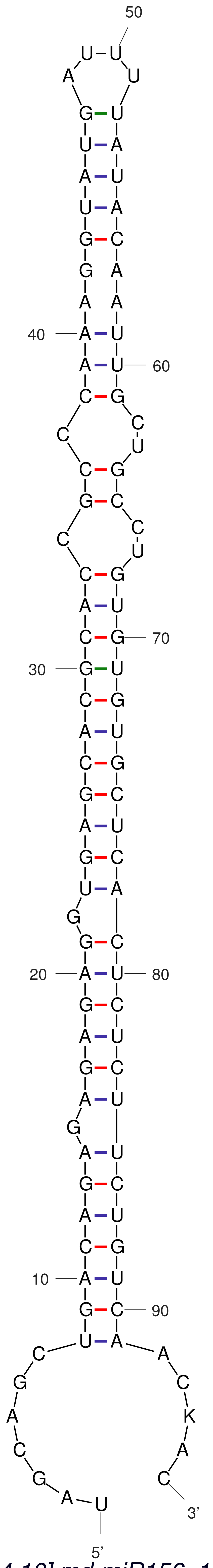

dG = -44.10 [Initially -44.10] md-miR156_16 NW_007545395 1

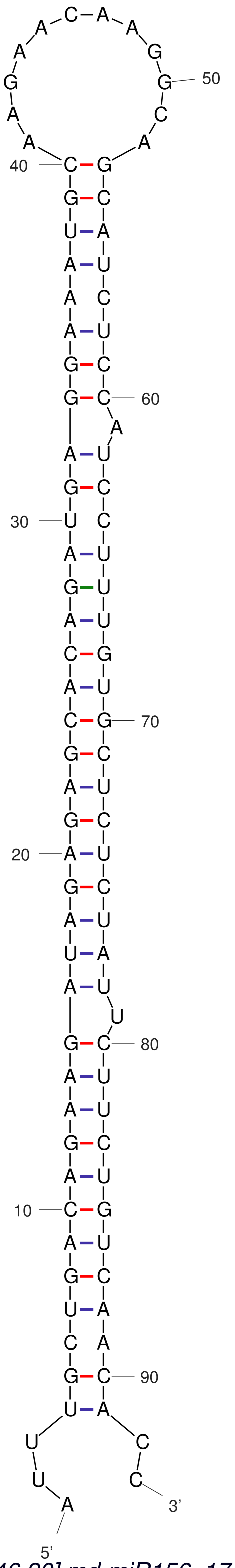

dG = -46.30 [Initially -46.30] md-miR156_17 NW_007545549 1

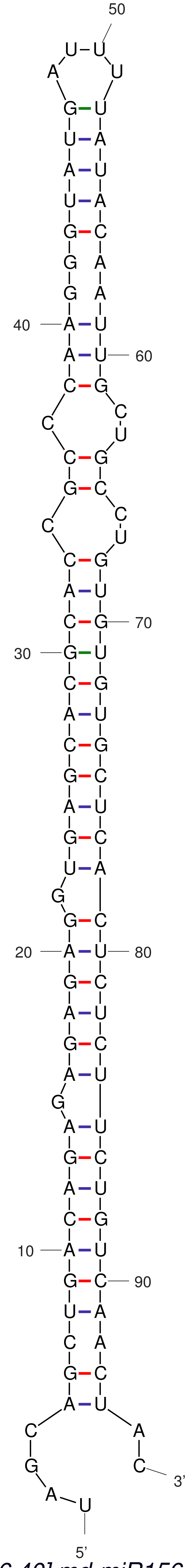

dG = -46.40 [Initially -46.40] md-miR156_18 NW_007545756 1

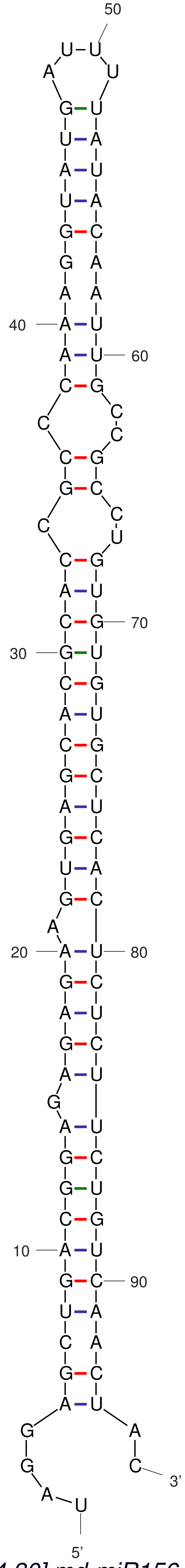

dG = -44.30 [Initially -44.30] md-miR156_19 NW_007545756 1

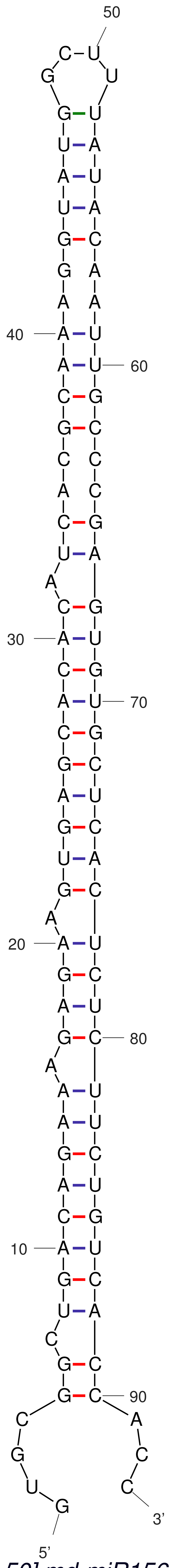

dG = -46.50 [Initially -46.50] md-miR156_20 NW_007546204 1

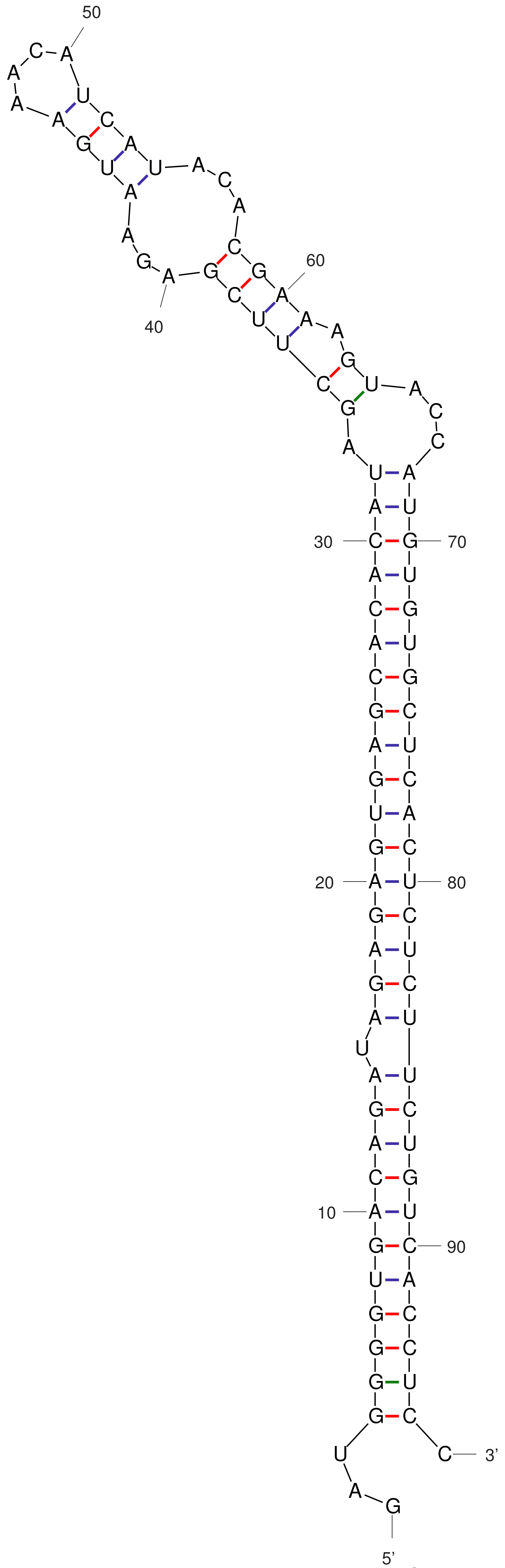

dG = -55.40 [Initially -55.40] mt-miR156_1 NC_016407 2

Output of sir_graph (©)
mfold_util 4.7

Created Mon Jul 22 08:40:33 2019

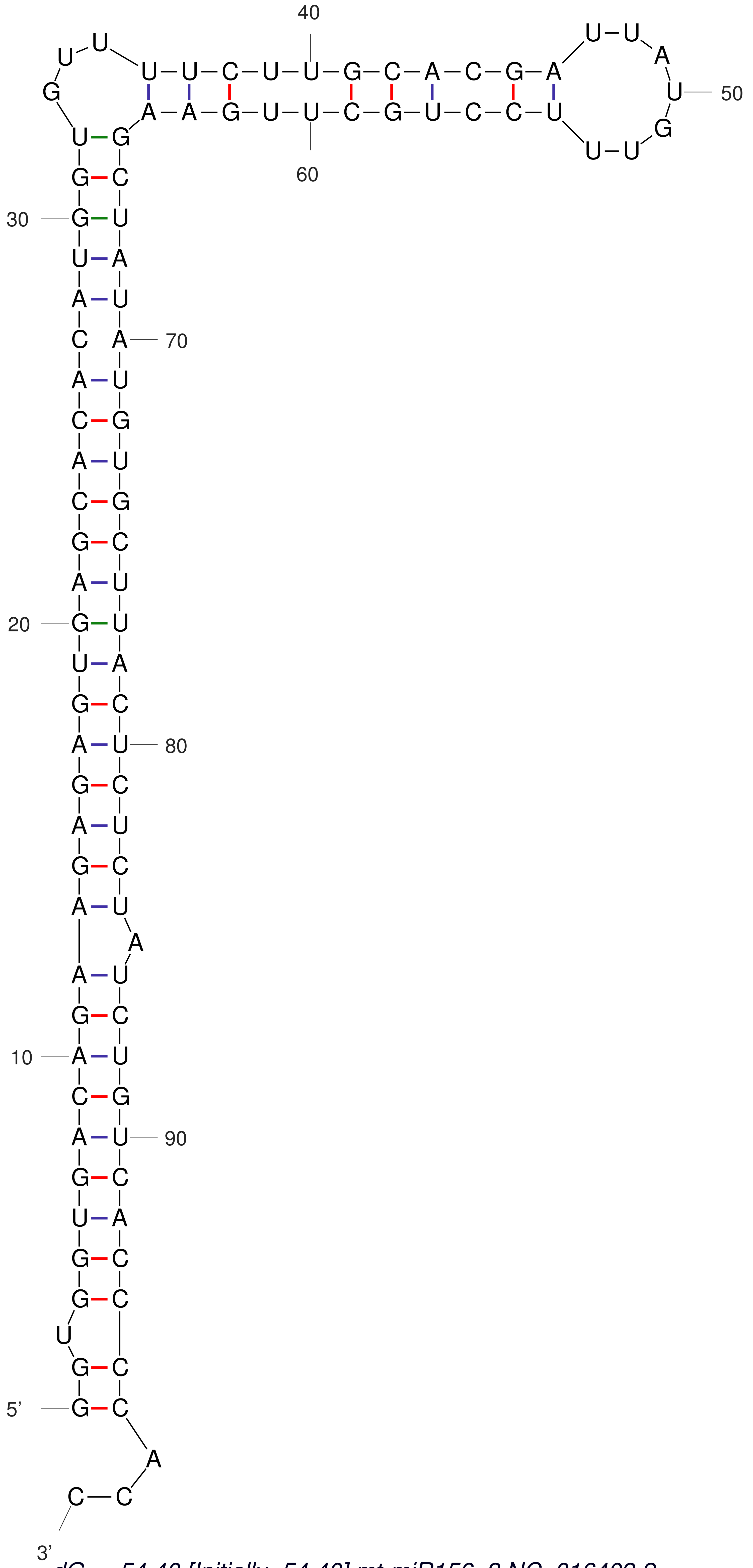

dG = -54.40 [Initially -54.40] mt-miR156_3 NC_016409 2

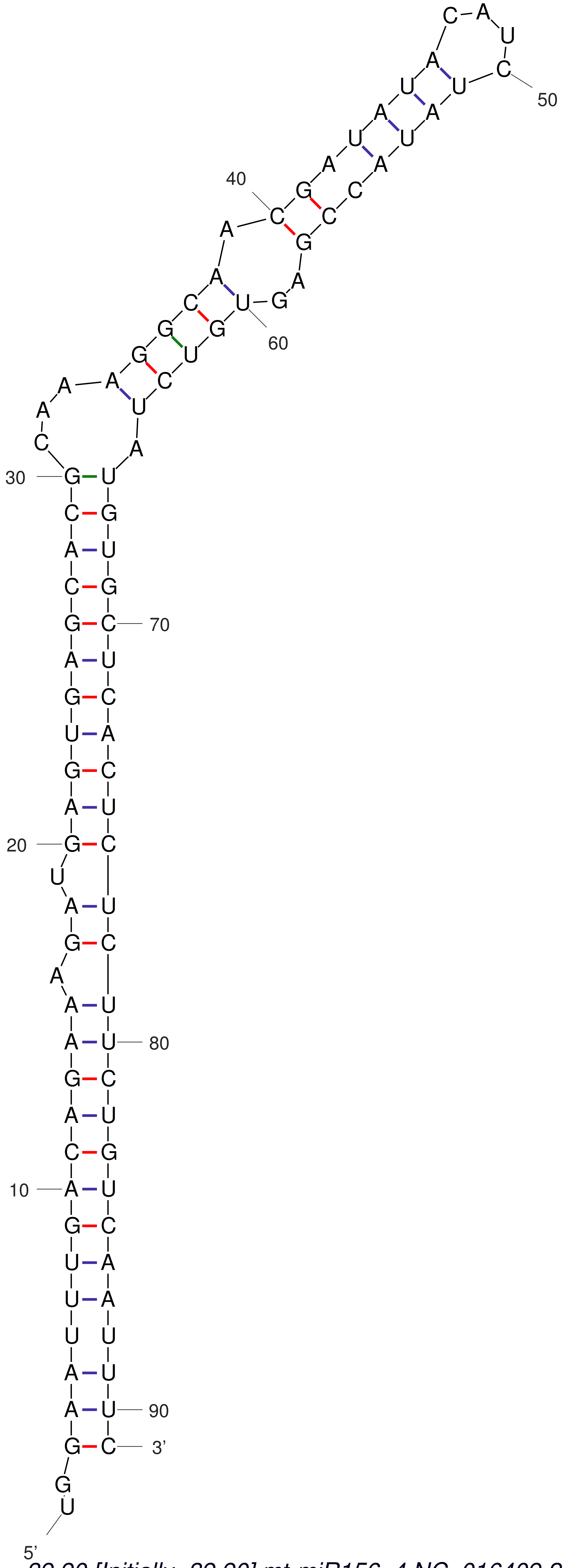

dG = -39.90 [Initially -39.90] mt-miR156_4 NC_016409 2

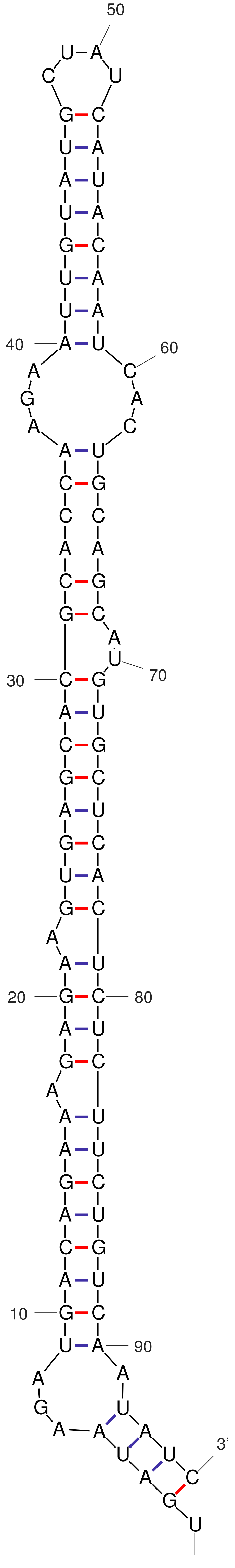

dG = -38.20 [Initially -38.20] mt-miR156_5 NC_016410 2

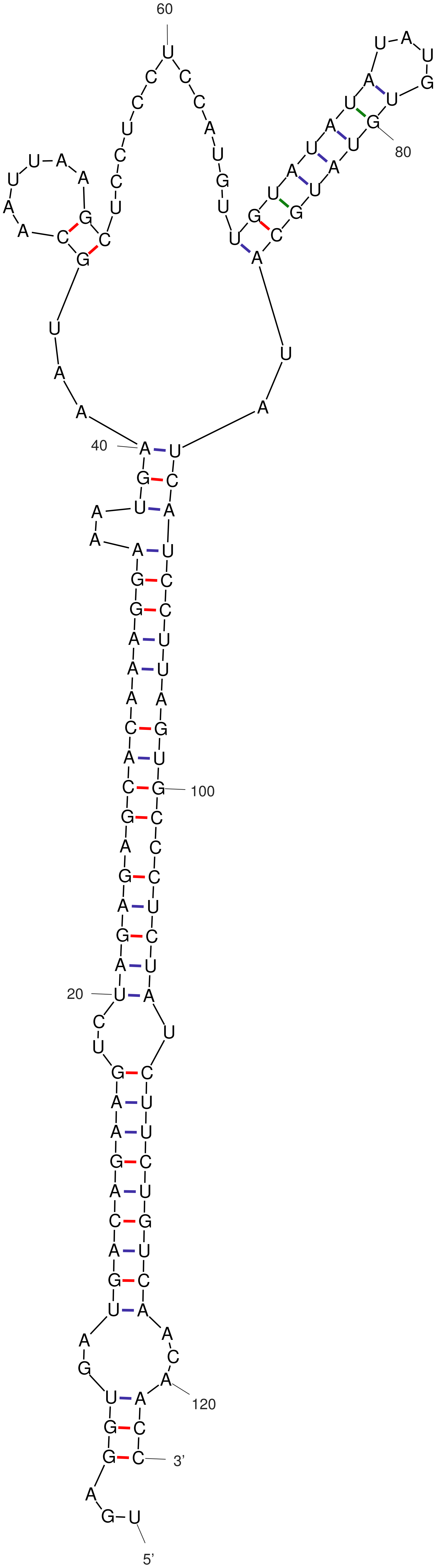

dG = -39.09 [Initially -42.90] mt-miR156_6 NC_016412 2

Output of sir_graph (©)
mfold_util 4.7

Created Mon Jul 22 08:42:37 2019

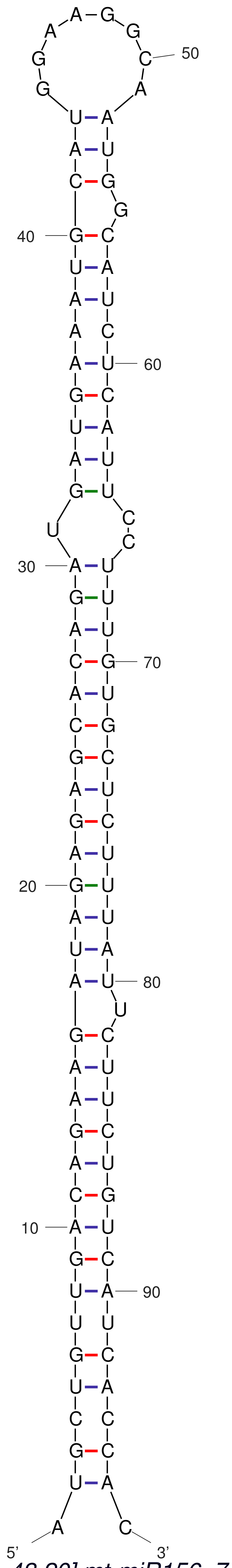

dG = -43.20 [Initially -43.20] mt-miR156_7 NC_016413 2

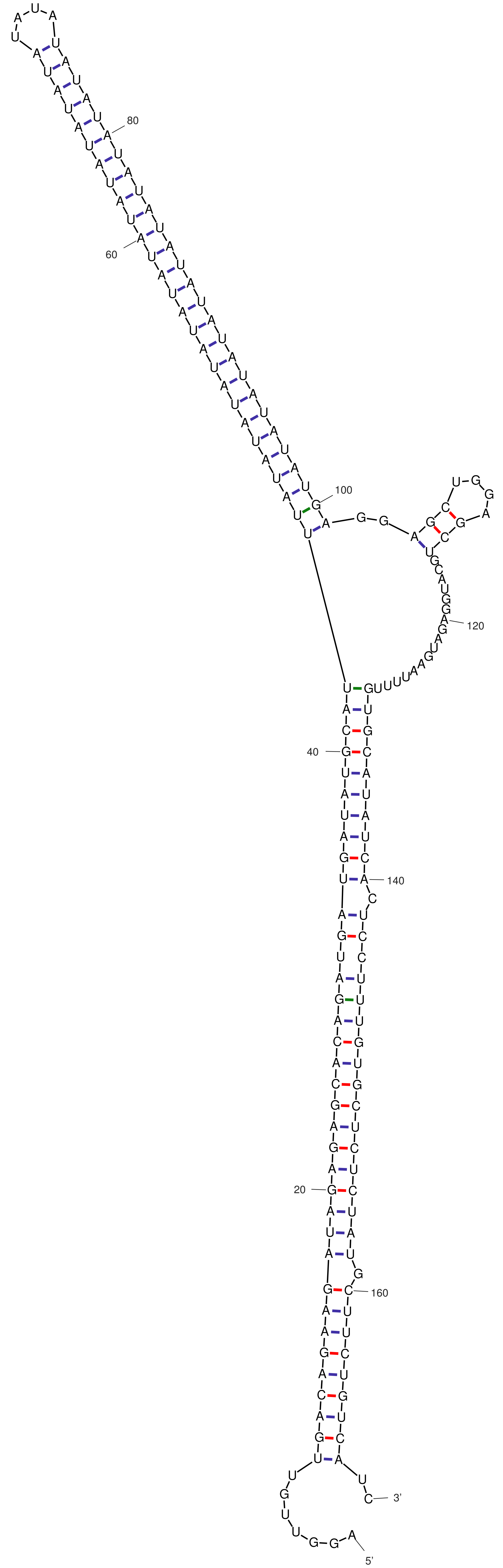

dG = -75.93 [Initially -79.40] mt-miR156_8 NC_016414 2

Output of sir_graph (©)
mfold_util 4.7

Created Tue May 21 00:30:21 2019

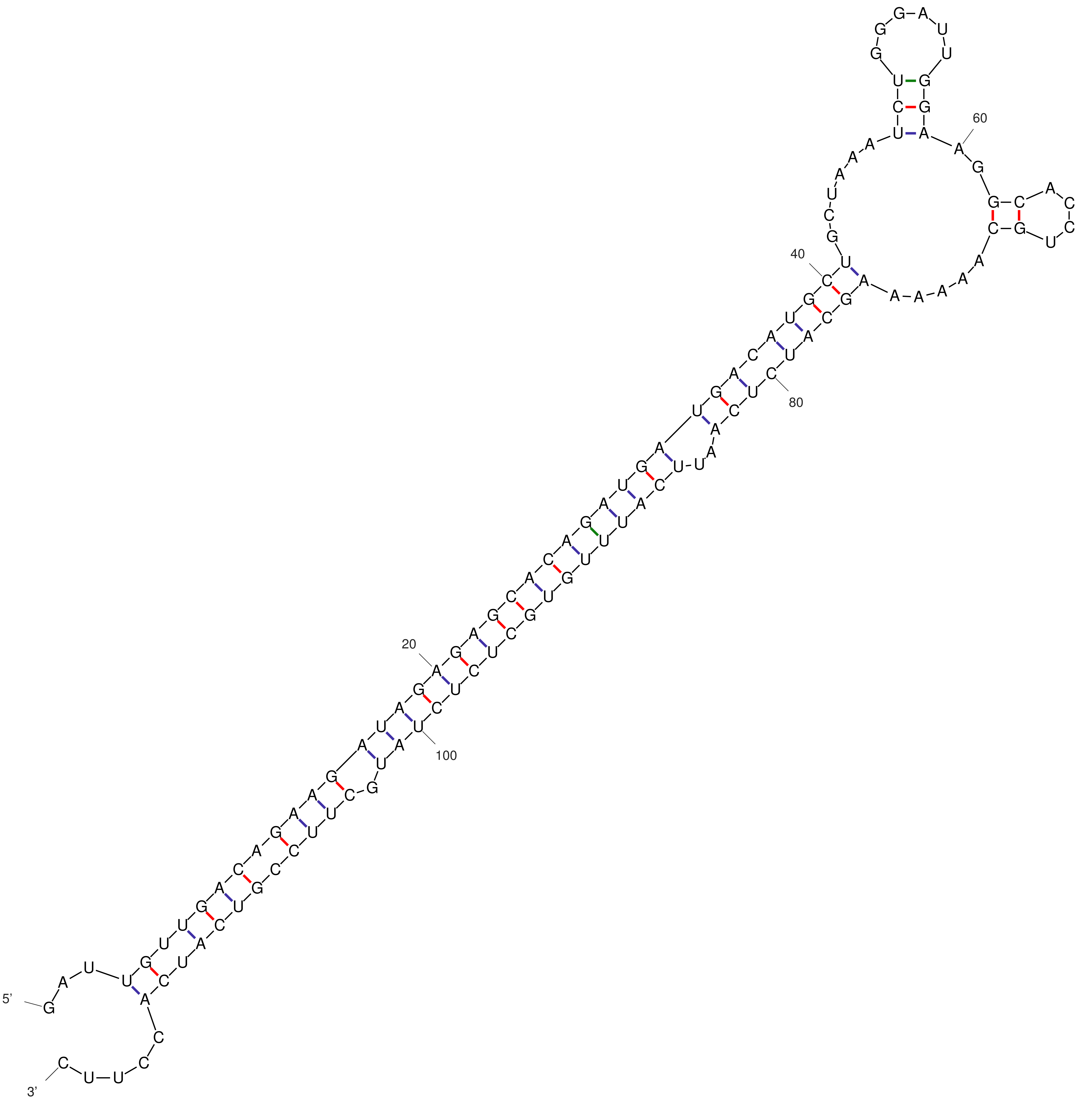

dG = -44.35 [Initially -47.70] Nt-miR156_1 NW_015790845 1

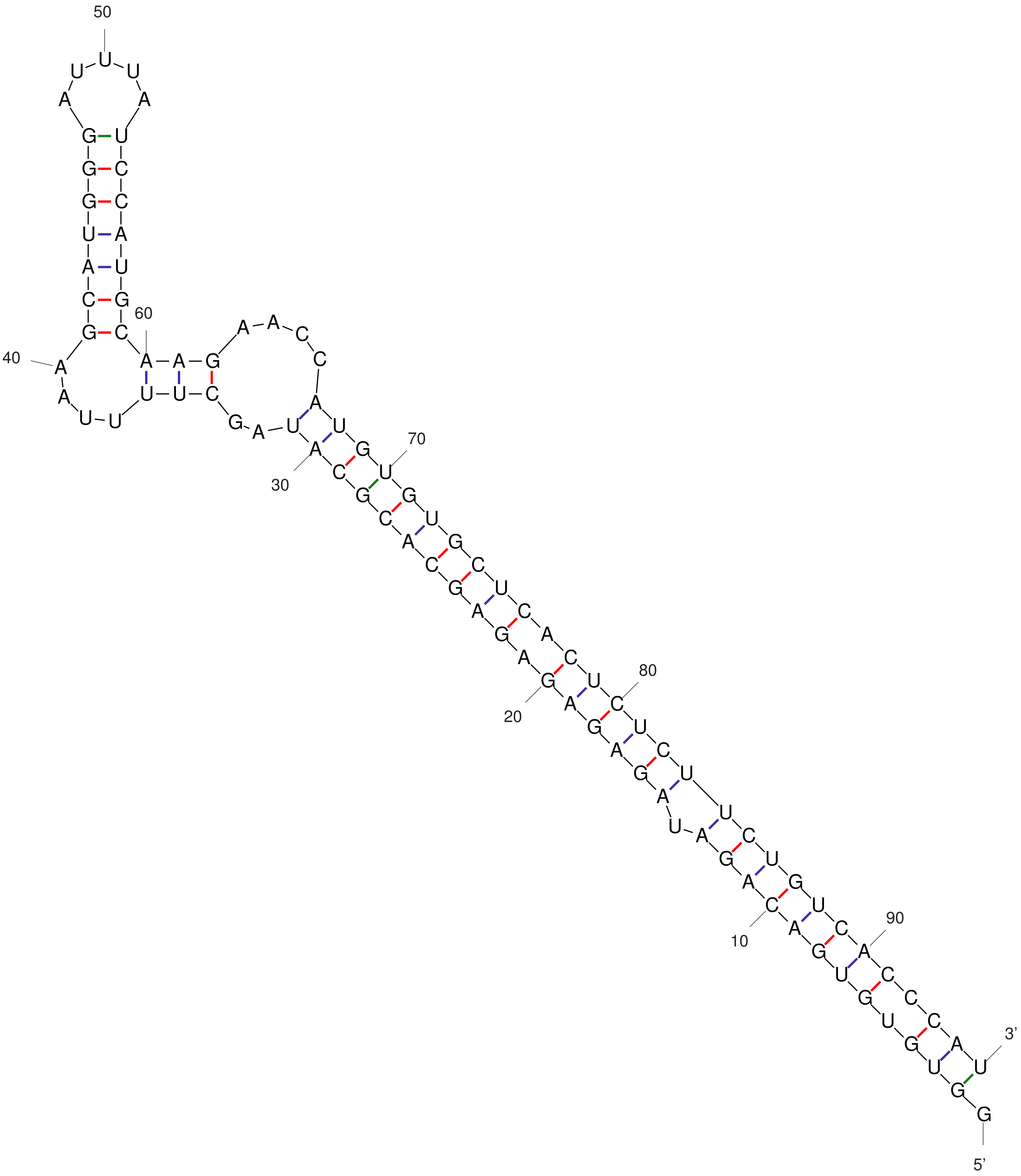

dG = -51.70 [Initially -51.70] Nt-miR156_2 NW_015793035 1

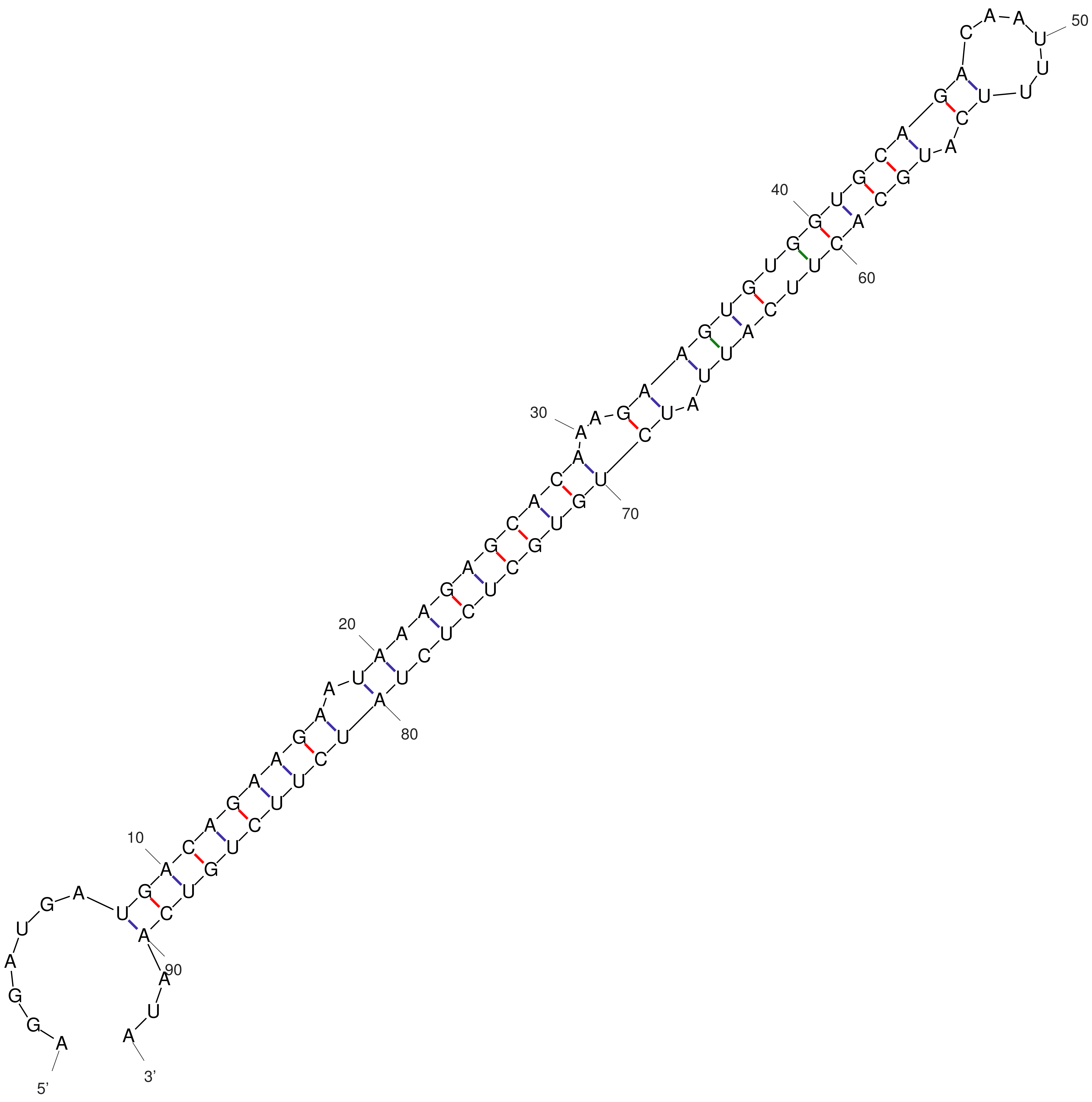

dG = -39.20 [Initially -39.20] Nt-miR156_3 NW_015799498 1

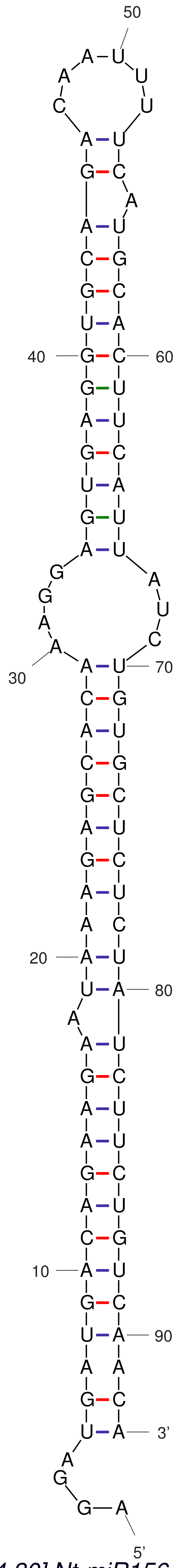

dG = -44.30 [Initially -44.30] Nt-miR156_4 NW_015799498 1

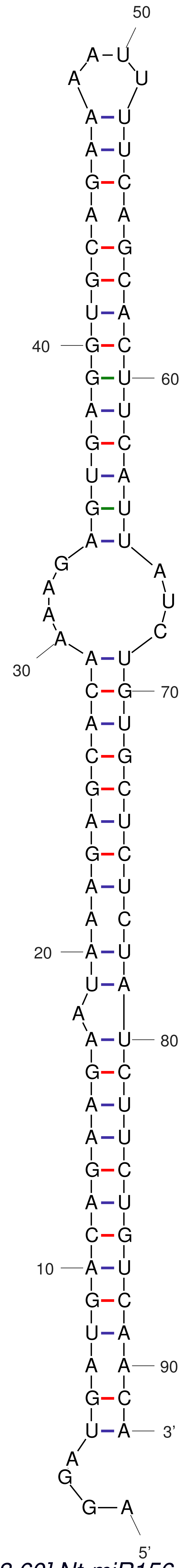

dG = -43.60 [Initially -43.60] Nt-miR156_5 NW_015799498 1

Output of sir_graph (©)
mfold_util 4.7

Created Tue May 21 00:35:49 2019

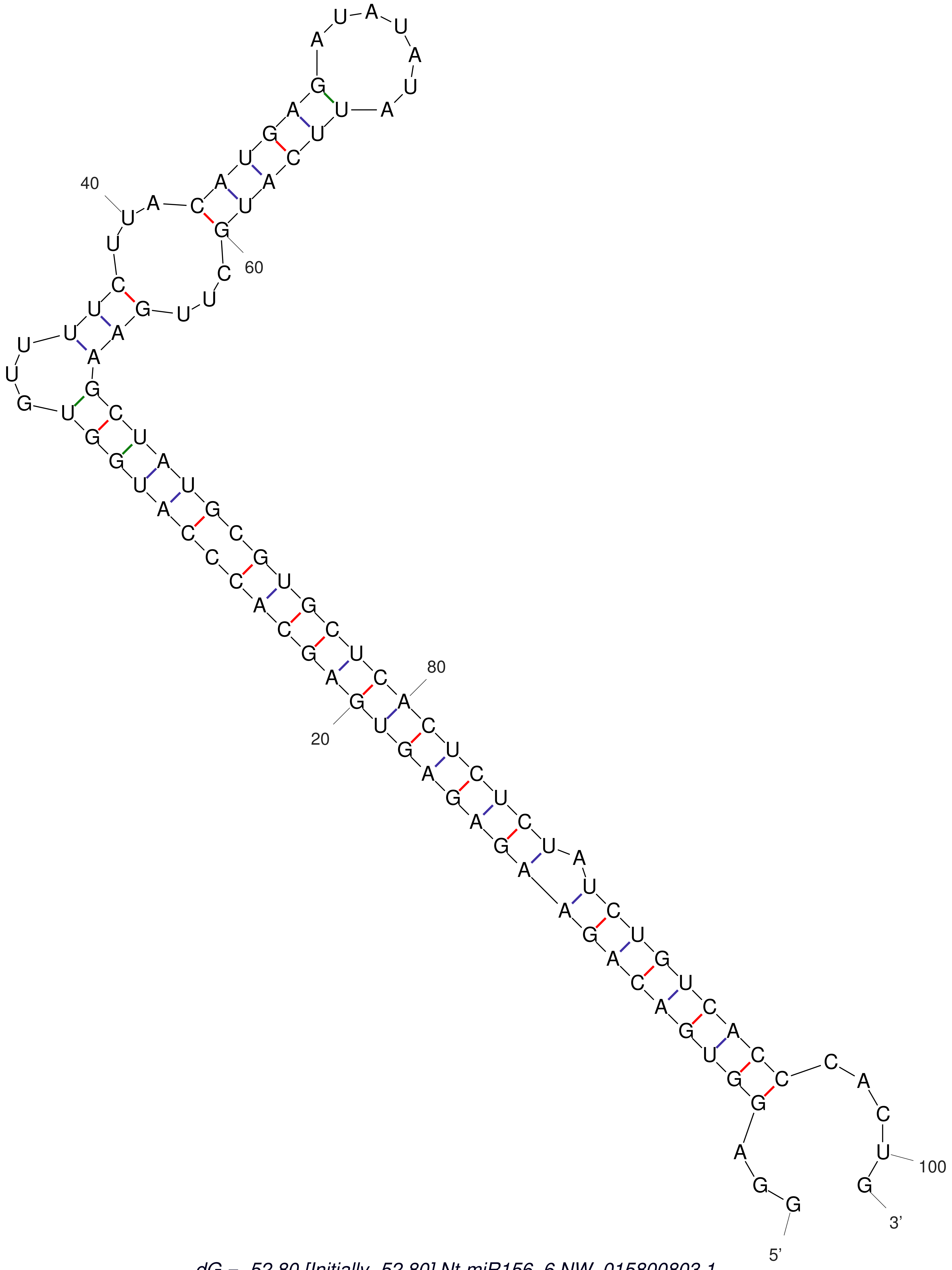

dG = -52.80 [Initially -52.80] Nt-miR156_6 NW_015800803 1

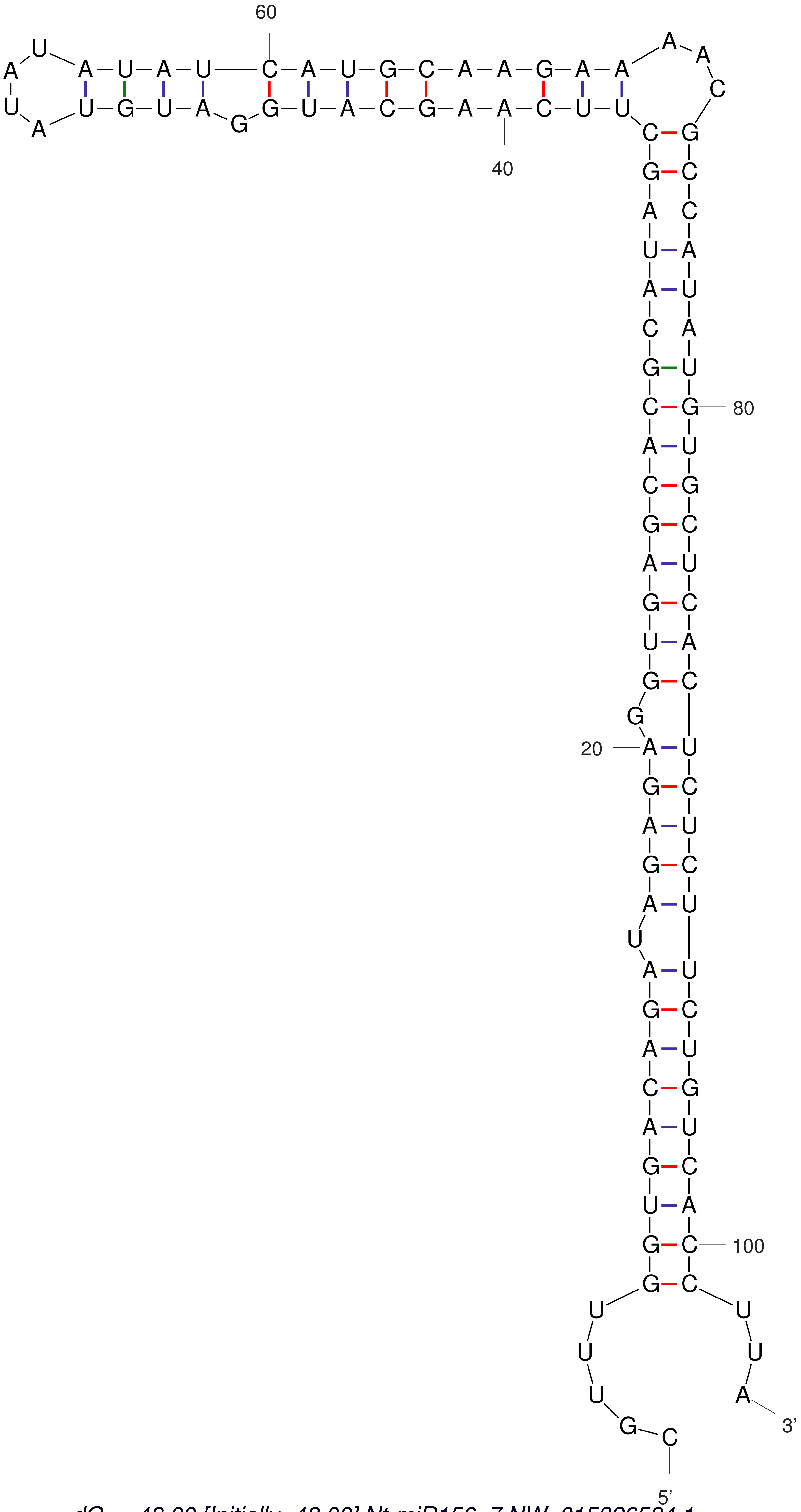

dG = -48.00 [Initially -48.00] Nt-miR156_7 NW_015826524 1

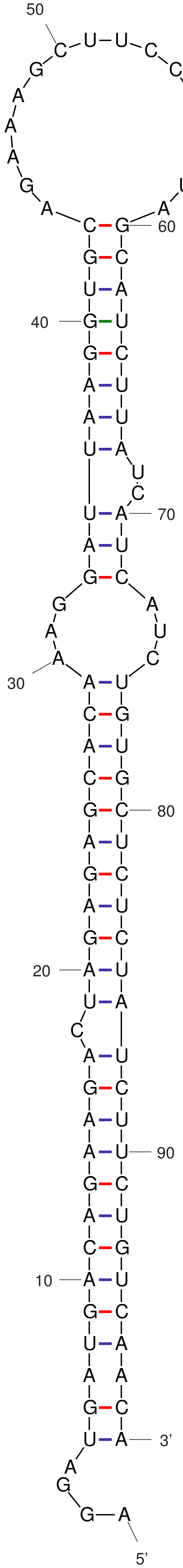

dG = -44.30 [Initially -44.30] Nt-miR156_8 NW_015841047 1

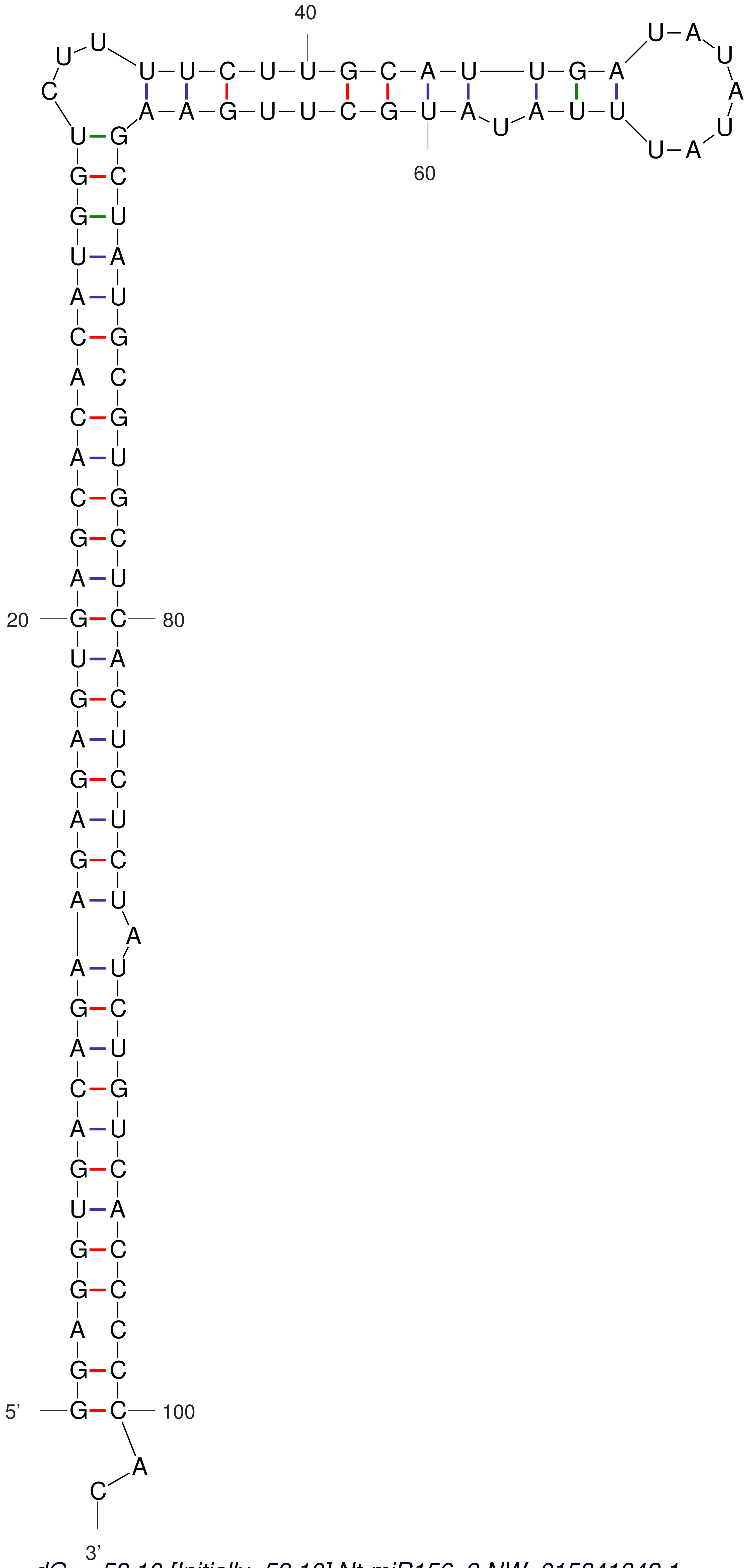

dG = -58.10 [Initially -58.10] Nt-miR156_9 NW_015841842 1

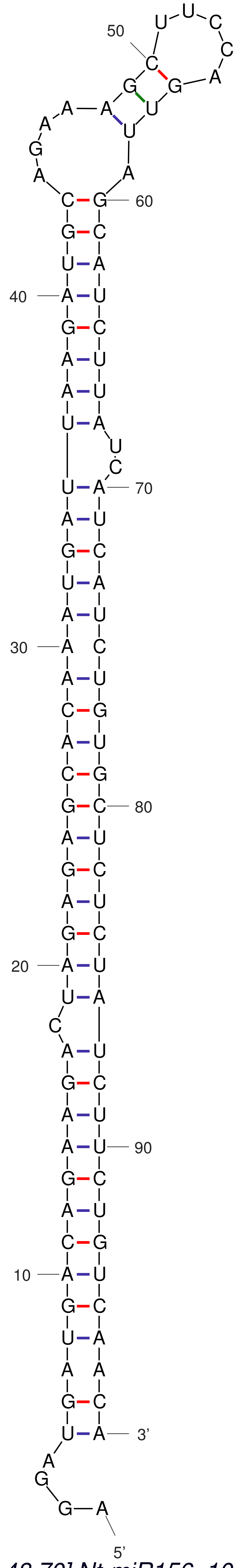

dG = -48.70 [Initially -48.70] Nt-miR156_10 NW_015848029 1

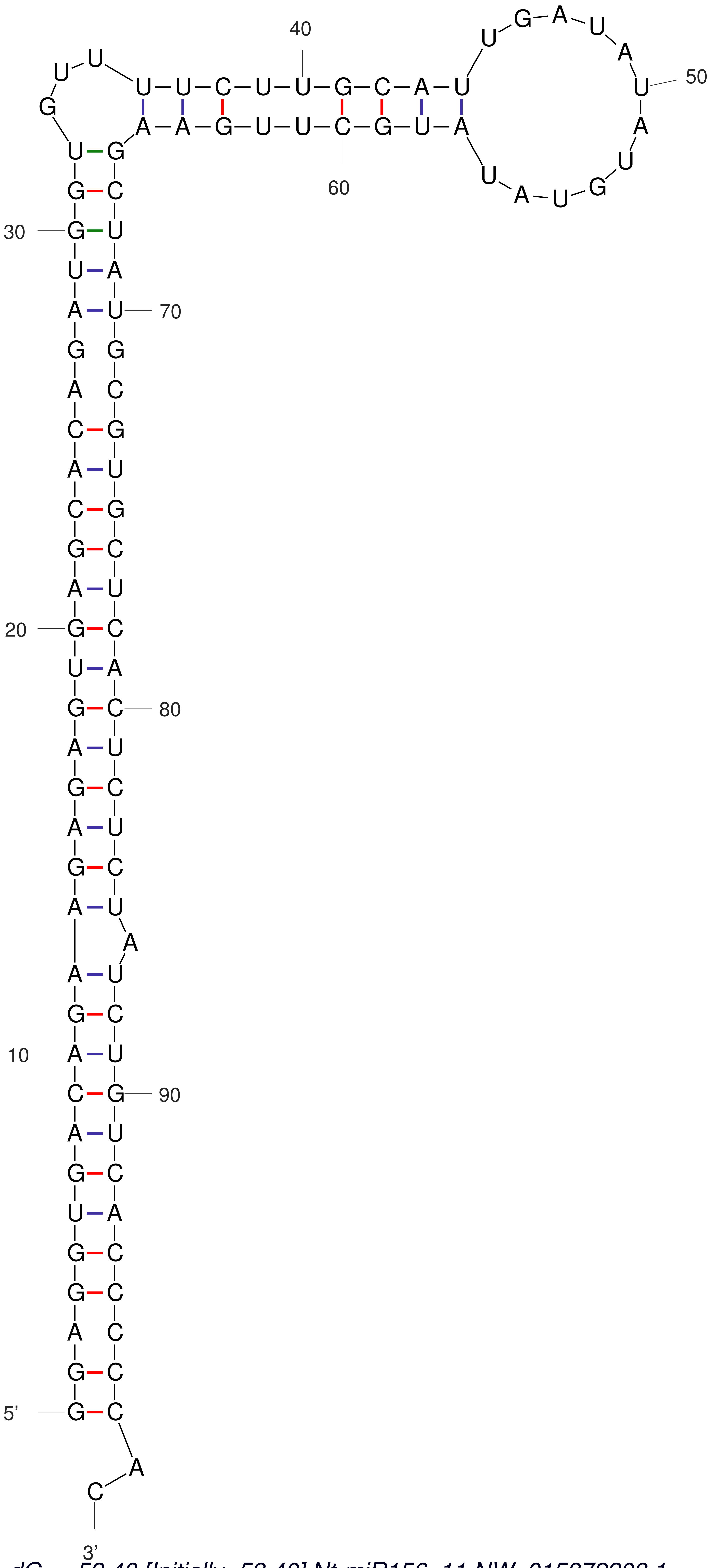

dG = -53.40 [Initially -53.40] Nt-miR156_11 NW_015872208 1

Output of sir_graph (©)
mfold_util 4.7

Created Tue May 21 00:37:31 2019

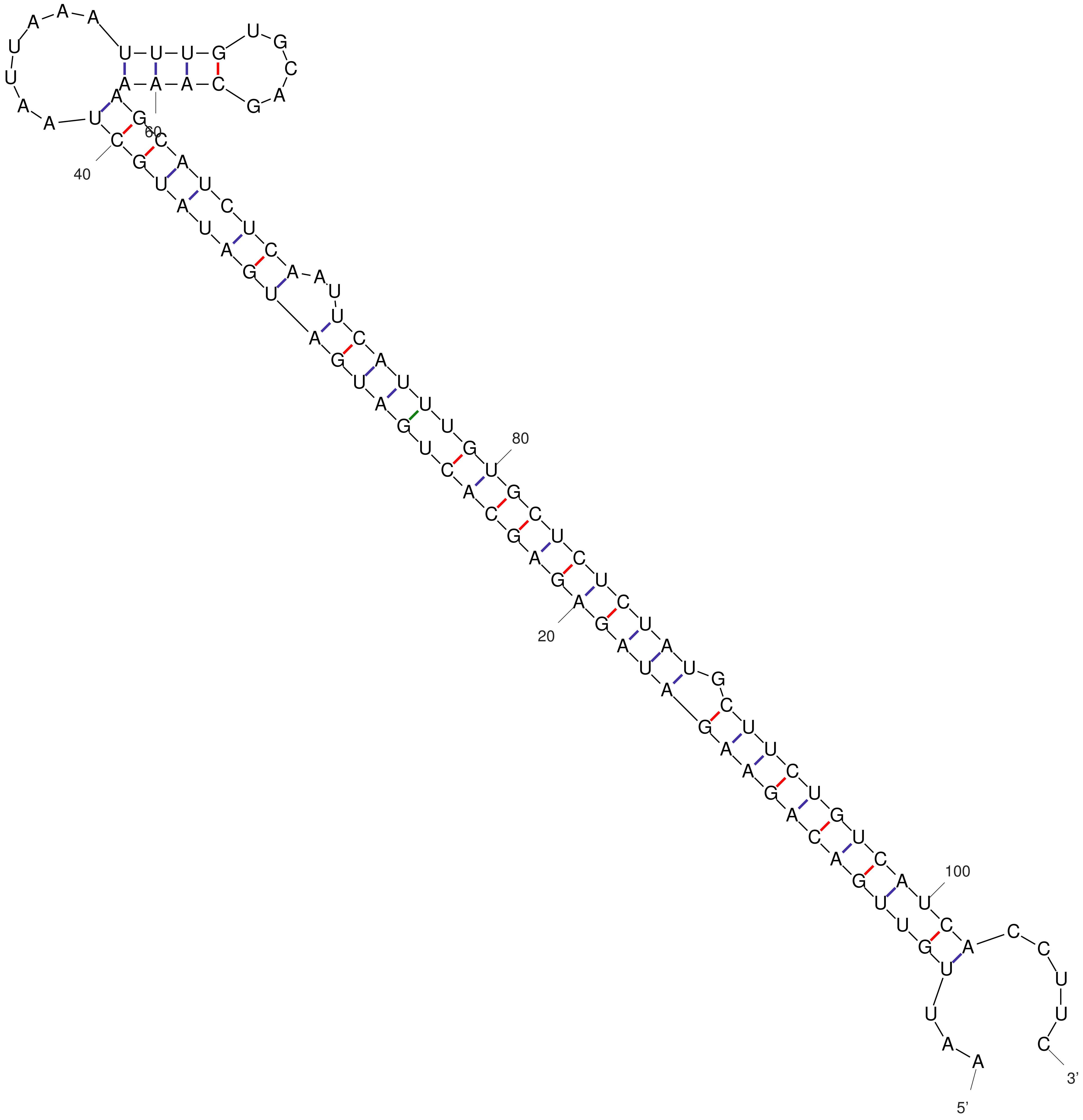

dG = -45.80 [Initially -45.80] Nt-miR156_12 NW_015884734 1

Output of sir_graph (©)
mfold_util 4.7

Created Tue May 21 00:37:37 2019

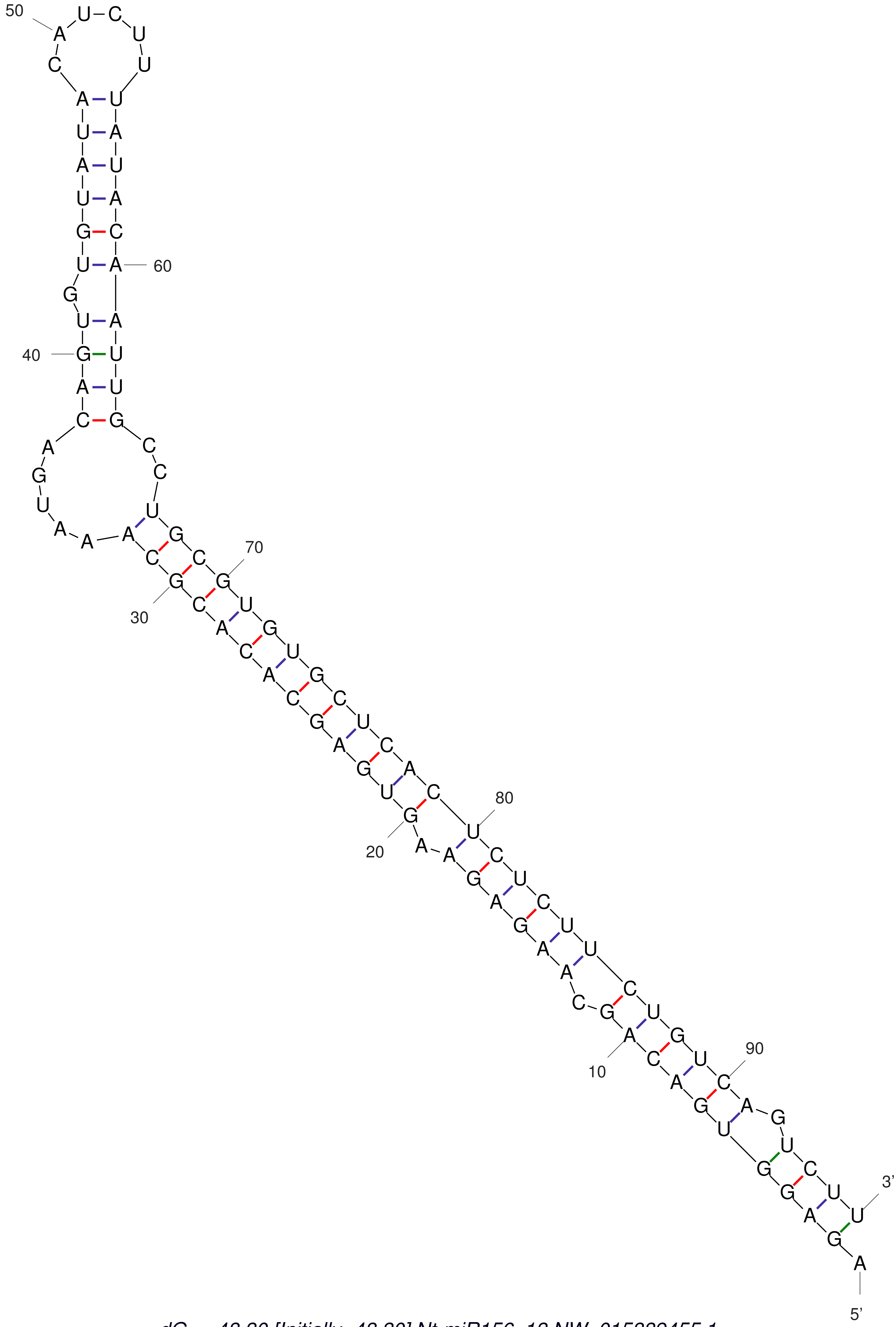

dG = -48.30 [Initially -48.30] Nt-miR156_13 NW_015889455 1

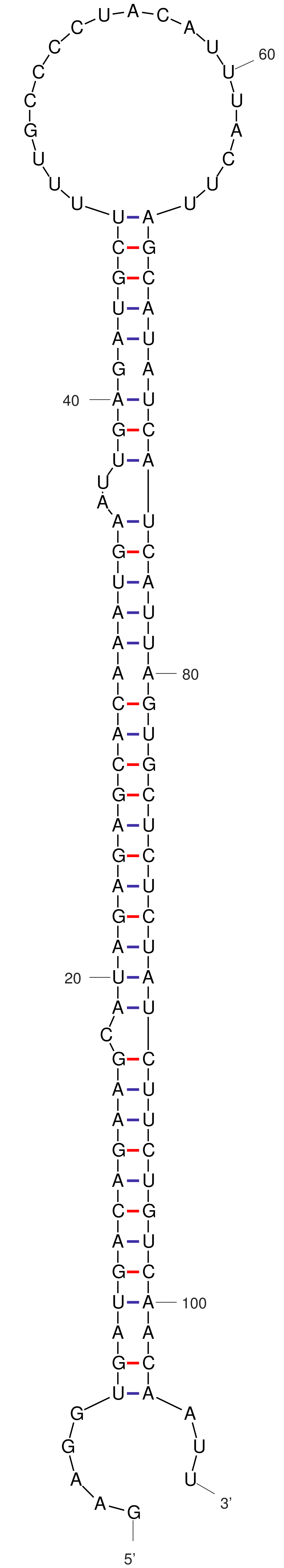

dG = -44.10 [Initially -44.30] Nt-miR156_14 NW_015891370 1

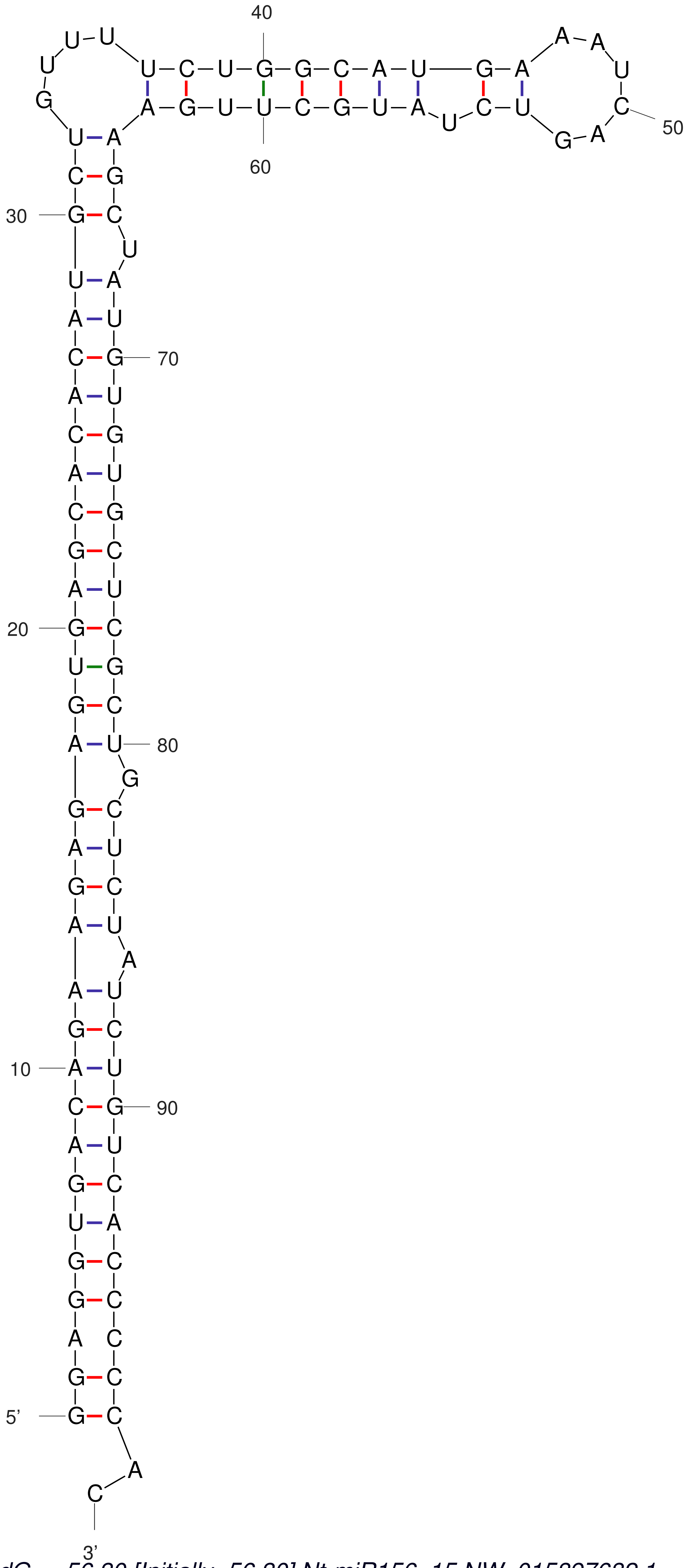

dG = -56.30 [Initially -56.30] Nt-miR156_15 NW_015897682 1

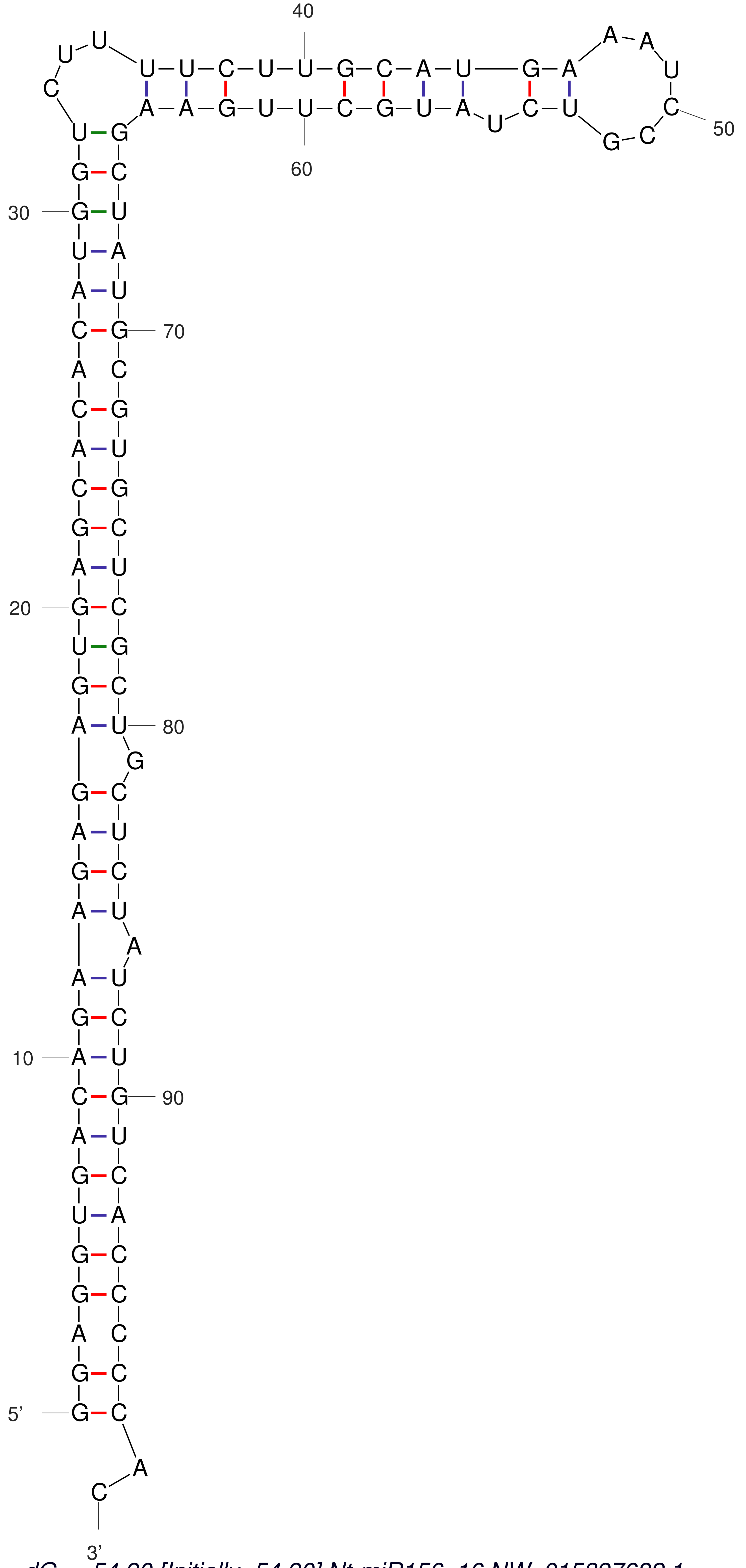

dG = -54.90 [Initially -54.90] Nt-miR156_16 NW_015897682 1

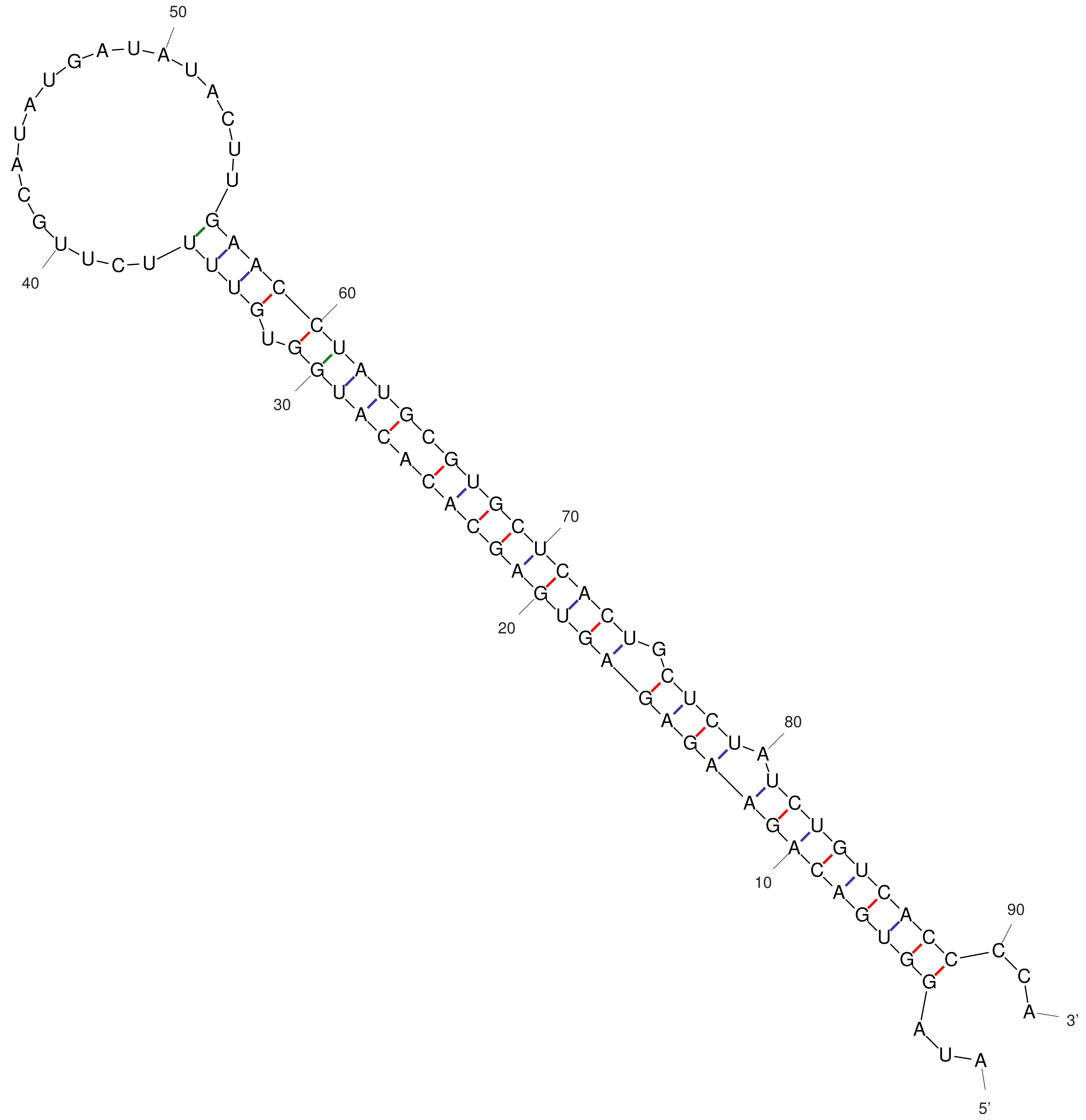

dG = -44.90 [Initially -44.90] Nt-miR156_17 NW_015897682 1

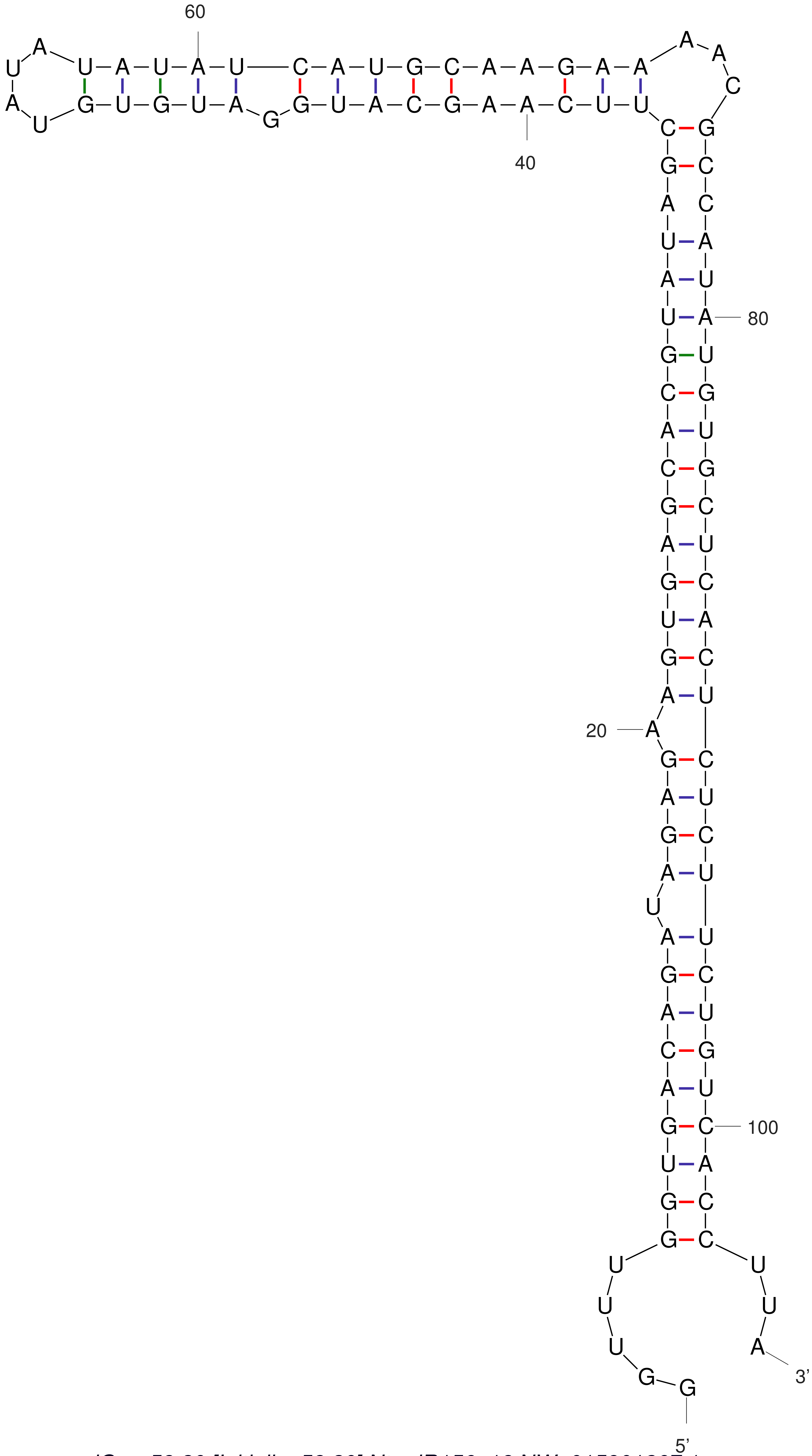

dG = -53.20 [Initially -53.20] Nt-miR156_18 NW_015901267 1

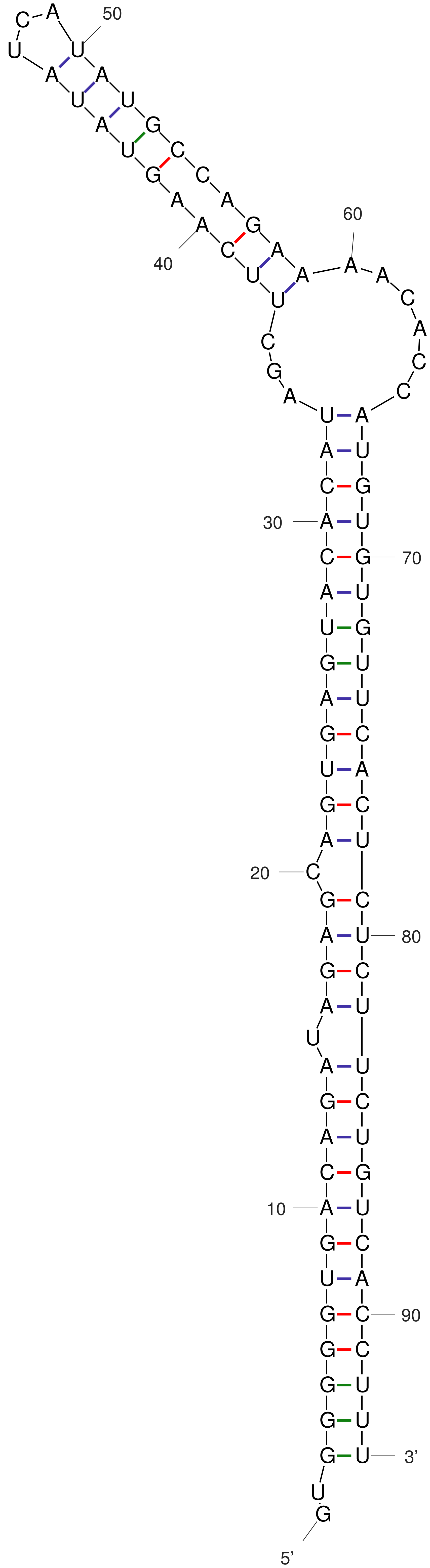

dG = -39.20 [Initially -39.20] Nt-miR156_19 NW_015902704 1

Output of sir_graph (©)
mfold_util 4.7

Created Tue May 21 00:39:36 2019

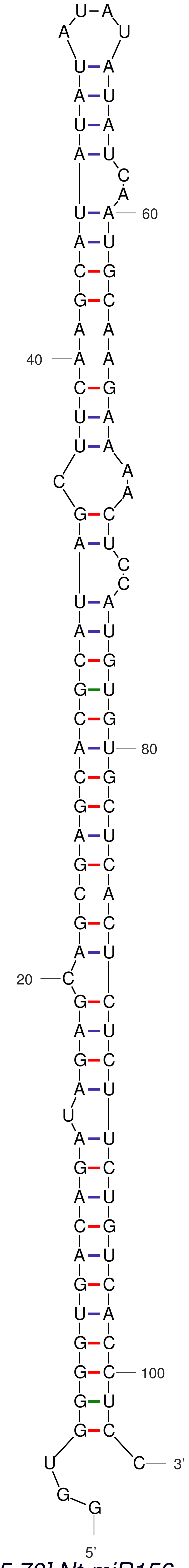

dG = -45.70 [Initially -45.70] Nt-miR156_20 NW_015902704 1

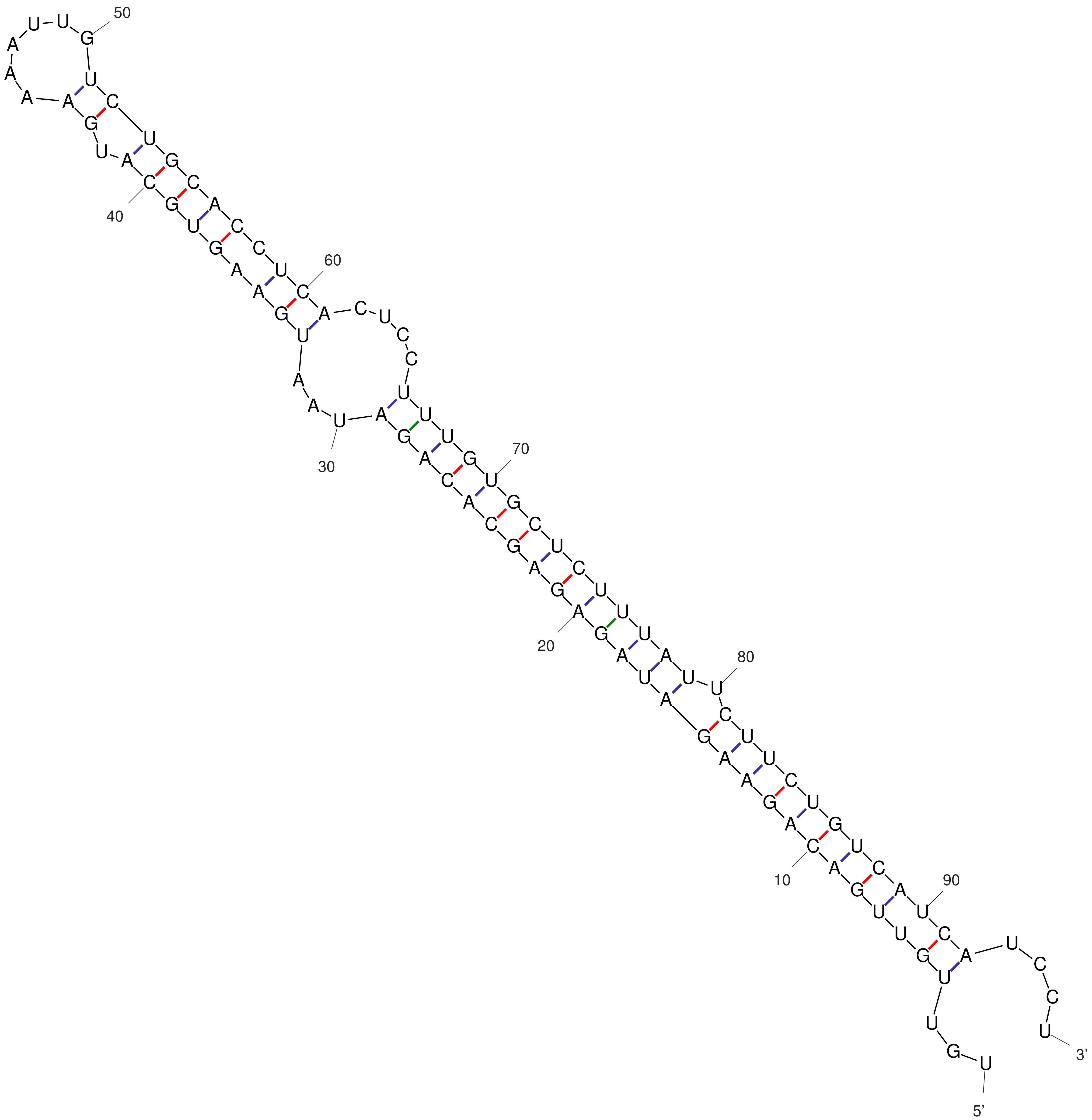

dG = -43.60 [Initially -43.60] Nt-miR156_21 NW_015907343 1

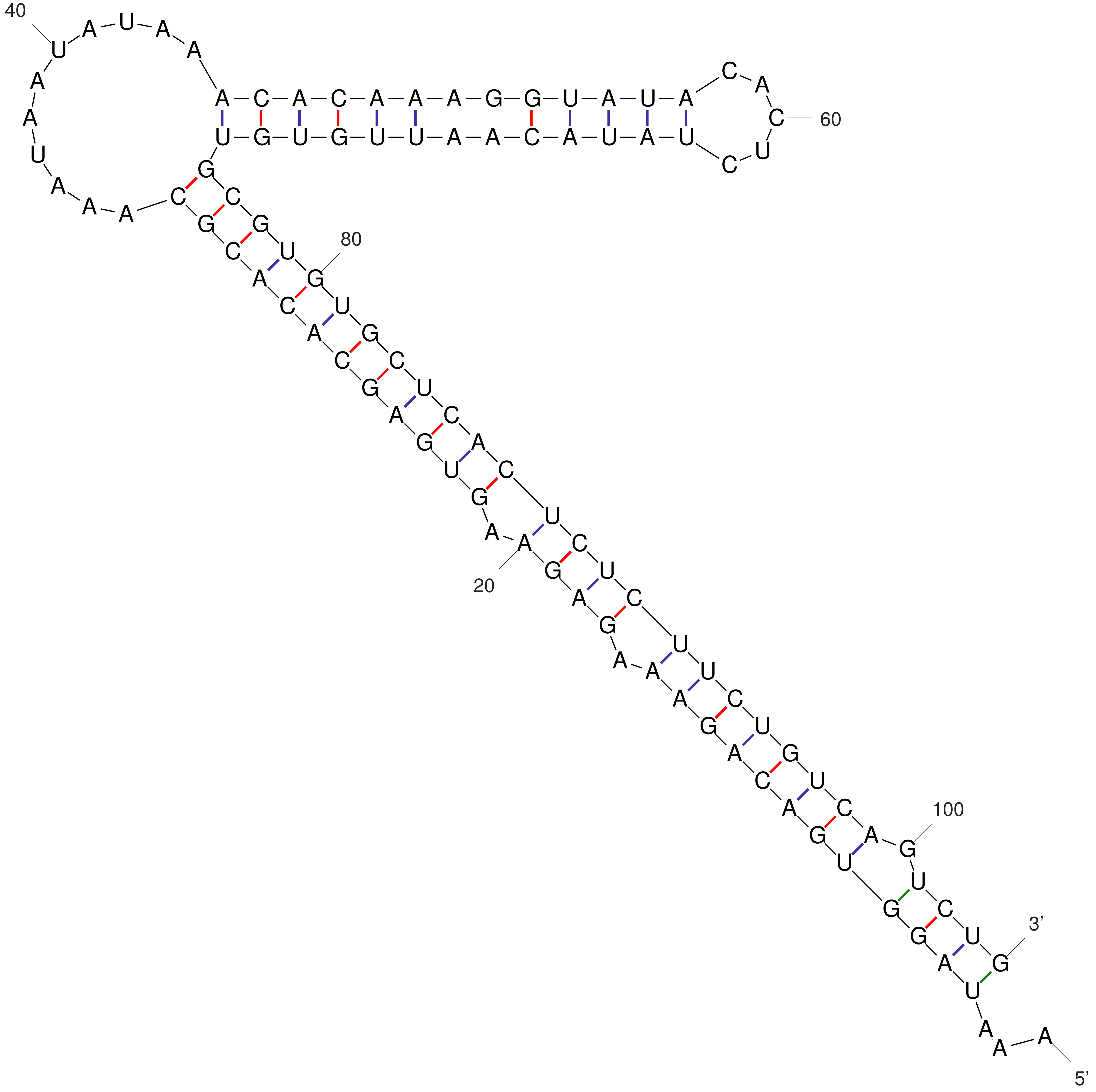

dG = -49.80 [Initially -49.80] Nt-miR156_22 NW_015908598 1

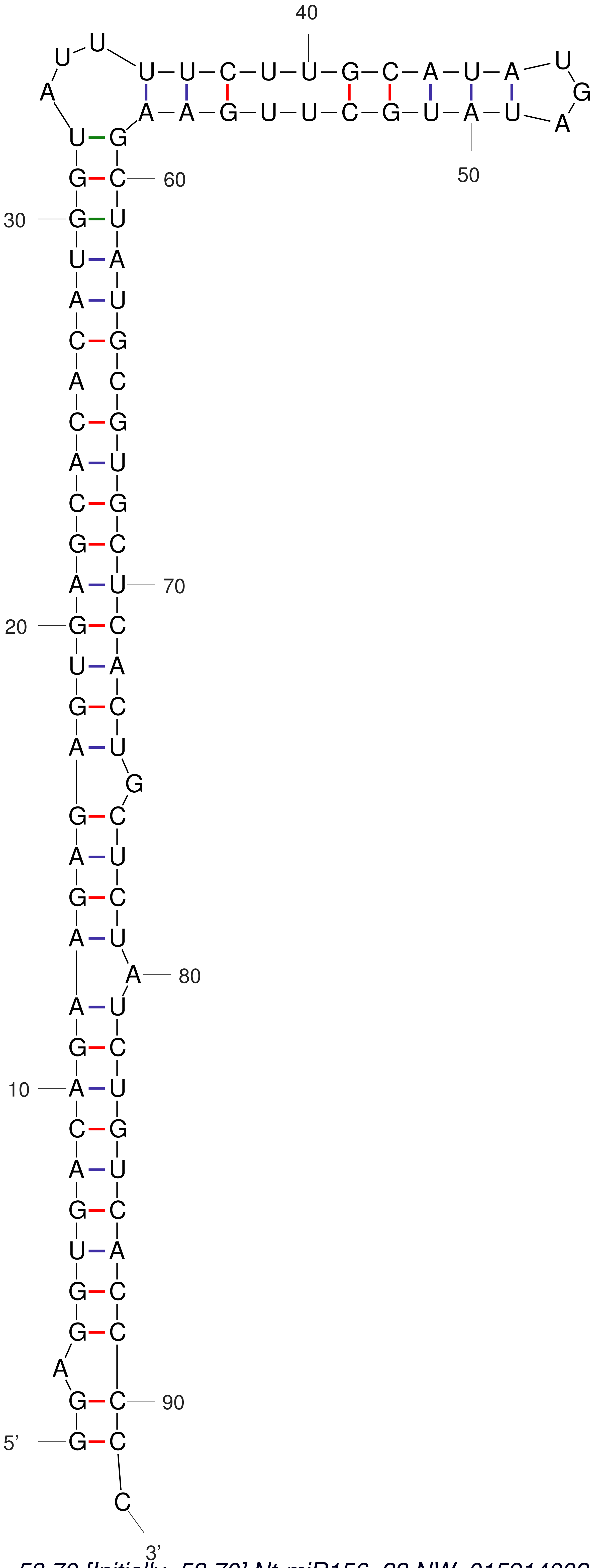

dG = -53.70 [Initially -53.70] Nt-miR156_23 NW_015914002 1

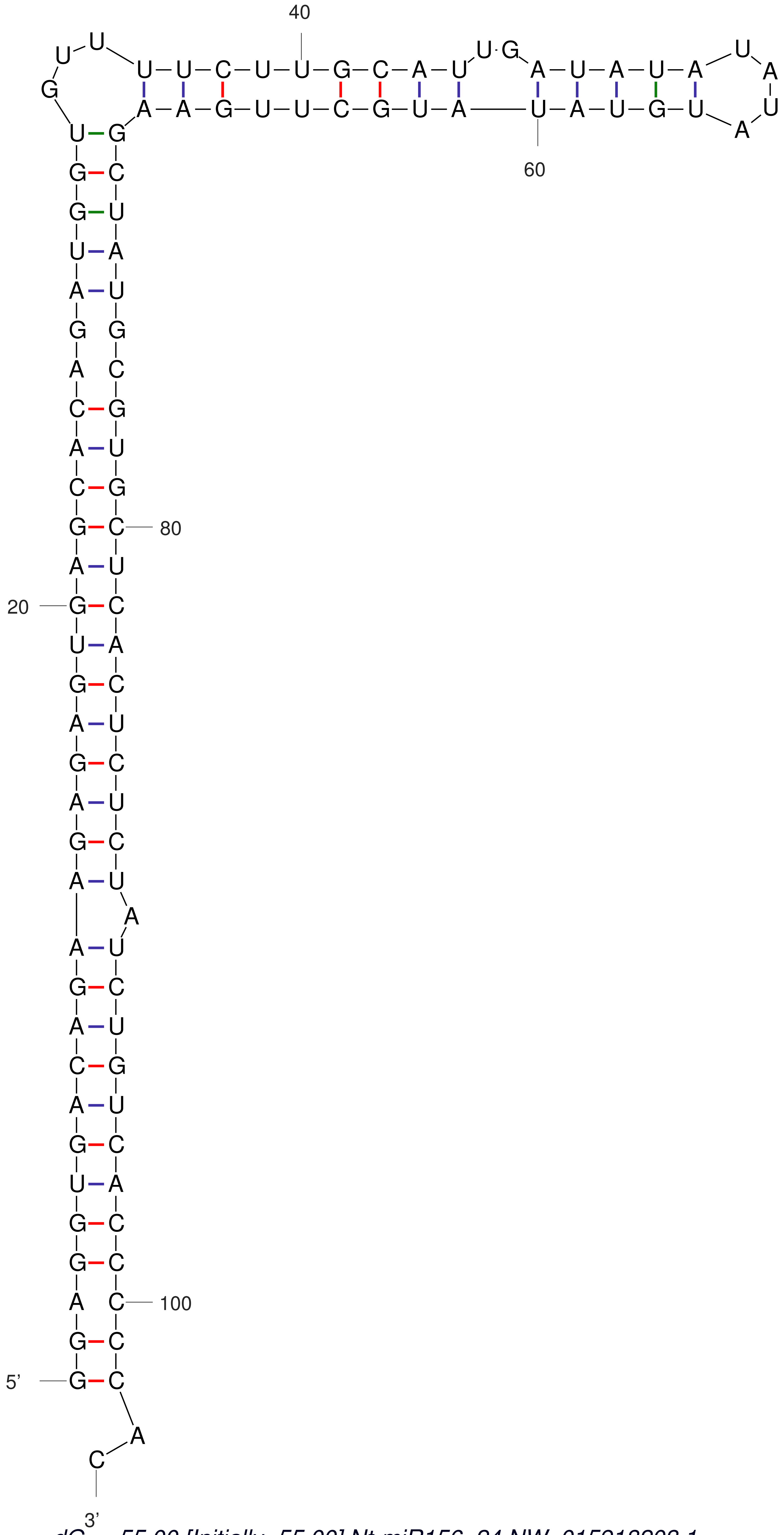

dG = -55.00 [Initially -55.00] Nt-miR156_24 NW_015918203 1

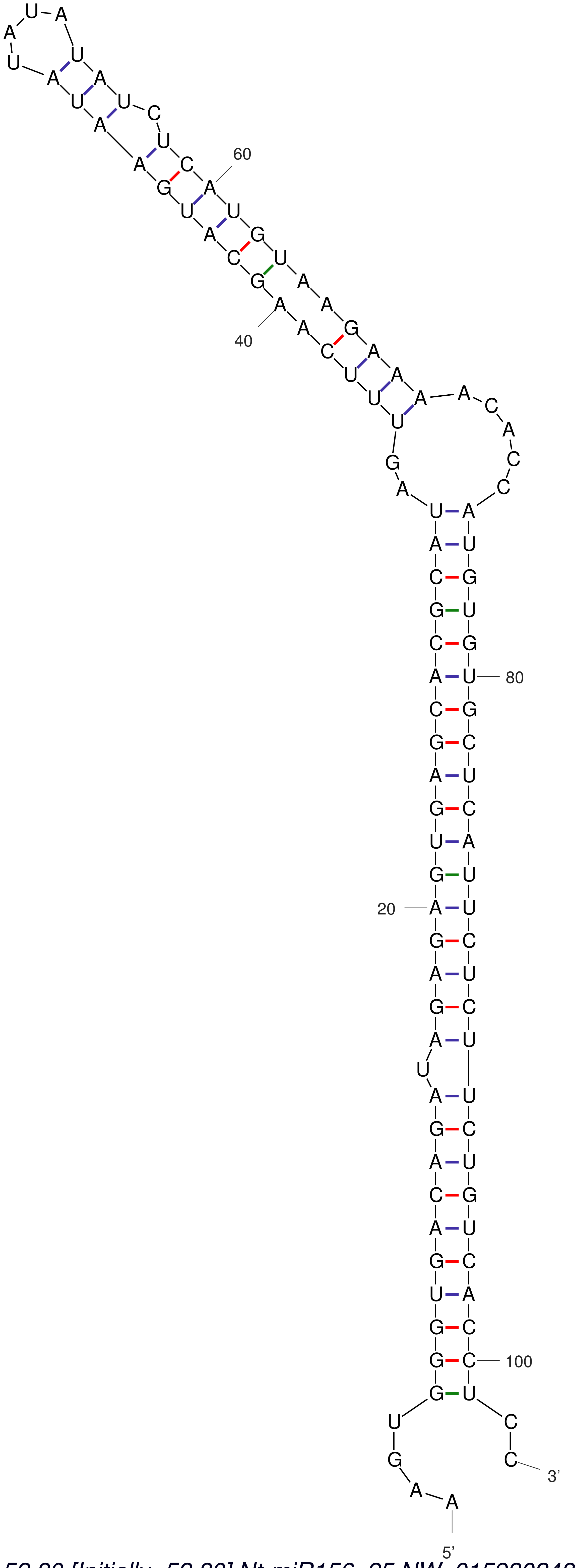

dG = -52.80 [Initially -52.80] Nt-miR156_25 NW_015930248 1

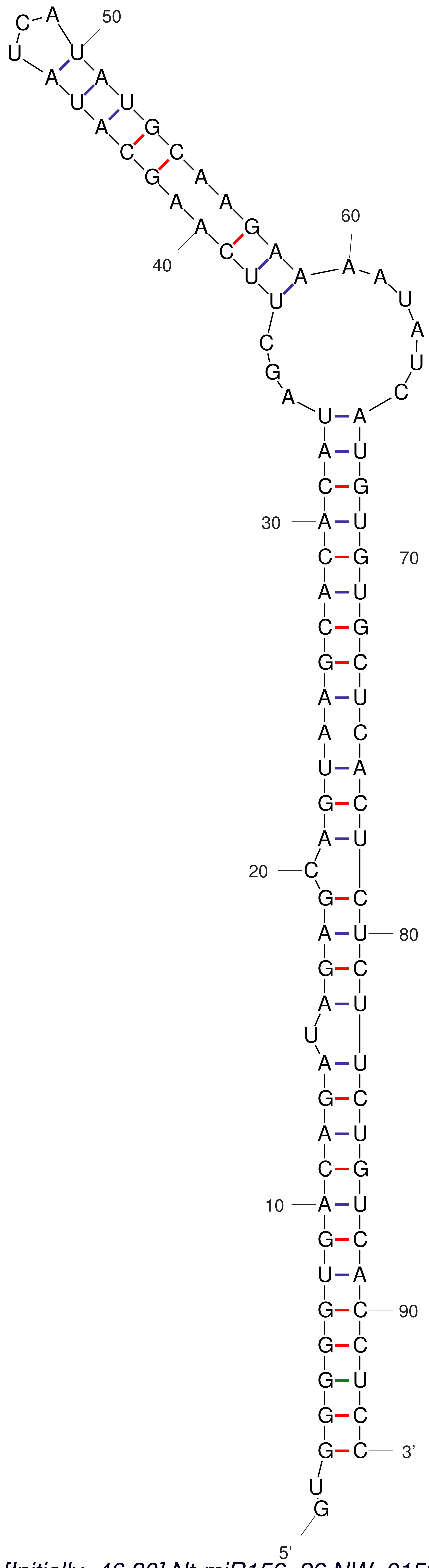

dG = -46.30 [Initially -46.30] Nt-miR156_26 NW_015950030 1

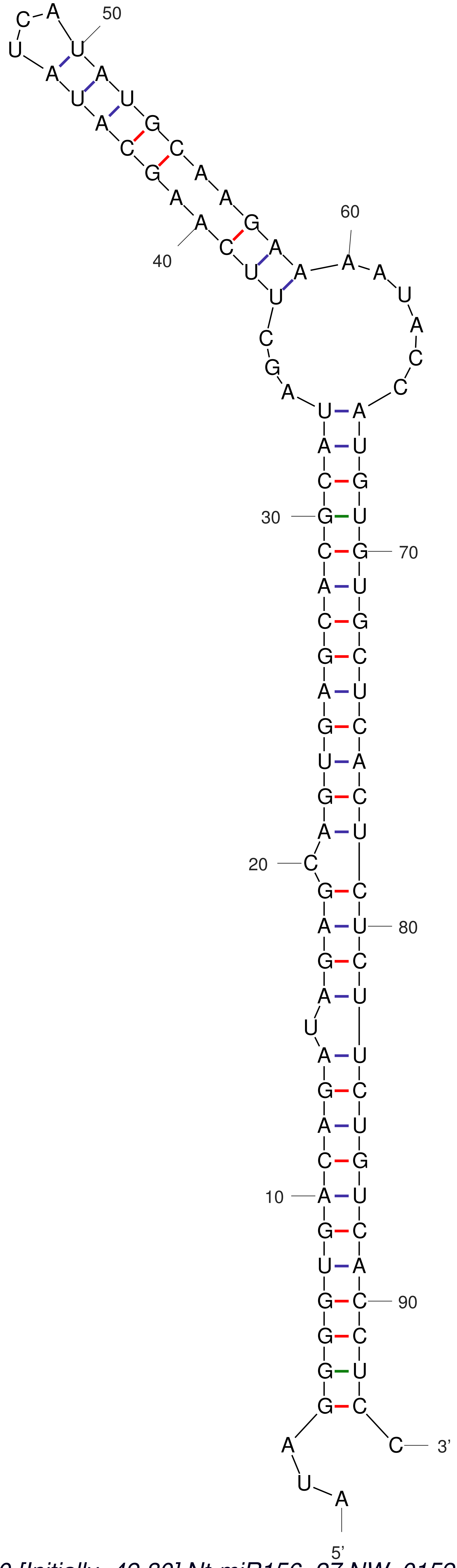

dG = -49.80 [Initially -49.80] Nt-miR156_27 NW_015950030 1

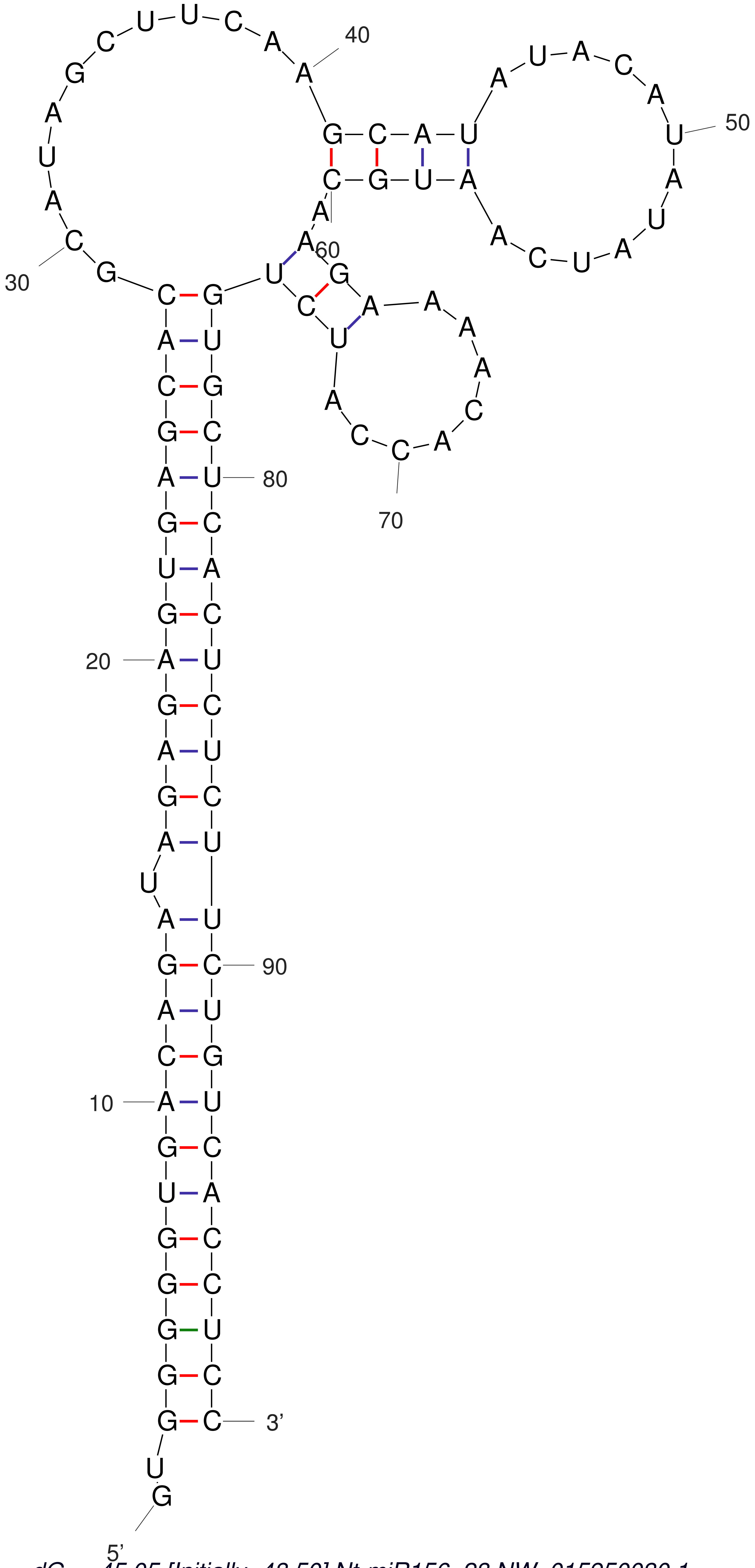

dG = -45.05 [Initially -48.50] Nt-miR156_28 NW_015950030 1

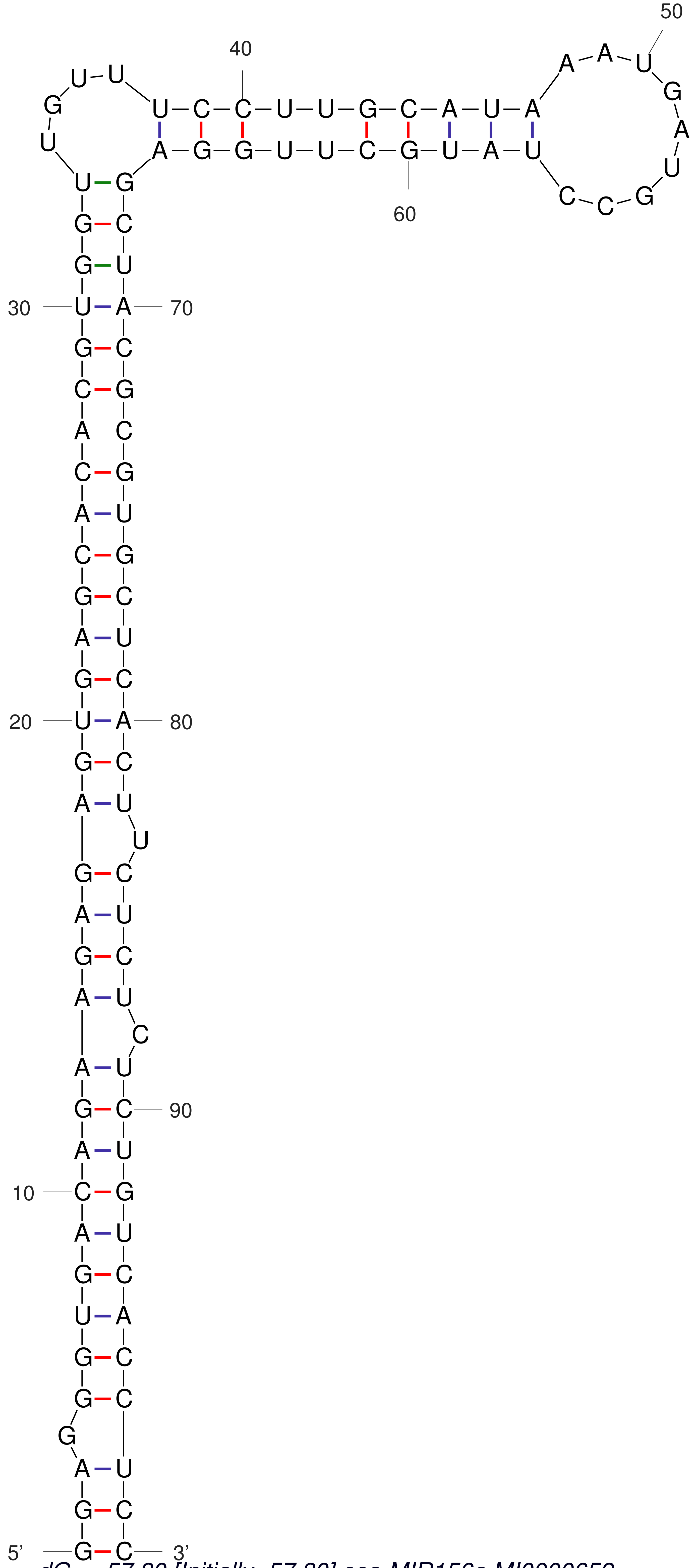

dG = -57.80 [Initially -57.80] osa-MIR156a MI0000653

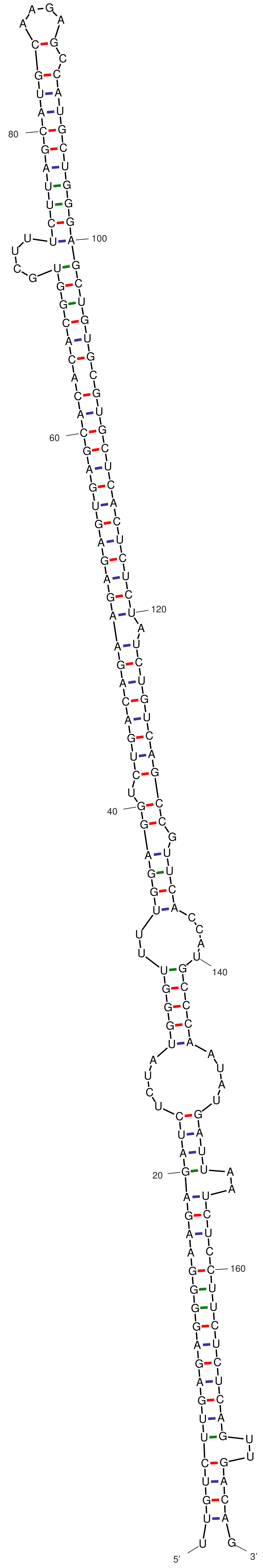

dG = -93.90 [Initially -93.90] osa-MIR156b MI0000654

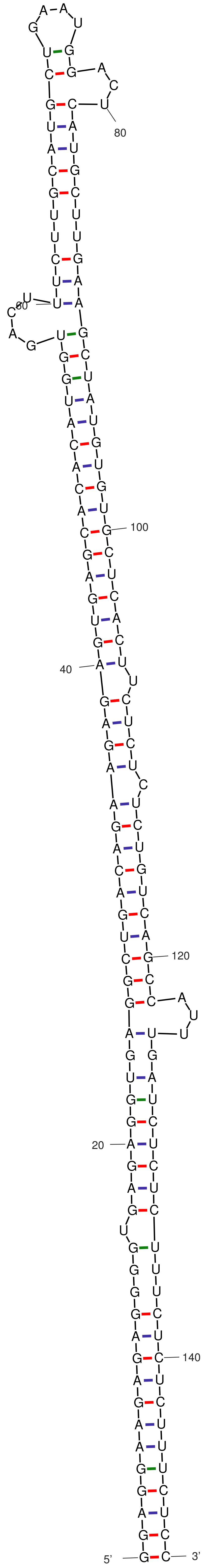

dG = -90.90 [Initially -90.90] osa-MIR156c MI0000655

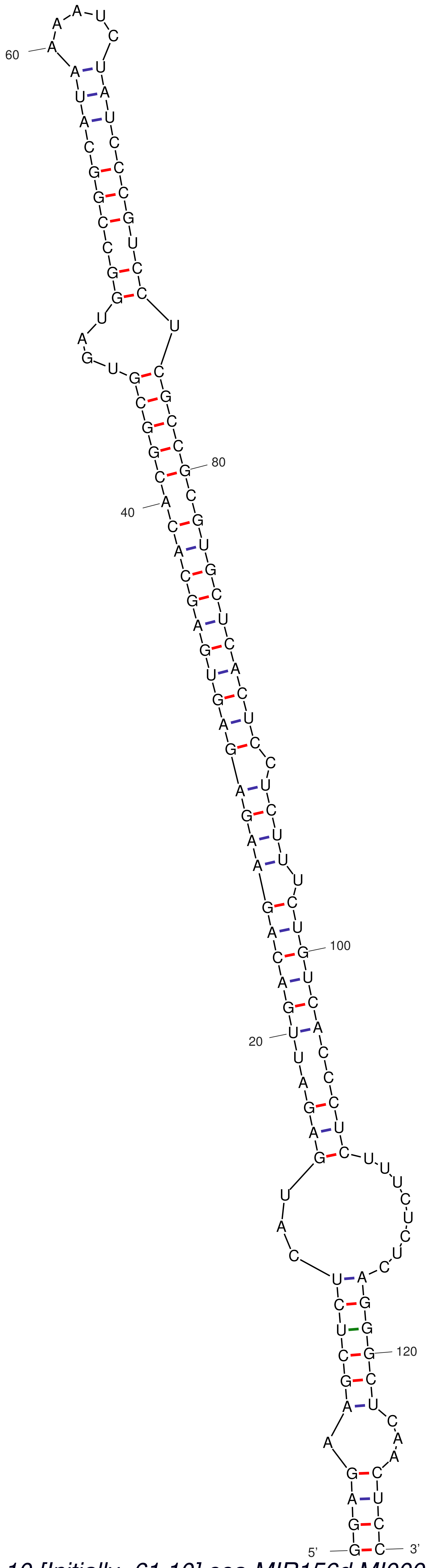

dG = -61.10 [Initially -61.10] osa-MIR156d MI0000656

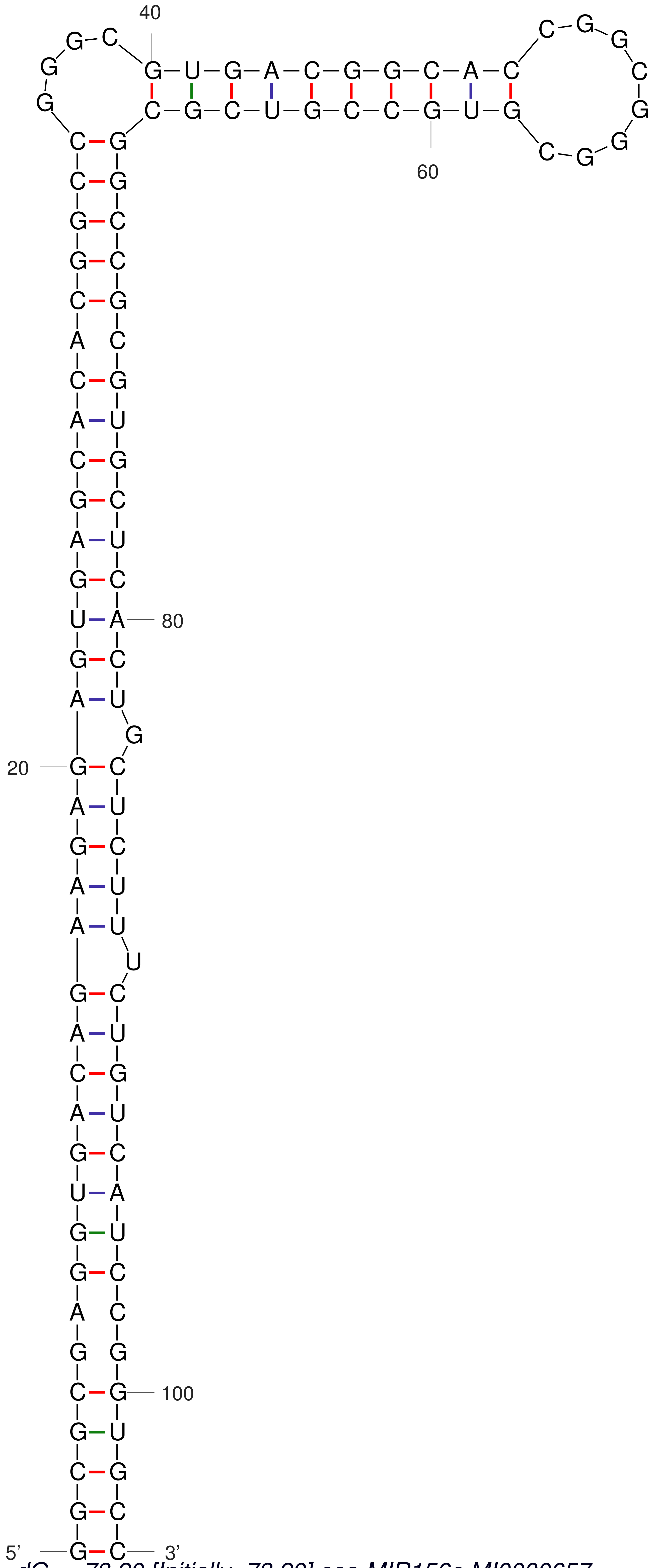

dG = -73.20 [Initially -73.20] osa-MIR156e MI0000657

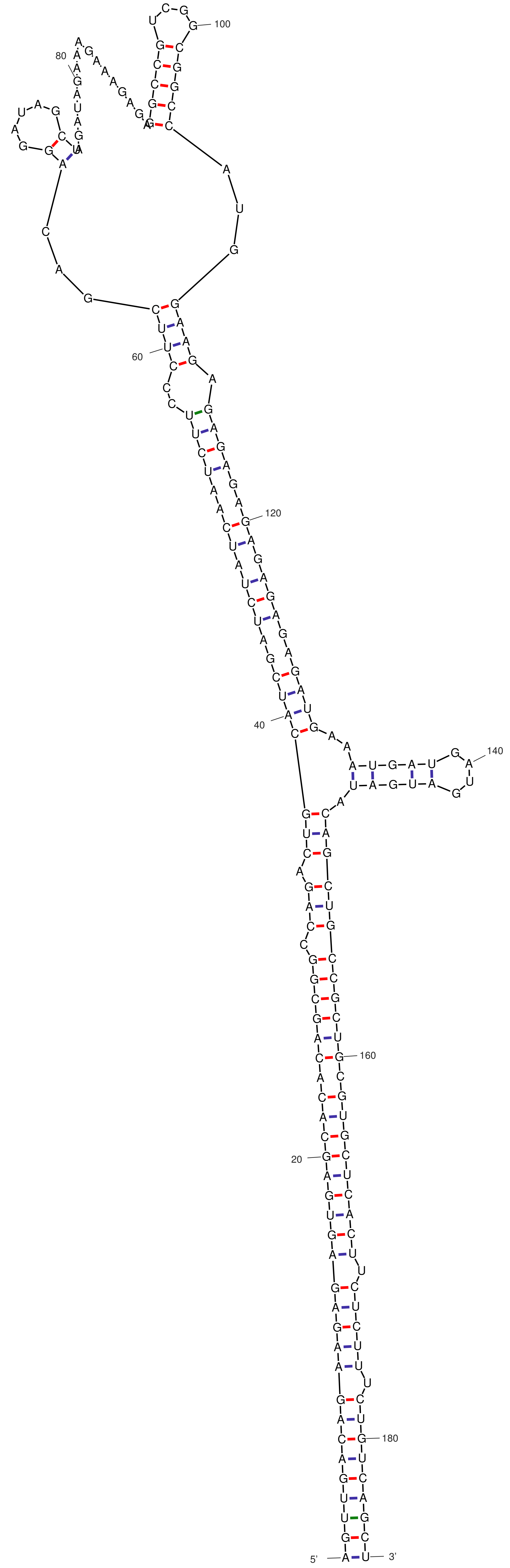

dG = -74.62 [Initially -80.20] osa-MIR156f MI0000658

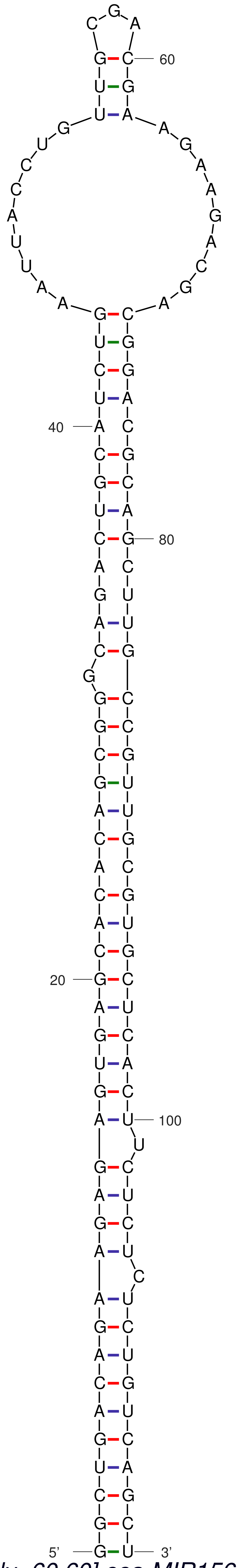

dG = -61.10 [Initially -60.60] osa-MIR156g MI0000659

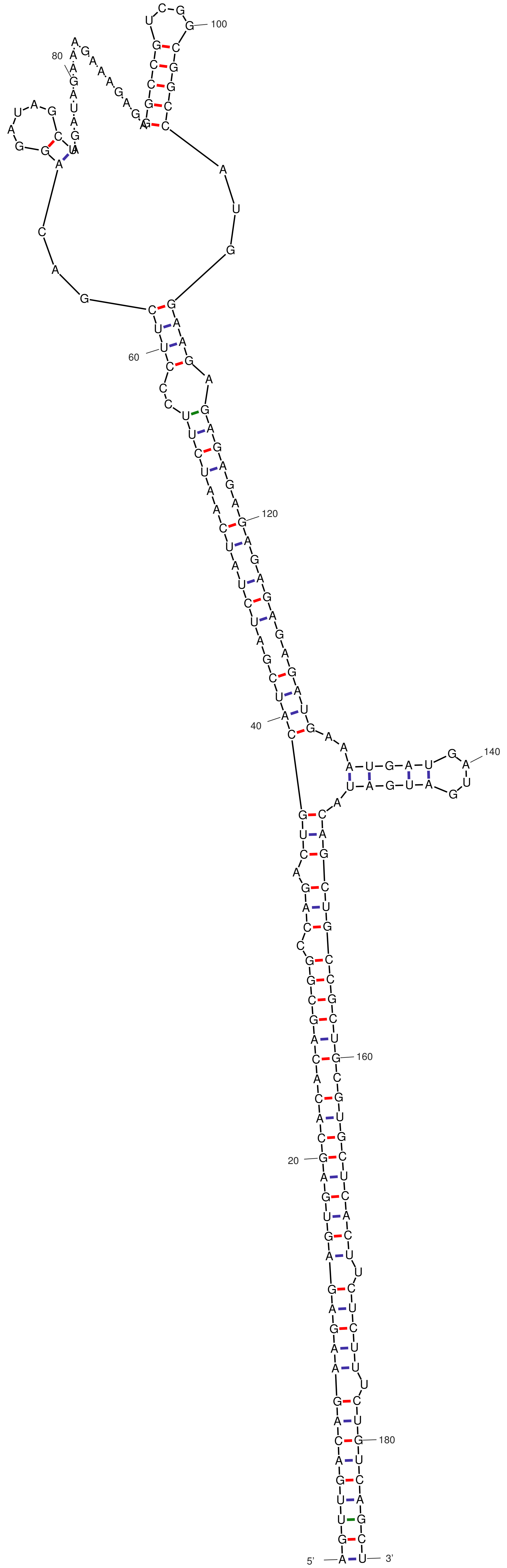

dG = -74.62 [Initially -80.20] osa-MIR156h MI0000660

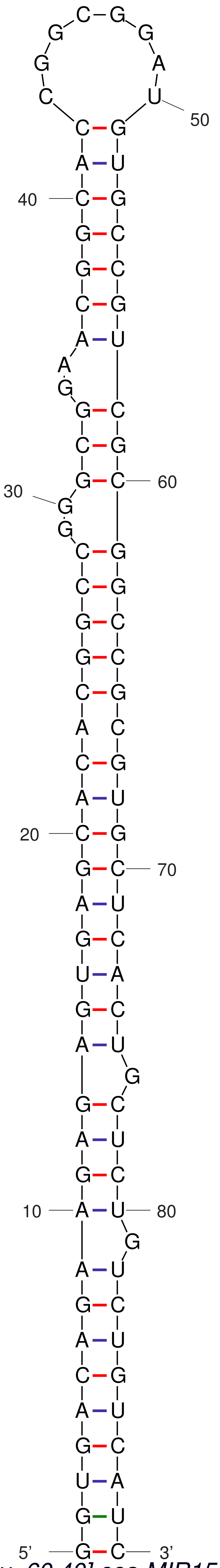

dG = -60.40 [Initially -60.40] osa-MIR156i MI0000661

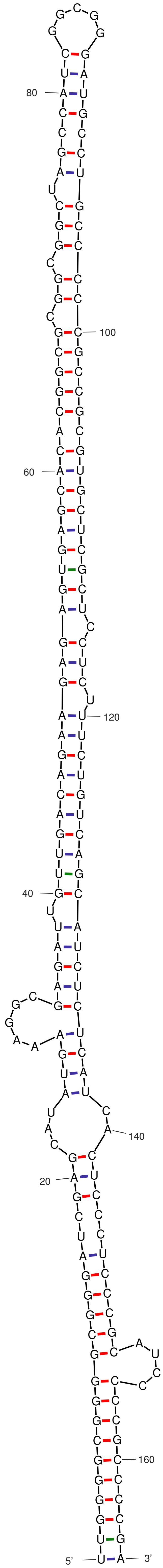

dG = -102.60 [Initially -102.10] osa-MIR156j MI0000662

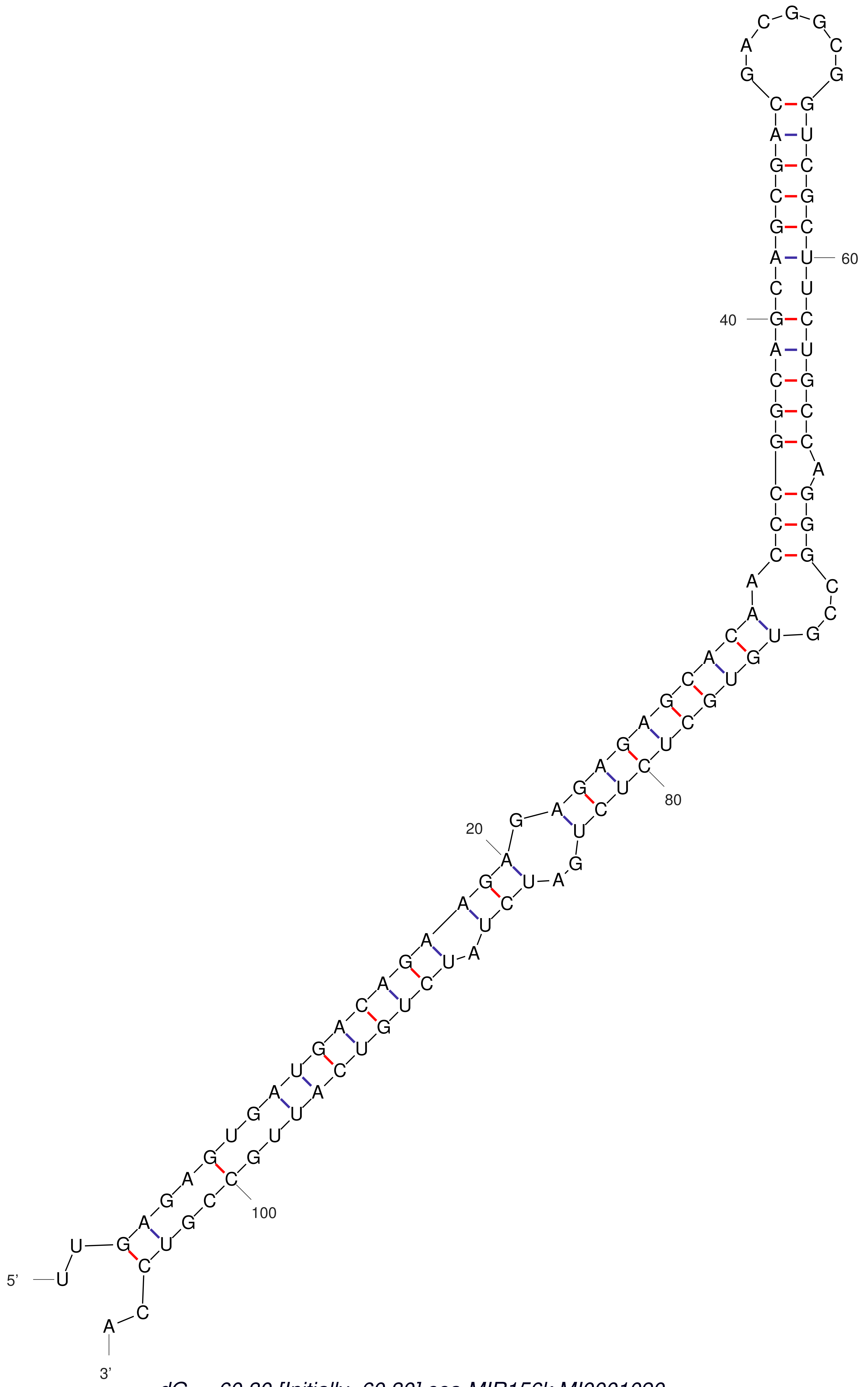

dG = -60.30 [Initially -60.30] osa-MIR156k MI0001090

Output of sir_graph (©)
mfold_util 4.7

Created Tue May 21 03:24:33 2019

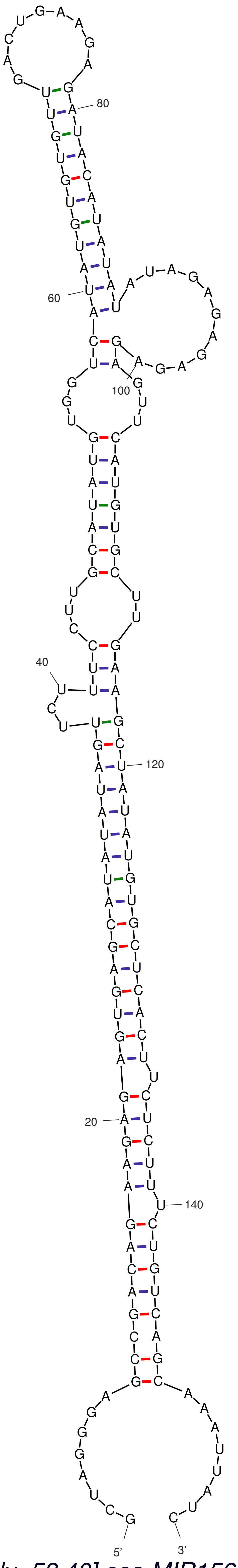

dG = -58.40 [Initially -58.40] osa-MIR156l MI0001091

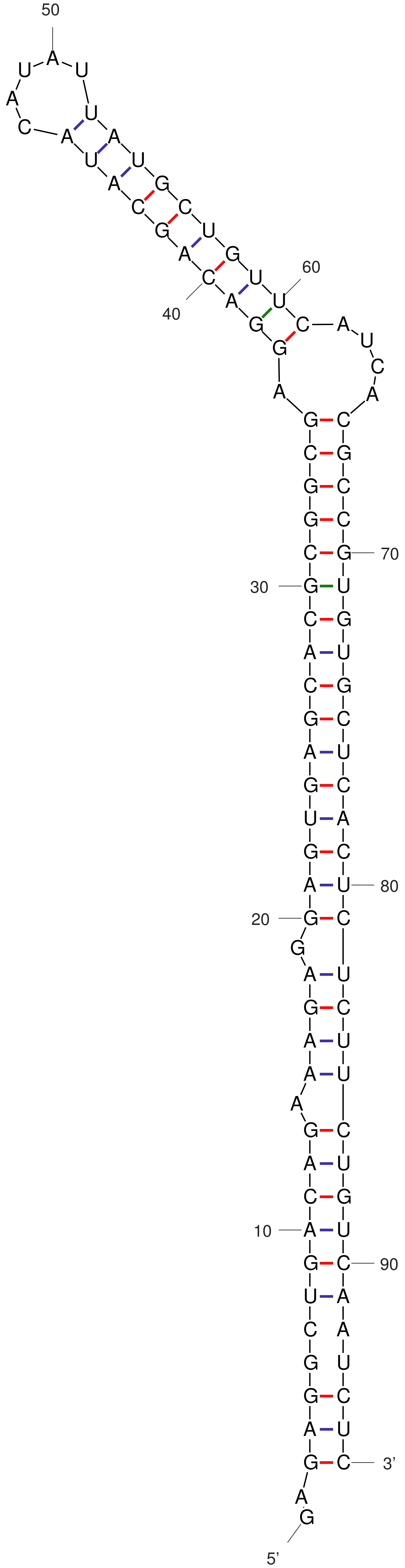

dG = -63.50 [Initially -63.50] Ph-miR156_1 NC_038042 1

Output of sir_graph (©)
mfold_util 4.7

Created Tue May 21 03:21:17 2019

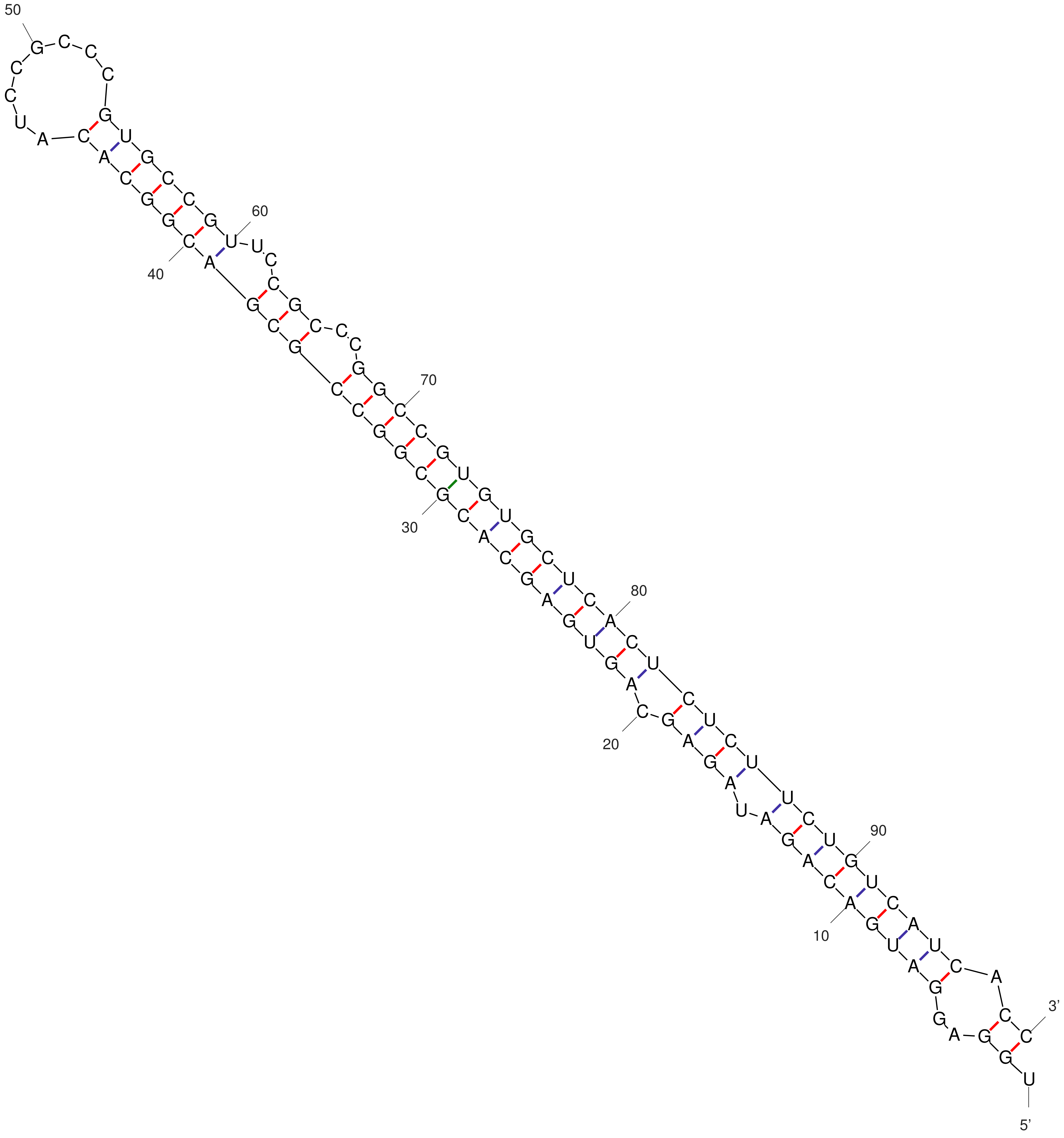

dG = -67.40 [Initially -67.40] Ph-miR156_2 NC_038042 1

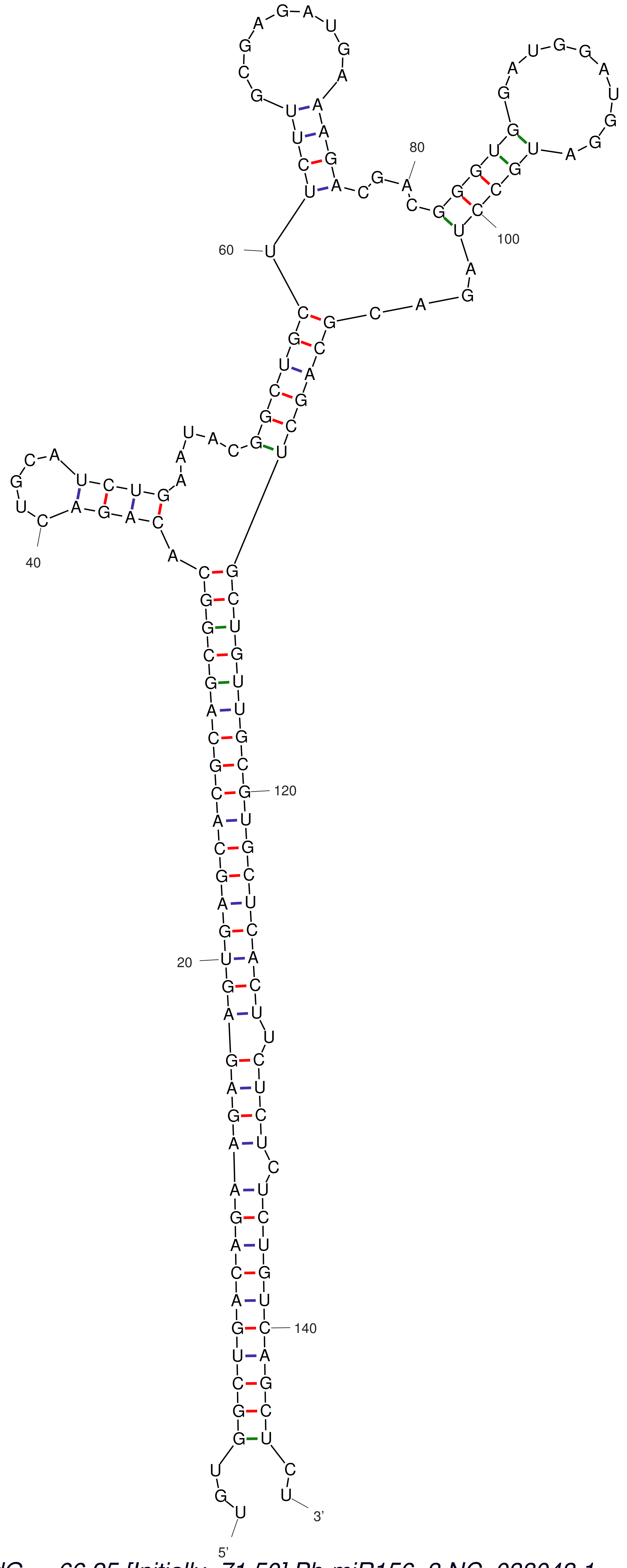

dG = -66.25 [Initially -71.50] Ph-miR156_3 NC_038043 1

Output of sir_graph (©)
mfold_util 4.7

Created Tue May 21 03:21:36 2019

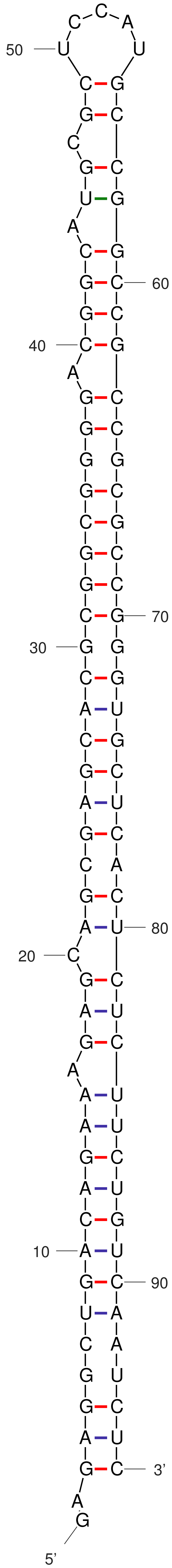

dG = -61.70 [Initially -61.70] Ph-miR156_4 NC_038045 1

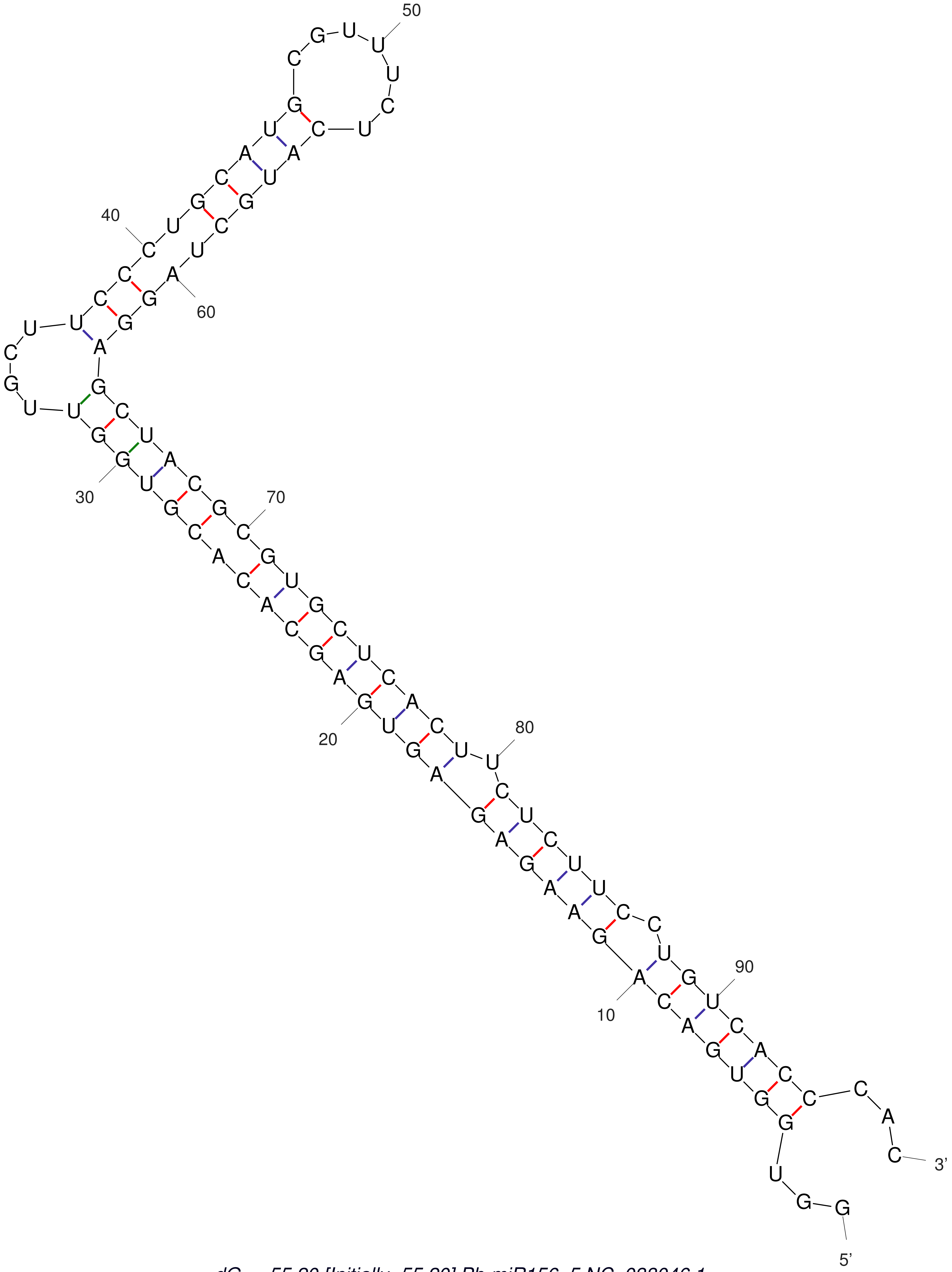

dG = -55.20 [Initially -55.20] Ph-miR156_5 NC_038046 1

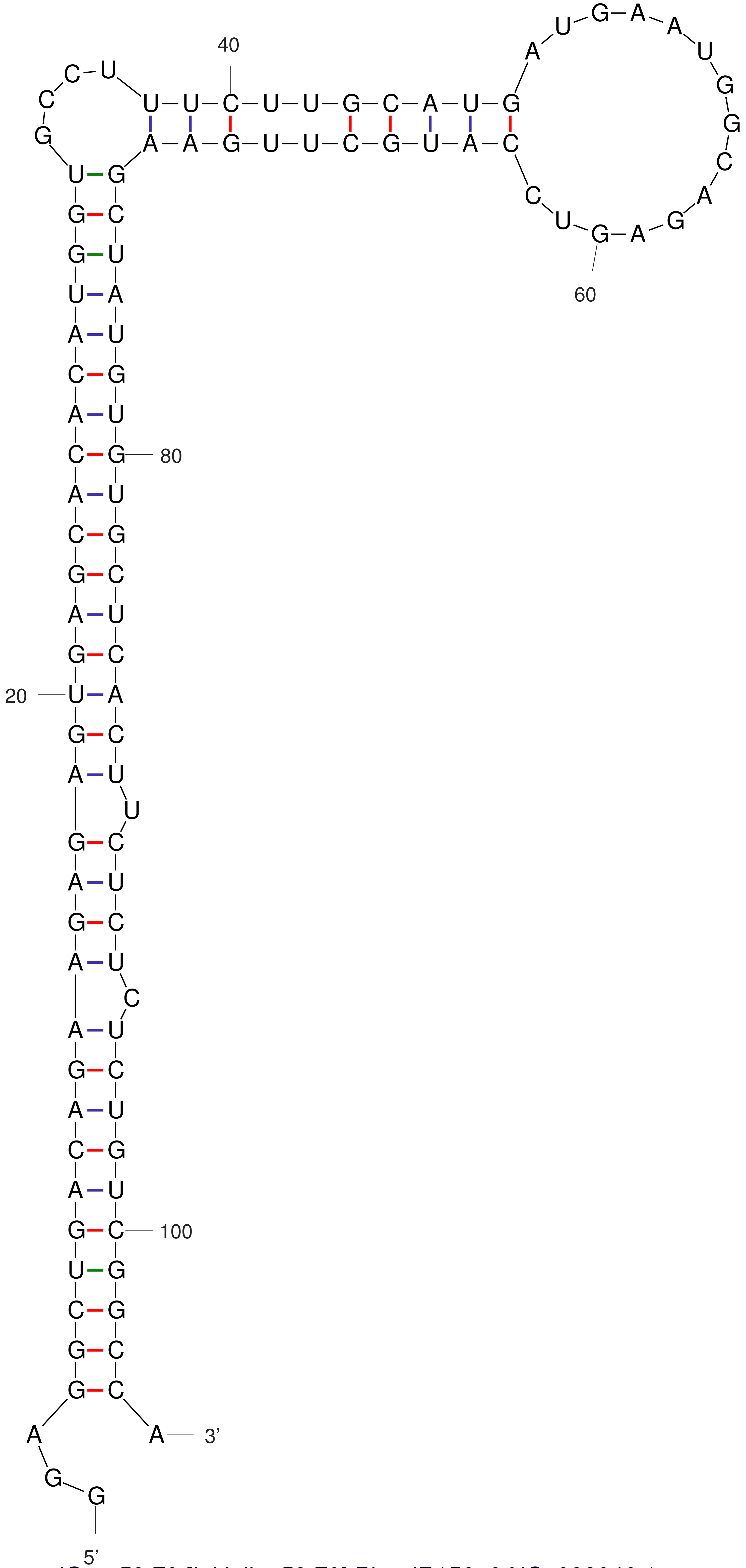

dG = -59.70 [Initially -59.70] Ph-miR156_6 NC_038046 1

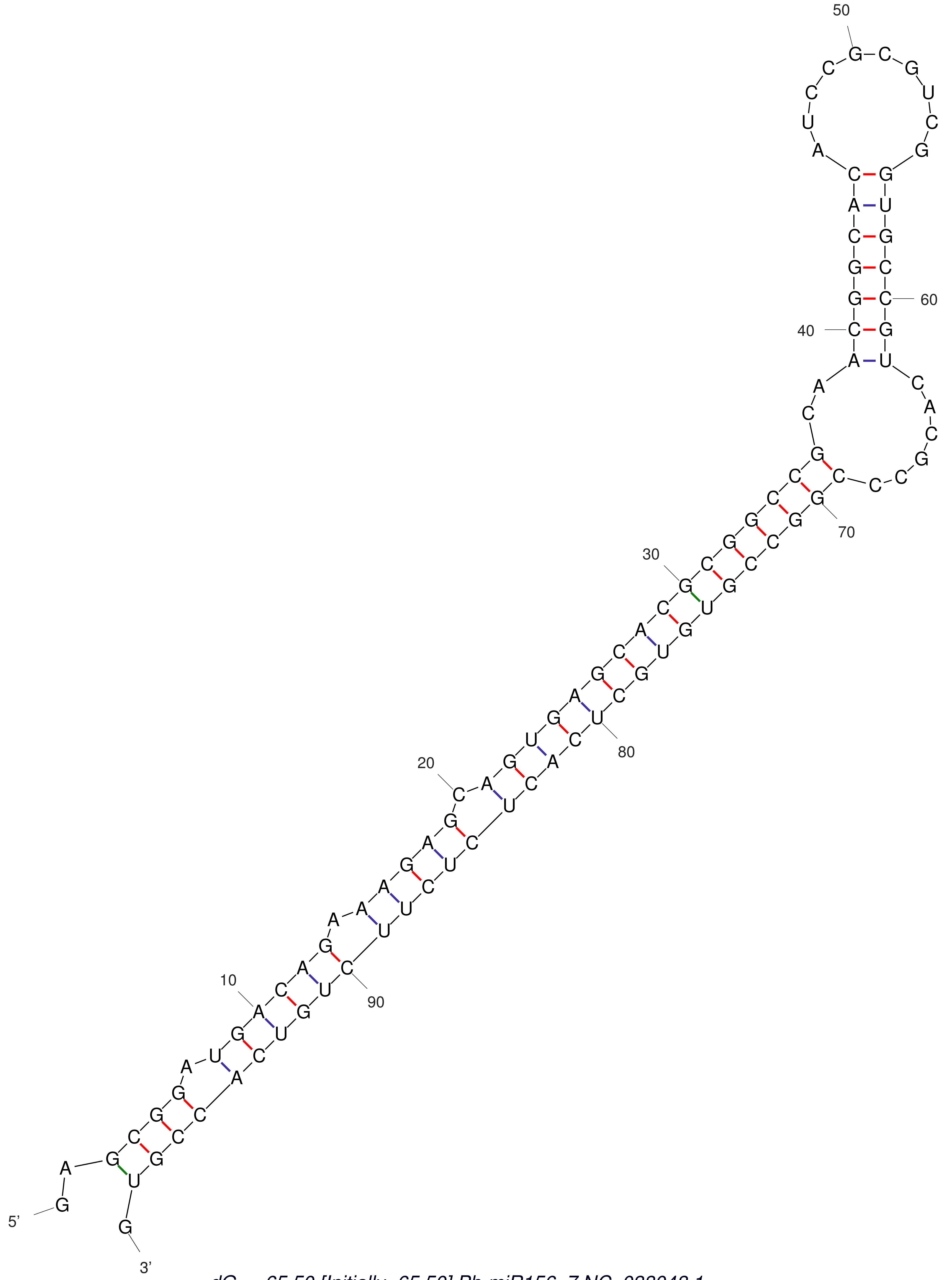

dG = -65.50 [Initially -65.50] Ph-miR156_7 NC_038048 1

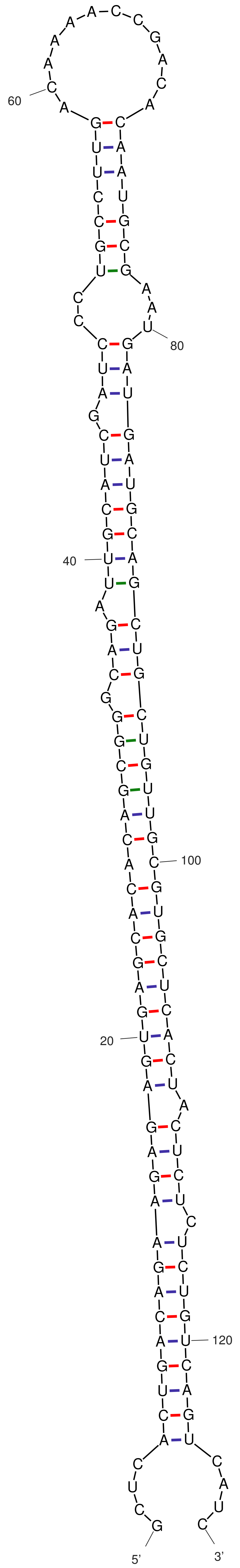

dG = -61.60 [Initially -61.60] Ph-miR156_8 NC_038049 1

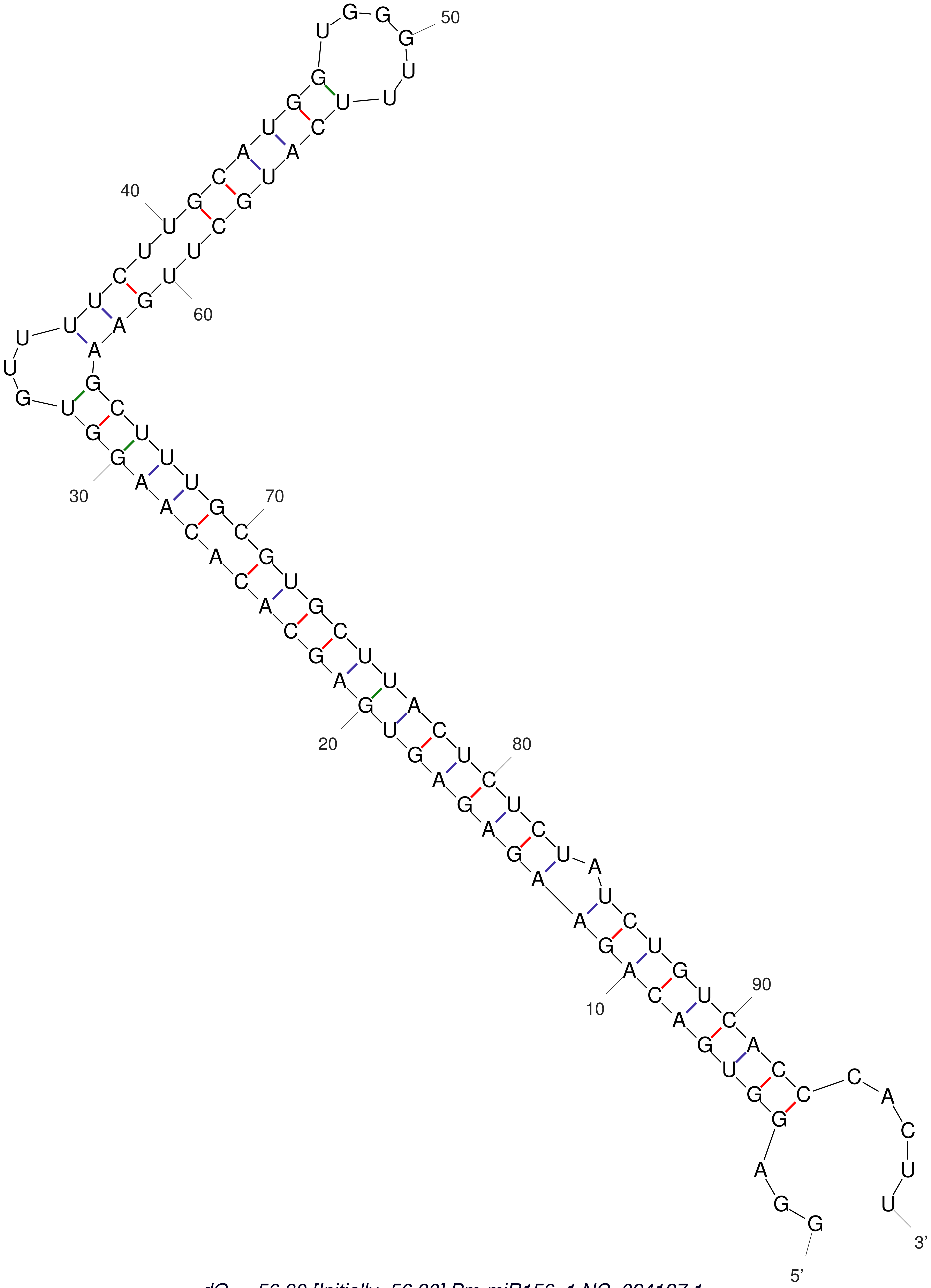

dG = -56.30 [Initially -56.30] Pm-miR156_1 NC_024127 1

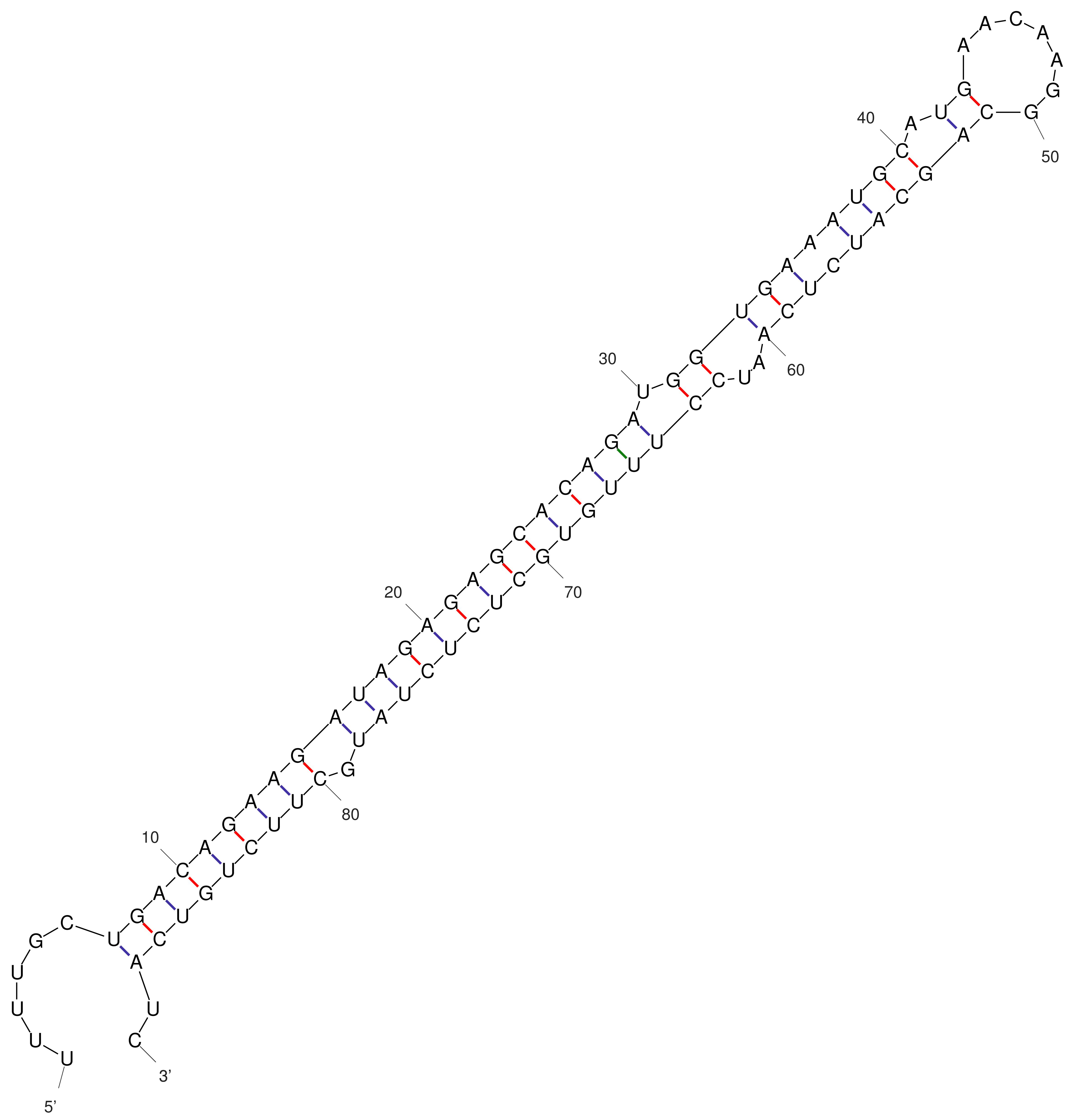

dG = -43.80 [Initially -43.80] Pm-miR156_2 NC_024129 1

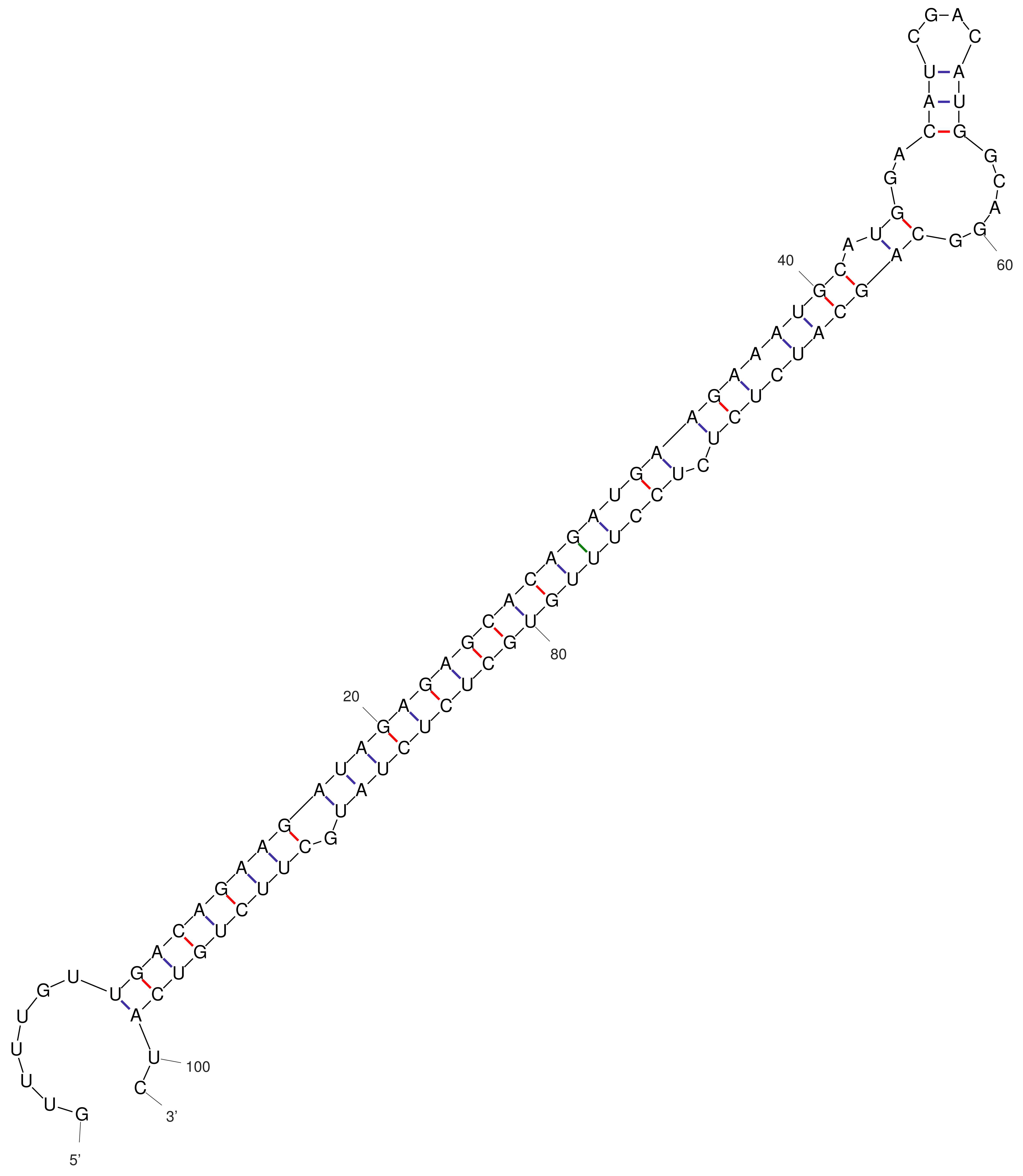

dG = -43.70 [Initially -43.70] Pm-miR156_3 NC_024129 1

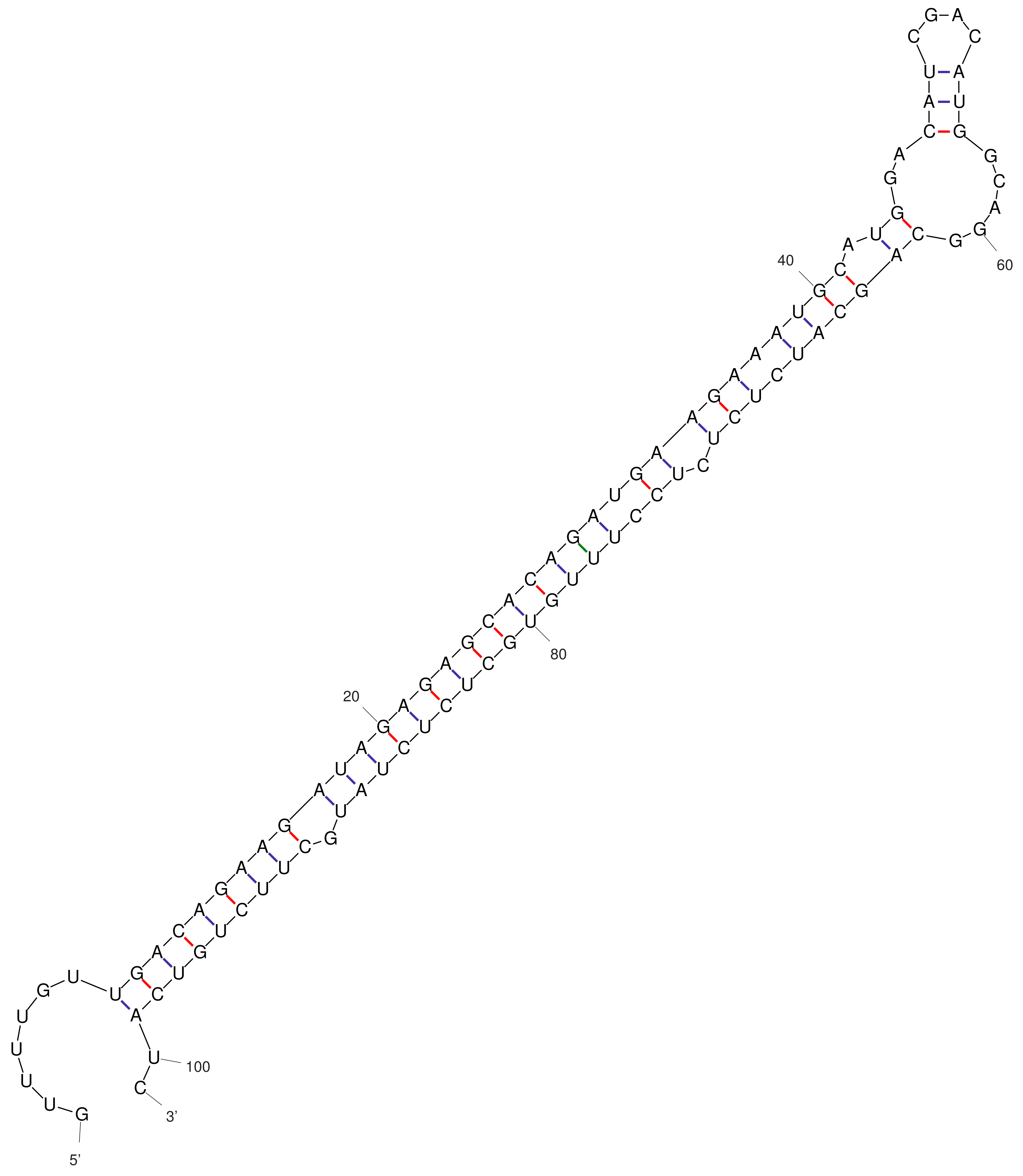

dG = -43.70 [Initially -43.70] Pm-miR156_4 NC_024129 1

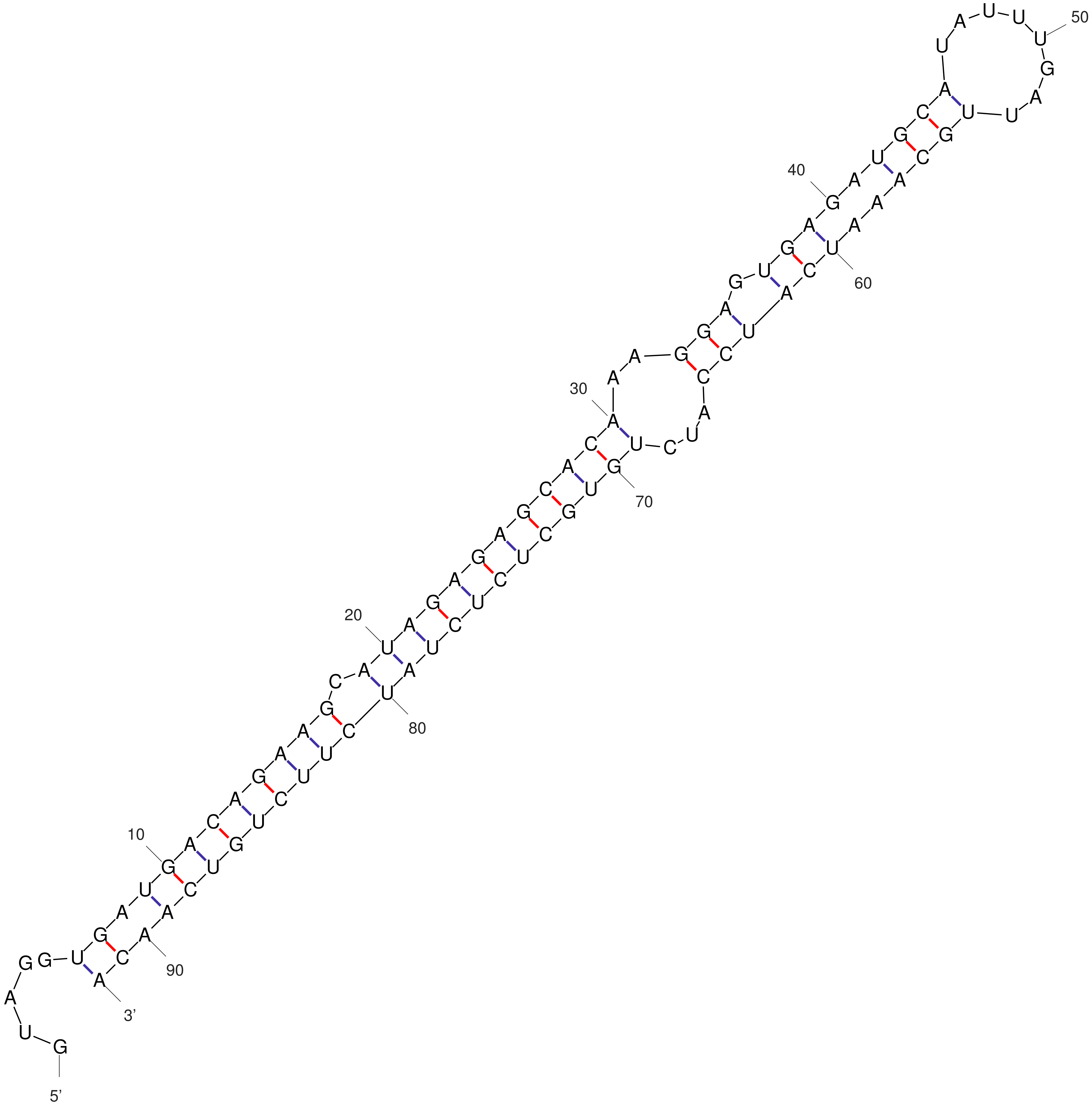

dG = -44.80 [Initially -45.00] Pm-miR156_5 NC_024132 1

Output of sir_graph (©)
mfold_util 4.7

Created Tue May 21 00:16:14 2019

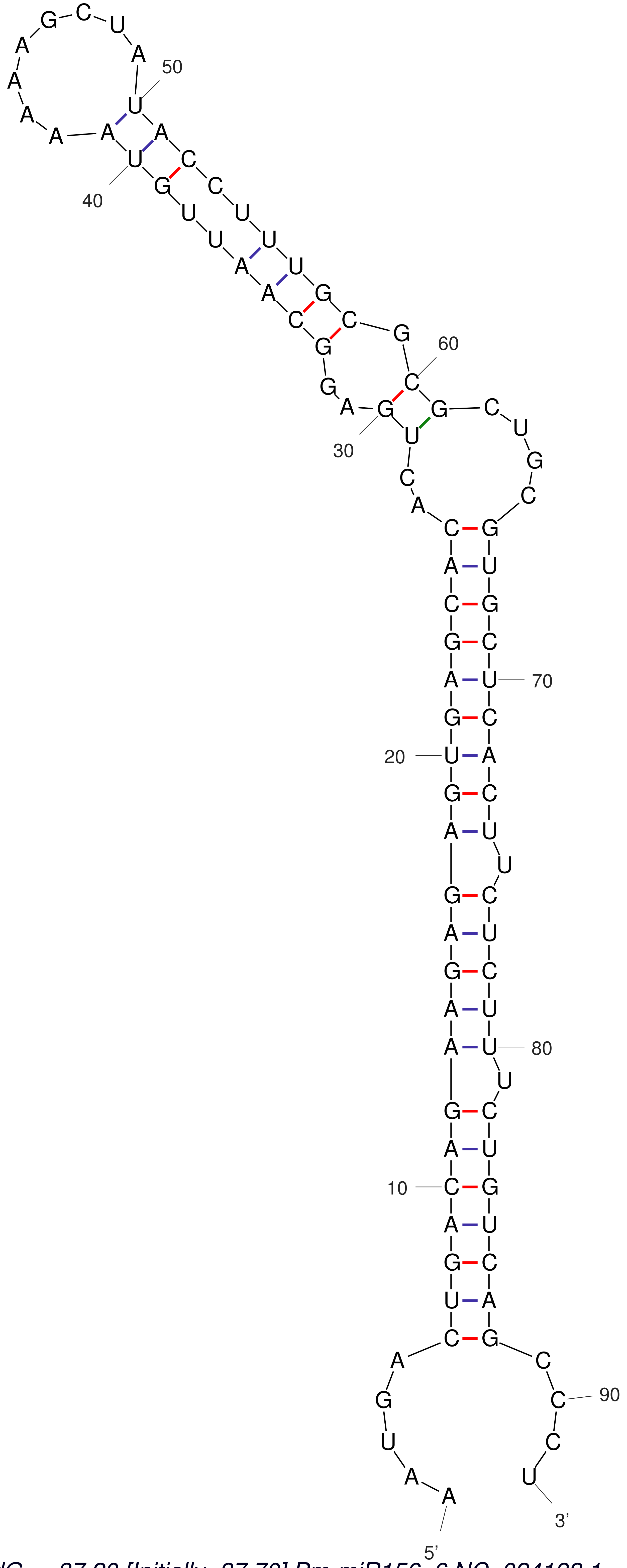

dG = -37.20 [Initially -37.70] Pm-miR156_6 NC_024133 1

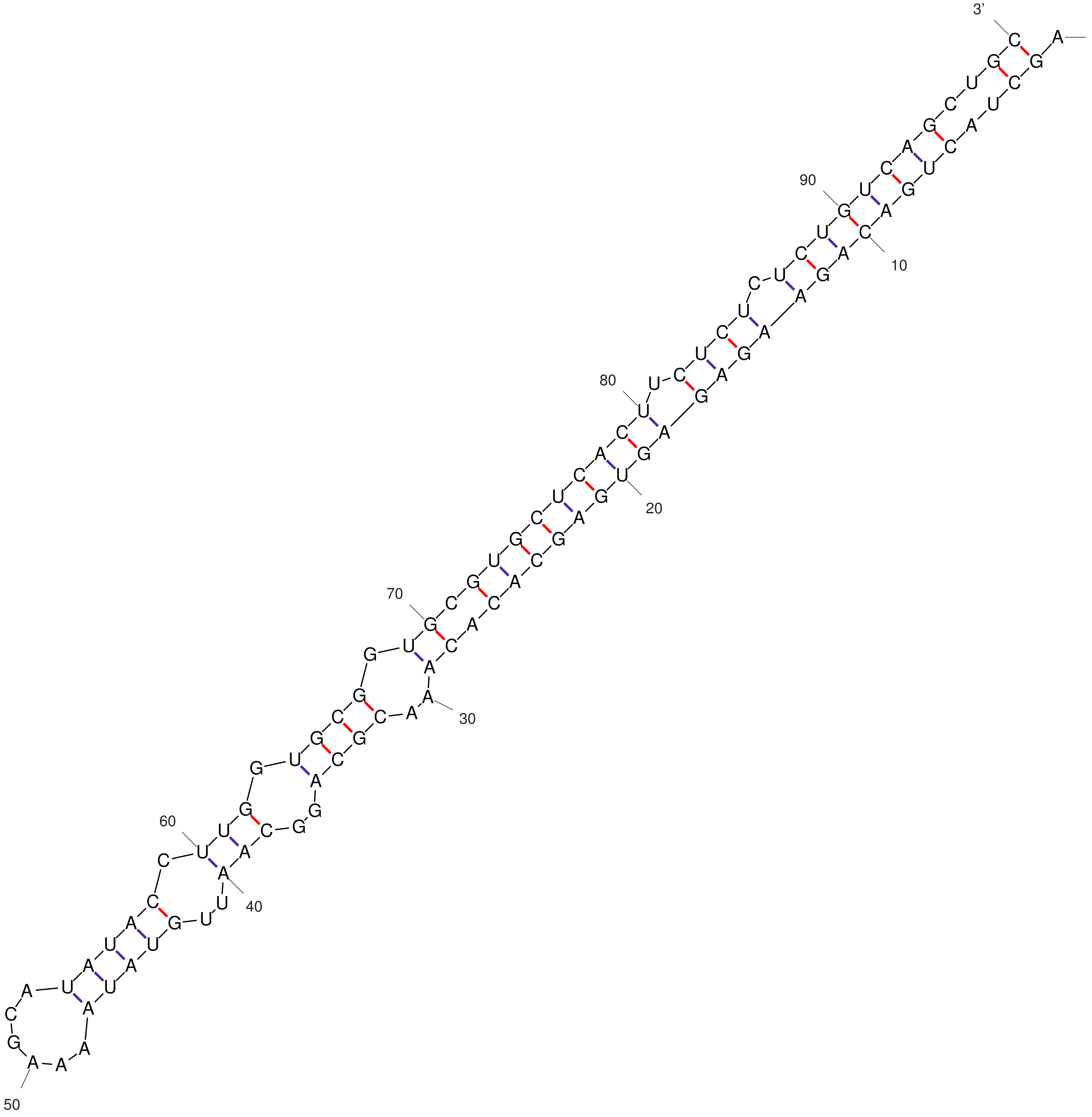

dG = -45.30 [Initially -45.30] Pm-miR156_7 NC_024133 1

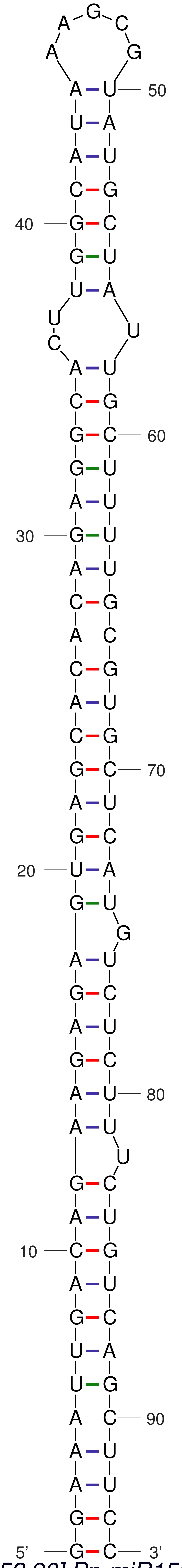

dG = -52.90 [Initially -52.90] Pp-miR156_2 NC_034009 1

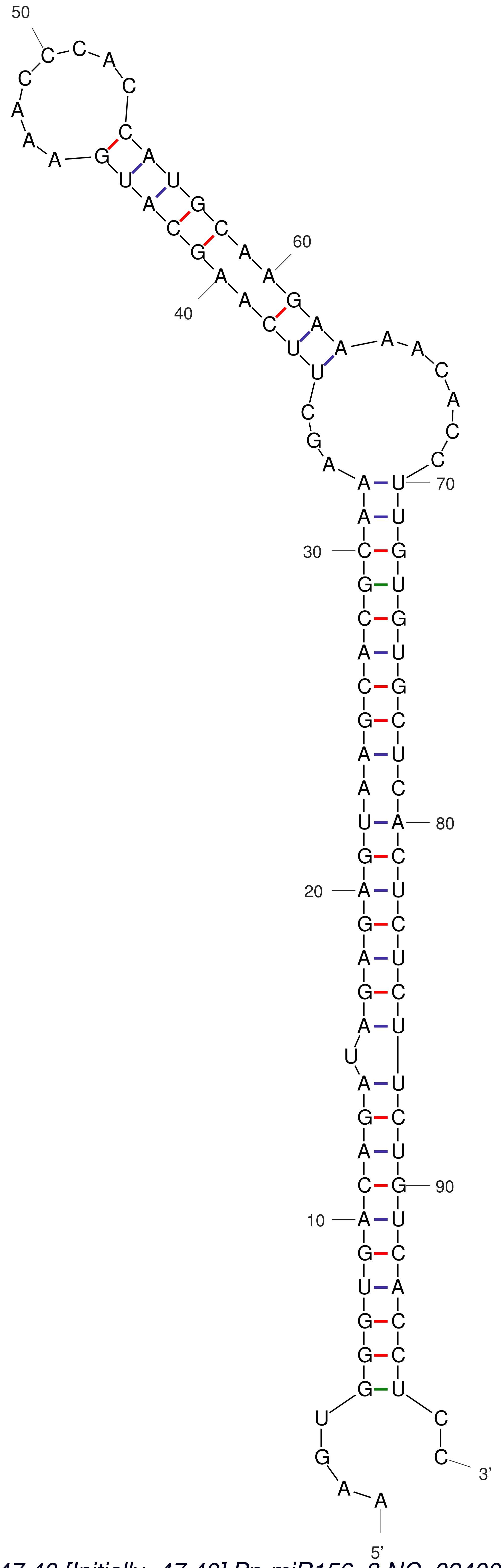

dG = -47.40 [Initially -47.40] Pp-miR156_3 NC_034009 1

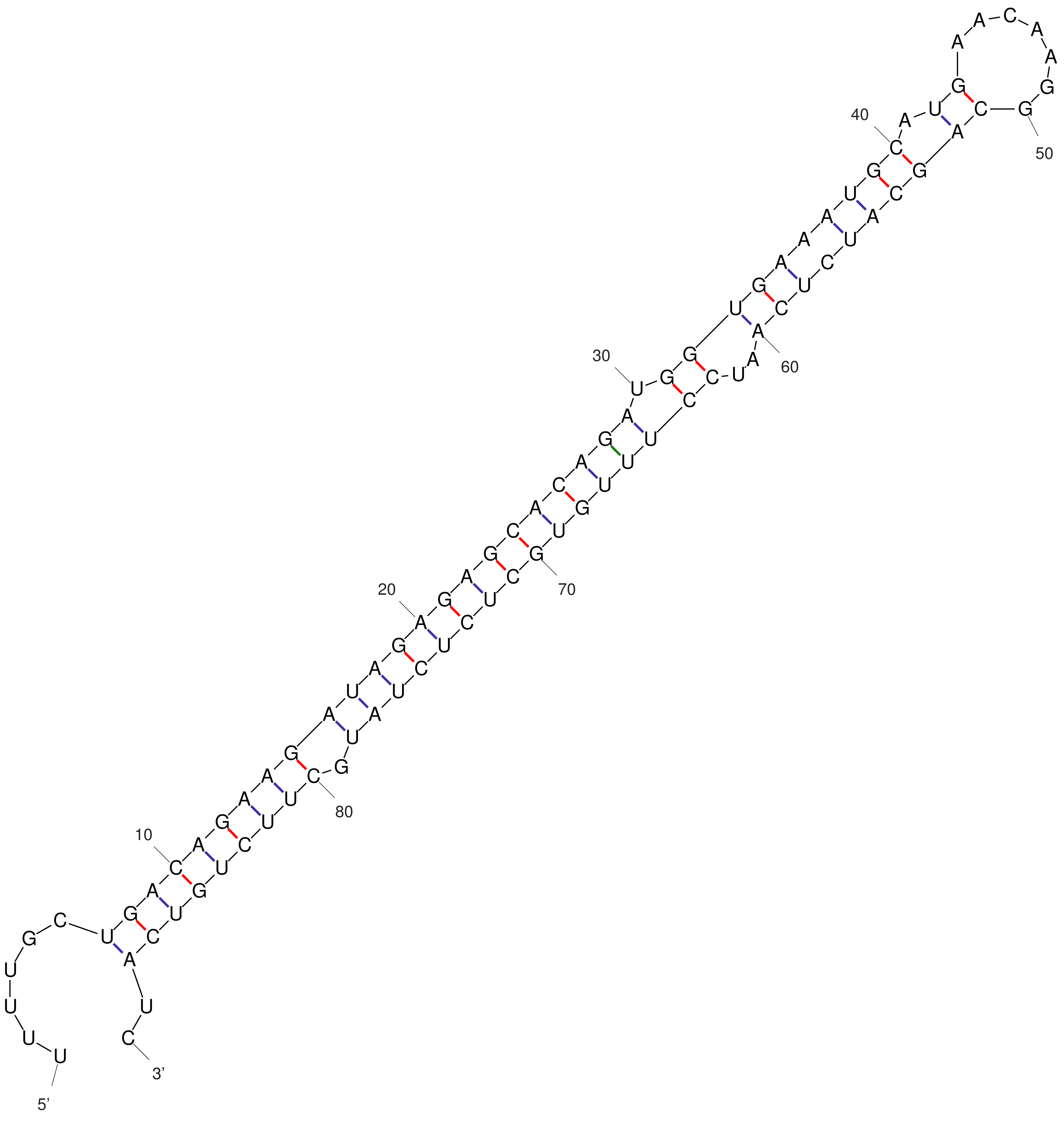

dG = -43.80 [Initially -43.80] Pp-miR156_4 NC_034011 1

Output of sir_graph (©)
mfold_util 4.7

Created Mon Jul 22 08:33:28 2019

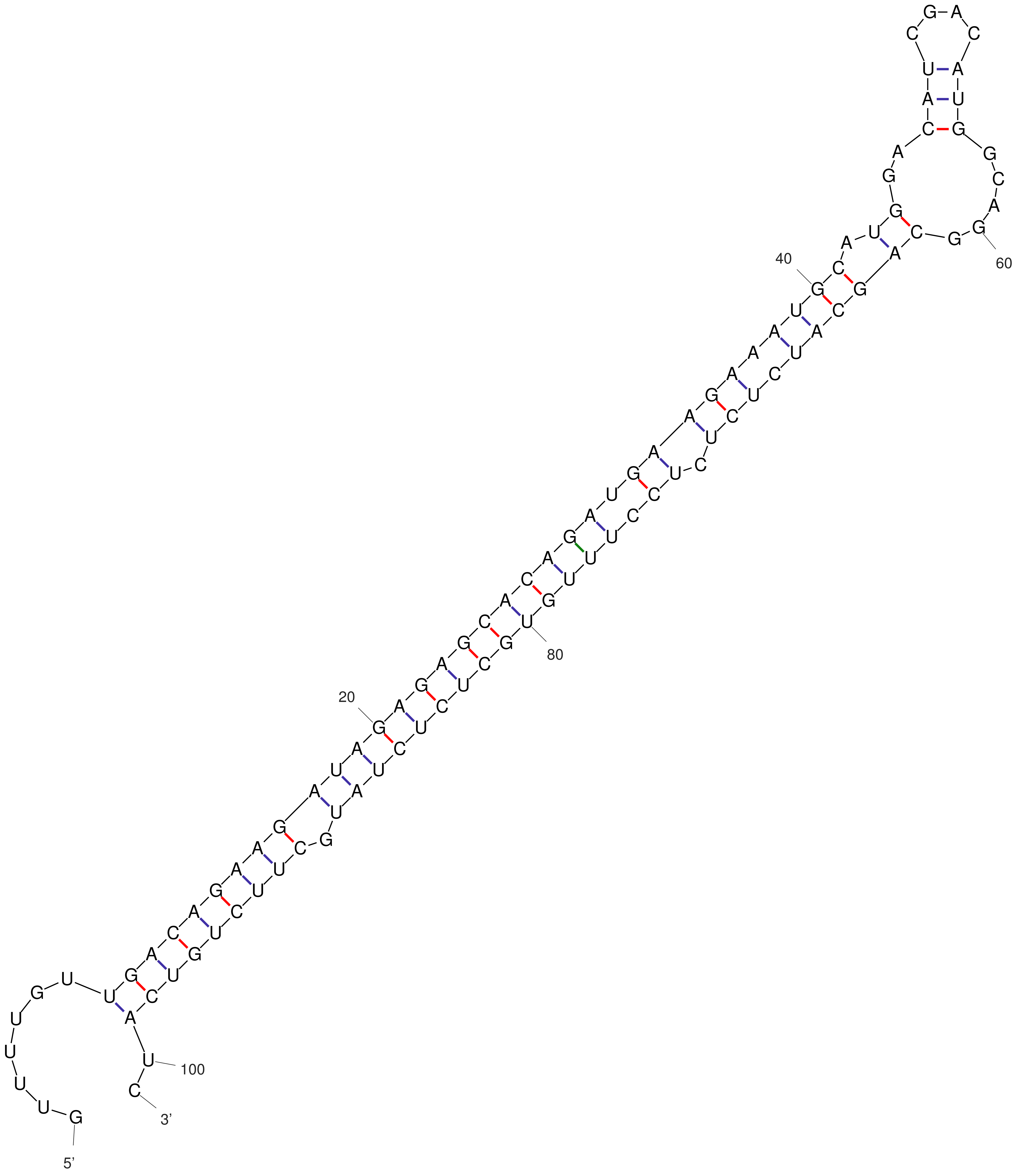

dG = -43.70 [Initially -43.70] Pp-miR156_5 NC_034011 1

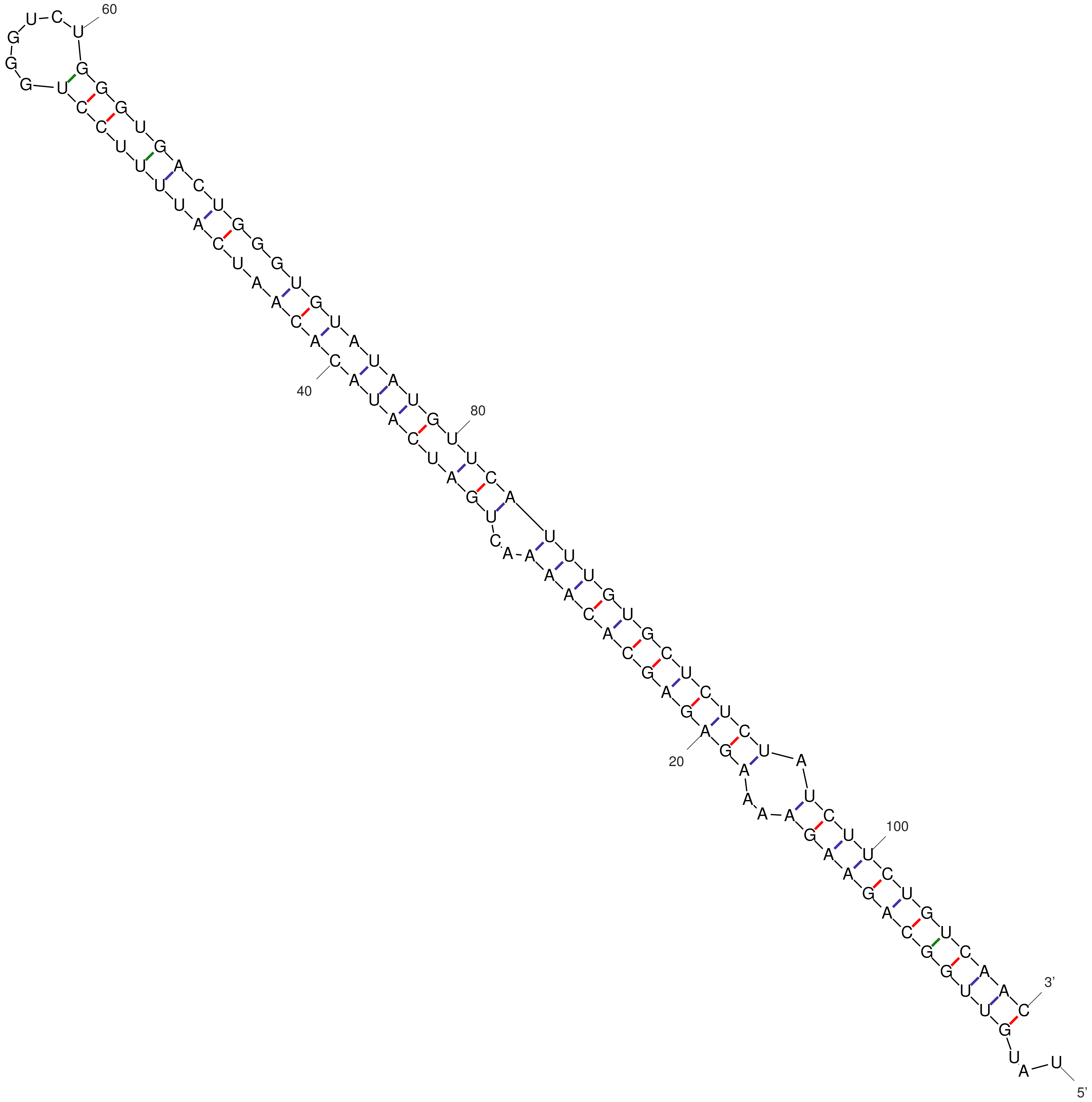

dG = -48.20 [Initially -48.20] Pp-miR156_6 NC_034011 1

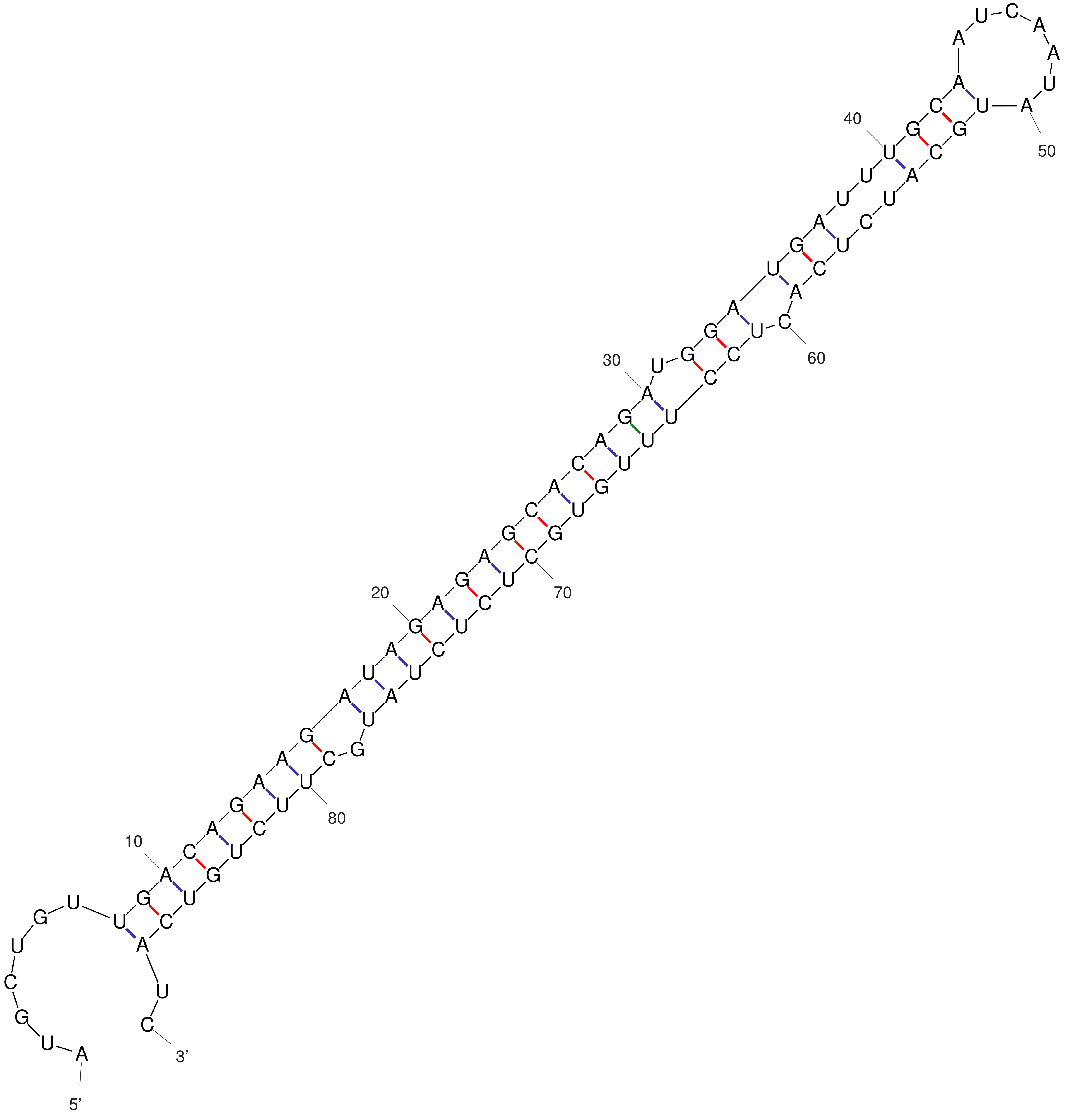

dG = -46.80 [Initially -46.80] Pp-miR156_7 NC_034013 1

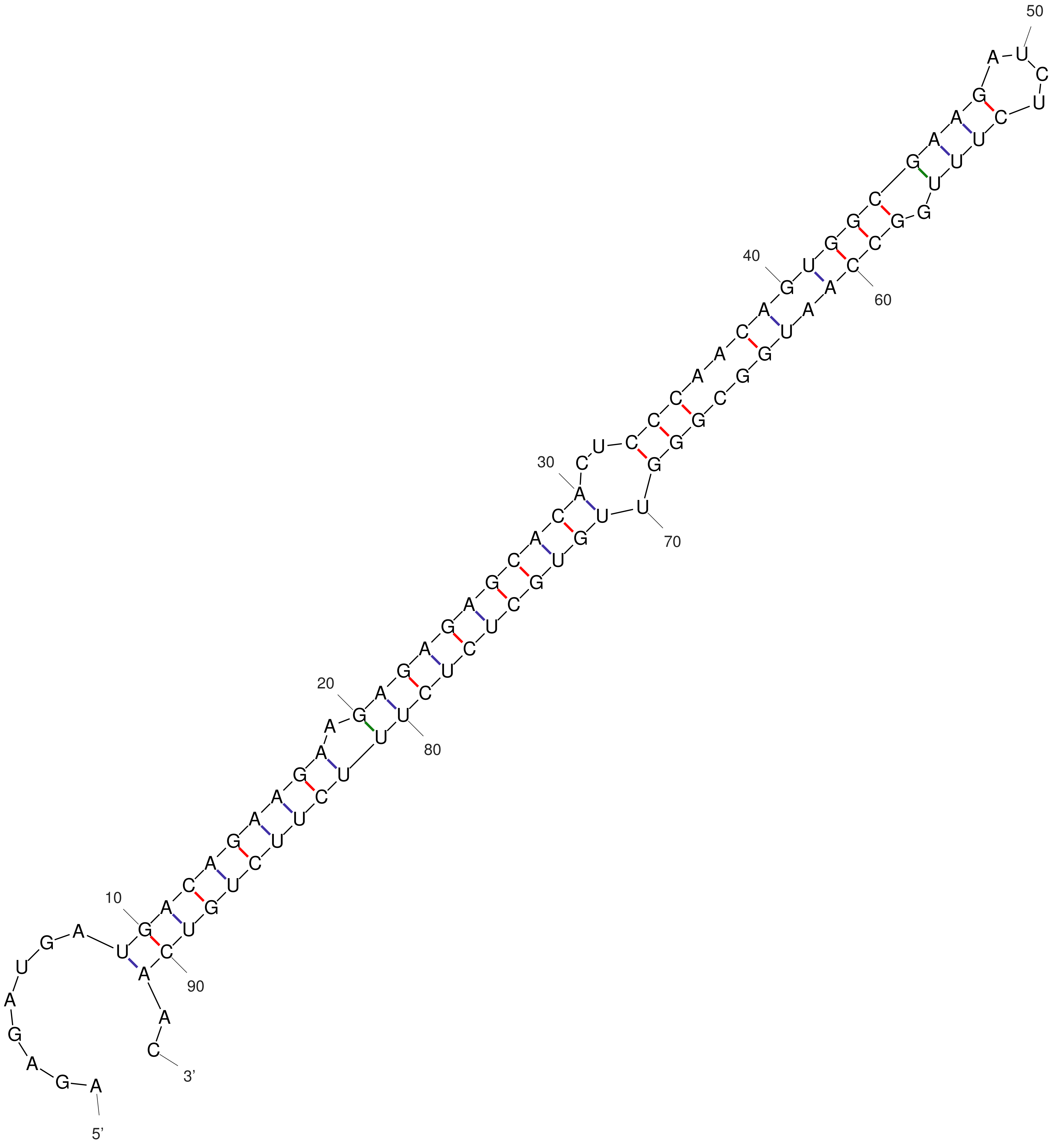

dG = -50.30 [Initially -50.30] Pp-miR156_8 NC_034014 1

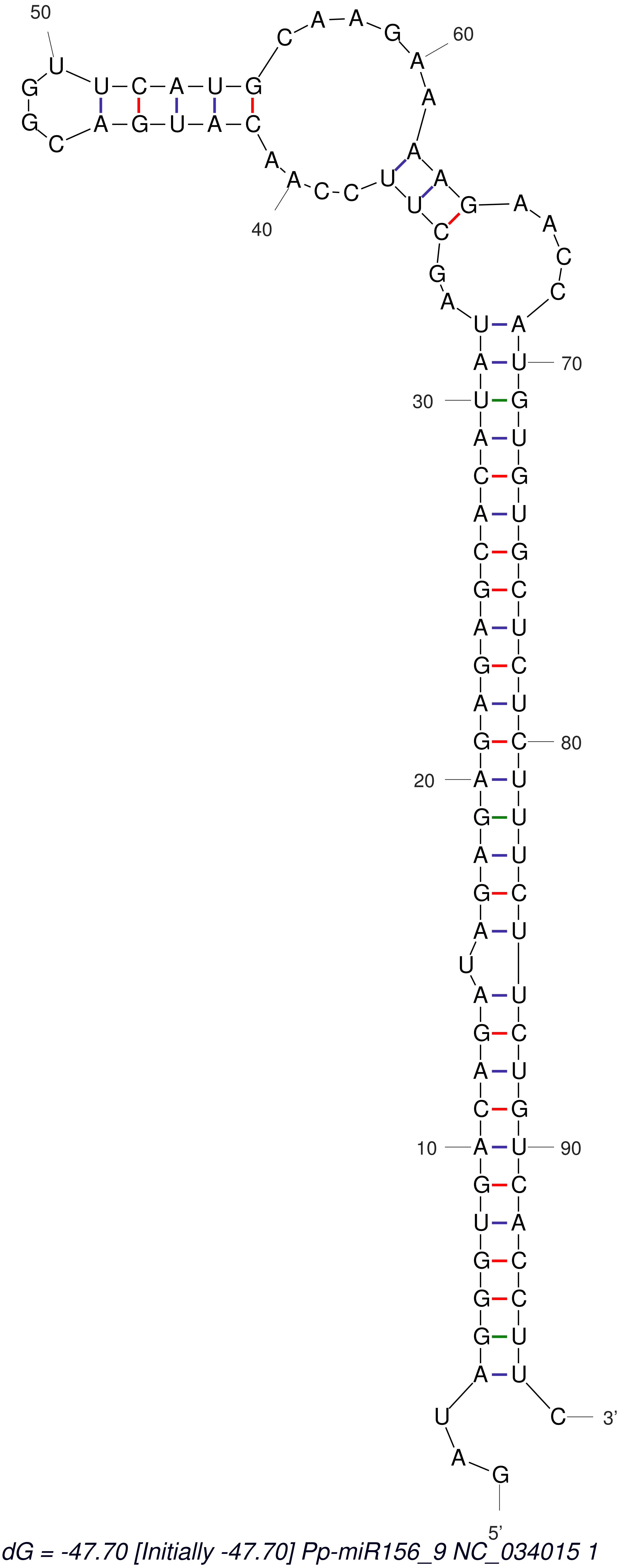

dG = -47.70 [Initially -47.70] Pp-miR156_9 NC_034015 1

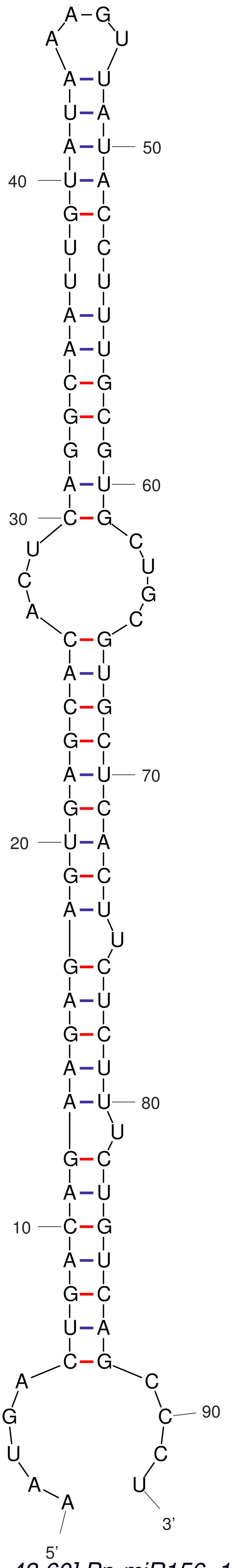

dG = -43.10 [Initially -43.60] Pp-miR156_10 NC_034015 1

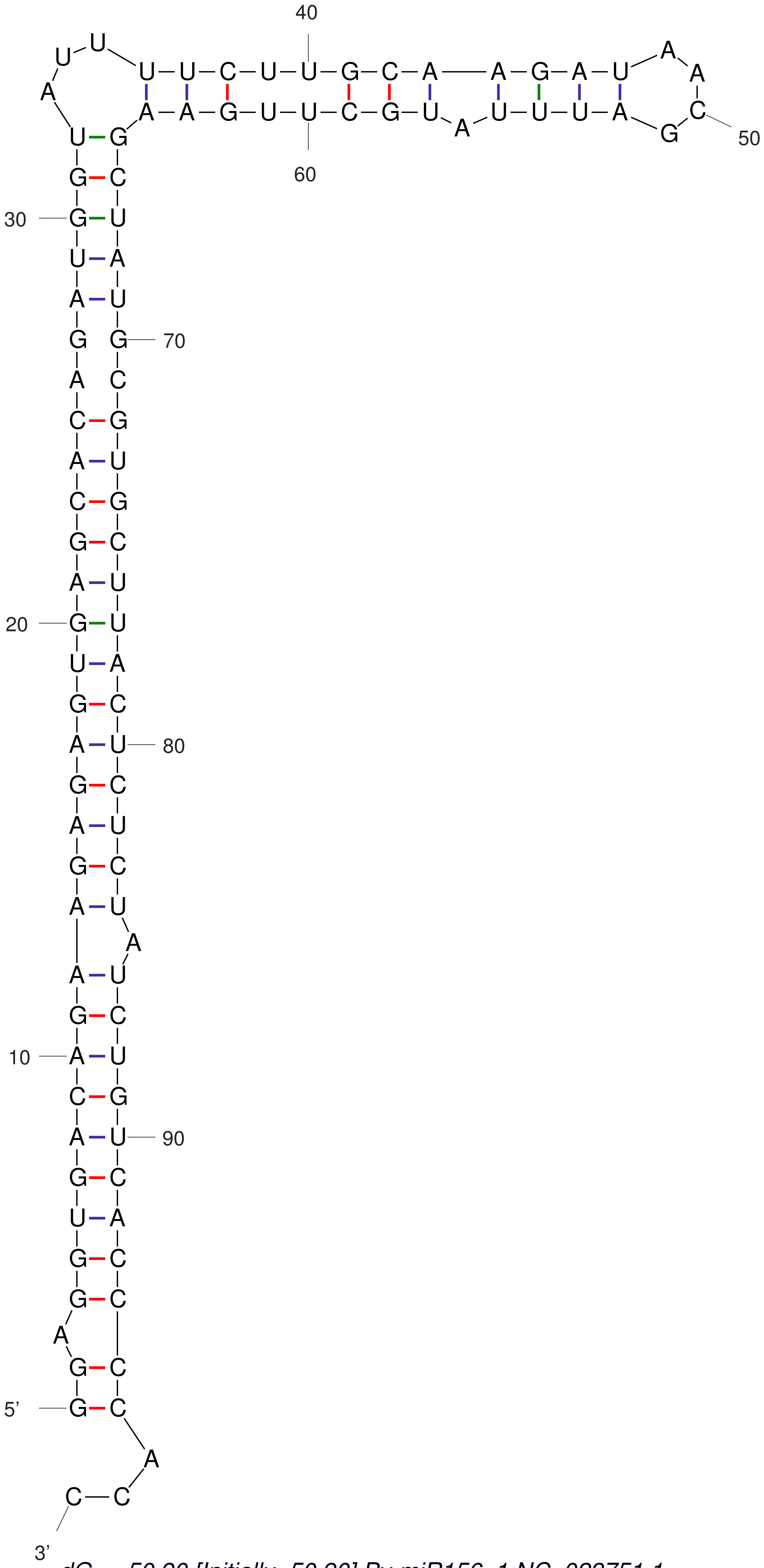

dG = -50.90 [Initially -50.90] Pv-miR156_1 NC_023751 1

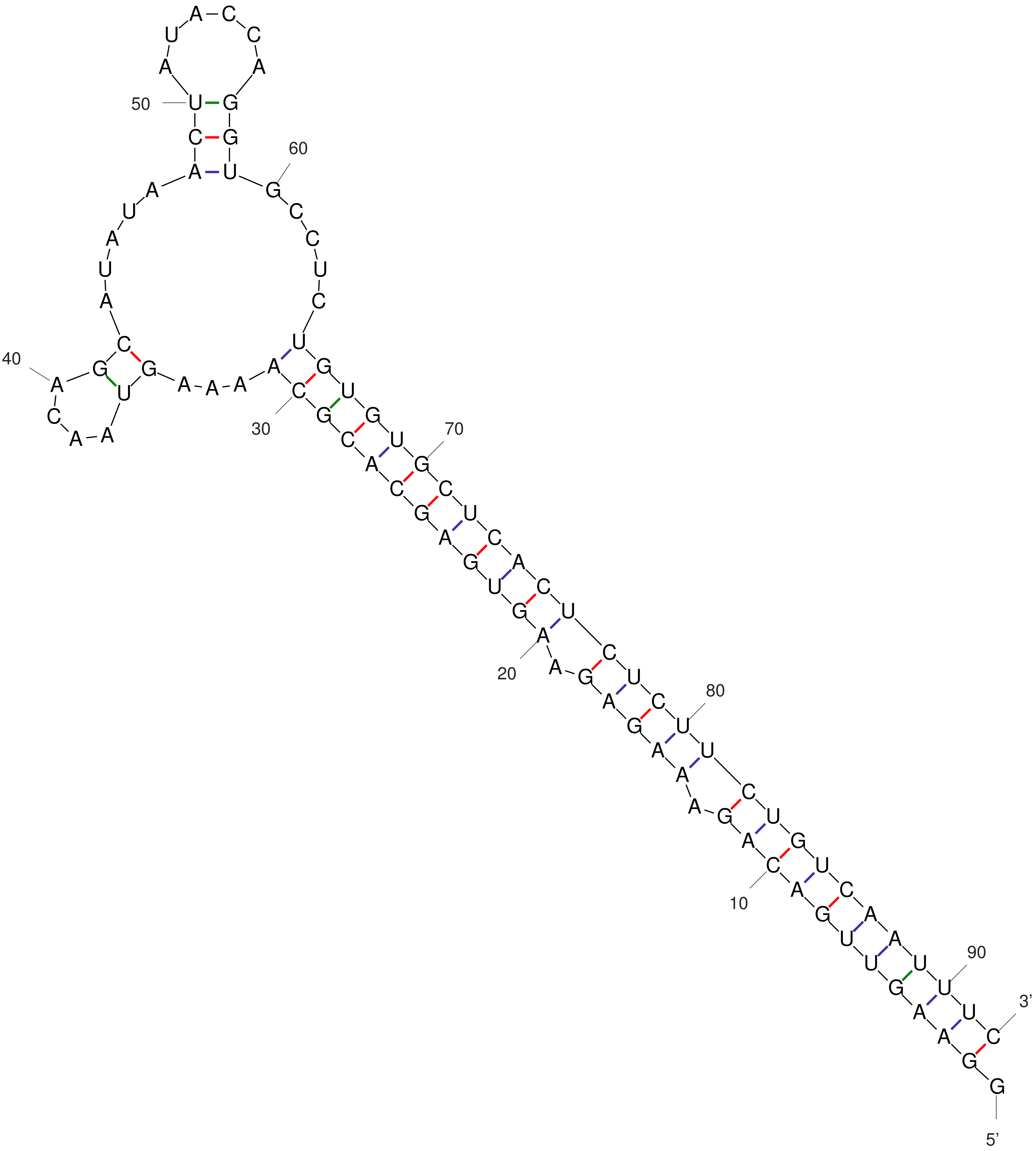

dG = -38.25 [Initially -41.40] Pv-miR156_2 NC_023751 1

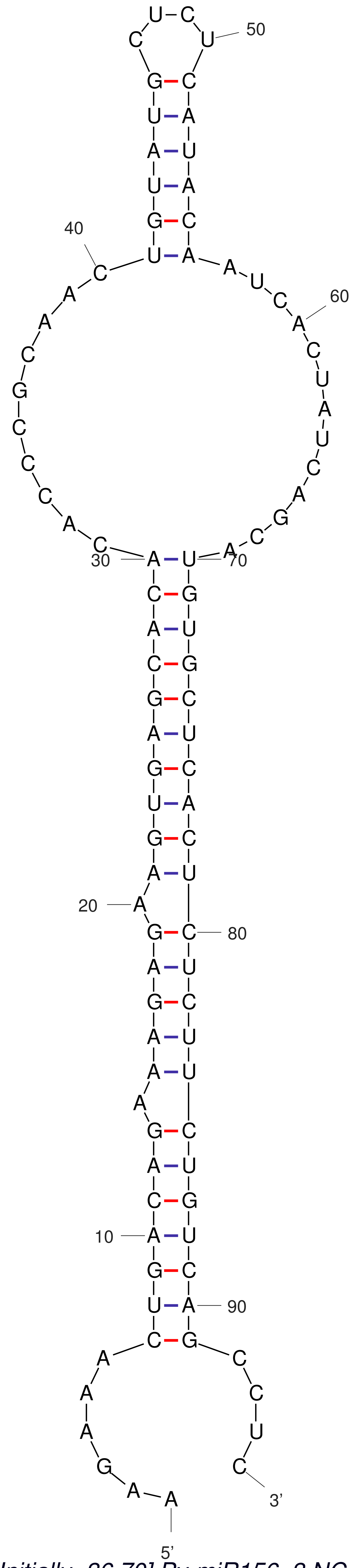

dG = -36.70 [Initially -36.70] Pv-miR156_3 NC_023757 1

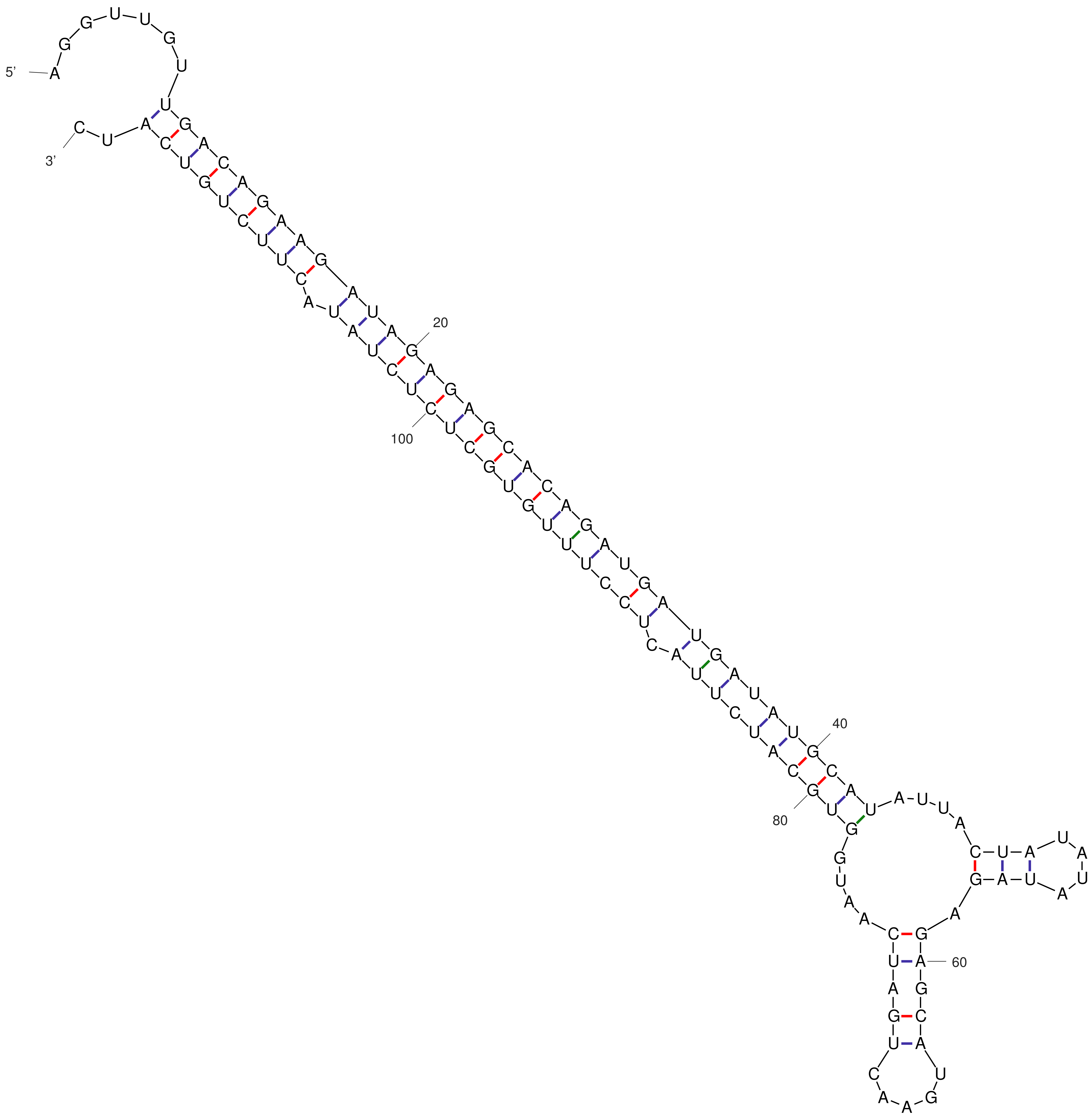

dG = -42.25 [Initially -45.20] Pv-miR156_4 NC_023758 1

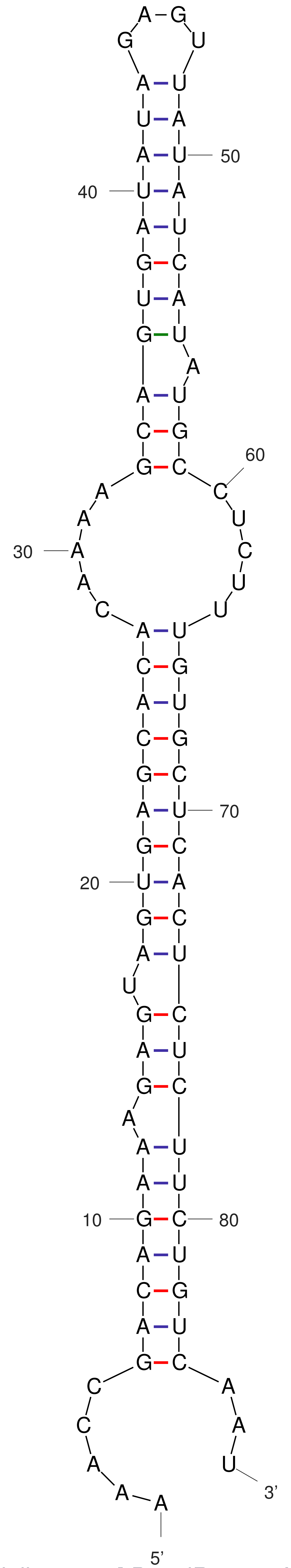

dG = -39.90 [Initially -40.20] Pv-miR156_5 NC_023759 1

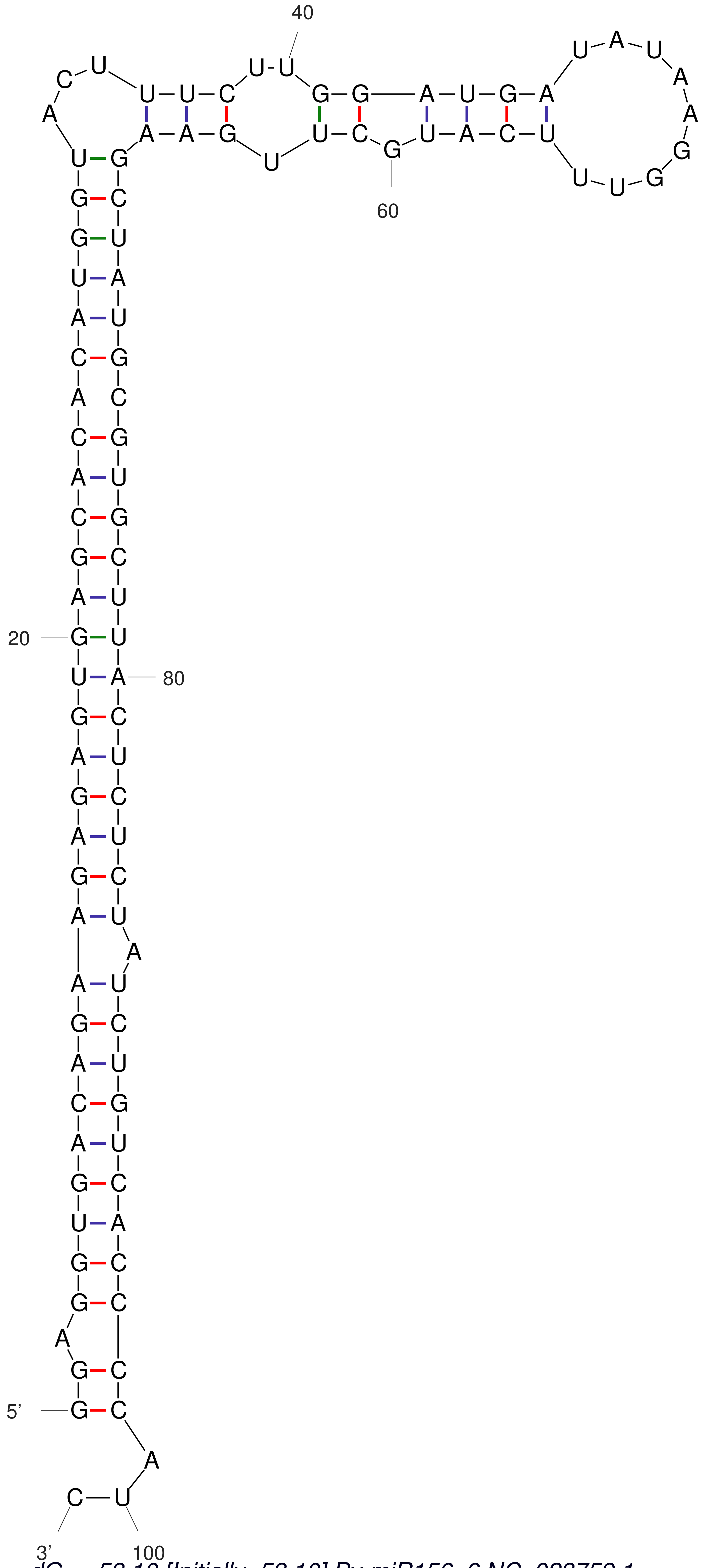

dG = -53.10 [Initially -53.10] Pv-miR156_6 NC_023759 1

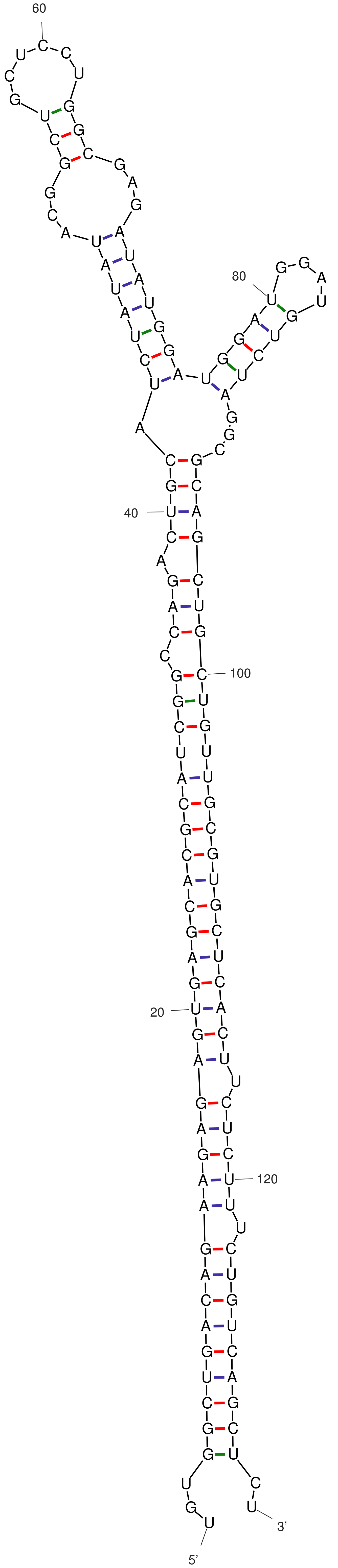

dG = -67.00 [Initially -70.40] sb-miR156_1 NC_012871 2

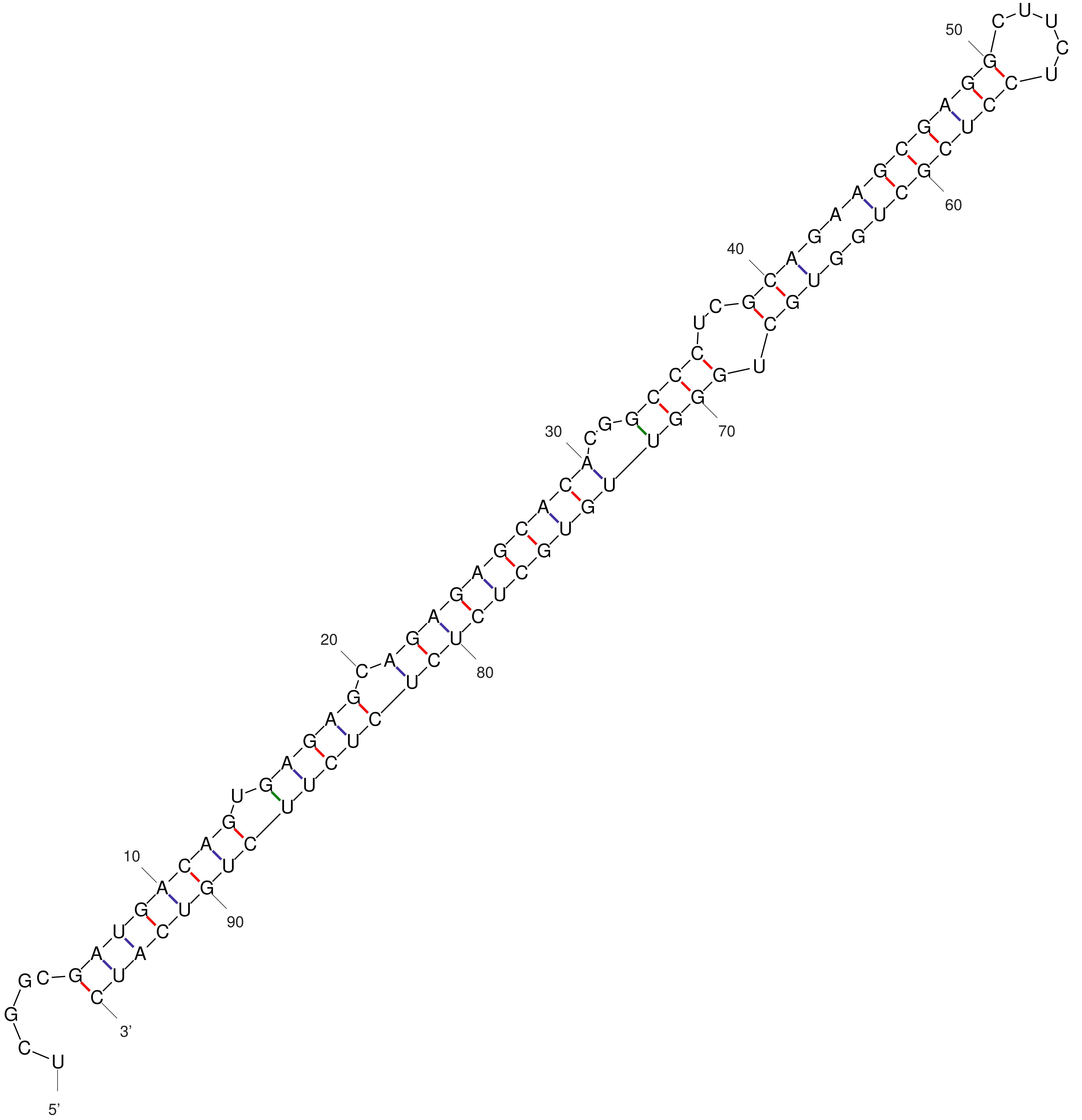

dG = -58.20 [Initially -58.50] sb-miR156_2 NC_012871 2

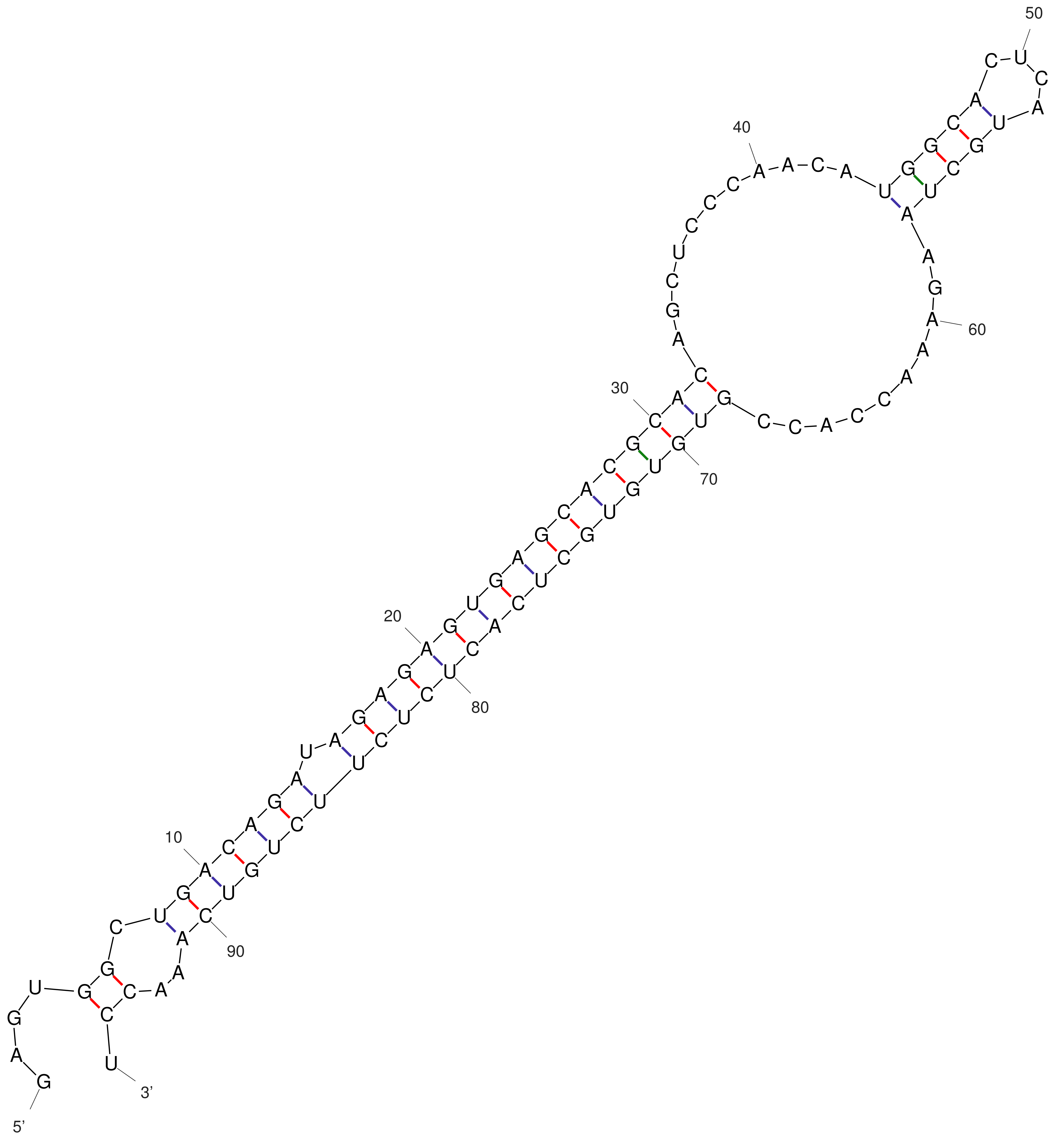

dG = -46.20 [Initially -46.20] sb-miR156_3 NC_012872 2

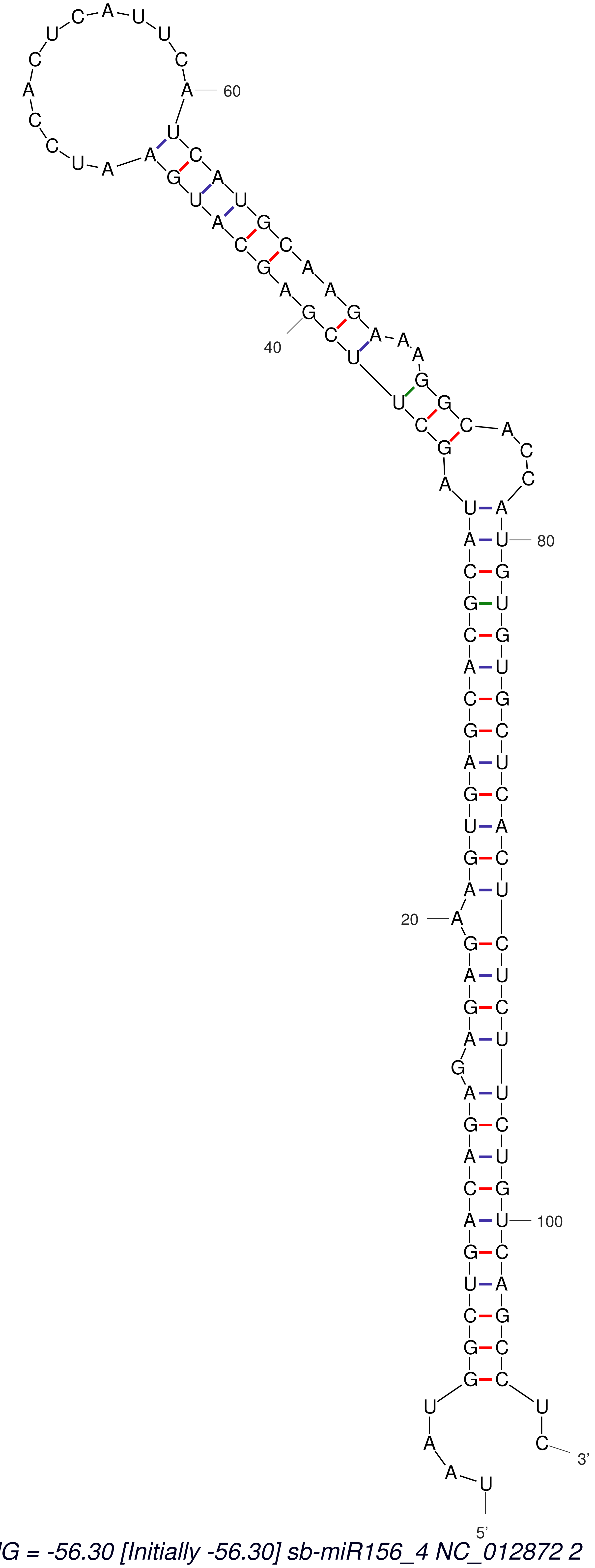

dG = -56.30 [Initially -56.30] sb-miR156_4 NC_012872 2

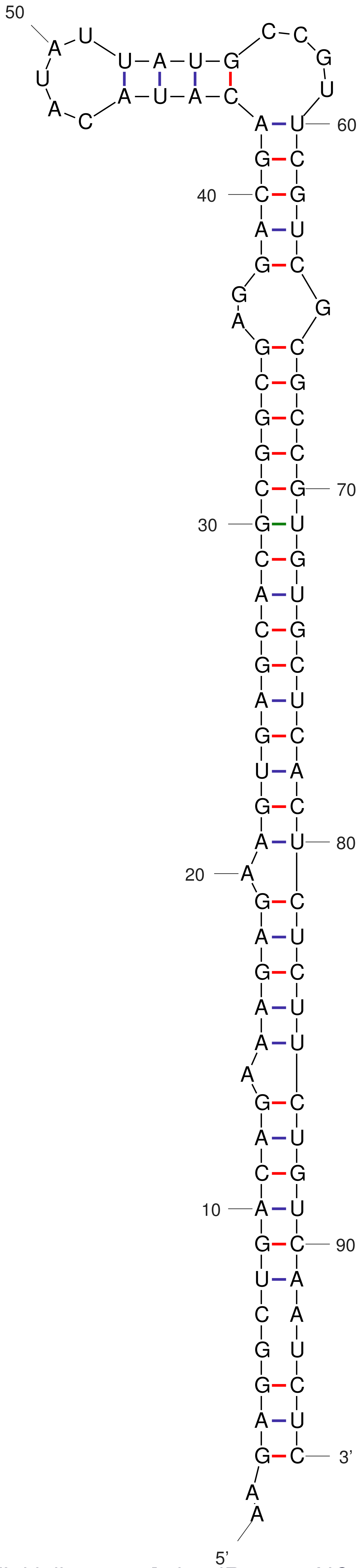

dG = -58.70 [Initially -58.70] sb-miR156_5 NC_012873 2

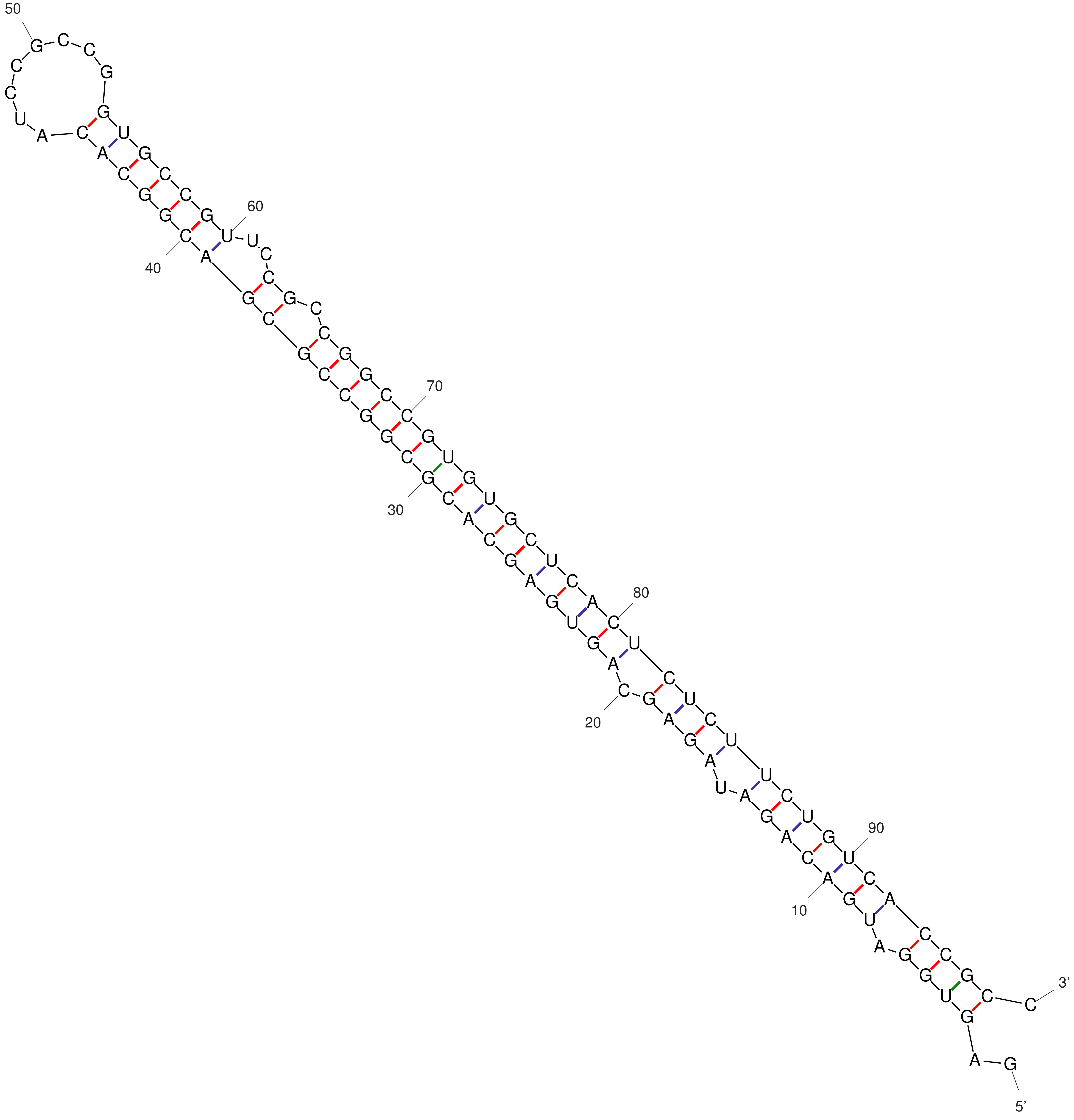

dG = -69.60 [Initially -69.60] sb-miR156_6 NC_012873 2

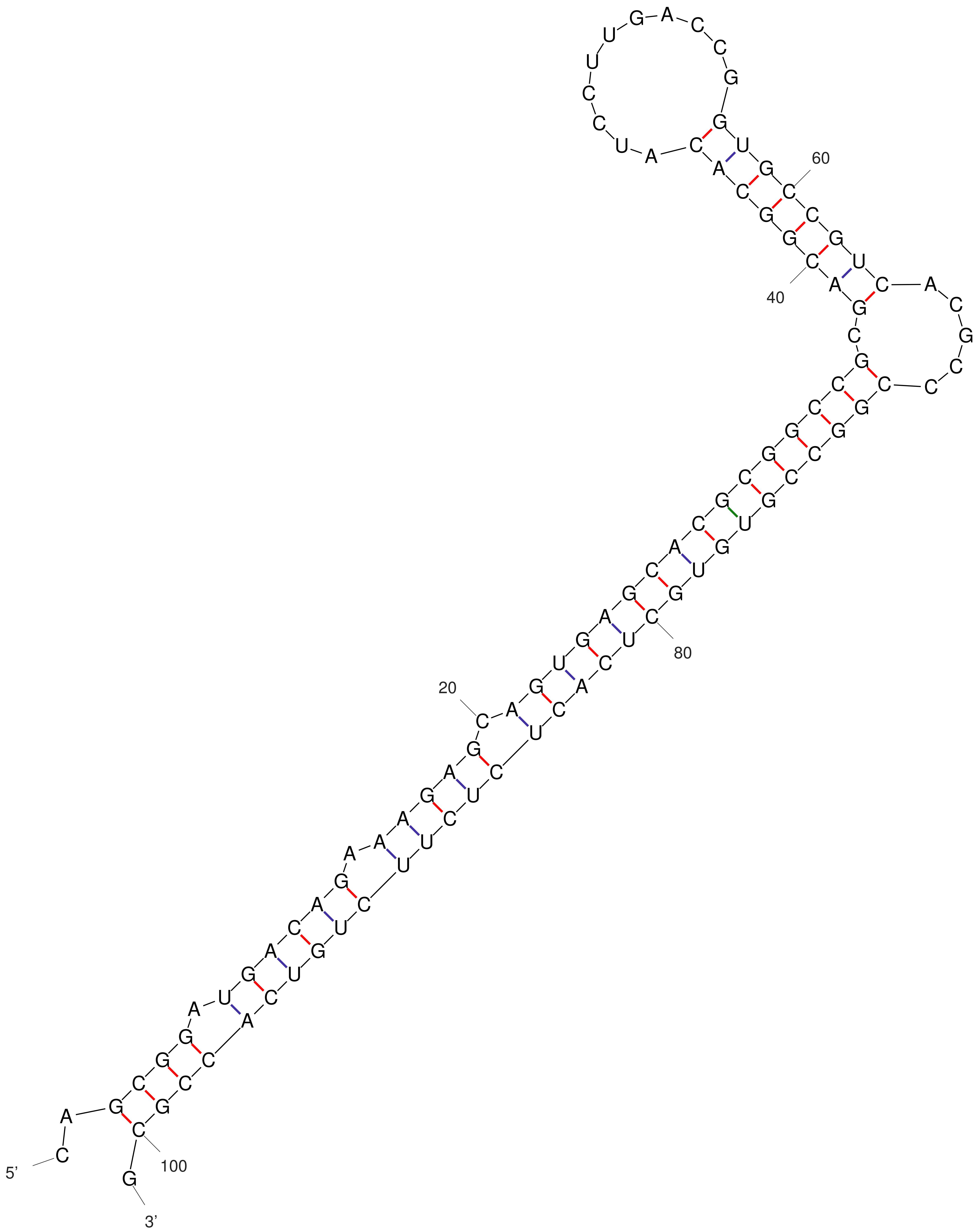

dG = -71.10 [Initially -71.10] sb-miR156_7 NC_012875 2

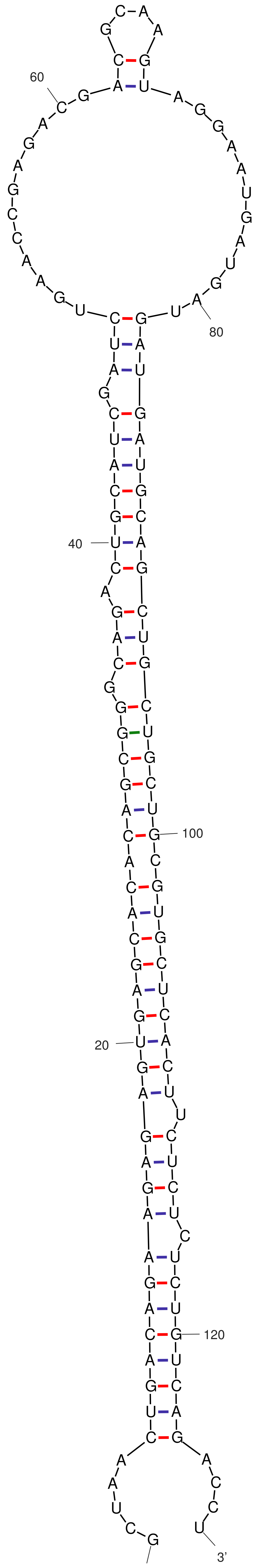

dG = -66.80 [Initially -67.30] sb-miR156_8 NC_012876 2

Output of sir_graph (©)
mfold_util 4.7

Created Mon Jul 22 08:54:38 2019

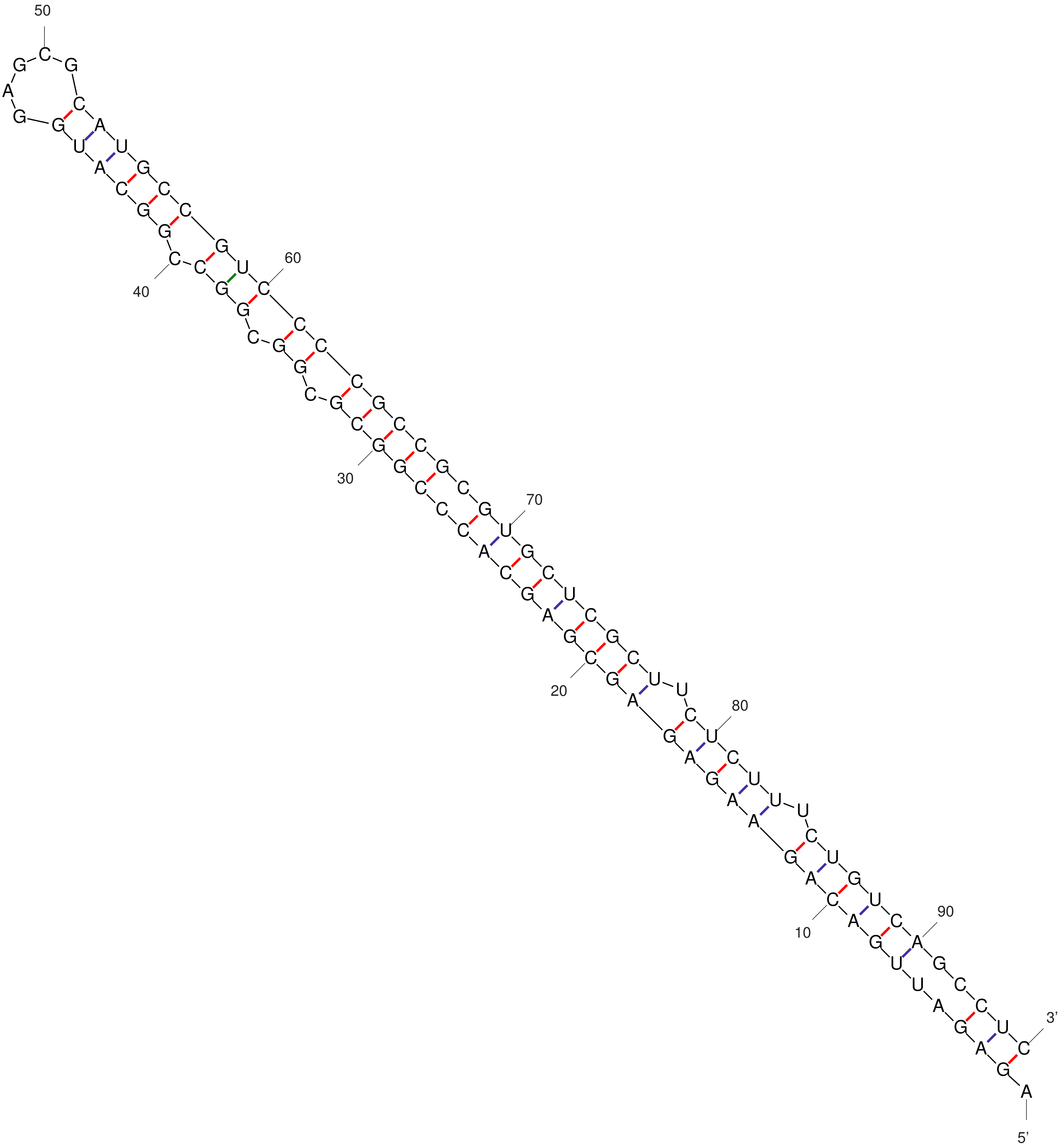

dG = -63.70 [Initially -63.70] sb-miR156_9 NC_012879 2

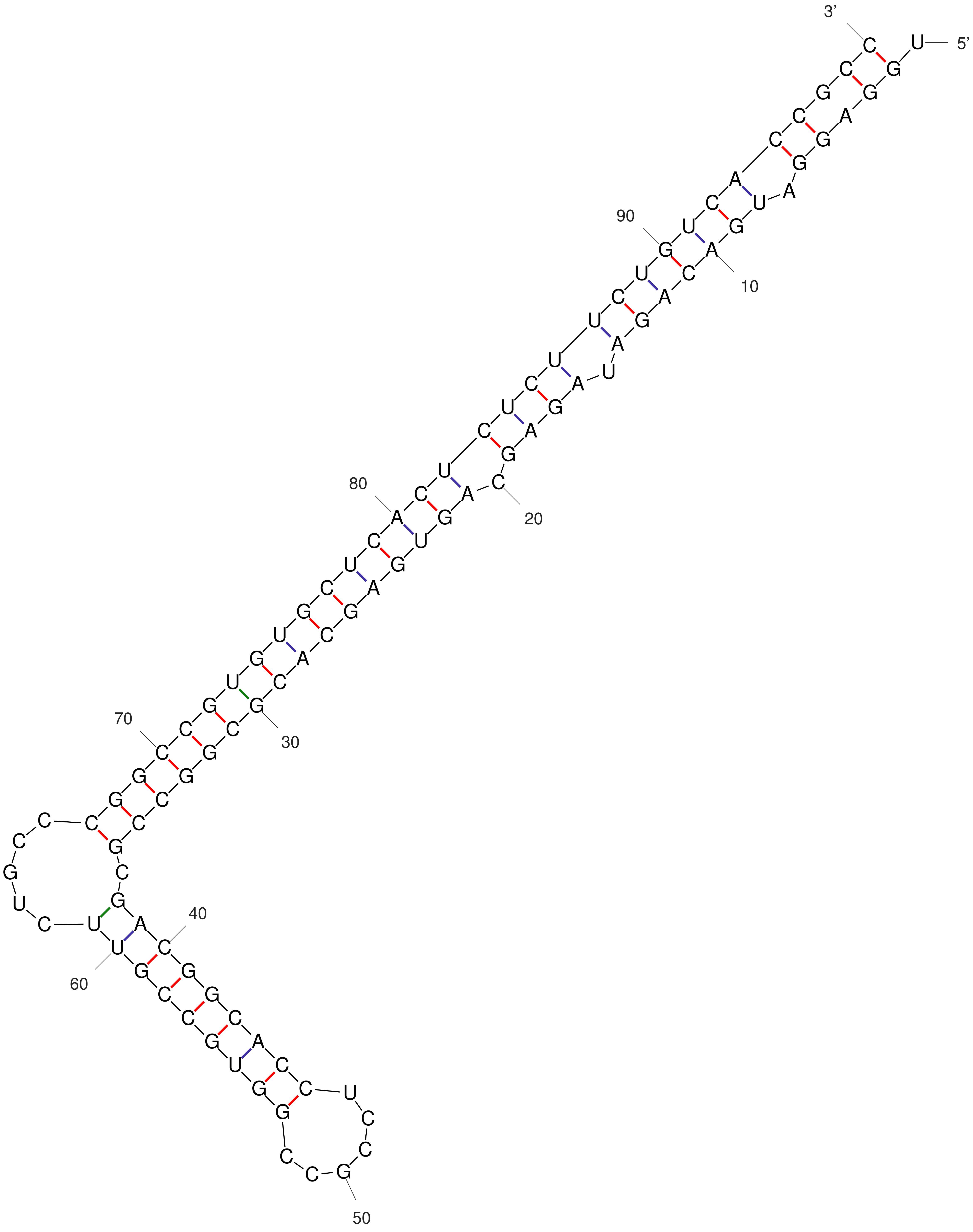

dG = -69.50 [Initially -69.50] Si-miR156_1 NC_028450 1

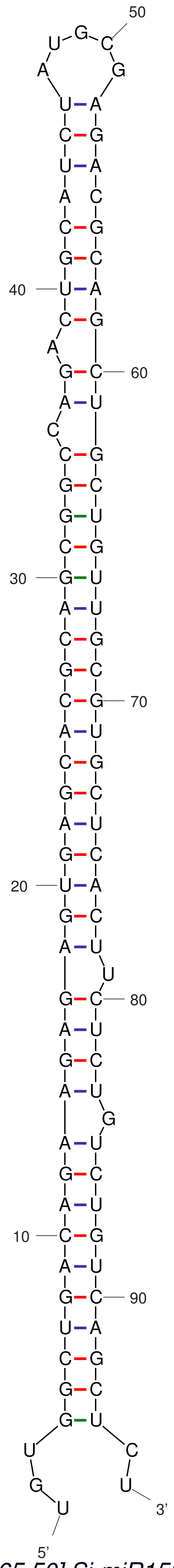

dG = -65.50 [Initially -65.50] Si-miR156_2 NC_028451 1

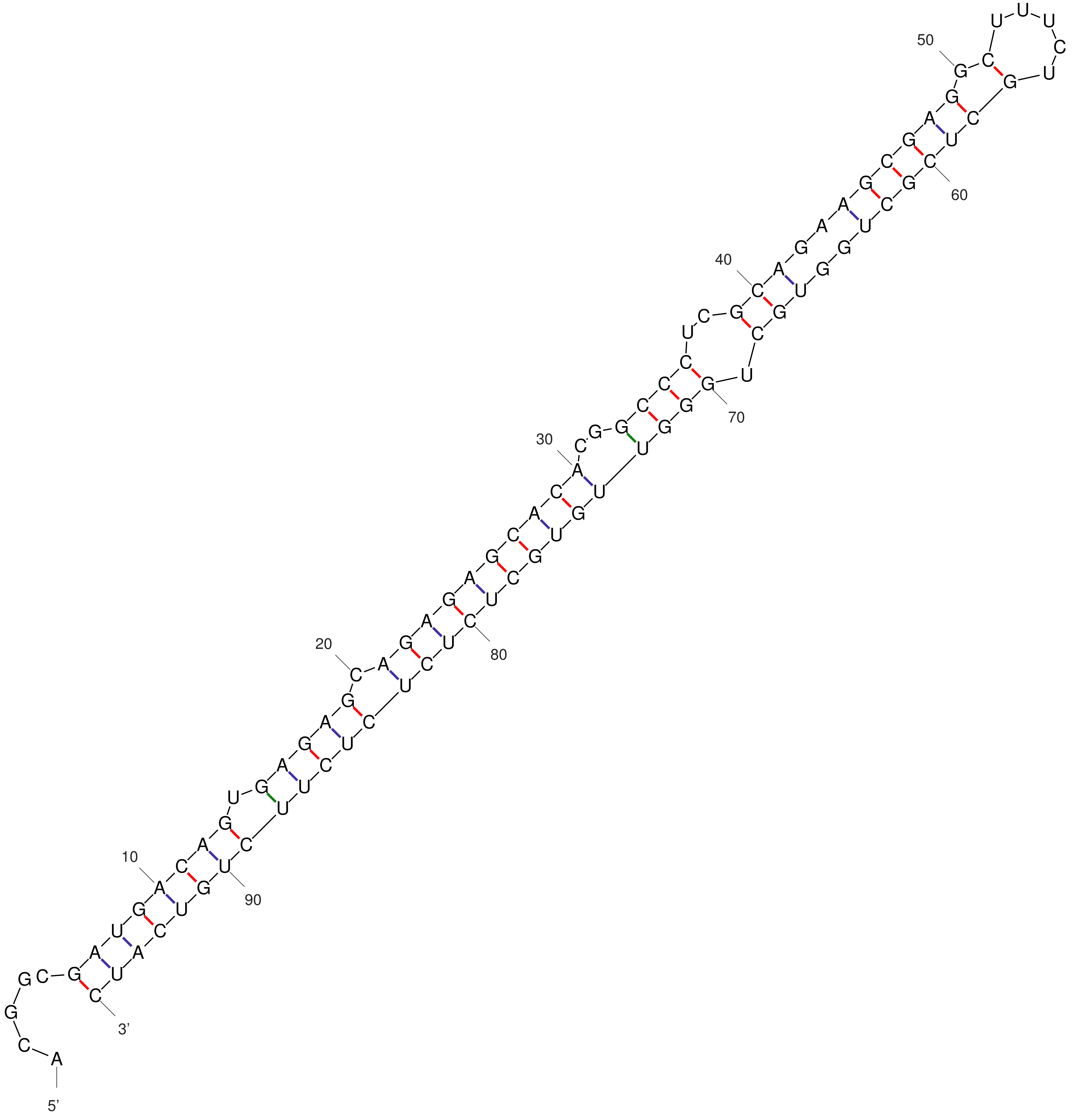

dG = -56.00 [Initially -56.30] Si-miR156_3 NC_028451 1

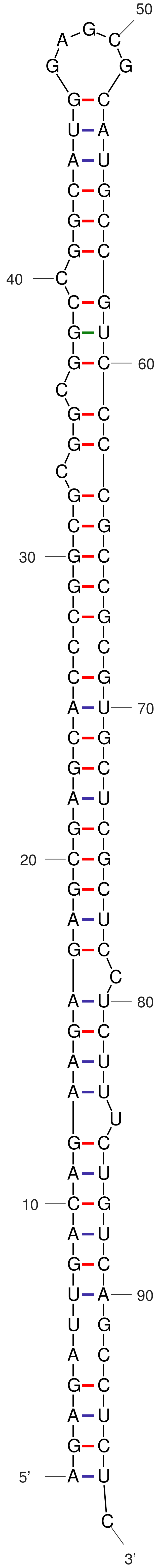

dG = -65.20 [Initially -65.20] Si-miR156_4 NC_028453 1

Output of sir_graph (©)
mfold_util 4.7

Created Tue May 21 04:15:51 2019

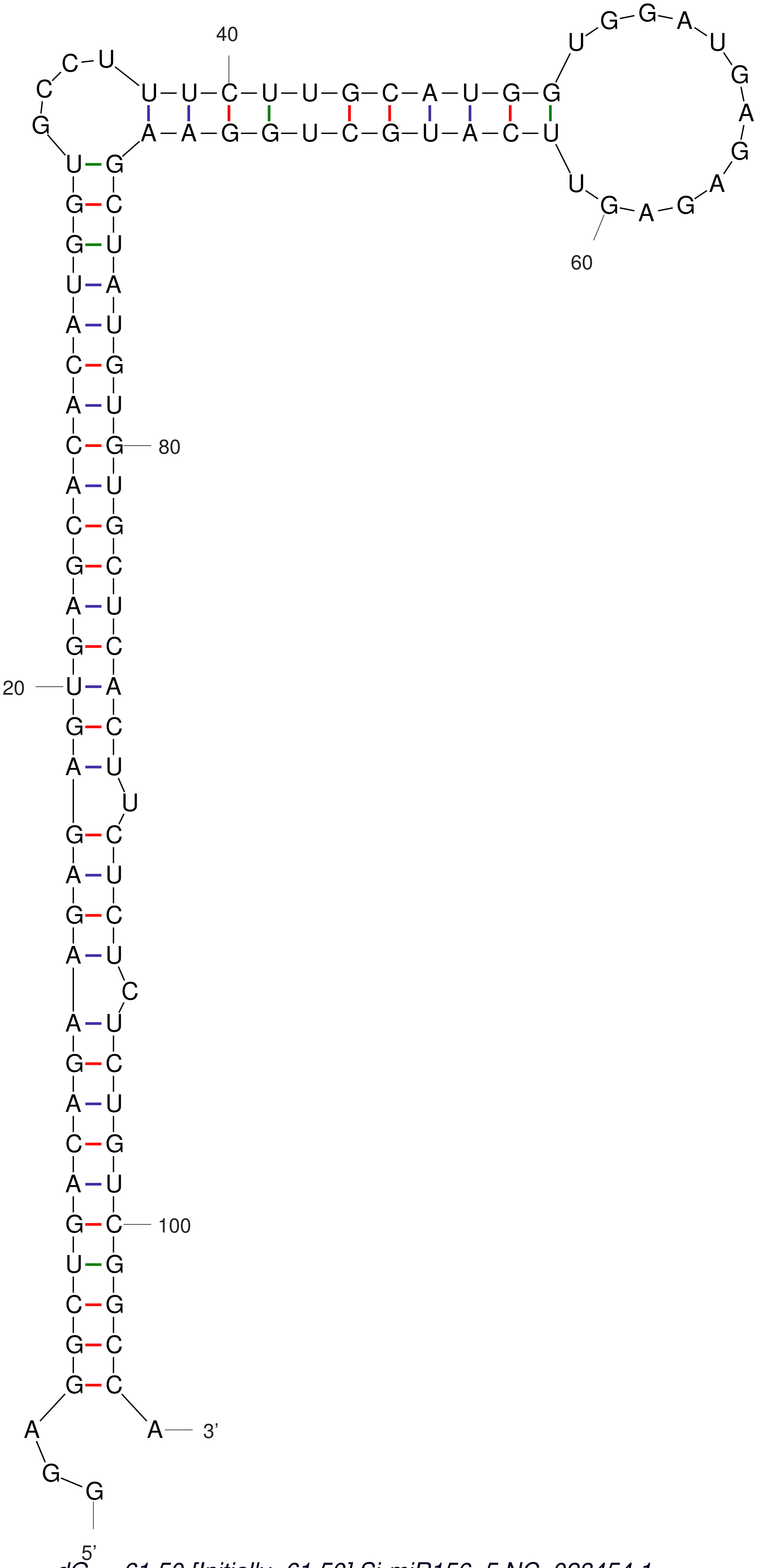

dG = -61.50 [Initially -61.50] Si-miR156_5 NC_028454 1

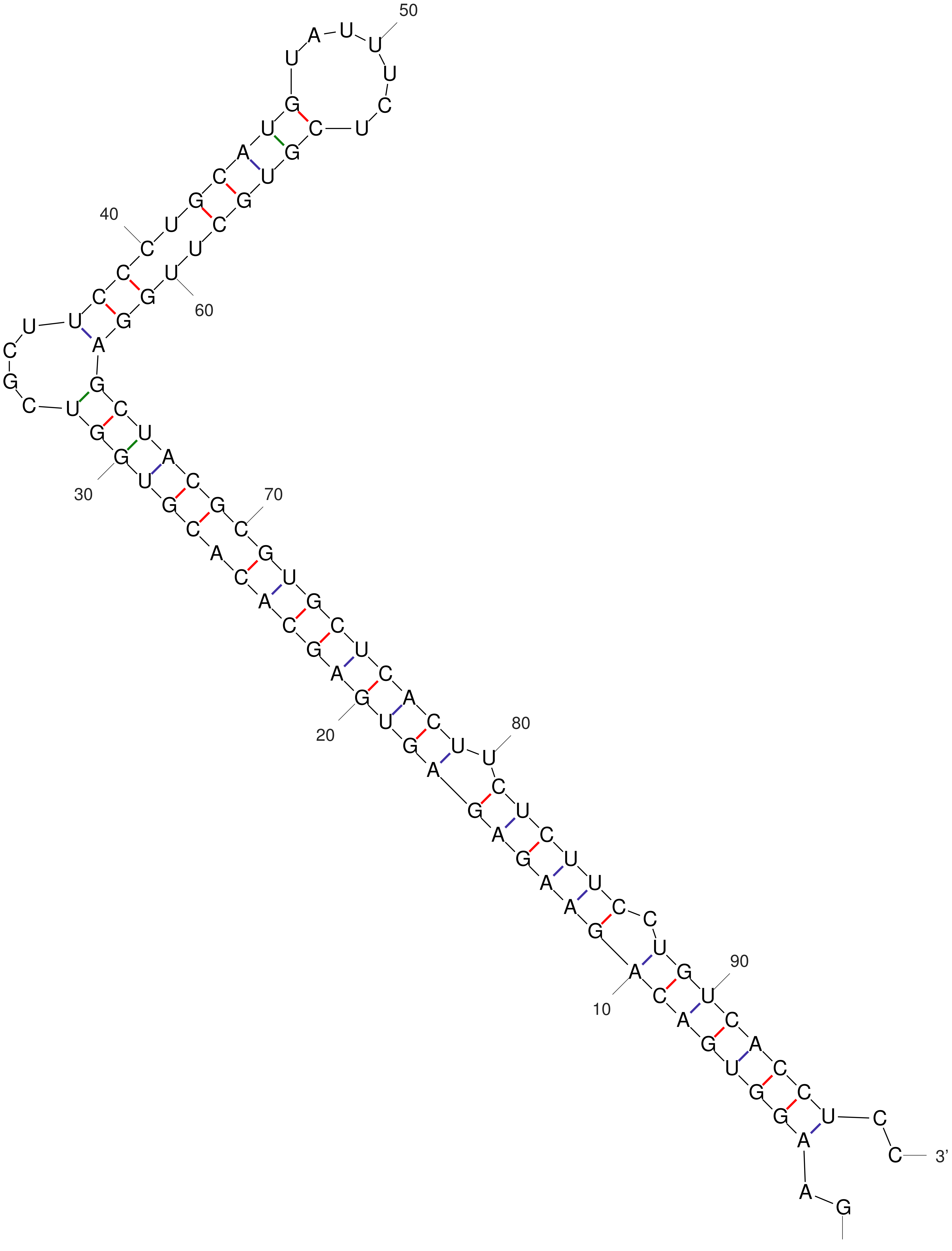

dG = -56.80 [Initially -56.80] Si-miR156_6 NC_028454 1

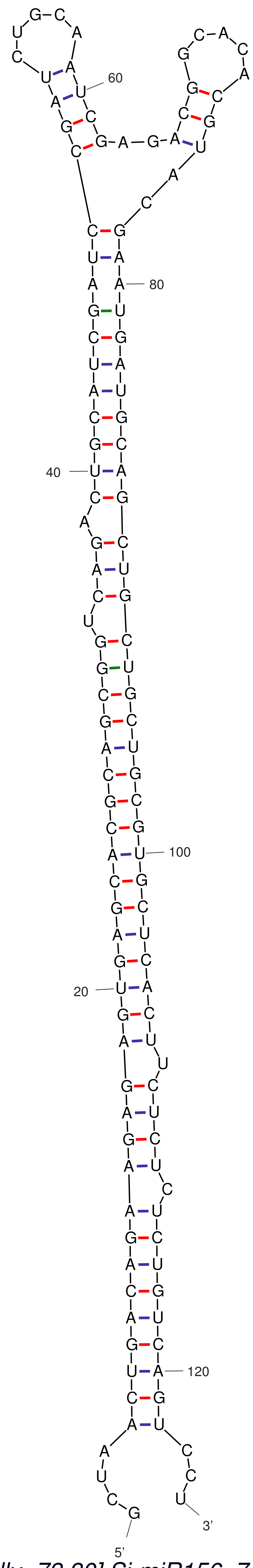

dG = -71.80 [Initially -73.30] Si-miR156_7 NC_028455 1

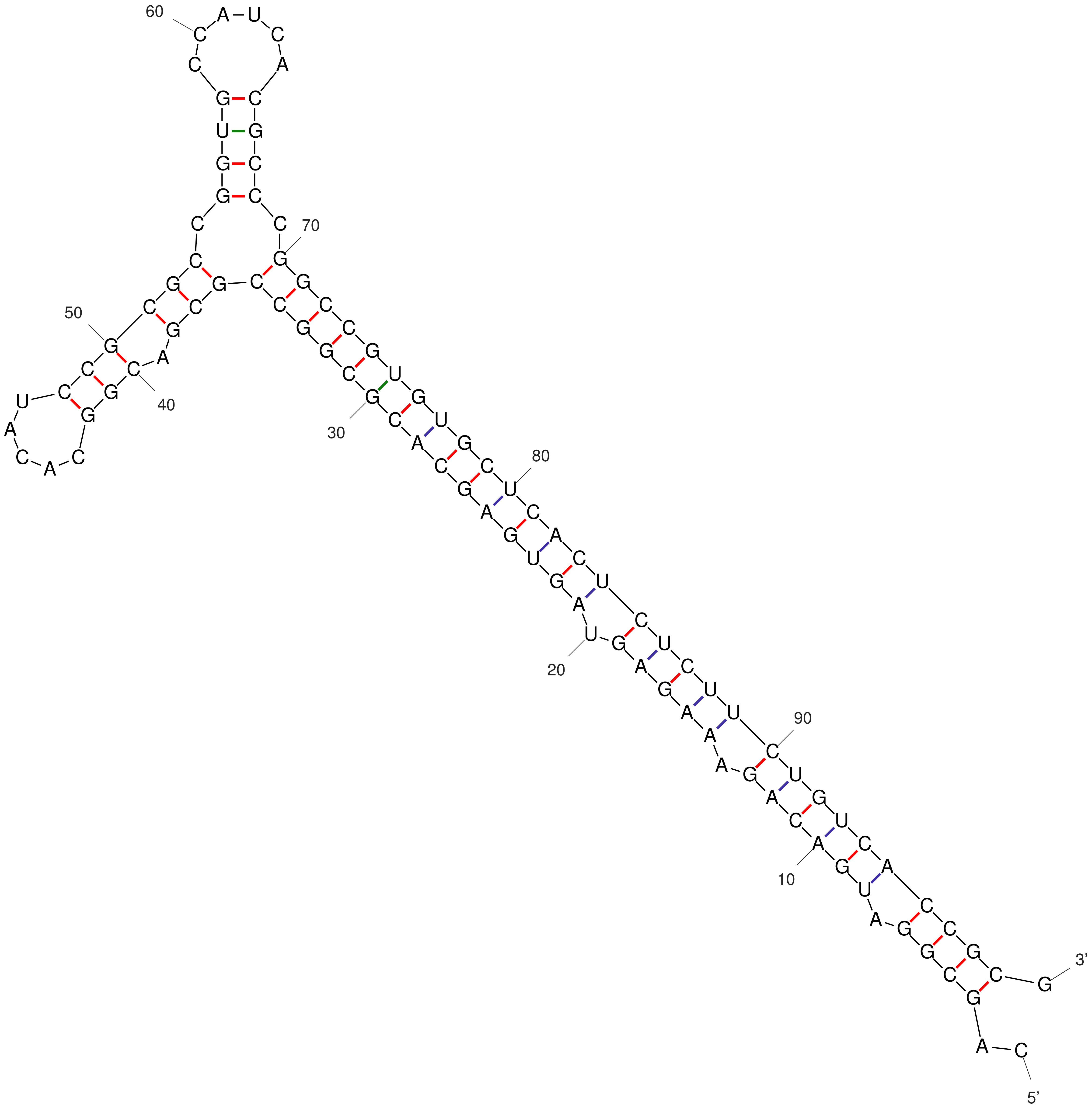

dG = -63.70 [Initially -65.80] Si-miR156_8 NC_028456 1

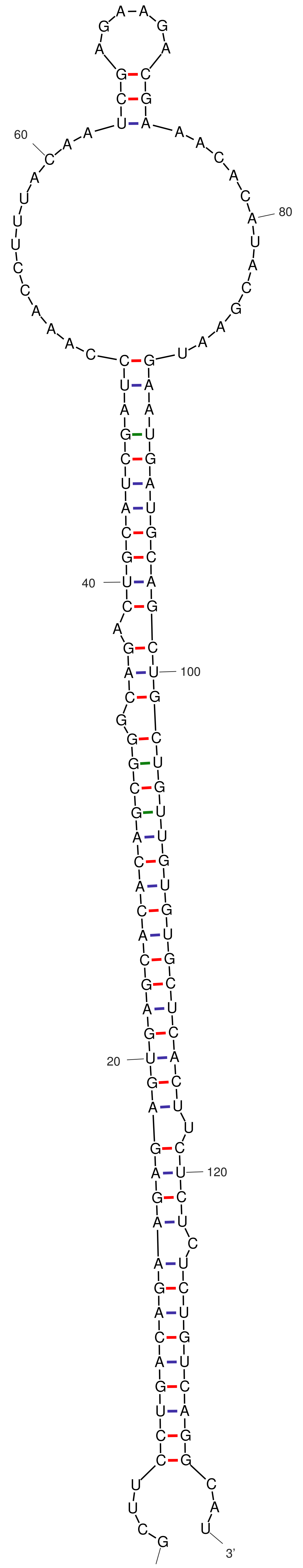

dG = -68.10 [Initially -68.10] Si-miR156_9 NC_028457 1

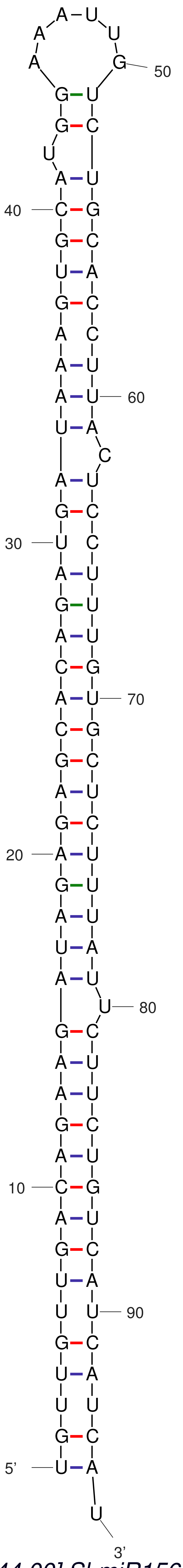

dG = -44.00 [Initially -44.00] Sl-miR156_1 NC_015439 3

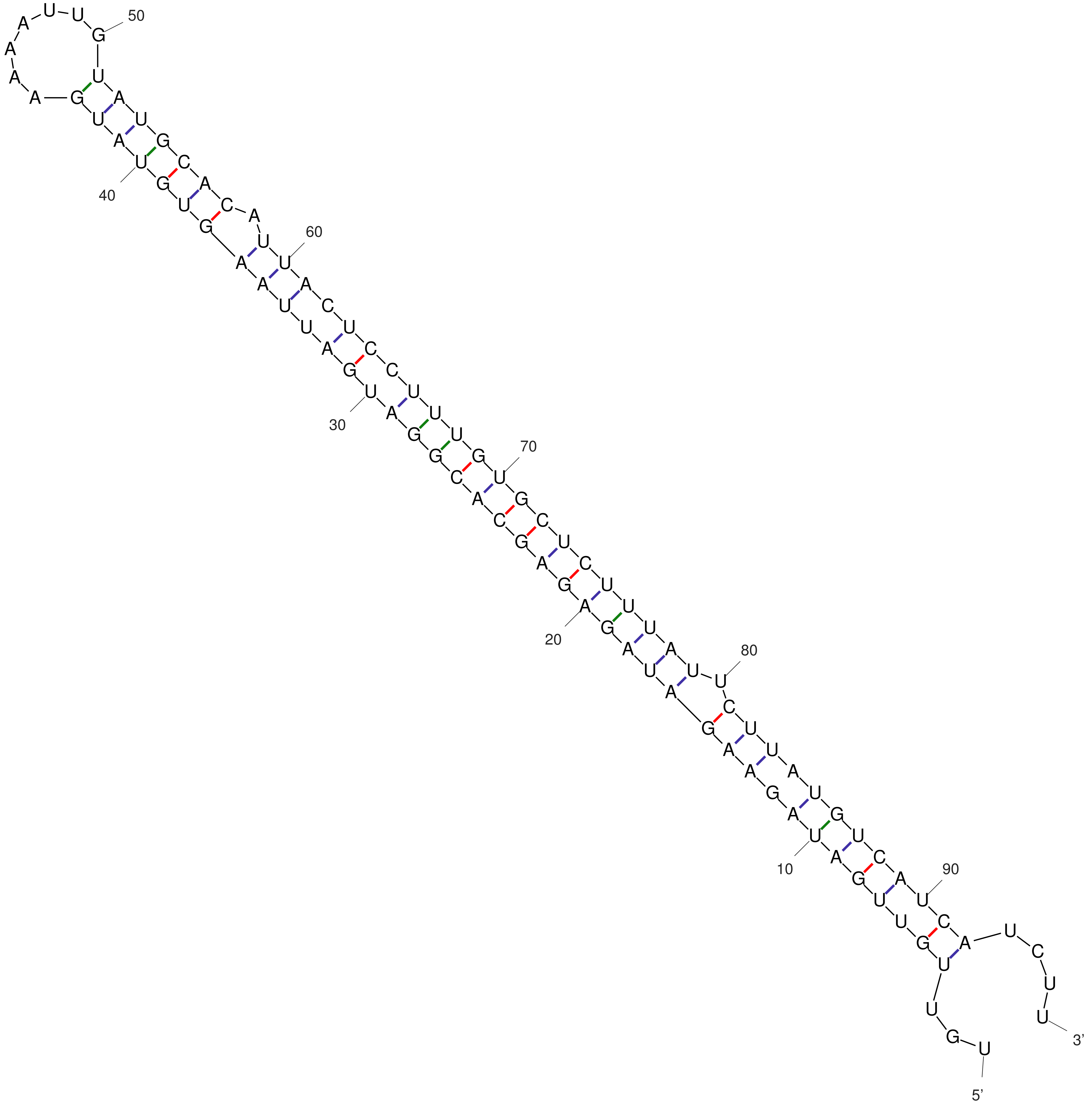

dG = -34.40 [Initially -34.40] Sl-miR156_2 NC_015439 3

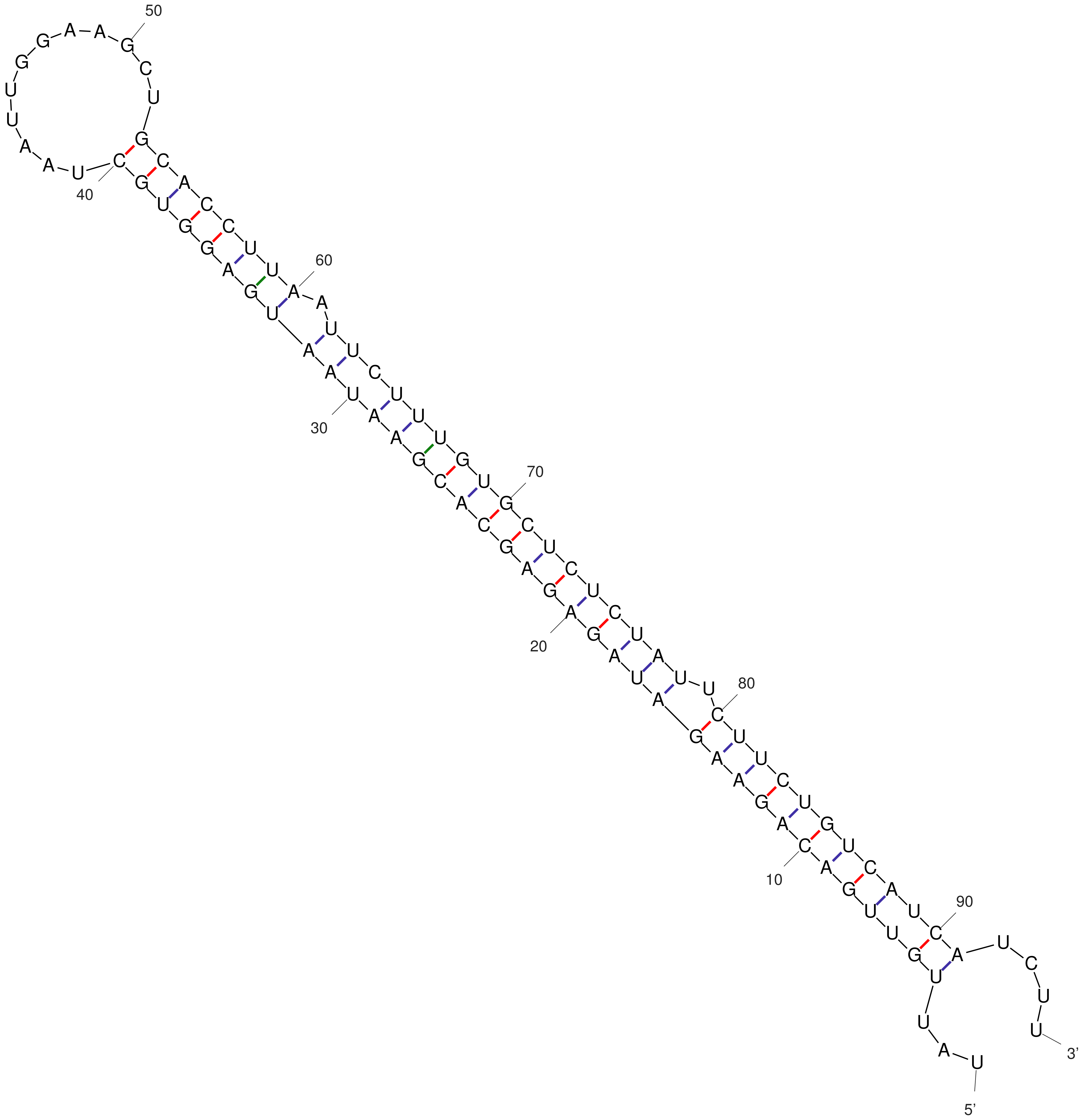

dG = -48.30 [Initially -48.30] Sl-miR156_3 NC_015439 3

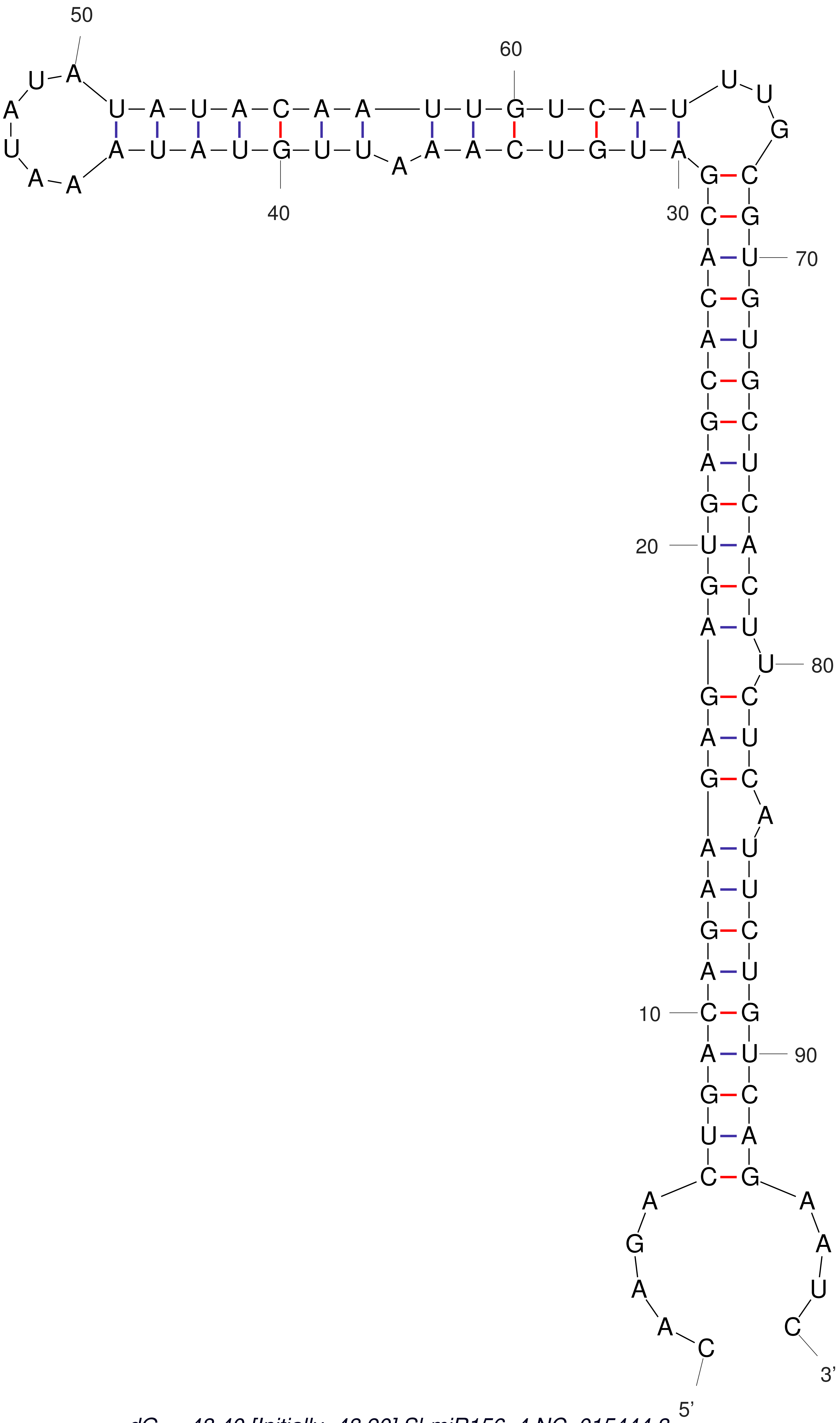

dG = -48.40 [Initially -48.90] Sl-miR156_4 NC_015444 3

Output of sir_graph (©)
mfold_util 4.7

Created Tue May 21 00:05:35 2019

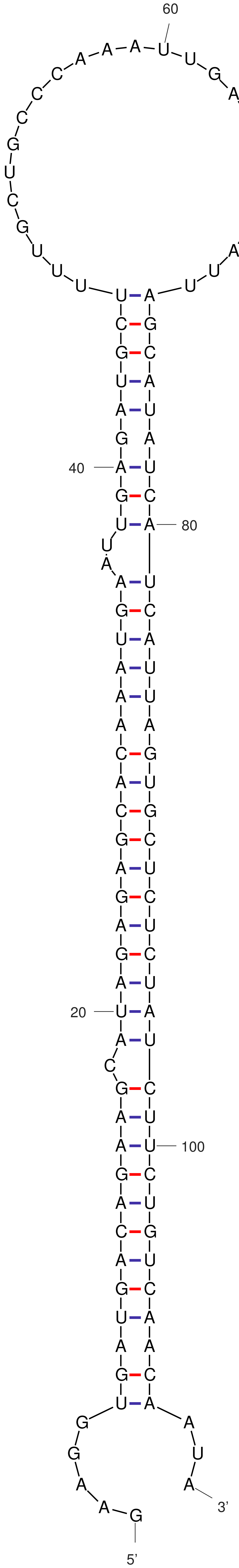

dG = -43.80 [Initially -44.00] Sl-miR156_5 NC_015443 3

Output of sir_graph (©)
mfold_util 4.7

Created Tue May 21 00:07:05 2019

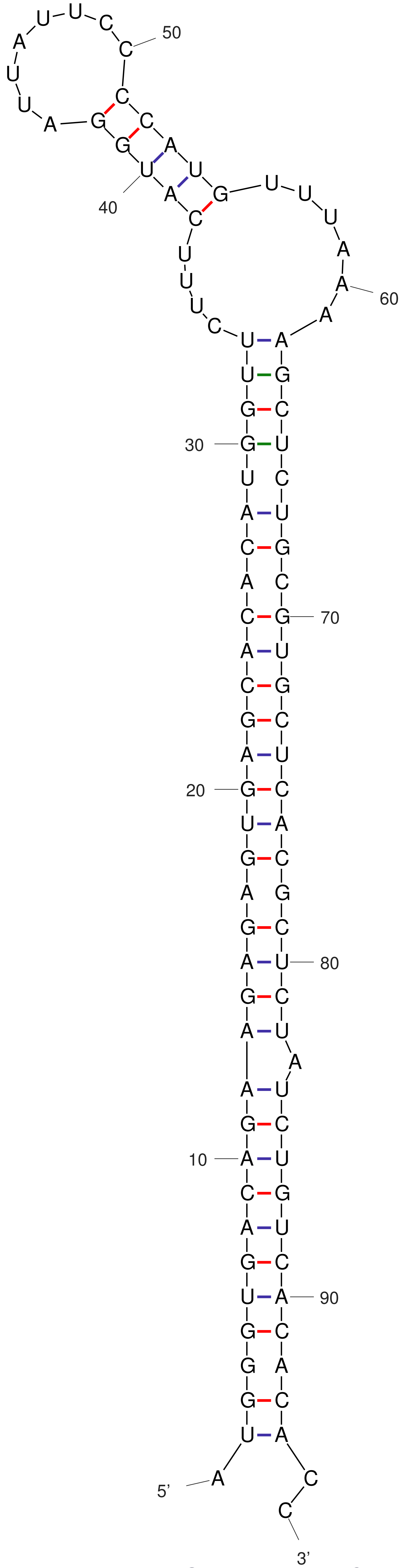

dG = -44.80 [Initially -44.80] Sl-miR156_6 NC_015444 3

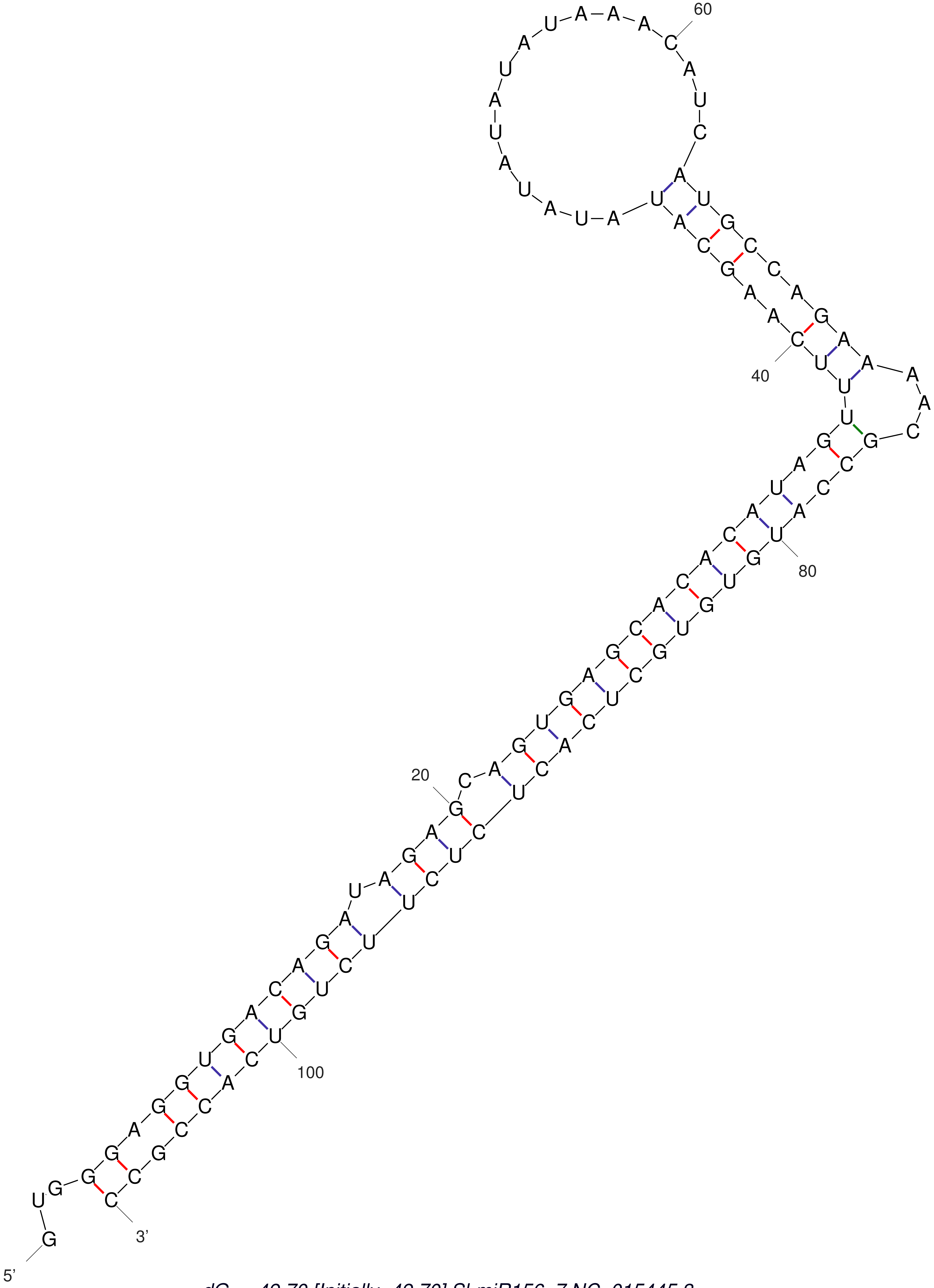

dG = -49.70 [Initially -49.70] Sl-miR156_7 NC_015445 3

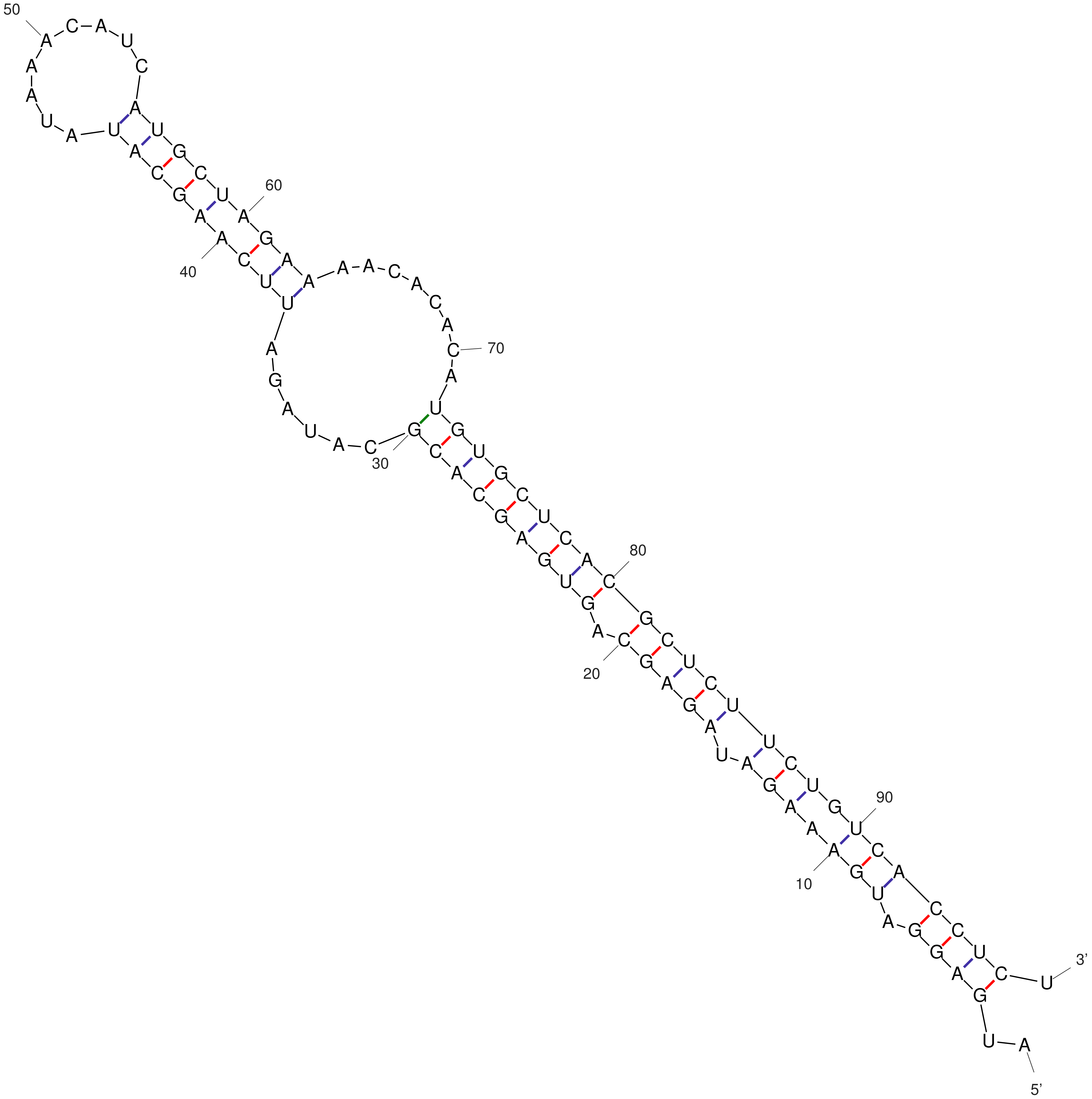

dG = -37.80 [Initially -37.80] Sl-miR156_8 NC_015445 3

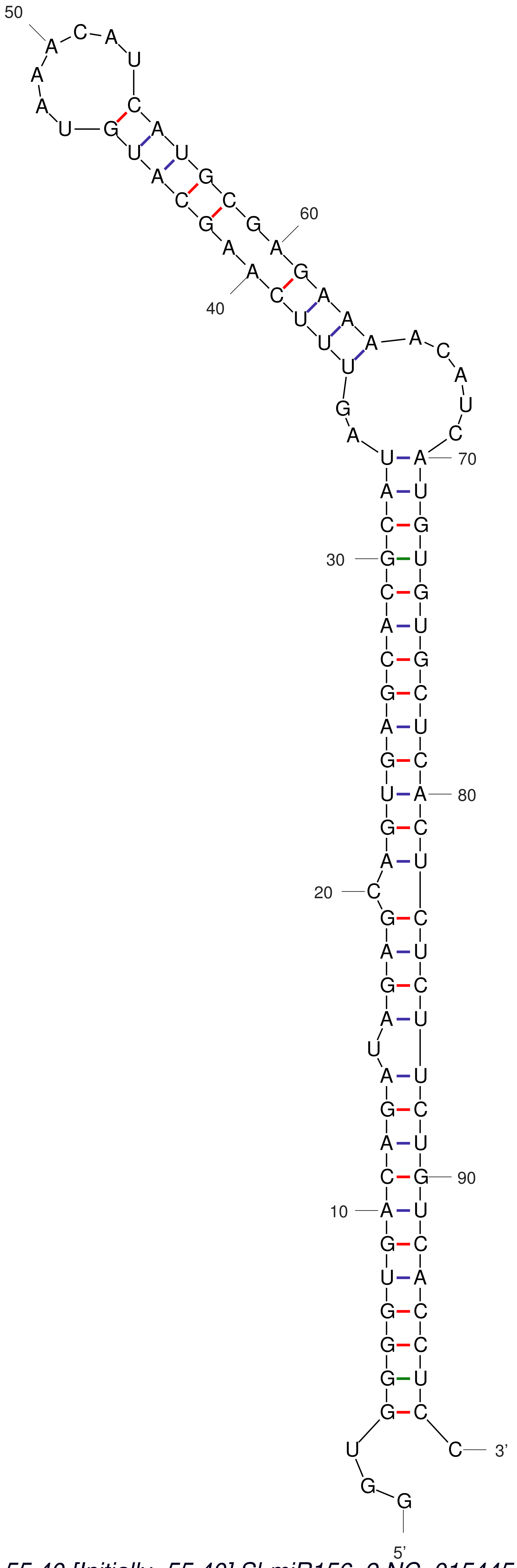

dG = -55.40 [Initially -55.40] Sl-miR156_9 NC_015445 3

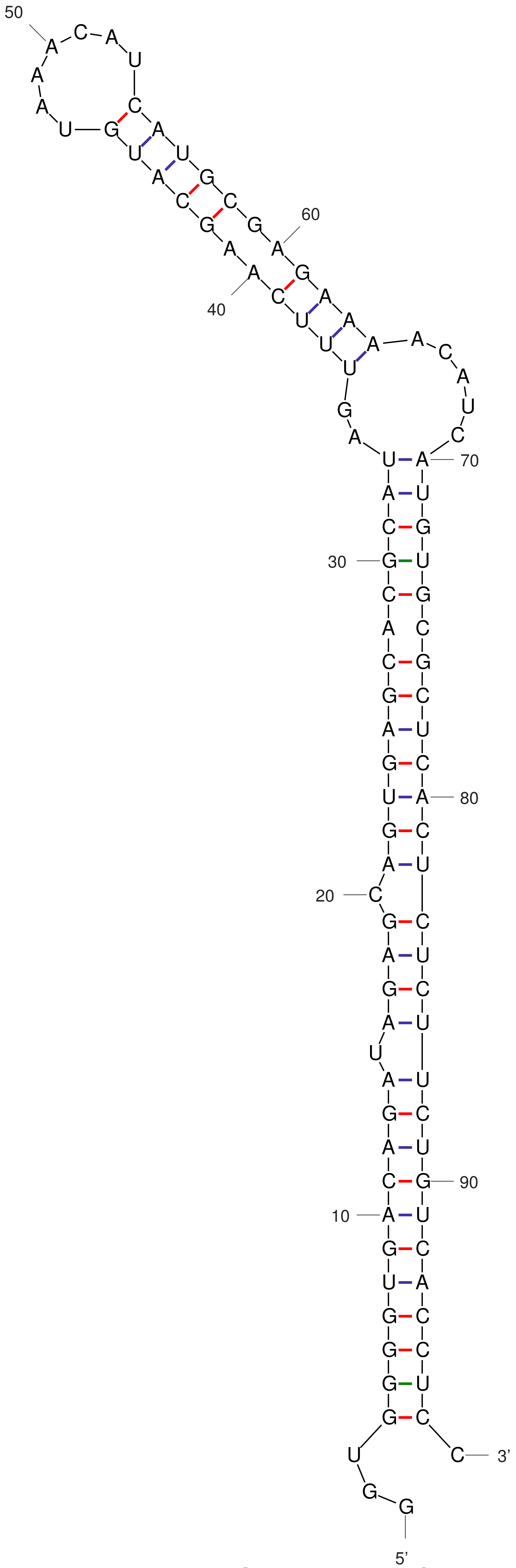

dG = -51.50 [Initially -51.50] Sl-miR156_10 NC_015445 3

Output of sir_graph (©)
mfold_util 4.7

Created Tue May 21 00:08:56 2019

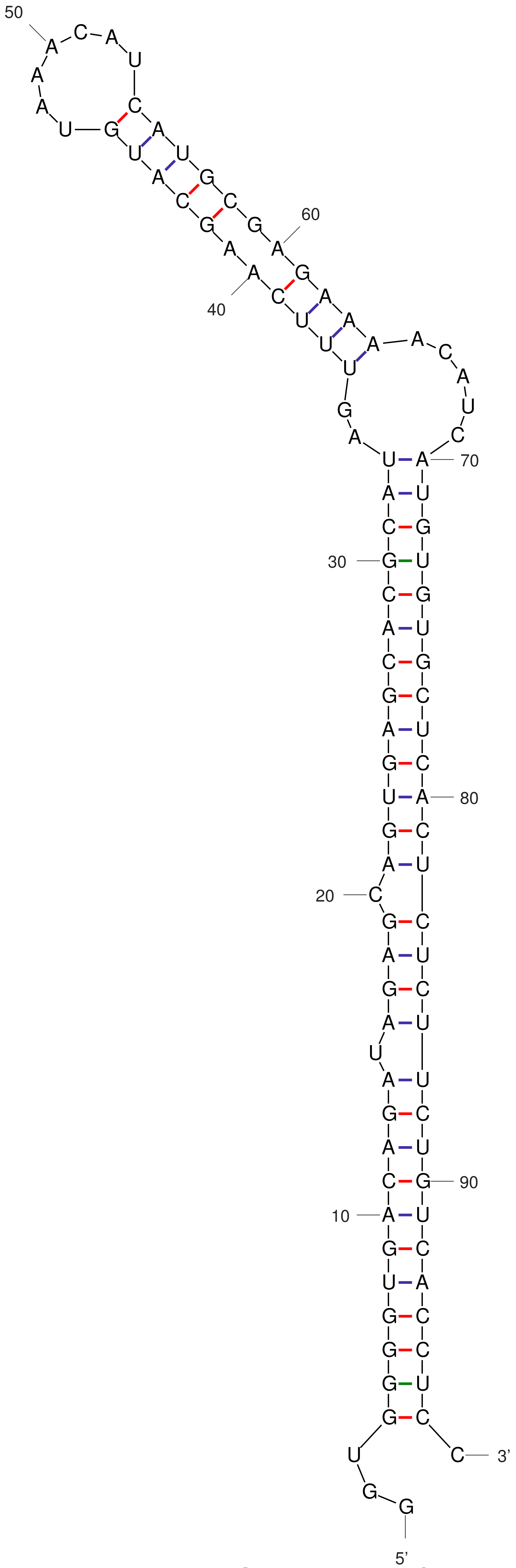

dG = -55.40 [Initially -55.40] Sl-miR156_11 NC_015445 3

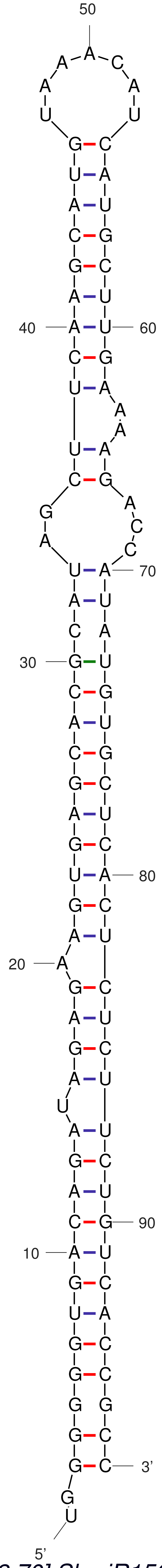

dG = -53.70 [Initially -53.70] Sl-miR156_12 NC_015445 3

Output of sir_graph (©)
mfold_util 4.7

Created Tue May 21 00:09:09 2019

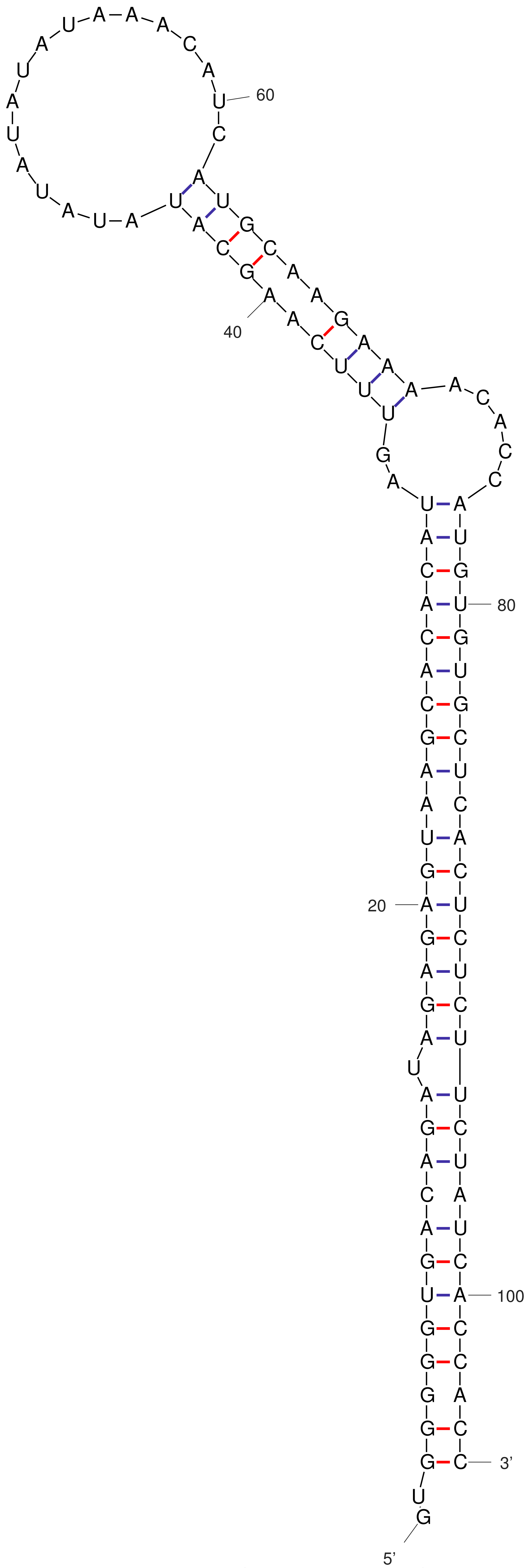

dG = -40.40 [Initially -40.40] Sl-miR156_13 NC_015445 3

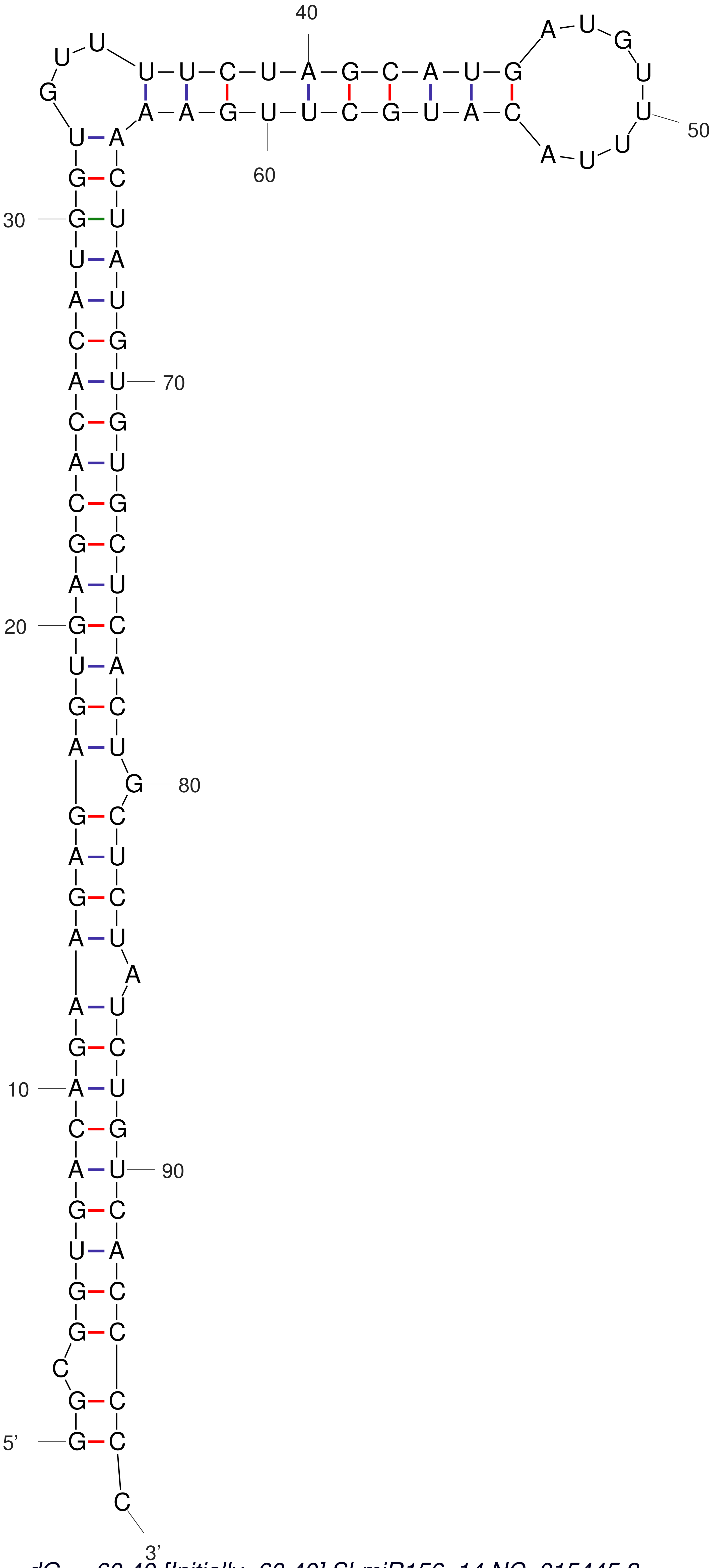

dG = -60.40 [Initially -60.40] Sl-miR156_14 NC_015445 3

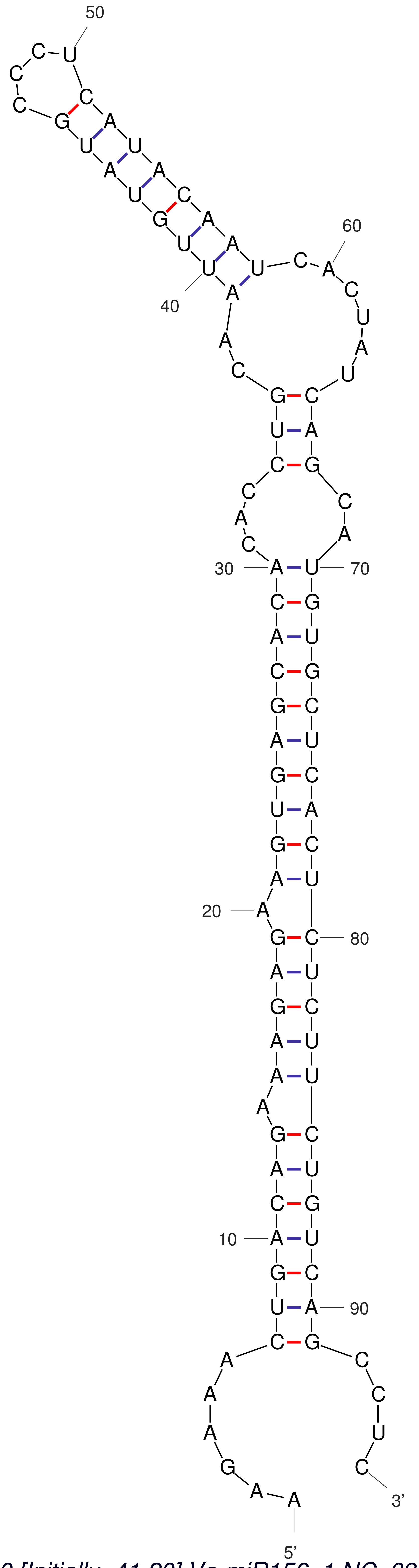

dG = -41.20 [Initially -41.20] Va-miR156_1 NC_030637 1

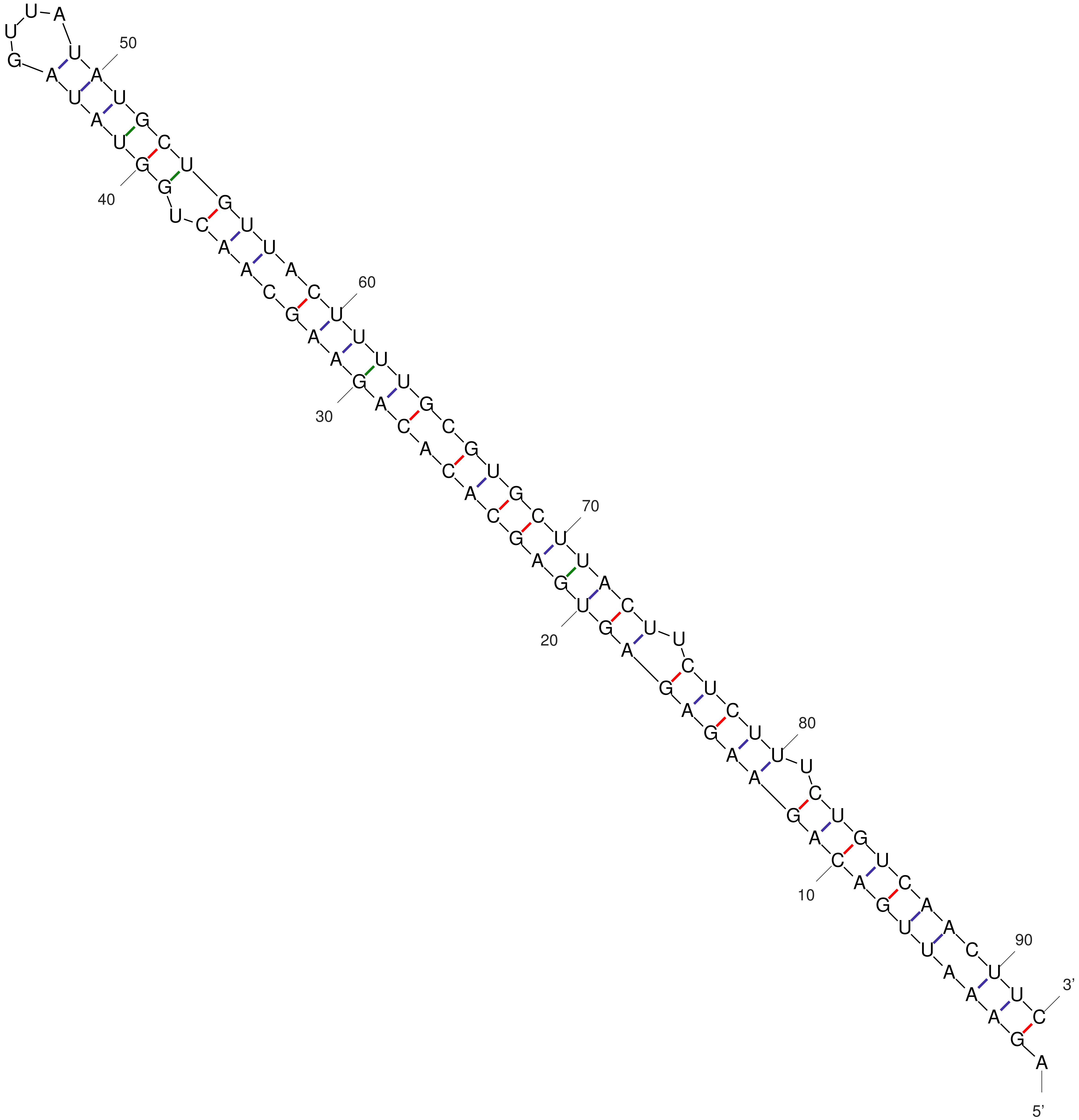

dG = -45.20 [Initially -45.20] Va-miR156_2 NC_030640 1

Output of sir_graph (©)
mfold_util 4.7

Created Tue May 21 00:00:24 2019

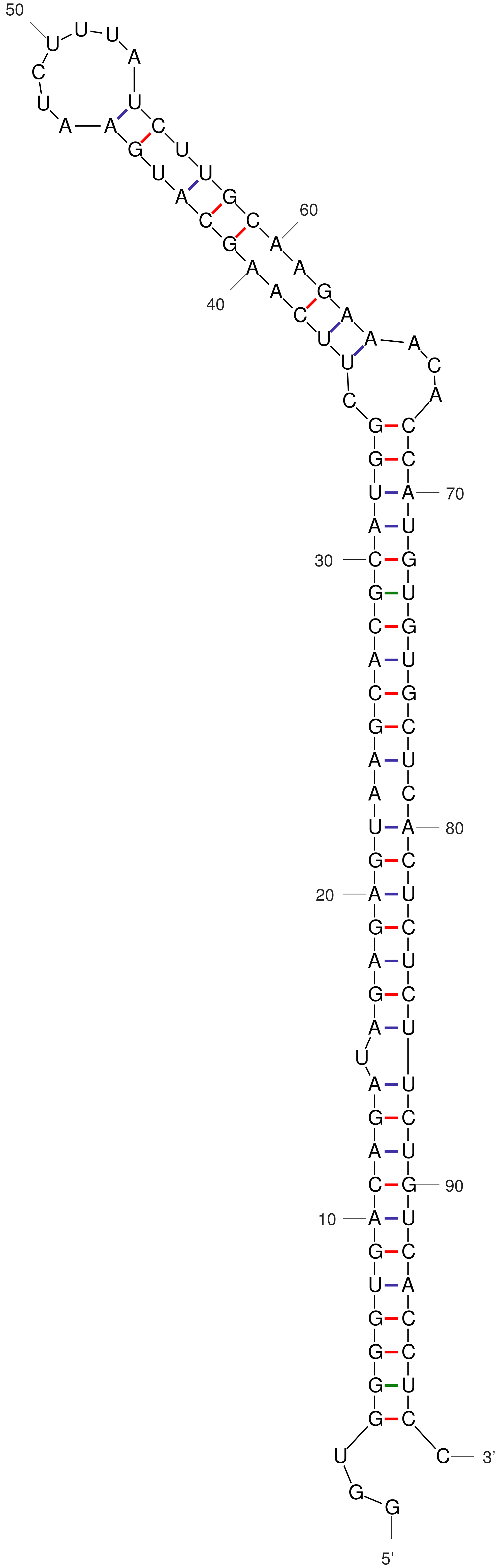

dG = -54.00 [Initially -54.00] Va-miR156_3 NC_030640 1

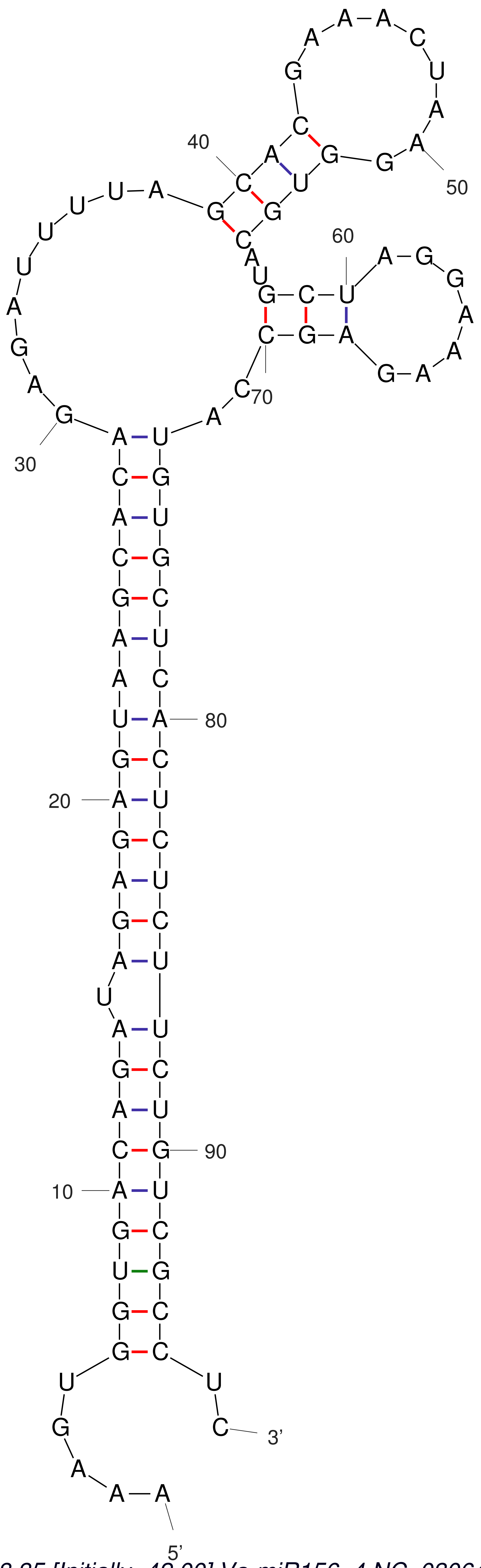

dG = -38.35 [Initially -42.00] Va-miR156_4 NC_030643 1

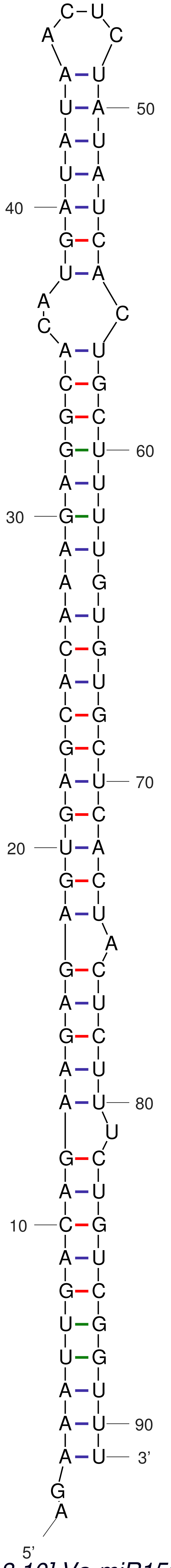

dG = -48.10 [Initially -48.10] Va-miR156_5 NC_030643 1

Output of sir_graph (©)
mfold_util 4.7

Created Tue May 21 00:03:29 2019

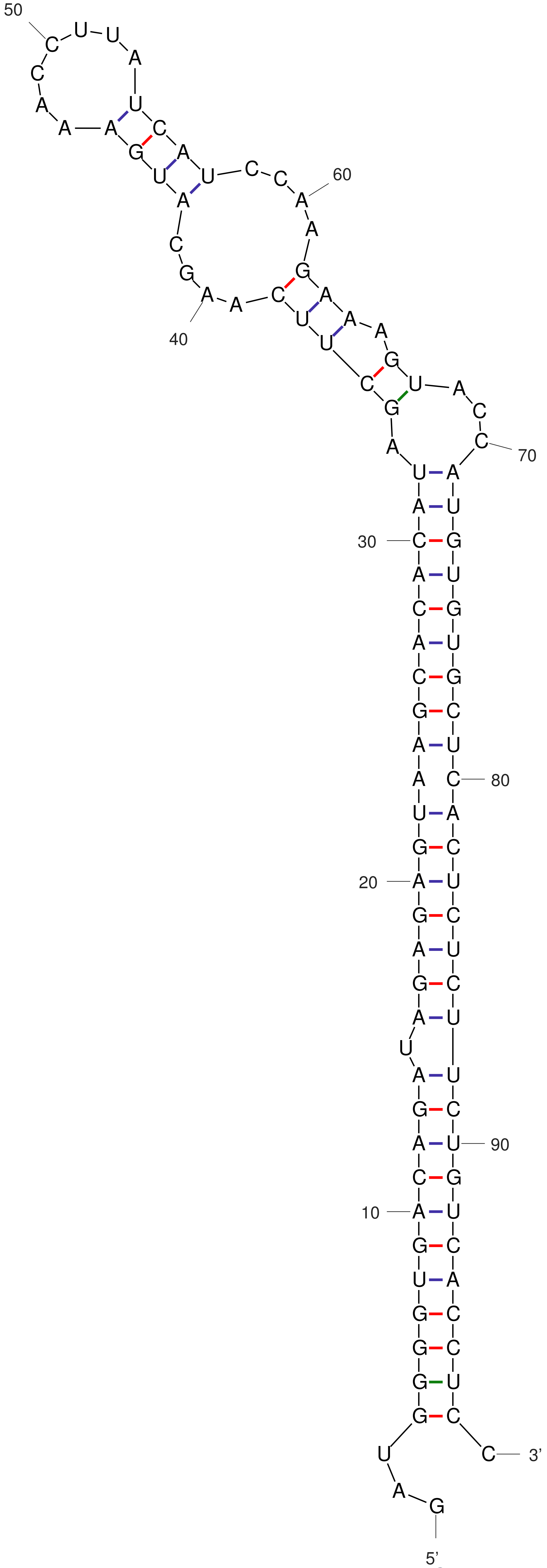

dG = -46.20 [Initially -46.20] Va-miR156_6 NC_030643 1

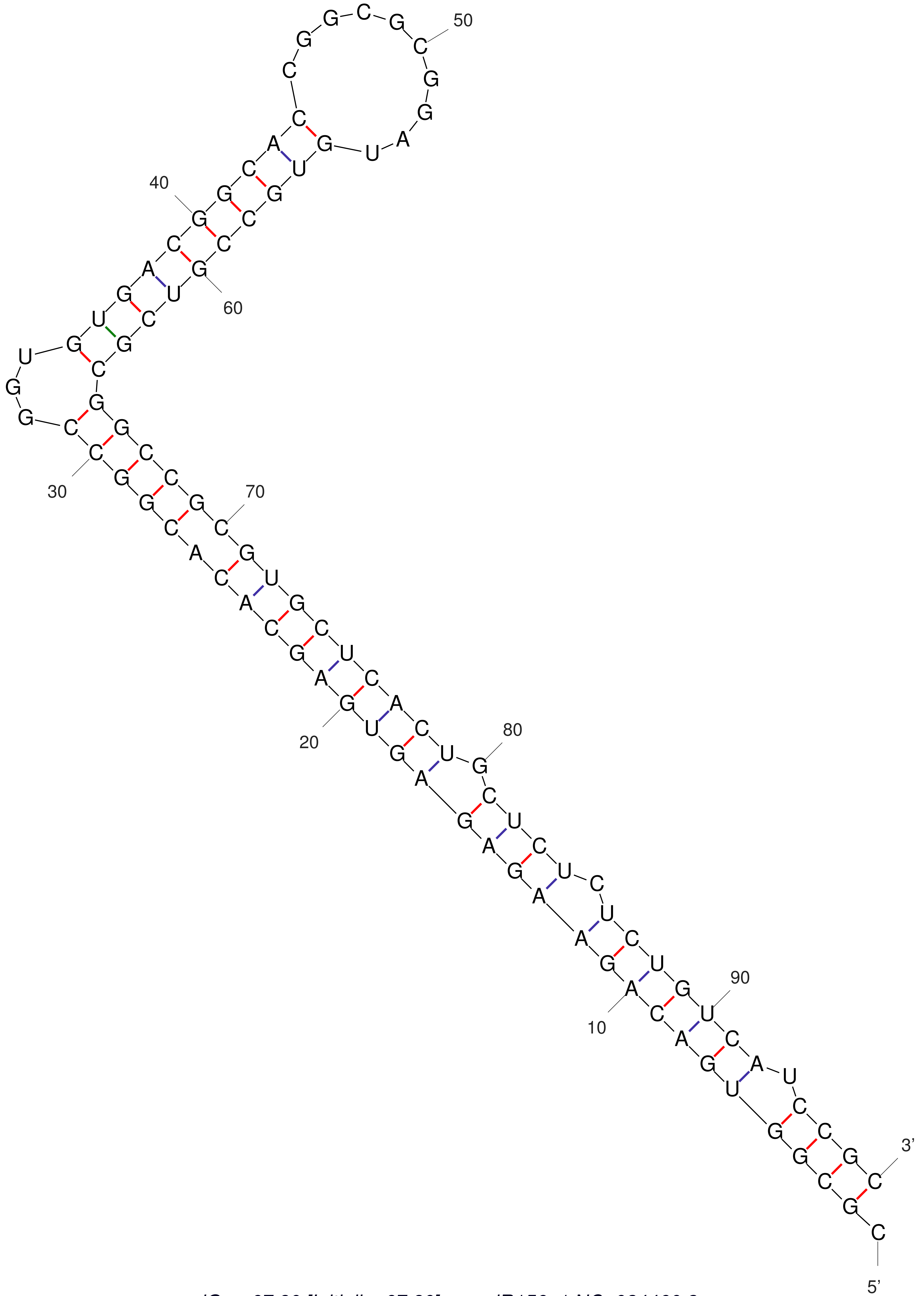

dG = -67.80 [Initially -67.80] zm-miR156_1 NC_024460 2

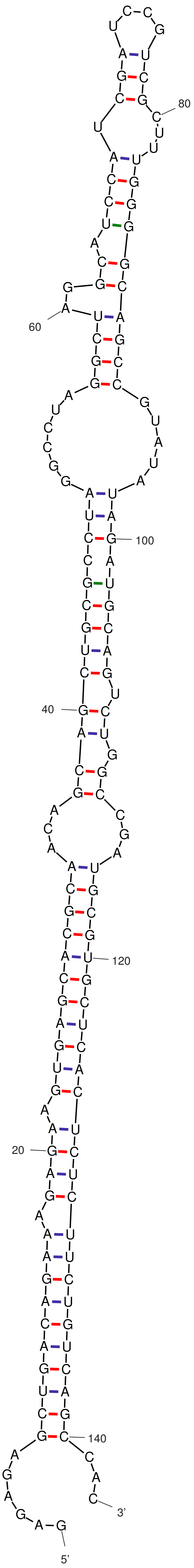

dG = -69.50 [Initially -69.50] zm-miR156_2 NC_024460 2

Output of sir_graph (©)
mfold_util 4.7

Created Mon Jul 22 08:57:36 2019

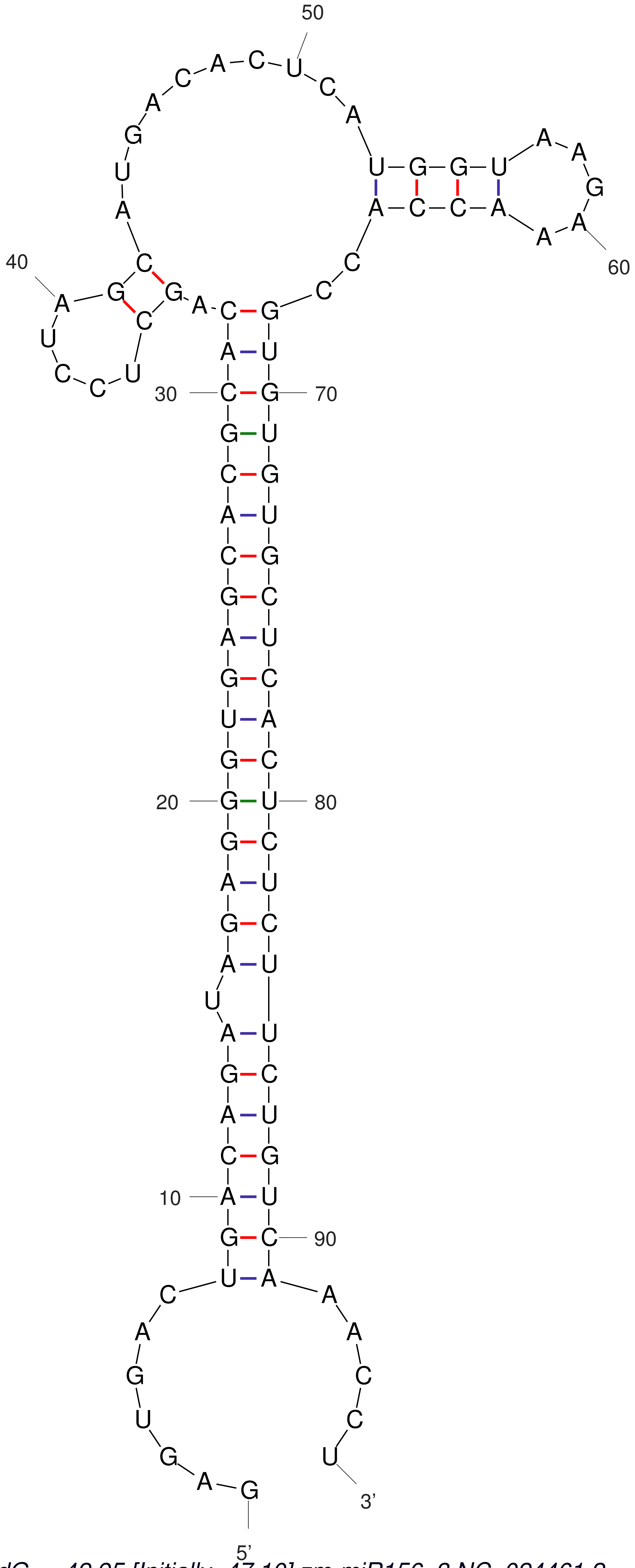

dG = -42.95 [Initially -47.10] zm-miR156_3 NC_024461 2

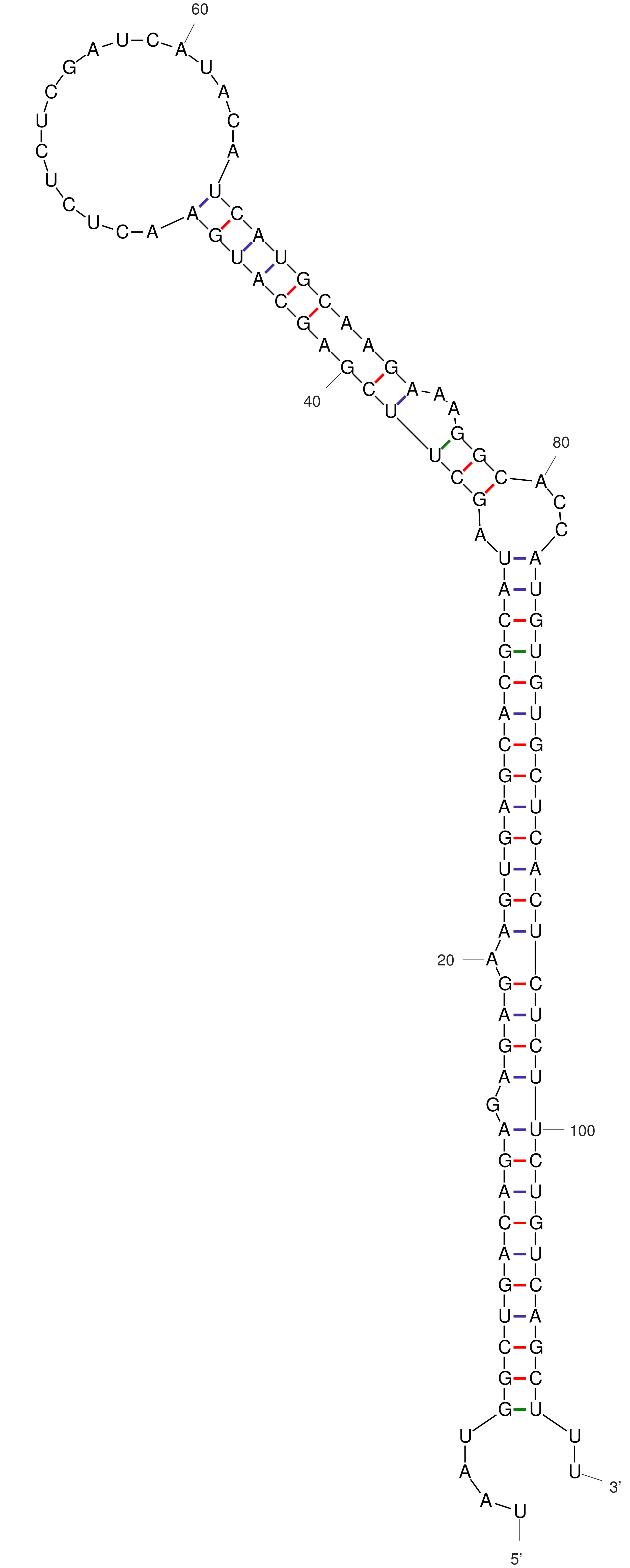

dG = -53.40 [Initially -53.40] zm-miR156_4 NC_024461 2

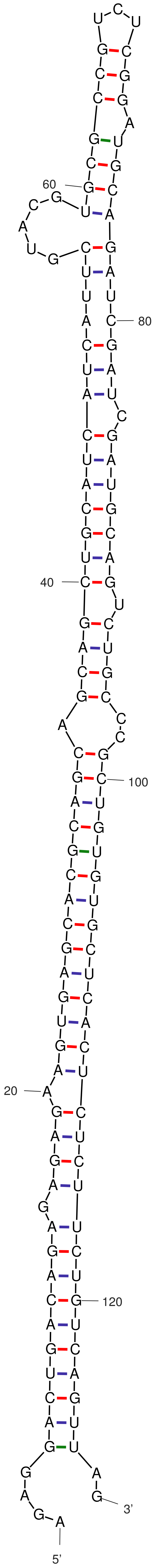

dG = -74.60 [Initially -74.60] zm-miR156_5 NC_024462 2

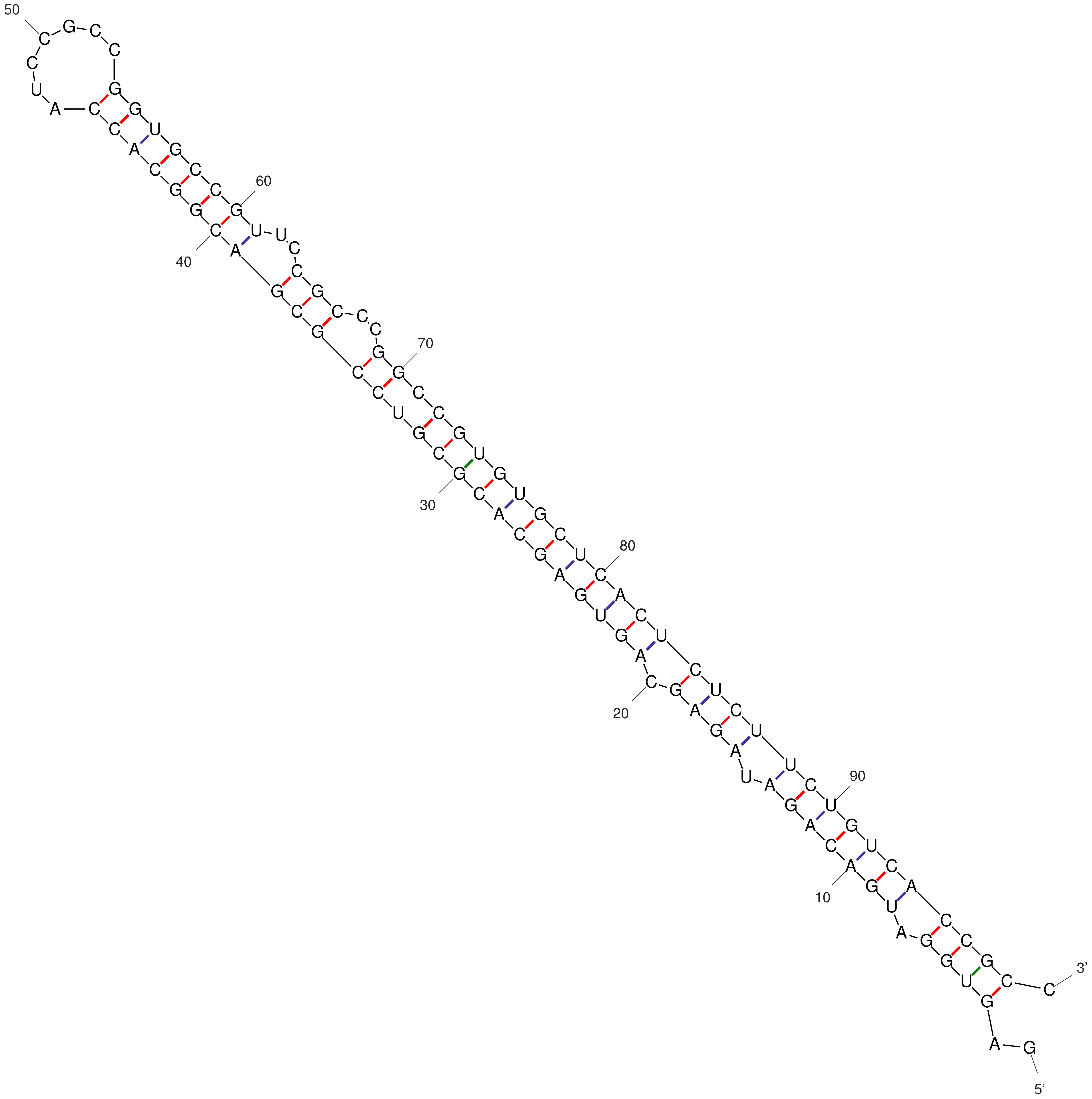

dG = -64.20 [Initially -64.20] zm-miR156_6 NC_024462 2

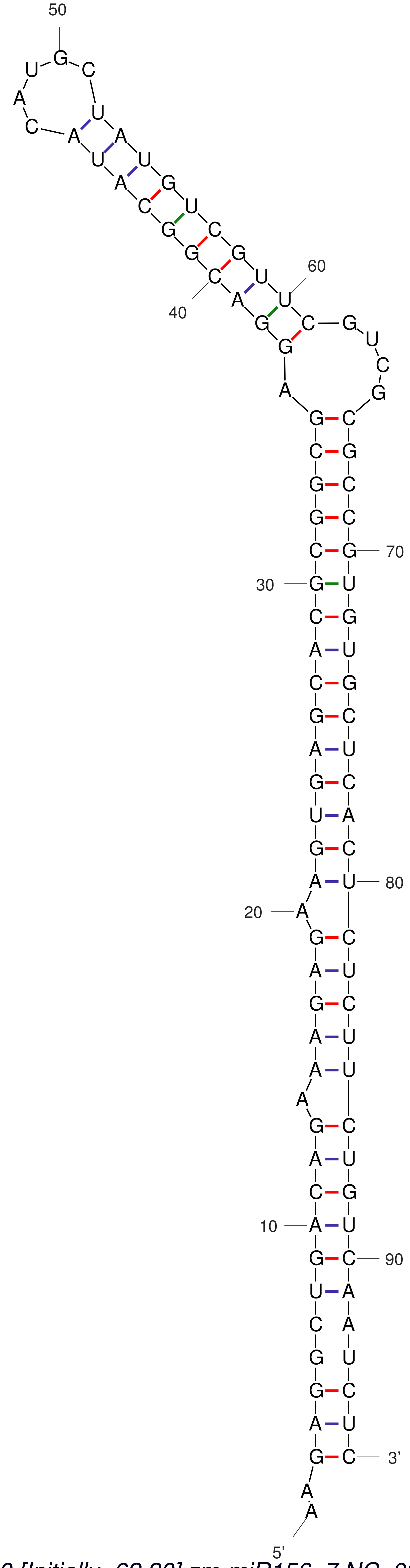

dG = -62.30 [Initially -62.30] zm-miR156_7 NC_024463 2

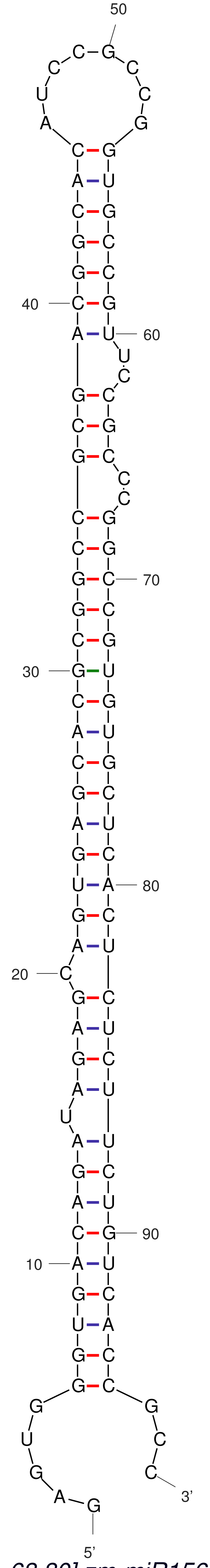

dG = -68.80 [Initially -68.80] zm-miR156_8 NC_024463 2

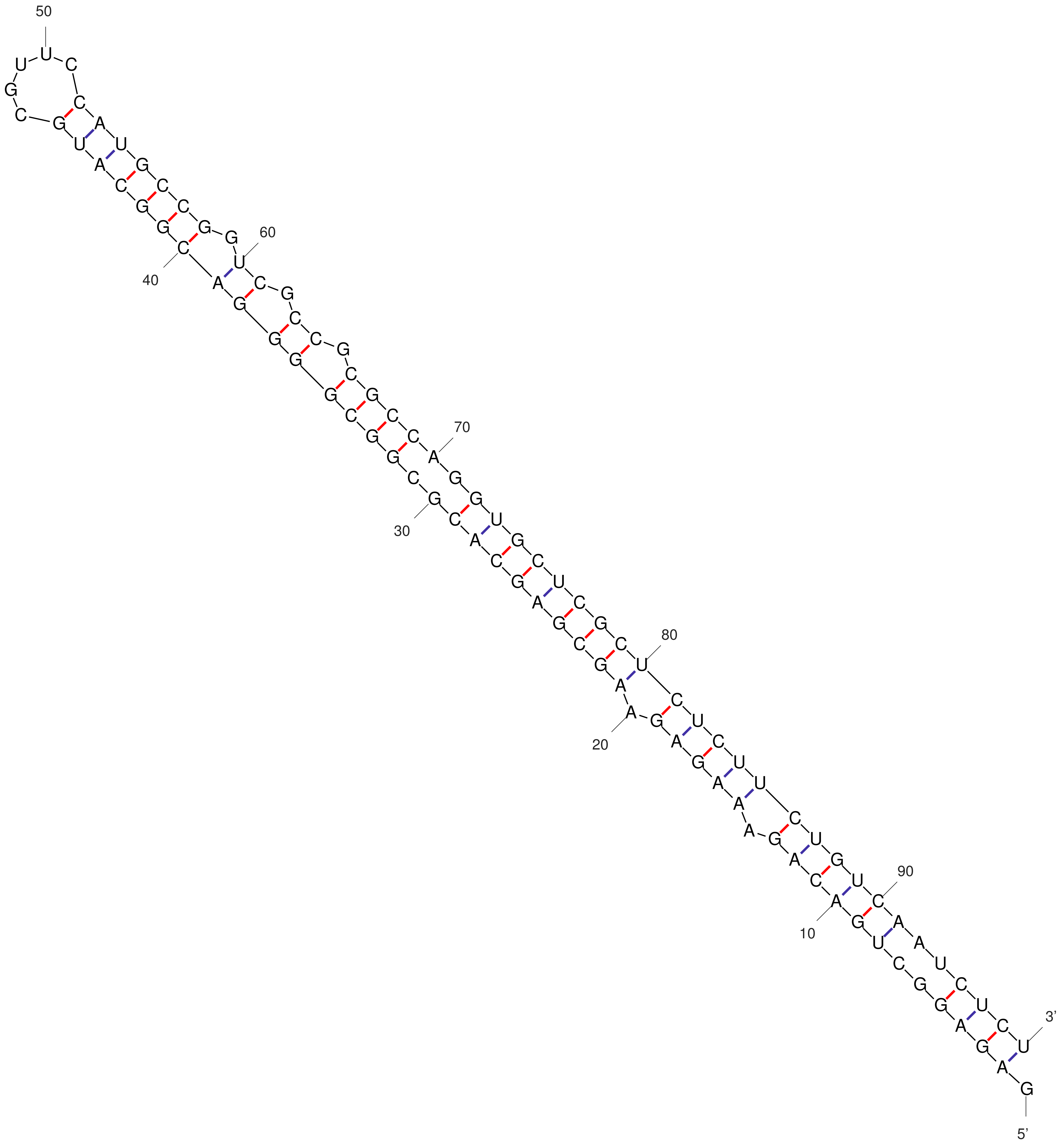

dG = -63.30 [Initially -63.30] zm-miR156_9 NC_024464 2

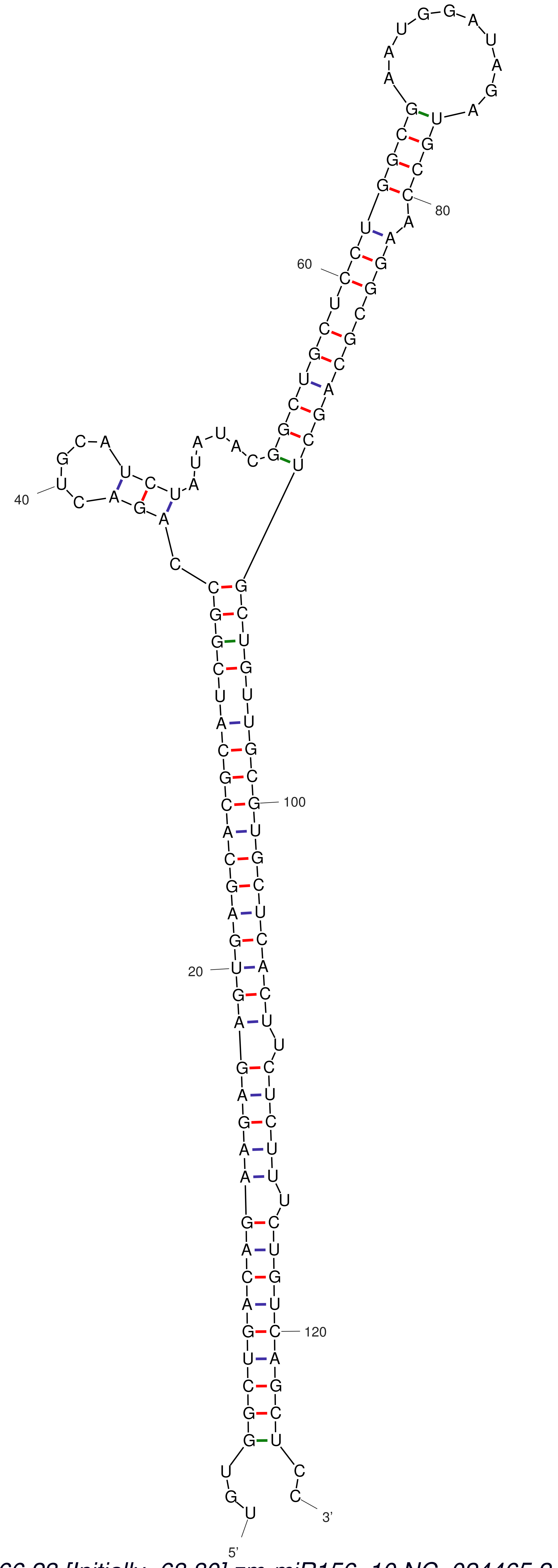

dG = -66.23 [Initially -68.80] zm-miR156_10 NC_024465 2

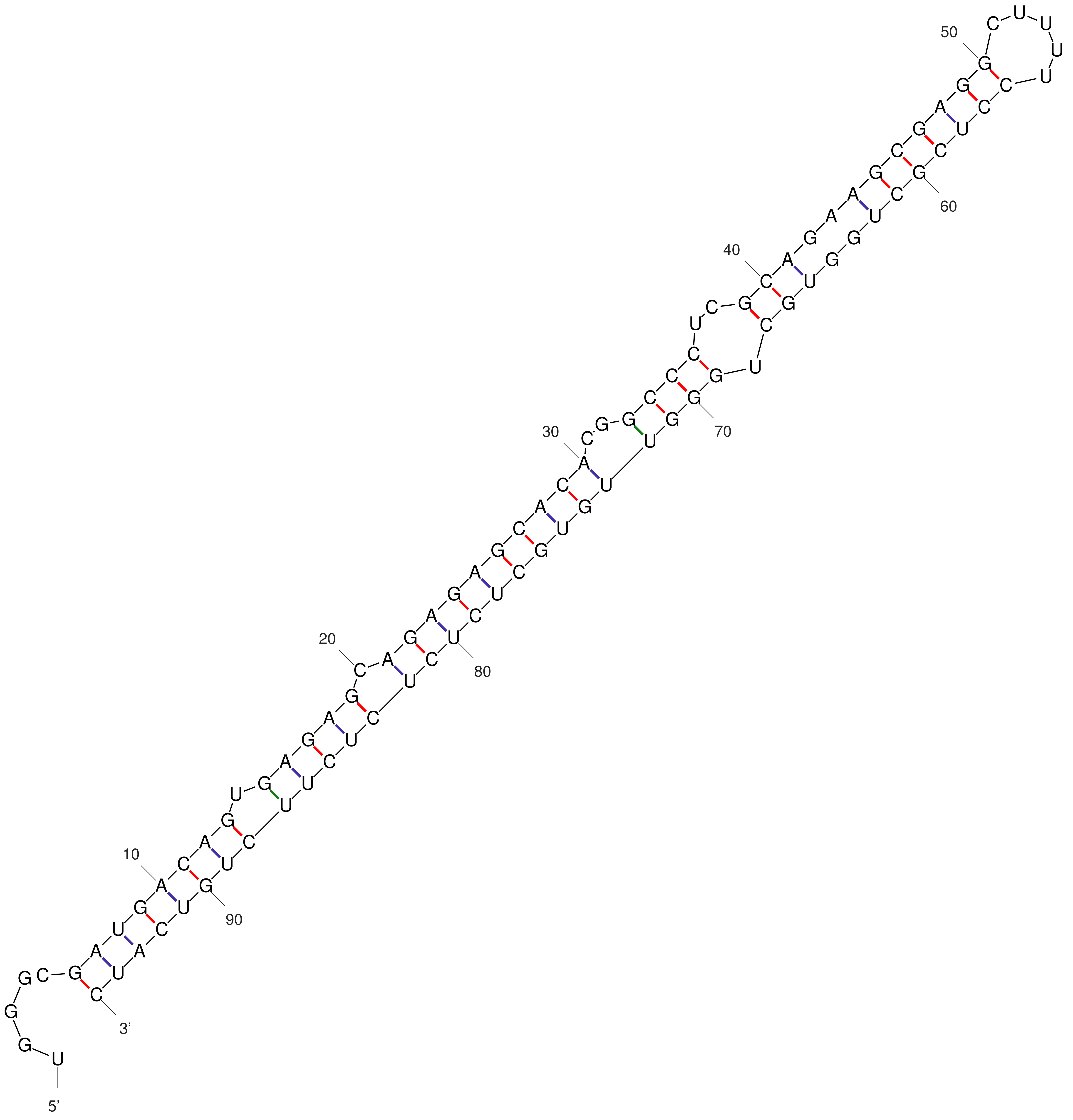

dG = -58.20 [Initially -58.50] zm-miR156_11 NC_024465 2

Output of sir_graph (©)
mfold_util 4.7

Created Mon Jul 22 09:00:12 2019

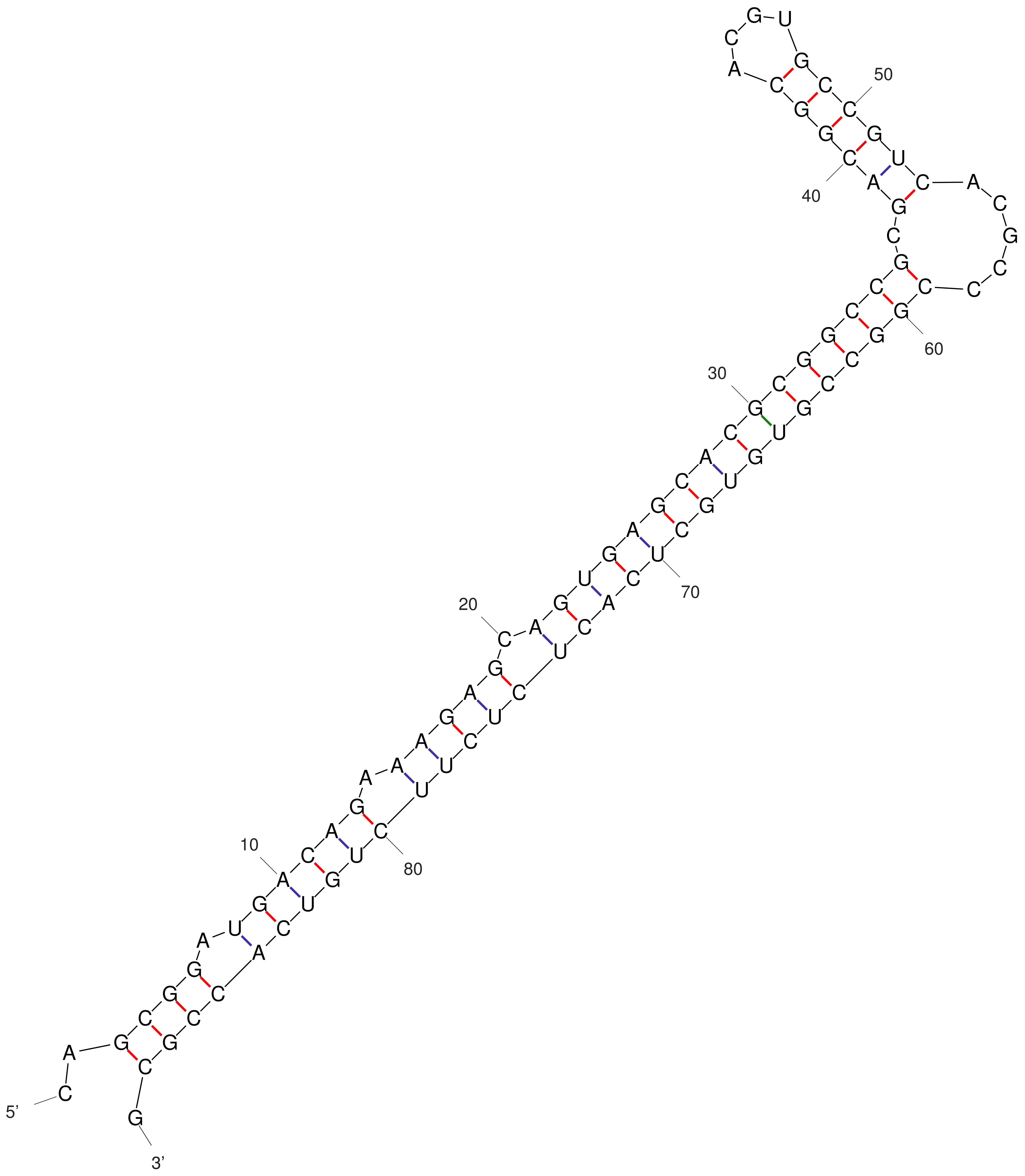

dG = -68.20 [Initially -68.20] zm-miR156_12 NC_024468 2
