# Supplementary material for: Dynamic Expansion and Functional Evolutionary Profiles of Plant Conservative Gene Family SBP-Box in Twenty Two Flowering Plants and the Origin of miR156
Source: Biomolecules. 2020 May 13;10(5):757. doi: 10.3390/biom10050757 (PMC7277735; doi:10.3390/biom10050757)
Supplement: Supplementary file 1 [file biomolecules-10-00757-s001.zip › Supplementary Materials/Figure S7.pdf]

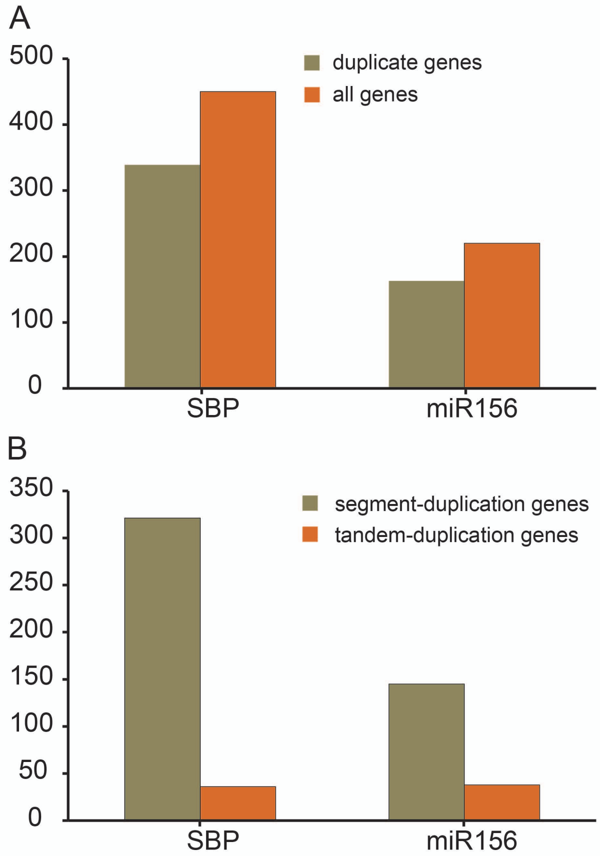

Figure S7. The number and type of duplicated genes. (A) The duplication gene number of SBP and miR156, and all gene number of SBP and miR156 that mapped on chromosome. (B) The number of segment-duplication gene of SBP and miR156, and the number of tandem-duplication gene of SBP and miR156.
