# Supplementary material for: Dynamic Expansion and Functional Evolutionary Profiles of Plant Conservative Gene Family SBP-Box in Twenty Two Flowering Plants and the Origin of miR156
Source: Biomolecules. 2020 May 13;10(5):757. doi: 10.3390/biom10050757 (PMC7277735; doi:10.3390/biom10050757)
Supplement: Supplementary file 1 [file biomolecules-10-00757-s001.zip › Supplementary Materials/Figure S8.pdf]

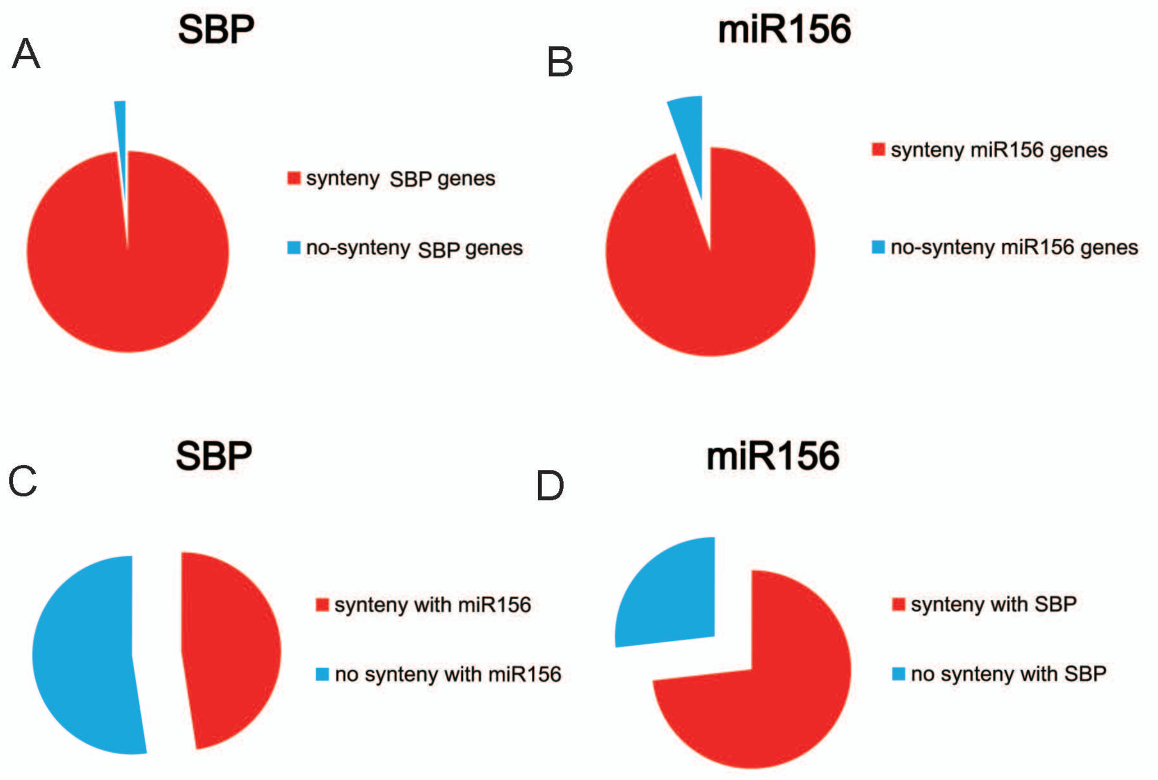

Figure S8. The number of genes in synteny relationships. (A) The number of SBP genes with synteny and without synteny relationships. (B) The number of miR156 with synteny and without synteny relationships. (C) The number of SBP genes with and without synteny relationships with miR156. (D) The number of miR156 with and without synteny relationships with SBP.
