# Supplementary material for: Dynamic Expansion and Functional Evolutionary Profiles of Plant Conservative Gene Family SBP-Box in Twenty Two Flowering Plants and the Origin of miR156
Source: Biomolecules. 2020 May 13;10(5):757. doi: 10.3390/biom10050757 (PMC7277735; doi:10.3390/biom10050757)
Supplement: Supplementary file 1 [file biomolecules-10-00757-s001.zip › Supplementary Materials/Figure S9.pdf]

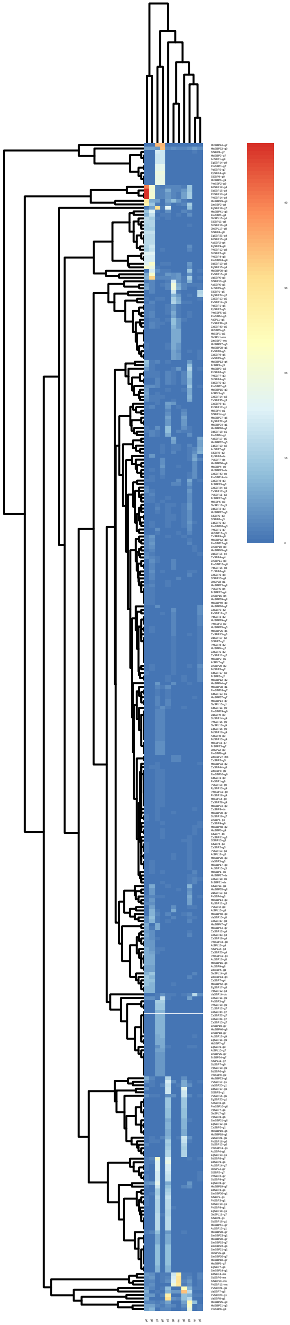

Figure S9. The heatmap clustering of synteny *SBP* genes and groups. The gene clustering was showed in the left and the groups clustering was showed on the top. The gene ID was showed in the right, and the corresponding group of each gene was marked after each gene ID.
