# Supplementary figures and images for: Dynamic Expansion and Functional Evolutionary Profiles of Plant Conservative Gene Family SBP-Box in Twenty Two Flowering Plants and the Origin of miR156
Source: Biomolecules. 2020 May 13;10(5):757. doi: 10.3390/biom10050757 (PMC7277735; doi:10.3390/biom10050757)

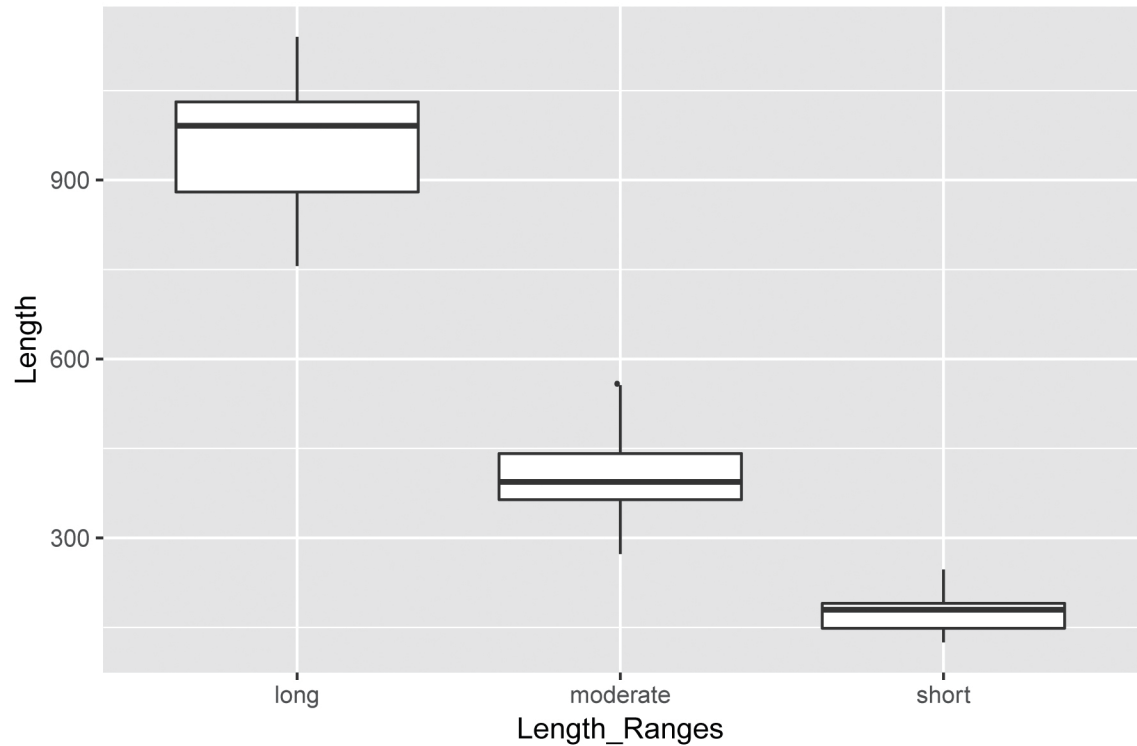

Figure S3. The three length ranges of SBP proteins.

Supplement: Supplementary file 1 [file biomolecules-10-00757-s001.zip › Supplementary Materials/Figure S3.pdf]

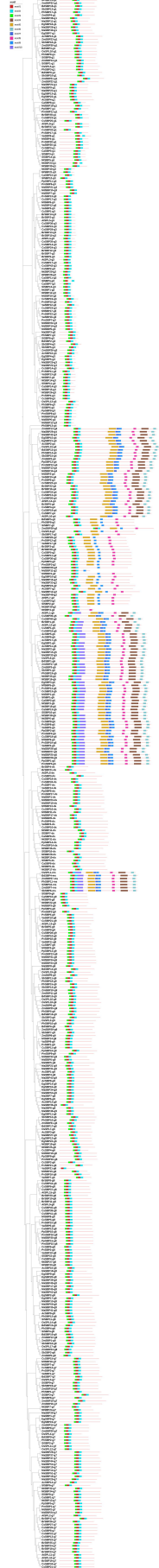

Figure S4. The conservative motifs for each SBP protein.

Supplement: Supplementary file 1 [file biomolecules-10-00757-s001.zip › Supplementary Materials/Figure S4.pdf]

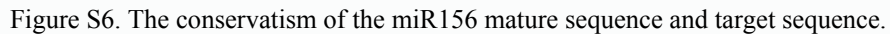

Supplement: Supplementary file 1 [file biomolecules-10-00757-s001.zip › Supplementary Materials/Figure S6.pdf]
